# Rh(I)-Catalyzed Modular Synthesis of Axially Chiral Alkylidene Azacycloalkanes

**DOI:** 10.1021/acscentsci.5c00232

**Published:** 2025-05-11

**Authors:** Yang Chen, Jiayan Chen, Shifa Zhu

**Affiliations:** † Key Laboratory of Functional Molecular Engineering of Guangdong Province, School of Chemistry and Chemical Engineering, 26467 South China University of Technology, Guangzhou 510640, China; ‡ State Key Laboratory of Bio-based Fiber Materials, School of Chemistry and Chemical Engineering, Zhejiang Sci-Tech University, Hangzhou 310018, China; § State Key Laboratory of Elemento-Organic Chemistry, Nankai University, Tianjin 300071, China

## Abstract

The structural prevalence of chiral *N*-bridged cyclic scaffolds in pharmacologically relevant substances originates from their multifaceted biofunctional capacities, particularly in privileged natural product architectures. Despite advances in central chirality, the precise and expeditious construction of axially chiral *N*-bridged cyclic scaffolds with impeccably full enantiocontrol and highly structural diversity remains largely underexploited due to the lack of efficient synthetic methods. Here, we disclose an unprecedented Rh­(I)/diene-catalyzed carbene coupling reaction of arylboronic acid with β-hydroxy α-diazocarbonyl compounds through a remotely controlled desymmetrization strategy, furnishing a diverse array of three-dimensional nonatropisomeric axially chiral alkylidene *N*-bridged [3.2.1] and [3.3.1] ring systems. This straightforward methodology operates without redox requirements, demonstrating extensive applicability across diverse substrates (>50 examples) while maintaining exceptional control of chemo- and enantioselectivity (up to 97% yield, mostly 95–99% ee). The synthetic utility of these compounds was further validated via sequential elaboration processes, allowing for modular assembly of novel *N*-bridged bicyclic systems with configurationally defined axial chirality.

## Graphical abstract

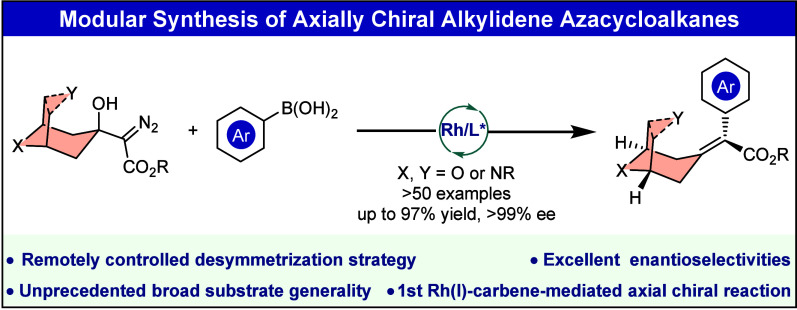

## Introduction

Axially chiral compounds, characterized by stereoisomerism arising from the restricted spatial orientation of substituents around a stereogenic axis, have become pivotal structural motifs in contemporary chemistry. These chiral architectures demonstrate versatile applications across multiple domains, including natural product synthesis,^1^ bioactive compound development,^2^ and advanced material design,^3^ and notably serve as privileged scaffolds for asymmetric catalysis and chiral ligand construction.^4^ In the last several decades, immense research efforts have been devoted in this area on both exploring synthetic strategies and practical utility of axially chiral compounds.^5–12^

However, current investigations remain predominantly centered on the catalytic asymmetric synthesis and application of established axially chiral architectures, particularly focusing on atropisomers^13–16^ and allenes (Figure 1A, left).^17–19^ Diverging markedly from conventional atropisomeric systems, axially chiral alkylidene cyloalkanes (ACACs) represent a distinct stereochemical paradigm characterized by a nonclassical stereogenic axis. Despite their considerable potential, they have suffered from persistent neglect in synthetic methodology development, and the enantioselective synthesis of these structures remains an intractable challenge. The first foray into catalytic asymmetric assembly of this unique structure can be traced back to 1988 when Fiaud and co-workers demonstrated his pioneering investigations on the enantioselective synthesis of 4-alkylcyclohexylidene derivatives enabled by asymmetric palladium catalysis, albeit with poor enantiocontrol.^20^ More recently, compelling progress has been achieved for efficient acquiring of these scaffolds.^21–30^ Despite such advances, most investigations in this realm have focused on a trisubstituted cyclohexylidene core (Figure 1A, right).^31,32^ Given the unique chiroptical property of ACACs as medically active molecules and their potential applications in other fields,^33–35^ the development of general and efficient strategies for synthesizing these axially chiral structures remains a high priority in the organic synthesis community. However, significant challenges persist, particularly in achieving high enantioselectivity and diversifying the structural scaffolds of these compounds.

**Figure 1:**
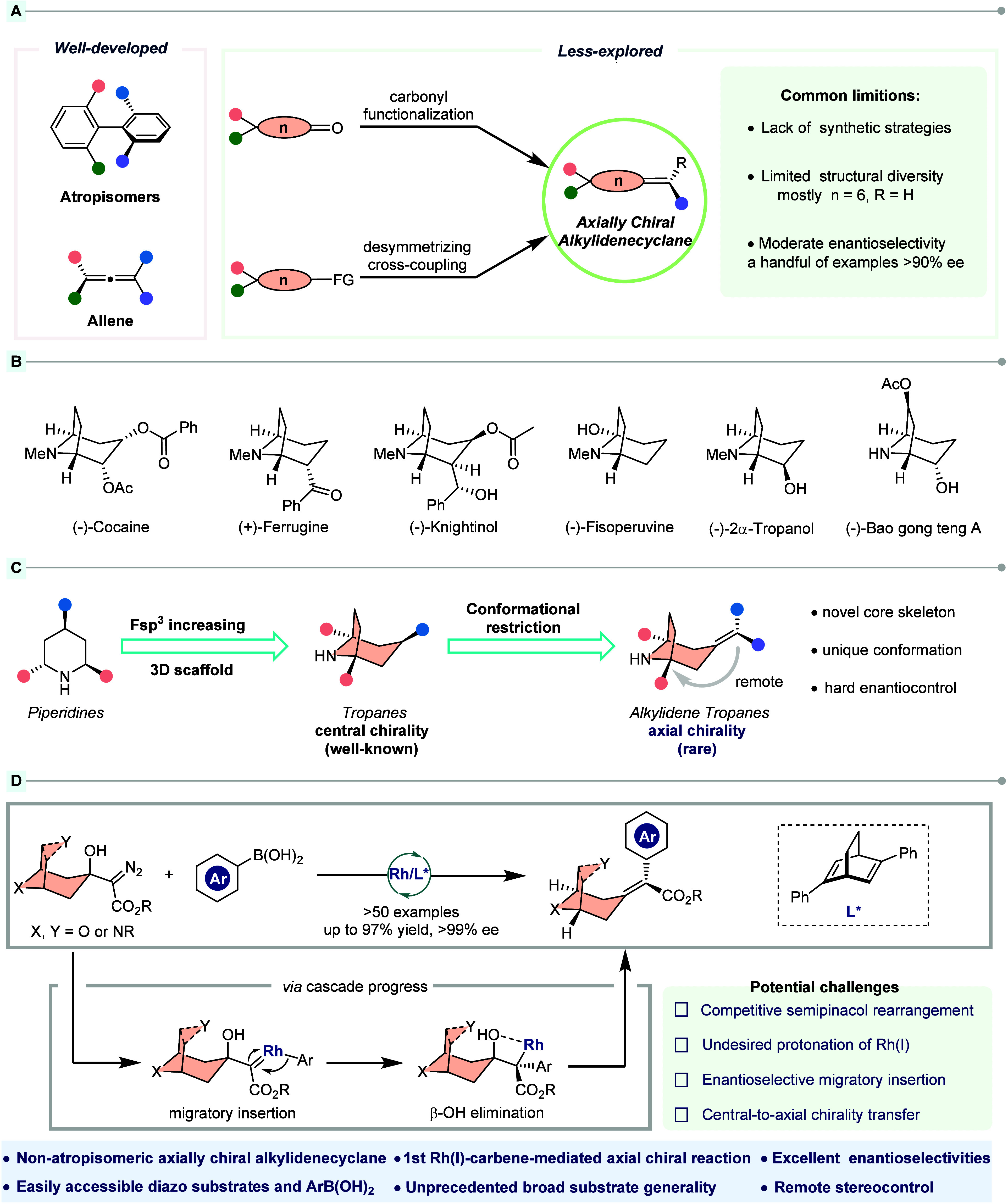
Background and project synopsis. (A) State of the art for the synthesis of axially chiral skeletons. (B) Representative chiral *N*-bridged [3.2.1] cyclic molecules. (C) Construction of chiral conformational restricted tropane skeletons. (D) This work: Rh­(I)-catalyzed modular synthesis of axially chiral alkylidene azacycloalkanes. Abbreviations: FG, functional group; Ar, aryl. F­(sp^3^), fraction of sp^3^-hybridized carbons.

In the fields of synthetic and medicinal chemistry, chiral *N*-bridged [3.2.1] cyclic scaffolds, particularly tropane-type molecules, are prevalent in natural products and bioactive compounds due to their diverse and significant biological activities, such as (−)-cocaine, (+)-ferrugine, and (−)-knightinol (Figure 1B).^36,37^ Meanwhile, these tropane-based skeletons often function as classical isosteric replacements for substituted piperidines (Figure 1C).^38^ Of note, such molecular architectures pose a long-standing and formidable synthetic challenge to organic chemists, arising not only from their conformational rigidity and stereochemical complexity but also from the absence of efficient catalytic systems capable of addressing these inherent issues. Notably, limited catalytic strategies have been developed to date for the efficient asymmetric synthesis of these motifs, with reported examples including metal-catalyzed cyclization^39,40^ and NHC-enabled double Mannich addition,^41^ among others.^42–46^ Besides, the current research about chiral tropanes so far has been focused on central chirality; the catalytic asymmetric construction of axial chiral tropanes still remains largely underexploited. Thus far, only a single strategy involving chiral phosphoric-acid-catalyzed condensation of cyclic ketones and hydroxylamines has been ingeniously established by the Liu and Shi groups to access chiral tropinone-based oxime esters.^47,48^ In structural terms, these reports generally involved a heteroatom nitrogen in the core structures, providing a handle for the control of the enantioselectivity. Recently, Bernhard and co-workers showed that by replacing the parent piperidine ring of eliprodil with an enantiomerically pure benzylidenetropane unit which was separated by chiral HPLC, marked improvement in the σ1 antagonistic activity could be achieved, clearly highlighting the promising avenue of axially chiral alkylidene tropanes in medicinal chemistry.^49^ However, the lack of an interaction site and the remote distance of the four chirality-related groups or atoms at the two remote ends of the chiral axis render the catalytic asymmetric synthesis of this structure highly challenging, hampering enantiodiscrimination (Figure 1C). Thus, the emerging exploration of understudied tropane-based axially chiral architectures aligns with broader efforts to diversify structural motifs and expand stereochemical possibilities in axial chirality.

Over the past few years, asymmetric catalytic Rh­(I)-mediated carbene transfer processes have enabled precise governance over central stereochemical configurations thanks to the Rh­(I)/chiral diene complexes as highly effective catalysts in modern organic synthesis.^50–61^ However, to the best of our knowledge, the potential of Rh­(I)–carbene intermediates to engage in axial chiral transformations has not yet been disclosed. In line with our interest in developing metal carbene chemistry and asymmetric catalysis,^62–69^ we proposed the utilization of readily accessible β-hydroxyl α-diazocarbonyl compounds to achieve the desired products through a desymmetrization strategy while maintaining remote control of the stereogenic axis. We envisioned that a Rh­(I)-catalyzed tandem asymmetric carbene migratory insertion/β-OH elimination reaction with arylboronic acid could be a viable approach to accomplish the desired transformation. Although this hypothesis sounds feasible, this endeavor involves a number of challenges. First, it is well-known that β-hydroxyl metal carbene intermediates are more inclined to undergo a semipinacol rearrangement to give an undesired ring expansion side product.^70–77^ Thus, the identification of a suitable catalytic system to promote carbene migratory insertion while suppressing 1,2-alkyl migration is crucial. Second, another major obstacle encountered in this study is controlling the enantioselectivity of 1,2-aryl migratory insertion of Rh­(I)-carbene.^78,79^ The inherent difficulty in achieving enantioselective migratory insertion reaction of an α-ester Rh­(I)-carbene lies in the presence of an oxa-π-allyl Rh­(I) intermediate, which may exchange with the base to form an enolate species, followed by protonation to afford the racemic product.^80^ Lastly, the challenge is to avoid racemization from central chirality to axial chirality via β-OH elimination to preserve the enantiopurity of the products. By overcoming these challenges, herein, we report an unprecedented Rh­(I)-catalyzed carbene coupling reaction of arylboronic acids with *N*-bridged cyclic ketone-derived α-diazocarbonyl compounds through a remotely controlled desymmetrization strategy. The strategic implementation of a *C*_2_-symmetric chiral diene ligand facilitated an asymmetric transformation under remarkably mild reaction conditions, successfully generating an extensive range of tetrasubstituted nonatropisomeric axially chiral 3D alkylidenecyclanes. This methodology demonstrated outstanding chemo- and enantiocontrol across a broad substrate scope (>50 examples), with most cases achieving 95–99% enantiomeric excess. Thus, this protocol not only features the first example of asymmetric synthesis of axially chiral alkylidene azacycloalkanes but also constitutes the first axially chiral transformation of Rh­(I)-carbene (Figure 1D).

## Results and Discussion

### Reaction Optimization

At the outset, the coupling reaction of tropinone-derived α-diazocarbonyl compounds (**1**) with *p*-tolylboronic acid (**2a**) was selected as the model reaction to verify our hypothesis. The relative stereochemistry of substrates is confirmed by single-crystal X-ray diffraction studies of **1b**. Its OH group is *trans* to the Boc-protected amino group (see the Supporting Information for details). As shown in Table 1, the reaction was conducted with [Rh­(NBD)­Cl]_2_ (2.5 mol %) as the catalyst and KO^*t*^Bu (1.1 equiv) as the base in methyl *tert*-butyl ether (MTBE) under a N_2_ atmosphere at room temperature for 12 h; the desired alkylidenecyclane product **3a** was obtained in 56% yield (Table 1, entry 1). Then, a systematic examination of various chiral ligands with [Rh­(C_2_H_4_)_2_Cl]_2_ as the catalyst was conducted (entries 2–9). A series of commercially available chiral phosphine ligands (**L1**–**L5**) was first investigated. Unfortunately, these ligands were ineffective for this reaction without any desired alkylidenecyclane product being detected. Gratifyingly, further examination revealed the superiority of Hayashi’s bicyclo[2.2.2]­octadiene ligand **L6** for this transformation, which produced the desired product **3a** in 55% yield with 53% ee (entry 7). This preliminary result implied that our design is feasible. To our delight, the enantioselectivity was improved to 97% ee by changing the ethyl ester of diazo to more sterically bulky *tert*-butyl ester and using mixed MTBE/DCM as the solvent; however, a large decrease of the reaction yield was also observed (entry 8). Switching to the opposite configurational diene ligand (*S*,*S*)-**L6**, the desired product **3b** was delivered in similar enantioselectivity with a poor yield (entry 9). Subsequently, an investigation of various *N*-protecting groups of the diazo substrates was carried out. The use of the *N*-Cbz-protected diazo substrate **1c** provided the desired axially chiral alkylidene tropane product **3c** in good yield (77%) with excellent enantioselectivity (97% ee) (entry 10). The higher enantioselectivity (99%) was obtained by using *N*-Bz-protected diazo substrate **1d** in 76% yield (entry 11). Employing the preformed chiral rhodium complex [Rh­(**L6**)­Cl]_2_ in the reaction delivered a comparable yield and identical enantioselectivity (entry 12). Evaluation of solvents returned MTBE and DCM as the more suitable ones (entries 13 and 14). Other base additives tended to give inferior results (entries 15–17). When the rhodium catalyst was excluded from the reaction system, no product formation was detected under otherwise identical conditions, confirming that rhodium species are indispensable for facilitating the catalytic transformation (entry 18).

**Table 1:**
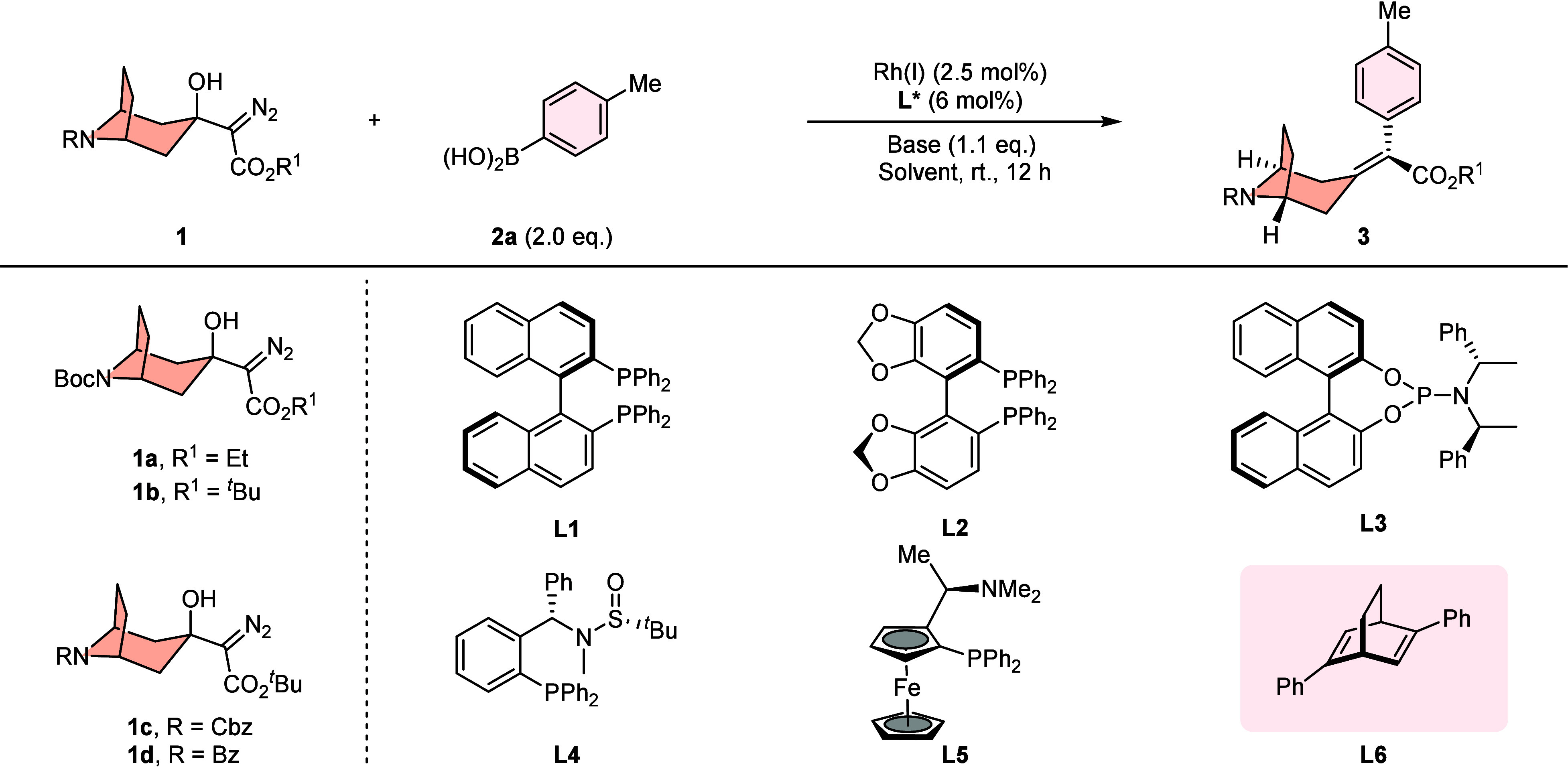
Optimization of the Reaction Conditions^a^

Entry	**1**	Rh catalyst (mol %)	Base	Solvent	**3**	Yield^b^ (%)	ee^c^ (%)
1	**1a**	[Rh(NBD)Cl]_2_	KO^*t*^Bu	MTBE	**3a**	56	
2	**1a**	[Rh] (2.5) + **L1** (6)	KO^*t*^Bu	MTBE	**3a**	ND^d^	
3	**1a**	[Rh] (2.5) + **L2** (6)	KO^*t*^Bu	MTBE	**3a**	ND^d^	
4	**1a**	[Rh] (2.5) + **L3** (6)	KO^*t*^Bu	MTBE	**3a**	ND^d^	
5	**1a**	[Rh] (2.5) + **L4** (6)	KO^*t*^Bu	MTBE	**3a**	ND^d^	
6	**1a**	[Rh] (2.5) + **L5** (6)	KO^*t*^Bu	MTBE	**3a**	ND^d^	
7	**1a**	[Rh] (2.5) + **L6** (6)	KO^*t*^Bu	MTBE	**3a**	55	55
8	**1b**	[Rh] (2.5) + **L6** (6)	KO^*t*^Bu	MTBE/DCM = 1:1	**3b**	39	97
9^e^	**1b**	[Rh] (2.5) + **L6** (6)	KO^*t*^Bu	MTBE/DCM = 1:1	**3b**	13	–96
10	**1c**	[Rh] (2.5) + **L6** (6)	KO^*t*^Bu	MTBE/DCM = 1:1	**3c**	77	97
11	**1d**	[Rh] (2.5) + **L6** (6)	KO^*t*^Bu	MTBE/DCM = 1:1	**3d**	76	99
12	**1d**	[Rh(**L6**)Cl]_2_ (2.5)	KO^*t*^Bu	MTBE/DCM = 1:1	**3d**	77	99
13	**1d**	[Rh(**L6**)Cl]_2_ (2.5)	KO^*t*^Bu	MTBE/THF = 1:1	**3d**	44	93
14	**1d**	[Rh(**L6**)Cl]_2_ (2.5)	KO^*t*^Bu	MTBE/Toluene = 1:1	**3d**	62	93
15	**1d**	[Rh(**L6**)Cl]_2_ (2.5)	Et_3_N	MTBE/DCM = 1:1	**3d**	ND^d^	
16	**1d**	[Rh(**L6**)Cl]_2_ (2.5)	KOH	MTBE/DCM = 1:1	**3d**	ND^d^	
17	**1d**	[Rh(**L6**)Cl]_2_ (2.5)	K_3_PO_4_	MTBE/DCM = 1:1	**3d**	ND^d^	
18	**1d**	none	KO^*t*^Bu	MTBE/DCM = 1:1	**3d**	ND^d^	

a Unless otherwise noted, all reactions were performed with **1** (0.1 mmol), **2a** (0.2 mmol), [Rh­(C_2_H_4_)_2_Cl]_2_ (2.5 mol %), L* (6 mol %), and KO^*t*^Bu (0.11 mmol) in 2 mL of solvent at room temperature for 12 h. Abbreviations: ^*t*^Bu, *tert*-butyl; Boc, *tert*-butoxycarbonyl; Cbz, carboxybenzyl; Bz, benzoxyl; NBD, norbornadiene.

b Isolated yield.

c Determined by chiral HPLC.

d Not detected.

e The opposite configurational diene ligand was used.

### Substrate Scope

Having established the optimal reaction conditions (Table 1, entry 12), we then evaluated the substrate scope of this Rh­(I)-catalyzed desymmetrizing carbene coupling reaction (Figure 2). First, we explored various arylboronic acids as coupling partners for this transformation. Gratifyingly, a broad range of (hetero)­aryl boronic acids with diverse functional groups showed good compatibility; for instance, alkyls (**3d**, **3f**, **3g**, and **3r**), aryls (**3s**), (thio)-alkoxys (**3i**, **3j**, **3l**, and **3t**), halogen (**3m**–**3o**, **3u**, and **3v**), TMS (**3h**), CF_3_ (**3p**), CO_2_Me (**3q**), and OCF_3_ (**3w**) can be preassembled into aryl boronic acid and lead to the production of the corresponding alkylidene tropanes with good yields (53–84%) and excellent enantioselectivities (93–99% ee). Disubstituted aromatic boronic acids were also feasible substrates for conversion (**3x** and **3y**). Particularly, ortho-substituted arylboronic acids afforded products with 50–94% yields and 94–98% ees, demonstrating that steric hindrance minimally impacted the whole reaction efficiency and stereoselectivities (**3z**, **3aa**, **3ae**, and **3af**). As expected, the fused-ring aromatic boronic acids were compatible with those transformations (**3ab**–**3ad**). Notably, heteroaromatic boronic acids including indole (**3ag**), carbazole (**3ah**), dibenzofuran (**3ai** and **3ak**), and others (**3aj**, **3al**, and **3am**) participated in the reaction smoothly to deliver the corresponding alkylidene tropane products, which showcased the robustness of this method.

**Figure 2:**
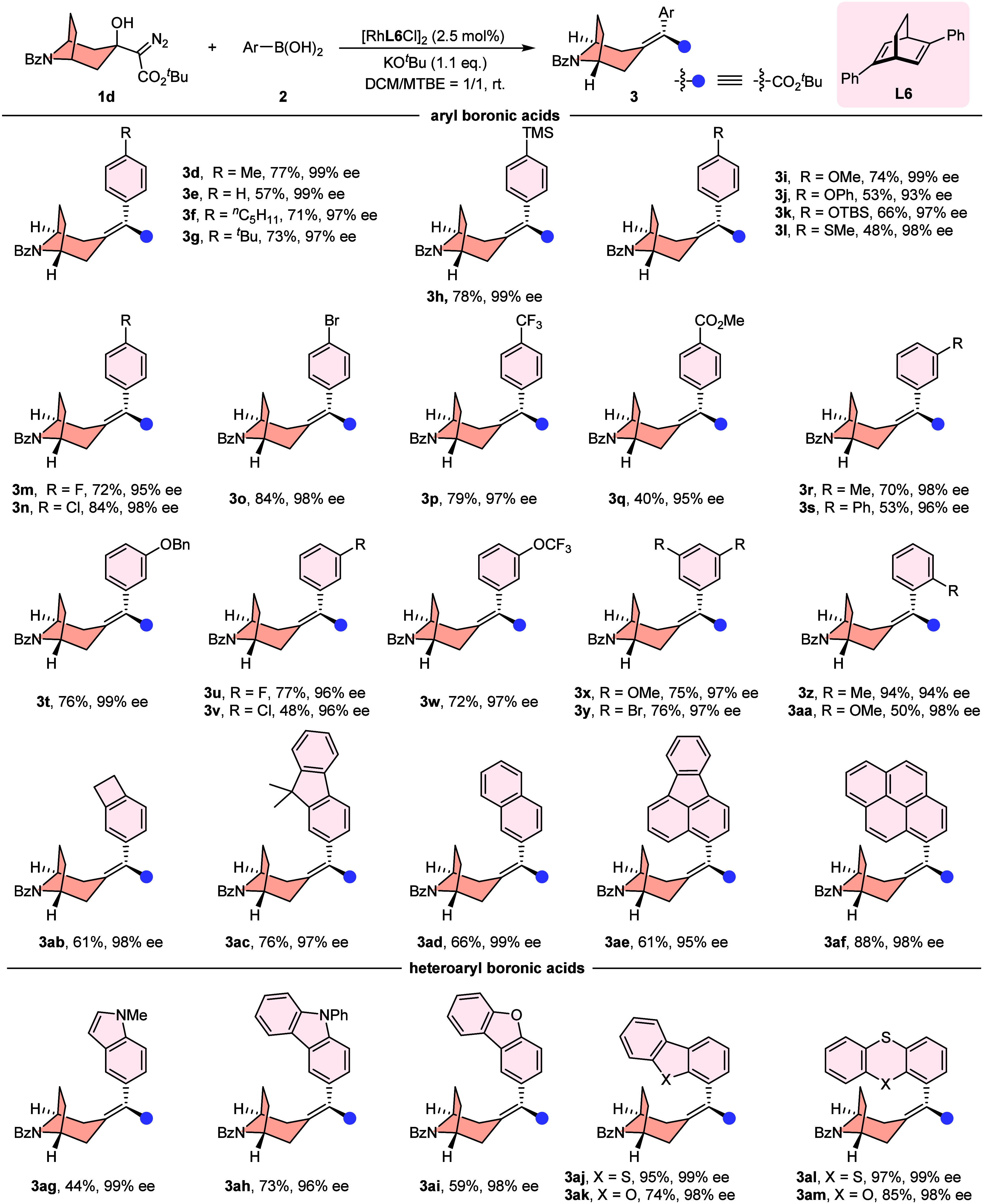
Substrate scope of arylboronic acids in the desymmetrizing carbene coupling reaction. Reaction conditions: **1d** (0.1 mmol, 1.0 equiv), **2** (2.0 equiv), [Rh­(**L6**)­Cl]_2_ (2.5 mol %), KO^*t*^Bu (1.1 equiv), DCM/MTBE = 1:1 (0.05 M) at room temperature for 12 h.

Subsequently, the substrate scope of α-diazo esters **1** was investigated under optimized reaction conditions. As listed in Figure 3, a variety of tropinone-derived α-diazocarbonyl compounds bearing different electron-withdrawing *N*-protecting groups were tolerated and, generally, moderate yields and good to excellent enantioselectivities were observed (**3an**–**3ax**). Besides, a tetraphenylethylene (**3ax**, an efficient fluorophore with aggregation-induced emission characteristics) could be embedded into the alkylidenecyclane in excellent enantioselectivity (96% ee) with moderate yield (66%) when using an *N*-Cbz-protected diazo substrate. Specifically, the 8-oxabicyclo[3.2.1]­octan-3-one-derived α-diazocarbonyl substrate could smoothly undergo the coupling reaction to yield the corresponding product **3ay** in 60% yield with 99% ee. Notably, the reaction is not limited to a bridged [3.2.1] ring system, and the [3.3.1] ring-derived α-diazocarbonyl compounds were also suitable substrates, providing the optically active products (**4a**–**4e**) in good yields. It is noteworthy that changing the *N*-protecting group from Boc to electron-donating group (Bn) had no significant influence on the enantioselectivity, albeit with a somewhat lower yield (**4f**).

**Figure 3:**
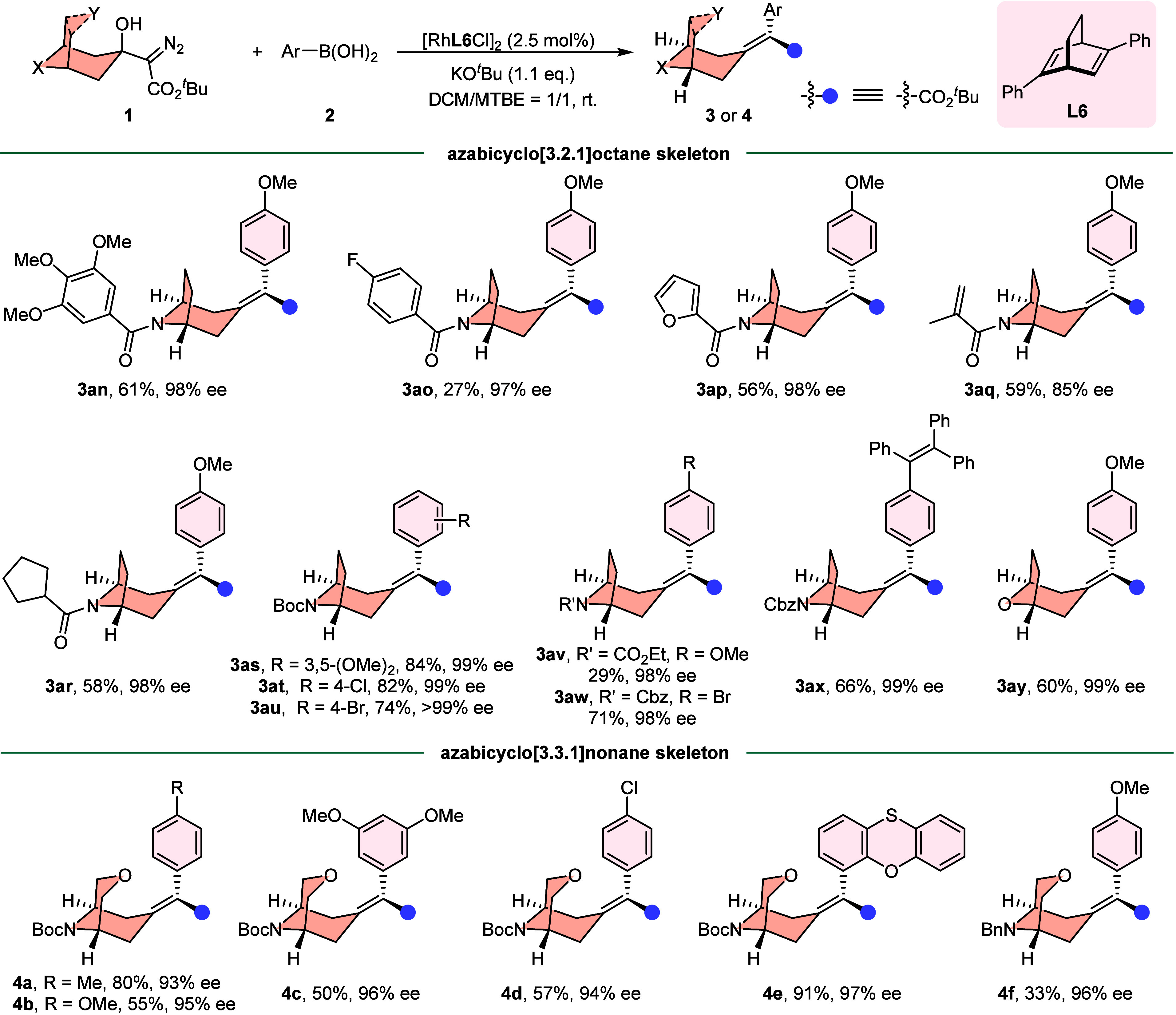
Substrate scope of β-hydroxyl α-diazocarbonyl compounds and arylboronic acids in the desymmetrizing carbene coupling reaction. Reaction conditions: **1** (0.1 mmol, 1.0 equiv), **2** (2.0 equiv), [Rh­(**L6**)­Cl]_2_ (2.5 mol %), KO^*t*^Bu (1.1 equiv), DCM/MTBE = 1:1 (0.05 M) at room temperature for 12 h.

### Applications

To assess the scalability and practical utility of this method, a gram-scale reaction of α-diazoester **1b** with 4-bromophenylboronic acid in the presence of 1.2 mol % catalyst was performed (Figure 4A). At this scale, 0.89 g of **3au** was isolated in higher yield (93%) with maintained excellent enantioselectivity (98% ee). To demonstrate the synthetic utility of the desymmetrizing carbene coupling reaction, we then performed several transformations of **3au**. The Boc-protecting group of **3au** could be successfully removed under acidic condition in the air, furnishing the secondary amine **5** in 44% yield and the appropriate hydrochloride salt **6** in 90% yield. Compound **6** was treated with Ph_2_PCl and BH_3_·THF to afford the phosphine borane complex **7** without loss of enantiopurity. Besides, the enantiopure δ-amino acid **8** was generated by simultaneously removing the Boc protecting group and hydrolyzing the ester linkage under a nitrogen atmosphere. Meanwhile, the ester unit of product **3au** could be reduced by DIBAL-H to an allyl alcohol **9**.

**Figure 4:**
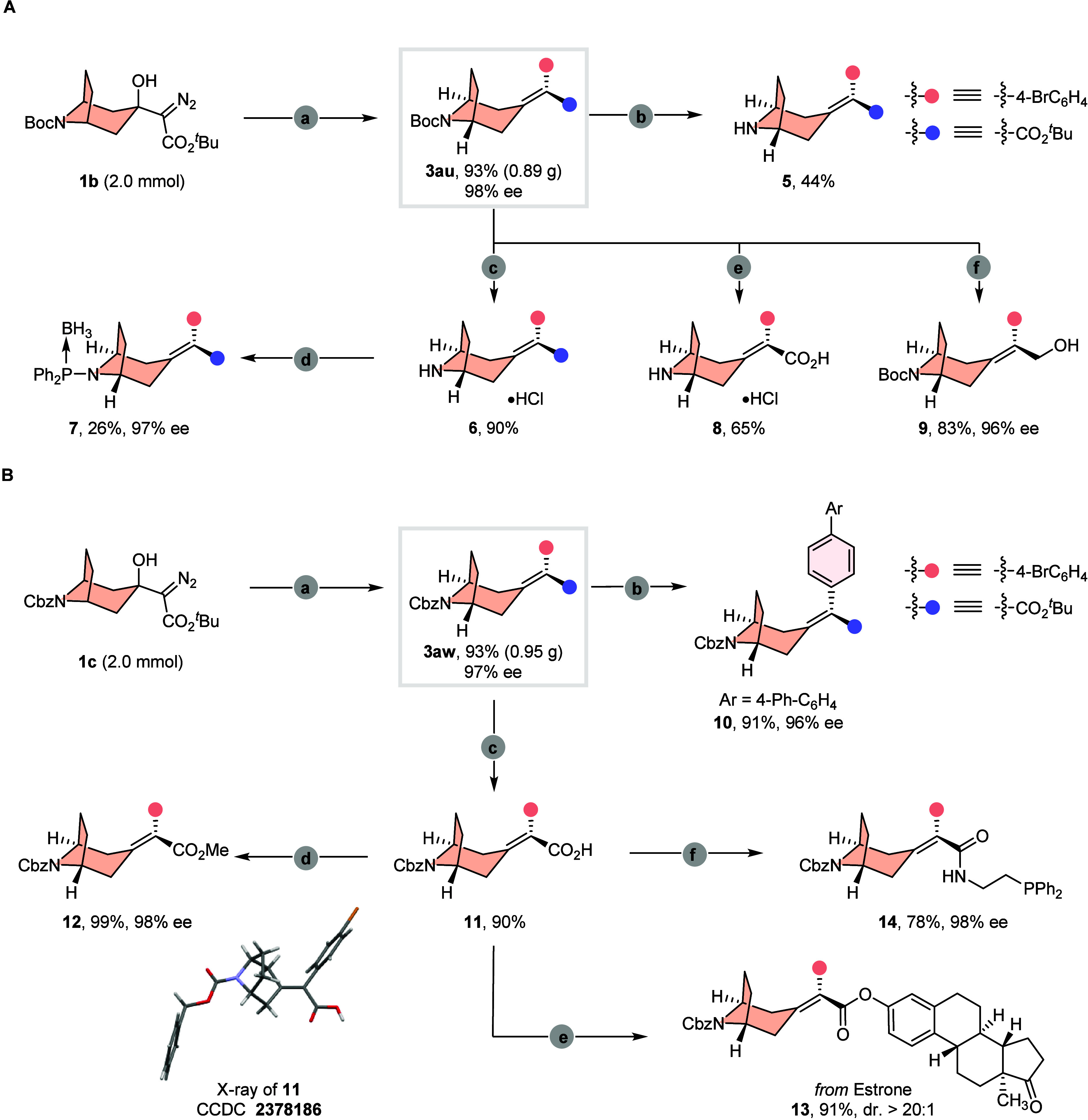
Gram-scale reactions and diverse transformations. (A) Gram-scale reaction and diverse transformations of **3au**: (a) **1b** (2.0 mmol), 4–Br-C_6_H_4_B­(OH)_2_ (2.0 equiv), [Rh­(C_2_H_4_)_2_Cl]_2_ (1.2 mol %), **L6** (3 mol %), KO^*t*^Bu (1.1 equiv), DCM/MTBE = 1/1, rt., 12 h; (b) HCl (4 M in dioxane), air, rt., 8 h, then NaOH (aq); (c) HCl (4 M in dioxane), air, rt., 8 h; (d) PPh_2_PCl (1.2 equiv), Et_3_N (6.0 equiv), THF, 0 °C to rt., 18 h, then BH_3_·THF (1.2 equiv), THF, 0 °C to rt., 1 h; (e) HCl (4 M in dioxane), N_2_, rt., 8 h; (f) DIBAL-H (2.2 equiv), DCM, 0 °C to rt., 24 h. (B) Gram-scale reaction and diverse transformations of **3aw**: (a) **1c** (2.0 mmol), 4-Br-C_6_H_4_B­(OH)_2_ (2.0 equiv), [Rh­(C_2_H_4_)_2_Cl]_2_ (1.2 mol %), **L6** (3 mol %), KO^*t*^Bu (1.1 equiv), DCM/MTBE = 1/1, rt., 12 h; (b) 4-Ph-C_6_H_4_B­(OH)_2_ (2.0 equiv), Pd­(PPh_3_)_4_ (5 mol %), Na_2_CO_3_ (3.5 equiv), toluene:EtOH:H_2_O = 5:1:2, 90 °C, 8 h; (c) CF_3_CO_2_H, DCM, rt., 3 h; (d) MeI (3.0 equiv), K_2_CO_3_ (4.0 equiv), DMF, 0 °C to rt., 8 h; (e) estrone (1.2 equiv), DMAP (20 mol %), DCC (1.3 equiv), DCM, 0 °C to rt., 8 h; (f) 2-(diphenylphosphanyl)­ethan-1-amine, BOP (1.2 equiv), Et_3_N (6.0 equiv), THF, 0 °C to rt., 8 h.

A 2.0 mmol scale coupling reaction was also performed to give product **3aw** in 93% yield (0.95 g) and 97% ee (Figure 4B). The utility of the obtained product was demonstrated through a palladium-catalyzed Suzuki coupling reaction with 4-biphenylboronic acid to give **10**. The ester unit of product **3aw** could be converted into axially chiral alkylidene tropane carboxylic acid **11** in 90% yield. In addition, the absolute configuration of this type of axial chiral skeleton was unambiguously confirmed by the X-ray crystallographic analysis of **11**. The esterification of the resulting carboxylic acid with estrone could install the complex fragment steadily into product **13** in high yield. Moreover, compound **11** could be transformed into axially chiral amide–tertiary phosphine **14** with retained enantioselectivity. In addition, stability studies of **3u** and **4f** revealed no racemization under thermal conditions (120 °C, 24 h), confirming the exceptional stability of the axially chiral architecture (see the Supporting Information for details).

### Mechanistic Discussion

On the basis of relevant studies,^81,82^ a probable mechanism is proposed for the Rh­(I)-catalyzed coupling reaction of α-diazocarbonyl compounds with arylboronic acids. The bis­(rhodium/diene) complex undergoes initial ligand dissociation to yield active monorhodium catalyst species **I**, which facilitates transmetalation with arylboronic acid **2** to generate arylrhodium intermediate **II** with the aid of KO^*t*^Bu. The intermediate **II** then reacts with diazo substrate **1** to give rhodium–carbene species **III** with the extrusion of a molecule of N_2_ gas. The subsequent asymmetric migratory insertion of **III** generates a quaternary carbon center with a C–Rh bond, to give chiral intermediate **IV**. Finally, β-OH elimination furnishes the chirality-transfer process from central-to-axial chirality to give the final axially chiral product **3** along with the regeneration of the active rhodium species to complete the catalytic cycle (Figure 5a). An empirical stereocontrol model is proposed to rationalize the observed stereoselectivity (Figure 5b). Species **III** is considered to serve as a critical role in the control of the enantioselectivity of products; the aryl group of the arylrhodium intermediate and the phenyl part of the chiral diene ligand are likely to form a π–π stacking interaction.

**Figure 5:**
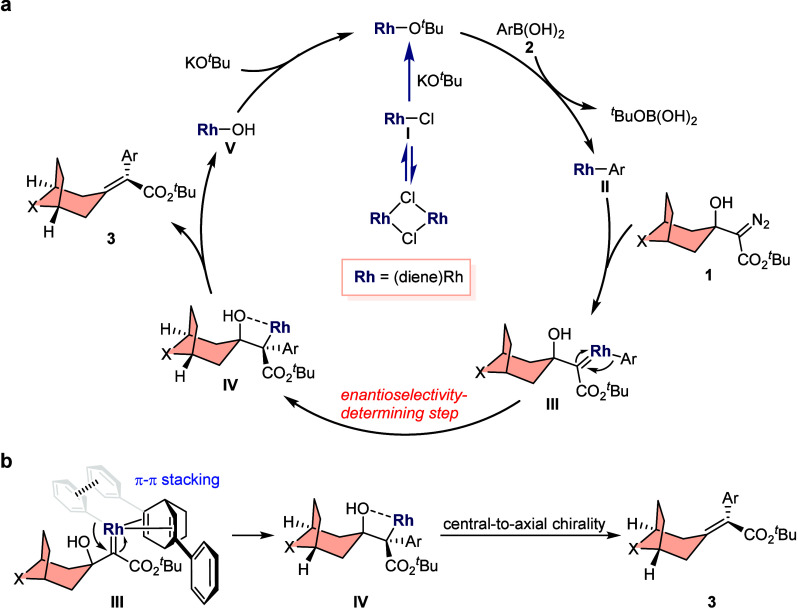
Mechanistic discussion: (a) proposed mechanism; (b) plausible model to explain the absolute stereochemistry of products.

## Conclusions

In summary, a novel axially chiral alkylidene *N*-bridged cyclic skeleton was successfully synthesized for the first time through a Rh­(I)/diene-catalyzed remotely controlled desymmetrization strategy. The protocol portrays readily accessible reagents and simple conditions and provides a straightforward access to a diverse range of three-dimensional nonatropisomeric axially chiral *N*-bridged [3.2.1] and [3.3.1] ring systems with high yields, remarkable chemoselectivity, and excellent enantiocontrol, which are difficult to access through other methods. Meanwhile, an axially chiral oxabicyclo[3.2.1]­octane skeleton could also be efficiently prepared with high yield and excellent enantioselectivity. Additionally, the Rh­(I)/diene-catalyzed asymmetric carbene coupling reaction shows excellent compatibility with versatile functional groups, which is proved by broad substrate scope. The applications of axially chiral products are demonstrated by a series of transformations to construct a variety of tetrasubstituted axially chiral alkylidene skeletons without erosion of stereoselectivities, thus emphasizing the practicality of this chemistry. The *C*_2_-symmetric chiral diene ligand was crucial in ensuring high efficiency and enantiocontrol, which may proceed through a π–π stacking interaction between the aryl group of the arylrhodium intermediate and the phenyl unit of the diene ligand. Further studies on the application of such an enantioselective carbene coupling reaction for the synthesis of other axially chiral molecules are currently underway.

## Supplementary Material











## Notes

General information, optimization of reaction conditions, general procedure for the preparation of substrates, general procedure for the cross-coupling reaction of α-diazocarbonyl compounds and aryl boronic acids, gram-scale synthesis of products **3au** and **3aw**, procedure for the synthetic applications, X-ray diffraction analysis, references, and NMR spectra (PDF)
Crystallographic information (CIF)
CheckCIF/PLATON report (PDF)
Crystallographic information (CIF)
CheckCIF/PLATON report (PDF)

## Notes

Deposition Numbers 2407660 and 2378186 contain the supplementary crystallographic data for this paper. These data can be obtained free of charge via the joint Cambridge Crystallographic Data Centre (CCDC) and Fachinformationszentrum Karlsruhe Access Structures service.
