# Supplementary material for: Rh(I)-Catalyzed Modular Synthesis of Axially Chiral Alkylidene Azacycloalkanes
Source: ACS Cent Sci. 2025 May 11;11(6):899–909. doi: 10.1021/acscentsci.5c00232 (PMC12203264; doi:10.1021/acscentsci.5c00232)
Supplement: Supplementary file 1 [file oc5c00232_si_001.pdf]

Supporting Information

Rh(I)-Catalyzed Modular Synthesis of Axially Chiral Alkylidene Azacycloalkanes

Yang Chen,[1,4] Jiayan Chen,[1,4] and Shifa Zhu*,[1,2,3]

[1]Key Laboratory of Functional Molecular Engineering of Guangdong Province, School of Chemistry and Chemical Engineering, South China University of Technology, Guangzhou 510640, China; *Correspondence: zhusf@scut.edu.cn

[2]State Key Laboratory of Bio-based Fiber Materials, School of Chemistry and Chemical Engineering, Zhejiang Sci-Tech University, Hangzhou 310018, China

[3]State Key Laboratory of Elemento-Organic Chemistry, Nankai University, Tianjin 300071, China

[4]These authors contributed equally: Yang Chen, Jiayan Chen

Contents

1. General information S3

2. Optimization of reaction conditions S4

3. General procedure for the preparation of substrates.. S5

4. General procedure for the cross-coupling reaction of α-diazocarbonyl compounds and aryl boronic acids.. S14

5. The gram-scale synthesis of products **3au** and **3aw**.. S71

6. The procedure for the synthetic applications... S72

8. The X-ray diffraction analysis.. S86

9. References S88

10. The NMR spectra S89

1. General information

All reactions were conducted under dry N_2 atmosphere in Schlenk tube. Catalysts were commercially available. Commercially obtained reagents were used without purification. Reaction starting materials were prepared as depicted in the literature. ^{1}H, ^{13}C NMR spectra were recorded on the Bruker AVANCE 400 (400 MHz for ^{1}H; 100 MHz for ^{13}C; 376 MHz for ^{19}F) and Bruker AVANCE 500 (500 MHz for ^{1}H; 125 MHz for ^{13}C; 471 MHz for ^{19}F), ^{1}H NMR and ^{13}C NMR chemical shifts were determined relative to internal standard TMS at δ 0.0. Chemical shifts (δ) are reported in ppm, and coupling constants (J) are in Hertz (Hz). The following abbreviations were used to explain the multiplicities: s = singlet, d =doublet, t = triplet, q = quartet, m = multiplet, br = broad. Mass spectra (HRMS) were obtained using Agilent UHD Accurate Mass Q-TOF LC/MS (ESI) mass spectrometer. Melting points were determined using a hot stage apparatus. Chiral HPLC analyses were performed on SHIMADZU LC-15C system. Specific rotation [a] was determined using a polarimeter Zhuo Guang GP30 with a 10 mL cell, long 10 cm, $[\alpha]_D^T$ values, reported in ° mL g^{-1} dm^{-1}, are calculated on the average value of 3 consecutive readings. All other reagents and solvents were used as received from commercial sources (Energy Chemical, Bidepharm, Innochem, etc.) unless specified otherwise, or prepared as described in the literatures. Rhodium(I) catalysts and chiral ligands were purchased from Strem Chemicals, TCI Chemicals or Bidepharm.

2. Optimization of reaction conditions

Table S1 Optimization of the reaction conditions[a]

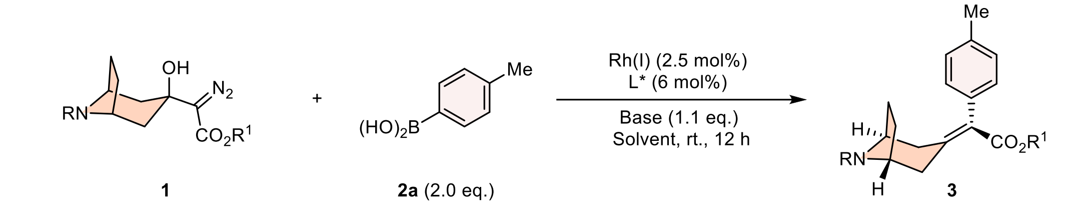

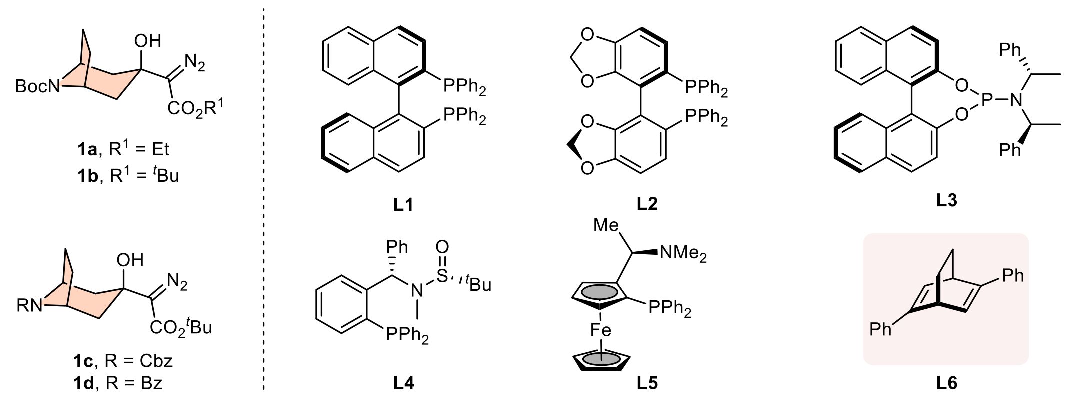

Entry	1	Rh catalyst (mol %)	Base	Solvent	3	Yield[b] (%)	Ee[c] (%)
1	**1a**	$[Rh(NBD)Cl]_2$	KO*t*Bu	MTBE	**3a**	56	/
2	**1a**	[Rh] (2.5) + **L1** (6)	KO*t*Bu	MTBE	**3a**	ND[d]	/
3	**1a**	[Rh] (2.5) + **L2** (6)	KO*t*Bu	MTBE	**3a**	ND[d]	/
4	**1a**	[Rh] (2.5) + **L3** (6)	KO*t*Bu	MTBE	**3a**	ND[d]	/
5	**1a**	[Rh] (2.5) + **L4** (6)	KO*t*Bu	MTBE	**3a**	ND[d]	/
6	**1a**	[Rh] (2.5) + **L5** (6)	KO*t*Bu	MTBE	**3a**	ND[d]	/
7	**1a**	[Rh] (2.5) + **L6** (6)	KO*t*Bu	MTBE	**3a**	55	53
8	**1b**	[Rh] (2.5) + **L6** (6)	KO*t*Bu	MTBE/DCM = 1:1	**3b**	39	97
9[e]	**1b**	[Rh] (2.5) + **L6** (6)	KO*t*Bu	MTBE/DCM = 1:1	**3b**	13	-96
10	**1c**	[Rh] (2.5) + **L6** (6)	KO*t*Bu	MTBE/DCM = 1:1	**3c**	77	97
11	**1d**	[Rh] (2.5) + **L6** (6)	KO*t*Bu	MTBE/DCM = 1:1	**3d**	76	99
12	**1d**	[Rh(**L6**)Cl]$_2$ (2.5)	KO*t*Bu	MTBE/DCM = 1:1	**3d**	77	99
13	**1d**	[Rh(**L6**)Cl]$_2$ (2.5)	KO*t*Bu	MTBE/THF = 1:1	**3d**	44	93
14	**1d**	[Rh(**L6**)Cl]$_2$ (2.5)	KO*t*Bu	MTBE/Toluene = 1:1	**3d**	62	93
15	**1d**	[Rh(**L6**)Cl]$_2$ (2.5)	Et_3N	MTBE/DCM = 1:1	**3d**	ND[d]	/
16	**1d**	[Rh(**L6**)Cl]$_2$ (2.5)	KOH	MTBE/DCM = 1:1	**3d**	ND[d]	/
17	**1d**	[Rh(**L6**)Cl]$_2$ (2.5)	K_3PO_4	MTBE/DCM = 1:1	**3d**	ND[d]	/
18	**1d**	none	KO*t*Bu	MTBE/DCM = 1:1	**3d**	ND[d]	/

[a]Unless otherwise noted, all reactions were performed with **1** (0.1 mmol), **2a** (0.2 mmol), $[Rh(C_2H_4)_2Cl]_2$ (2.5 mol %), **L*** (6 mol%) and KO*t*Bu (0.11 mmol) in 2 mL solvent at room temperature for 12 h. [b]Isolated yield. [c]Determined by chiral HPLC. [d]Not detected. [e]The opposite configurational diene ligand was used. *t*Bu, *tert*-butyl; Boc, *tert*-butoxycarbonyl; Cbz, carboxybenzyl; Bz, benzoxyl; NBD, norbornadiene.

3. General procedure for the preparation of substrates

The related substrates are listed below.

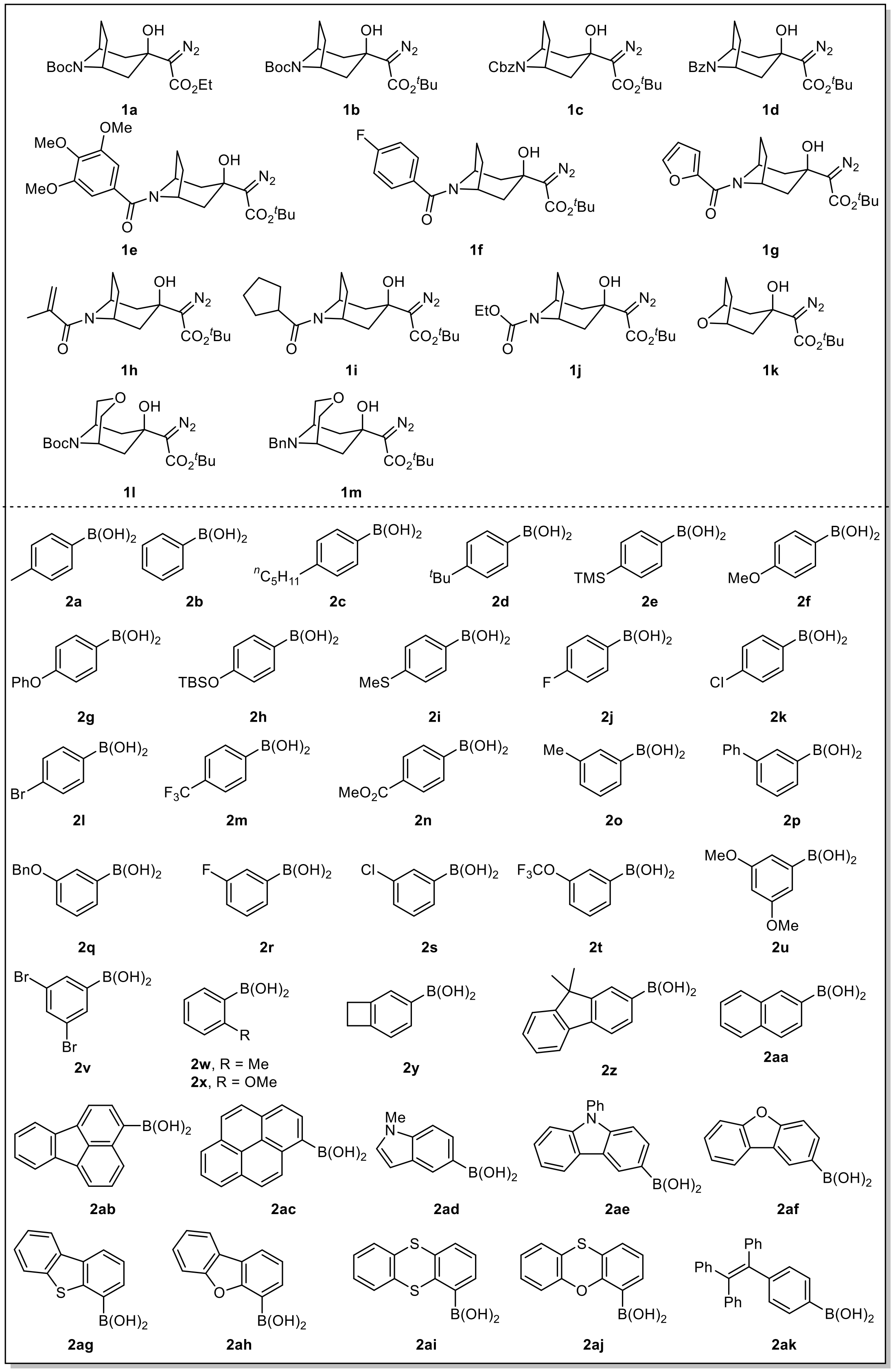

General procedure for the preparation of **1**:

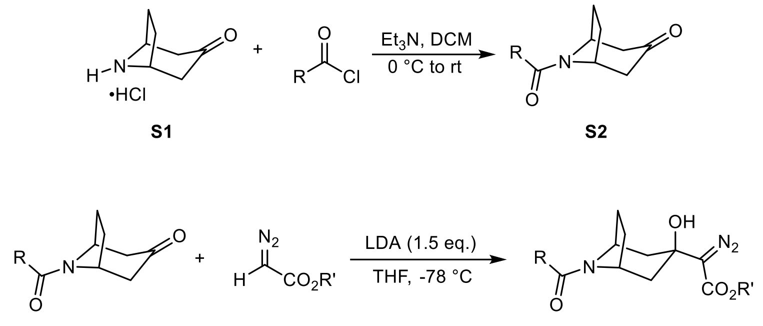

Synthesis of **S2**.[1] The nortropinone hydrochloride (**S1**, 20.0 mmol) in DCM (100 mL) was added Et_3N (35 mL), acyl chloride (1.0 eq.) was added dropwise to the mixture at 0 °C. The resulting solution was warmed to room temperature and stirred for 5 h. The mixture was concentrated under reduced pressure. The resulting crude product was purified by flash column chromatography to give the product **S2** (eluent: PE/EtOAc = 6:4).

Synthesis of **1**.[2] Add lithium diisopropylamide in THF (2 M, 1.5 eq.) dropwise to a solution of **S2** (2.0 mmol) and diazoacetate (1.6 eq.) in anhydrous THF (10 mL) at -78 °C. The reaction was stirred at -78 °C for 7 h and then quenched with saturated aqueous solution of NH_4Cl and extracted with EtOAc. The combined organic phases were washed with brine, dried over Na_2SO_4, and concentrated under reduced pressure. The resulting crude product was purified by flash column chromatography to give the product **1** (eluent: PE/EtOAc = 3:1).

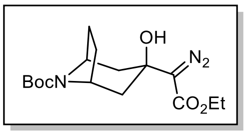

***tert*-butyl-3-(1-diazo-2-ethoxy-2-oxoethyl)-3-hydroxy-8-azabicyclo[3.2.1]octane-8-carboxylate (1a)**

Yield: 63%, yellow solid, R_f = 0.42 (PE/EtOAc = 10:1), M.P. = 102.8 - 103.3 °C.

^{1}H NMR (400 MHz, $CDCl_3$) δ 4.28 (s, 1H), 4.24 – 4.17 (m, 3H), 4.01 (s, 1H), 2.20 (q, *J* = 5.8 Hz, 2H), 2.05 (d, *J* = 23.8 Hz, 4H), 1.89 (dd, *J* = 8.1, 4.5 Hz, 2H), 1.45 (s, 9H), 1.26 (t, *J* = 7.1 Hz, 3H).

^{13}C NMR (100 MHz, $CDCl_3$) δ 167.1, 153.4, 80.3, 79.5, 69.6, 64.3, 60.9, 53.3, 40.7, 28.5, 28.4, 14.4.

HRMS (ESI) ([M + Na]$^+$) Calcd. for $[C_{16}H_{25}NaN_3O_5]^+$: 362.1686, Found. 362.1691.

HRMS spectrum of **1a**

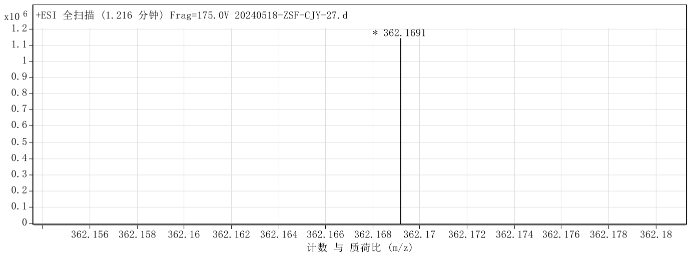

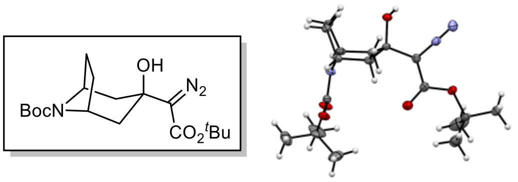

***tert*-butyl-3-(2-(tert-butoxy)-1-diazo-2-oxoethyl)-3-hydroxy-8-azabicyclo[3.2.1]octane-8-carboxylate (1b)**

Yield: 59%, yellow solid, R_f = 0.43 (PE/EtOAc = 10:1), M.P. = 123.3 - 124.5 °C.

^{1}H NMR (500 MHz, CDCl$_3$) δ 4.21 (d, *J* = 45.6 Hz, 2H), 4.11 (s, 1H), 2.24 – 2.12 (m, 2H), 2.05 (s, 3H), 1.94 (dd, *J* = 9.6, 5.3 Hz, 1H), 1.90 – 1.83 (m, 2H), 1.55 – 1.36 (m, 18H).

^{13}C NMR (125 MHz, CDCl$_3$) δ 166.8, 153.4, 82.3, 79.4, 69.7, 69.7, 53.3, 52.5, 41.6, 40.9, 28.5, 28.4, 28.3, 28.1, 27.9, 27.4.

HRMS (ESI) ([M + Na]$^+$) Calcd. for [$C_{18}H_{29}NaN_3O_5$]$^+$: 390.1999, Found. 390.2007.

HRMS spectrum of **1b**

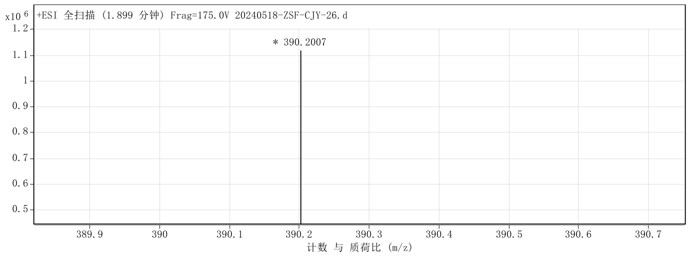

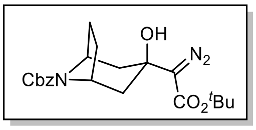

benzyl-3-(2-(tert-butoxy)-1-diazo-2-oxoethyl)-3-hydroxy-8-azabicyclo[3.2.1]octane-8-carboxylate (1c)

Yield: 65%, yellow solid, R_f = 0.41 (PE/EtOAc = 5:1), M.P. = 119.5 - 120.3 °C.

^{1}H NMR (400 MHz, CDCl$_3$) δ 7.40 – 7.34 (m, 4H), 7.34 – 7.29 (m, 1H), 5.15 (d, *J* = 4.7 Hz, 2H), 4.36 (d, *J* = 15.3 Hz, 2H), 4.11 (s, 1H), 2.24 (t, *J* = 6.8 Hz, 2H), 2.11 (s, 3H), 2.04 (t, *J* = 10.8 Hz, 1H), 1.94 – 1.89 (m, 2H), 1.47 (s, 9H).

^{13}C NMR (100 MHz, CDCl$_3$) δ 166.6, 153.4, 136.8, 128.5, 128.0, 127.9, 82.3, 69.6, 66.8, 53.1, 41.9, 41.0, 28.4, 28.2, 27.4.

HRMS (ESI) ([M + Na]$^+$) Calcd. for $[C_{21}H_{27}NaN_3O_5]^+$: 424.1843, Found. 424.1846.

HRMS spectrum of **1c**

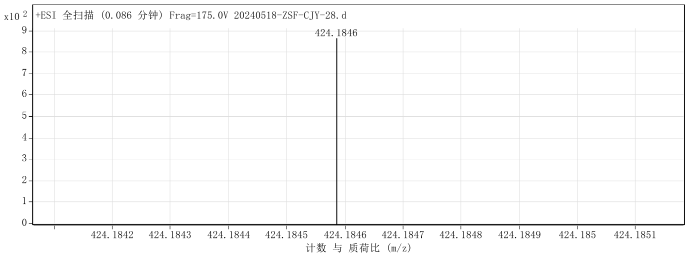

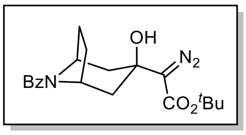

***tert*-butyl-2-(8-benzoyl-3-hydroxy-8-azabicyclo[3.2.1]octan-3-yl)-2-diazoacetate (1d)**

Yield: 68%, yellow solid, R_f = 0.43 (PE/EtOAc = 3:1), M.P. = 162.9 - 164.2 °C.

^{1}H NMR (400 MHz, CDCl$_3$) δ 7.50 – 7.44 (m, 2H), 7.44 – 7.35 (m, 3H), 4.98 – 4.77 (m, 1H), 4.21 (s, 1H), 4.15 – 4.05 (m, 1H), 2.44 – 2.21 (m, 3H), 2.14 (dd, *J* = 25.1, 12.5 Hz, 2H), 2.08 – 1.87 (m, 3H), 1.47 (s, 9H).

^{13}C NMR (100 MHz, CDCl$_3$) δ 168.0, 166.6, 136.0, 130.1, 128.5, 127.1, 82.4, 69.6, 56.2, 51.2, 43.6, 40.9, 28.4, 26.9.

HRMS (ESI) ([M + Na]$^+$) Calcd. for $[C_{20}H_{25}NaN_3O_4]^+$: 394.1737, Found. 394.1739.

HRMS spectrum of **1d**

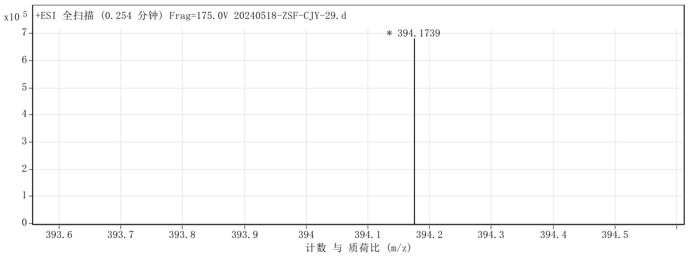

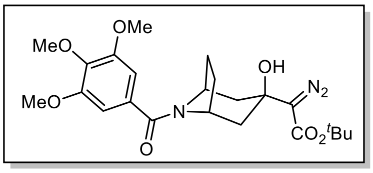

***tert*-butyl-2-diazo-2-(3-hydroxy-8-(3,4,5-trimethoxybenzoyl)-8-azabicyclo[3.2.1]octan-3-yl)acetate (1e)**

Yield: 58%, yellow solid, R_f = 0.35 (PE/EtOAc = 1:1), M.P. = 153.9 - 155.3 °C.

^{1}H NMR (500 MHz, $CDCl_3$) δ 6.67 (s, 2H), 4.74 (s, 1H), 4.20 (s, 1H), 4.12 – 4.00 (m, 1H), 3.80 (d, *J* = 5.8 Hz, 9H), 2.23 (dt, *J* = 35.4, 12.1 Hz, 3H), 2.06 (q, *J* = 20.8, 17.7 Hz, 3H), 1.94 – 1.84 (m, 2H), 1.40 (s, 9H).

^{13}C NMR (125 MHz, $CDCl_3$) δ 171.0, 167.8, 166.0, 153.2, 153.2, 139.3, 131.6, 104.3, 103.5, 82.0, 69.1, 65.1, 60.8, 60.4, 56.2, 56.1, 51.3, 43.1, 41.2, 28.3, 28.0, 26.8, 21.0, 14.2.

HRMS (ESI) ([M + Na]$^+$) Calcd. for $[C_{23}H_{31}NaN_3O_7]^+$: 484.2054, Found. 484.2054.

HRMS spectrum of **1e**

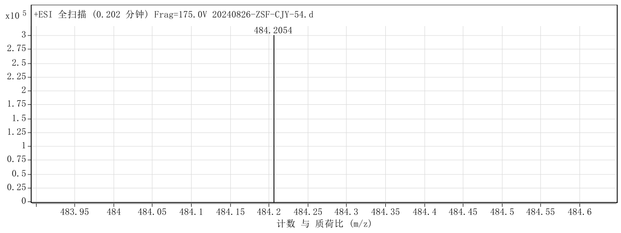

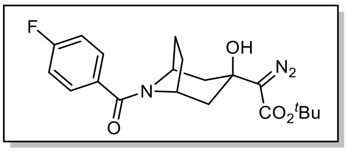

***tert*-butyl-2-diazo-2-(8-(4-fluorobenzoyl)-3-hydroxy-8-azabicyclo[3.2.1]octan-3-yl)acetate (1f)**

Yield: 69%, yellow solid, R_f = 0.37 (PE/EtOAc = 3:1), M.P. = 154.9 - 155.2 °C.

^{1}H NMR (500 MHz, $CDCl_3$) δ 7.46 (dd, *J* = 8.5, 5.5 Hz, 2H), 7.10 – 7.01 (m, 2H), 4.80 (s, 1H), 4.16 (s, 1H), 4.12 – 4.06 (m, 1H), 2.27 (dt, *J* = 20.3, 10.5 Hz, 3H), 2.13 (d, *J* = 14.3 Hz, 2H), 2.00 – 1.84 (m, 3H), 1.44 (s, 9H).

^{13}C NMR (125 MHz, $CDCl_3$) δ 167.1, 166.4, 164.6, 162.7, 132.1, 132.0, 129.6, 129.5, 129.4, 129.4, 115.9, 115.7, 115.6, 115.4, 82.3, 69.5, 56.4, 51.4, 43.5, 40.8, 28.3, 28.1, 28.0, 26.8.

^{19}F NMR (376 MHz, $CDCl_3$) δ -109.8.

HRMS (ESI) ([M + Na]$^+$) Calcd. for $[C_{20}H_{24}NaFN_3O_4]^+$: 412.1643, Found. 412.1643.

HRMS spectrum of **1f**

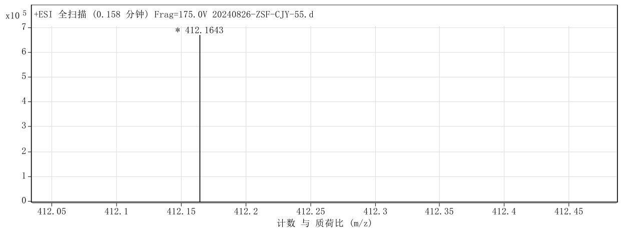

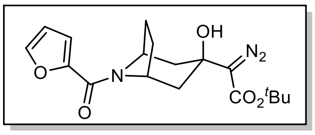

***tert*-butyl-2-diazo-2-(8-(furan-2-carbonyl)-3-hydroxy-8-azabicyclo[3.2.1]octan-3-yl)acetate (1g)**

Yield: 39%, yellow oil, R_f = 0.49 (PE/EtOAc = 3:1).

^{1}H NMR (400 MHz, CDCl$_3$) δ 7.46 (dd, *J* = 1.8, 0.9 Hz, 1H), 7.07 (dd, *J* = 3.4, 1.0 Hz, 1H), 6.46 (dd, *J* = 3.5, 1.8 Hz, 1H), 4.93 (d, *J* = 49.9 Hz, 2H), 4.28 (d, *J* = 2.1 Hz, 1H), 2.45 – 2.22 (m, 3H), 2.16 (d, *J* = 3.4 Hz, 3H), 1.99 (d, *J* = 22.9 Hz, 1H), 1.88 (s, 1H), 1.42 (s, 9H).

^{13}C NMR (100 MHz, CDCl$_3$) δ 166.4, 155.5, 148.6, 143.8, 116.5, 111.5, 82.2, 77.4, 69.5, 54.5, 52.0, 43.8, 41.3, 28.7, 28.3, 26.0.

HRMS (ESI) ([M + Na]$^+$) Calcd. for $[C_{18}H_{23}NaN_3O_5]^+$: 384.1530, Found. 384.1534.

HRMS spectrum of **1g**

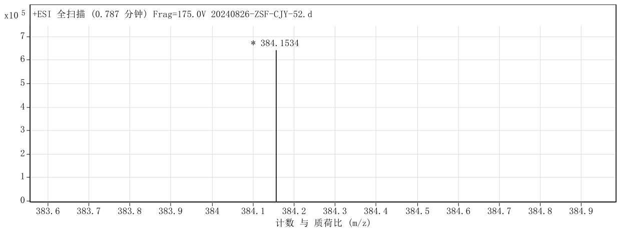

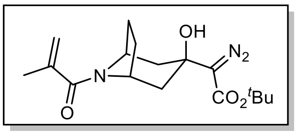

***tert*-butyl-2-diazo-2-(3-hydroxy-8-methacryloyl-8-azabicyclo[3.2.1]octan-3-yl)acetate (1h)**

Yield: 31%, yellow solid, R_f = 0.28 (PE/EtOAc = 3:1), M.P. = 147.5 - 148.8 °C.

^{1}H NMR (500 MHz, CDCl$_3$) δ 5.14 (d, *J* = 2.0 Hz, 1H), 5.06 (d, *J* = 2.0 Hz, 1H), 4.68 (s, 1H), 4.34 – 4.18 (m, 2H), 2.25 (dd, *J* = 7.2, 2.0 Hz, 2H), 2.17 (d, *J* = 13.8 Hz, 1H), 2.09 (s, 2H), 2.00 – 1.93 (m, 1H), 1.95 – 1.88 (m, 3H), 1.87 – 1.79 (m, 2H), 1.55 – 1.27 (m, 9H).

^{13}C NMR (125 MHz, CDCl$_3$) δ 168.5, 166.4, 140.7, 115.6, 82.2, 69.5, 64.9, 55.6, 50.5, 43.7, 41.1, 28.3, 28.2, 26.7, 20.4.

HRMS (ESI) ([M + Na]$^+$) Calcd. for [$C_{17}H_{25}NaN_3O_4$]$^+$: 358.1737, Found. 358.1737.

HRMS spectrum of **1h**

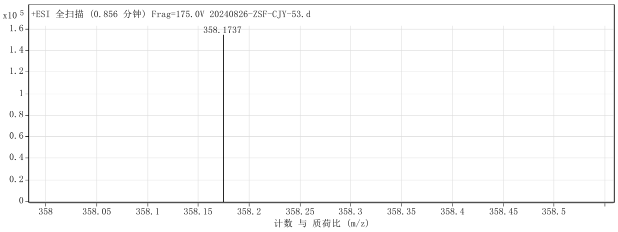

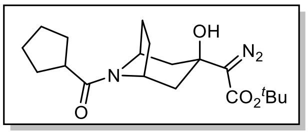

***tert*-butyl-2-(8-(cyclopentanecarbonyl)-3-hydroxy-8-azabicyclo[3.2.1]octan-3-yl)-2-diazoacetate (1i)**

Yield: 48%, yellow solid, R_f = 0.43 (PE/EtOAc = 3:1), M.P. = 146.7 - 148.0 °C.

^{1}H NMR (500 MHz, CDCl$_3$) δ 4.67 (dt, *J* = 7.0, 3.1 Hz, 1H), 4.26 (s, 1H), 4.25 (dd, *J* = 8.3, 4.6 Hz, 1H), 2.84 – 2.69 (m, 1H), 2.28 (ddd, *J* = 11.4, 9.4, 3.7 Hz, 1H), 2.24 – 2.17 (m, 2H), 2.03 (dt, *J* = 5.5, 2.8 Hz, 2H), 1.96 (dd, *J* = 14.1, 3.9 Hz, 1H), 1.91 – 1.66 (m, 8H), 1.51 (dq, *J* = 8.3, 2.9, 2.4 Hz, 2H), 1.41 (s, 9H).

^{13}C NMR (125 MHz, CDCl$_3$) δ 171.9, 166.4, 82.2, 69.5, 53.9, 50.7, 43.7, 42.0, 41.1, 30.6, 30.0, 28.6, 28.3, 26.7, 26.1, 26.1.

HRMS (ESI) ([M + Na]$^+$) Calcd. for [$C_{19}H_{29}NaN_3O_4$]$^+$: 386.2050, Found. 386.2050.

HRMS spectrum of **1i**

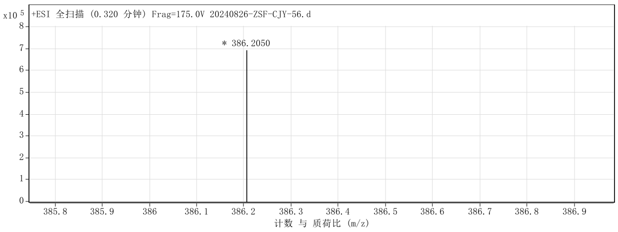

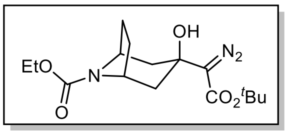

***tert*-butyl-2-diazo-2-(3-hydroxy-8-propionyl-8-azabicyclo[3.2.1]octan-3-yl)acetate (1j)**

Yield: 51%, yellow solid, R_f = 0.44 (PE/EtOAc = 3:1), M.P. = 98.8 - 100.1 °C.

^{1}H NMR (500 MHz, DMSO-*d*$_6$) δ 5.63 (s, 1H), 4.12 (s, 2H), 4.10 – 3.97 (m, 2H), 2.27 (t, *J* = 13.0 Hz, 2H), 2.12 (s, 2H), 1.78 (d, 2H), 1.72 (d, *J* = 14.0 Hz, 2H), 1.39 (s, 9H), 1.18 (t, *J* = 7.1 Hz, 3H).

^{13}C NMR (125 MHz, DMSO-*d*$_6$) δ 164.5, 153.3, 81.2, 67.7, 60.5, 53.1, 28.4, 27.4, 15.1.

HRMS (ESI) ([M + Na]$^+$) Calcd. for [$C_{16}H_{25}NaN_3O_5$]$^+$: 362.1686, Found. 362.1686.

HRMS spectrum of **1j**

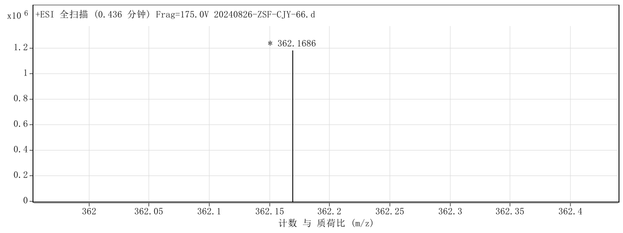

Procedure for formation of **1k**:[2]

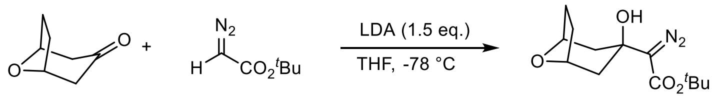

S3 **1k**

Add lithium diisopropylamide in THF (2 M, 1.5 eq.) dropwise to a solution of **S3** (1.0 mmol) and *tert*-butyl diazoacetate (1.6 eq.) in anhydrous THF (3 mL) at -78 °C. The reaction was stirred at -78 ℃ for 7 h and then quenched with saturated aqueous solution of NH_4Cl and extracted with EtOAc. The combined organic phases were washed with brine, dried over Na_2SO_4, and concentrated under reduced pressure. The resulting crude product was purified by flash column chromatography to give the product **1k** (eluent: PE/EtOAc = 5:1).

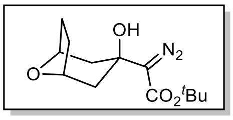

***tert*-butyl-2-diazo-2-(3-hydroxy-8-oxabicyclo[3.2.1]octan-3-yl)acetate (1k)**

Yield: 48%, yellow solid, R_f = 0.51 (PE/EtOAc = 5:1), M.P. = 105.4 - 106.7 ℃.

^{1}H NMR (500 MHz, $CDCl_3$) δ 4.36 (dd, *J* = 5.5, 2.9 Hz, 2H), 4.01 (s, 1H), 2.36 – 2.16 (m, 2H), 1.97 (d, *J* = 3.1 Hz, 4H), 1.90 – 1.76 (m, 2H), 1.61 – 1.29 (m, 9H).

^{13}C NMR (125 MHz, $CDCl_3$) δ 166.7, 82.2, 82.2, 82.1, 73.8, 68.8, 68.7, 68.7, 41.8, 28.3.

HRMS (ESI) ([M + Na]$^+$) Calcd. for [$C_{13}H_{20}NaN_2O_4$]$^+$: 291.1315, Found. 291.1323.

HRMS spectrum of **1k**

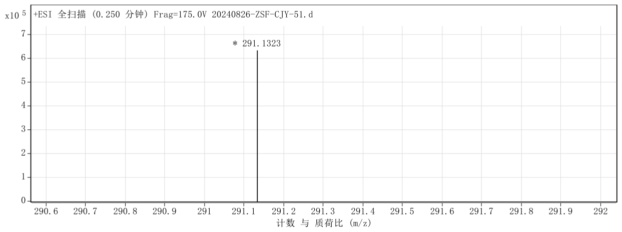

Procedure for formation of **1l** and **1m**:[2]

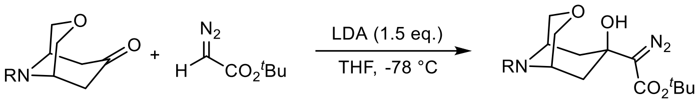

Add lithium diisopropylamide in THF (2 M, 1.5 eq.) dropwise to a solution of **S4** (2.0 mmol) and *tert*-butyl diazoacetate (1.6 eq.) in anhydrous THF (10 mL) at -78 °C. The reaction was stirred at -78 °C for 7 h and then quenched with saturated aqueous solution of NH_4Cl and extracted with EtOAc. The combined organic phases were washed with brine, dried over Na_2SO_4, and concentrated under reduced pressure. The resulting crude product was purified by flash column chromatography to give the product **1l** or **1m** (eluent: PE/EtOAc = 10:1).

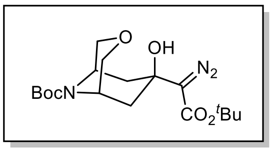

***tert*-butyl-7-(2-(tert-butoxy)-1-diazo-2-oxoethyl)-7-hydroxy-3-oxa-9-azabicyclo[3.3.1]nonane-9-carboxylate (1l)**

Yield: 58%, yellow solid, R_f = 0.49 (PE/EtOAc = 10:1), M.P. = 105.4 - 106.9 °C.

^{1}H NMR (400 MHz, CDCl$_3$) δ 6.61 (s, 1H), 4.21 (d, *J* = 4.2 Hz, 1H), 4.08 (d, *J* = 4.0 Hz, 1H), 3.88 (t, *J* = 11.5 Hz, 2H), 3.82 – 3.71 (m, 2H), 2.70 (dt, *J* = 14.7, 5.0 Hz, 2H), 1.90 – 1.74 (m, 2H), 1.49 (s, 9H), 1.46 (s, 9H).

^{13}C NMR (100 MHz, CDCl$_3$) δ 164.7, 153.6, 81.0, 80.5, 69.8, 69.6, 65.4, 48.3, 46.6, 39.0, 38.7, 28.4, 28.4.

HRMS (ESI) ($[M + Na]^+$) Calcd. for $[C_{18}H_{29}NaN_3O_6]^+$: 406.1948, Found. 406.1952.

HRMS spectrum of **1l**

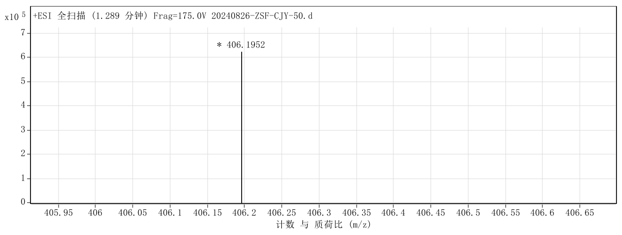

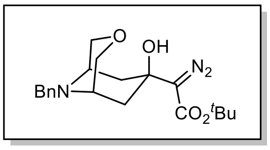

***tert*-butyl-2-(9-benzyl-7-hydroxy-3-oxa-9-azabicyclo[3.3.1]nonan-7-yl)-2-diazoacetate (1m)**

Yield: 40%, yellow solid, R_f = 0.52 (PE/EtOAc = 10:1), M.P. = 116.1 - 117.5 °C.

^{1}H NMR (500 MHz, $CDCl_3$) δ 7.41 (d, *J* = 7.1 Hz, 2H), 7.30 (t, *J* = 7.5 Hz, 2H), 7.24 (d, *J* = 6.3 Hz, 1H), 6.91 (s, 1H), 3.96 (s, 2H), 3.91 (d, *J* = 11.2 Hz, 2H), 3.80 (d, *J* = 11.0 Hz, 2H), 2.84 (dd, *J* = 14.7, 5.7 Hz, 2H), 2.77 (d, *J* = 5.6 Hz, 2H), 1.53 (s, 9H), 1.51 (s, 1H).

^{13}C NMR (125 MHz, $CDCl_3$) δ 165.6, 138.8, 128.9, 128.3, 127.1, 80.9, 71.2, 65.2, 56.0, 51.6, 33.3, 28.6.

HRMS (ESI) ($[M + Na]^+$) Calcd. for $[C_{20}H_{27}NaN_3O_4]^+$: 396.1894, Found. 396.1894.

HRMS spectrum of **1m**

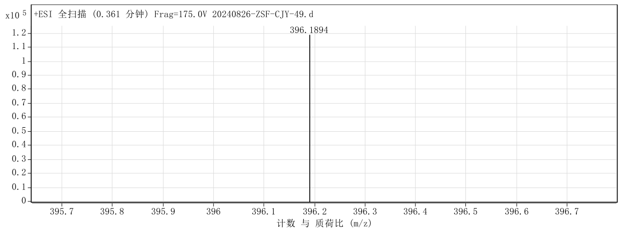

4. General procedure for the cross-coupling reaction of α-diazocarbonyl compounds and aryl boronic acids

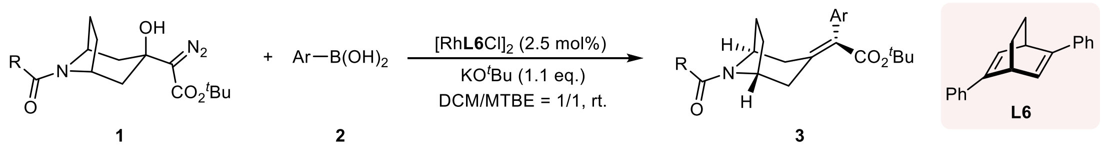

Under nitrogen atmosphere, to a solution of KO^{t}Bu (1.1 eq.) and $ArB(OH)_2$ (2.0 eq.) in DCM/MTBE (0.05 M), 2.5 mol% $[Rh(\mathbf{L6})Cl]_2$ (2.0 mg, dissolved in 0.1 mL DCM) was added. Then, the α-diazoester **1** (0.1 mmol dissolved in another 0.5 mL DCM) was introduced in one portion into the above system. The resulting mixture was stirred at room temperature for 12 h. After the reaction was completed, the reaction mixture was filtered through short silica gel, and then the solvent was removed under reduced

pressure. The crude product was purified by flash column chromatography on silica gel with EtOAc–petroleum ether to yield the products.

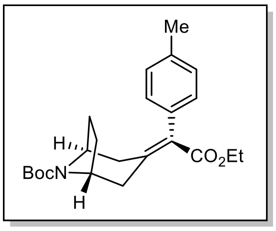

***tert*-butyl-*(1R,5S,Z)*-3-(2-ethoxy-2-oxo-1-(p-tolyl)ethylidene)-8-azabicyclo[3.2.1]octane-8-carboxylate (3a)**

21.1 mg, 55% yield, 55% ee, colorless oil, R_f = 0.55 (PE/EtOAc = 10:1).

^{1}H NMR (400 MHz, $CDCl_3$) δ 7.15 (d, *J* = 7.9 Hz, 2H), 7.09 (d, *J* = 7.9 Hz, 2H), 4.30 (d, *J* = 37.7 Hz, 1H), 4.16 (qd, *J* = 7.1, 2.6 Hz, 3H), 2.80 (d, *J* = 14.9 Hz, 1H), 2.34 (s, 3H), 2.20 (dd, *J* = 14.6, 2.6 Hz, 1H), 1.96 – 1.74 (m, 3H), 1.54 – 1.43 (m, 12H), 1.23 (t, *J* = 7.1 Hz, 3H).

^{13}C NMR (100 MHz, $CDCl_3$) δ 168.8, 153.5, 153.4, 140.5, 137.1, 133.8, 129.5, 129.0, 129.0, 79.5, 60.7, 54.2, 53.7, 38.0, 37.4, 36.9, 28.5, 28.4, 21.2, 14.2.

$[\alpha]_D^{28}$ = -46.2 (c = 0.21, CH_2Cl_2).

HRMS (ESI) ($[M + Na]^+$) Calcd. for $[C_{23}H_{31}NaNO_4]^+$: 408.2145, Found. 408.2139.

HRMS spectrum of **3a**

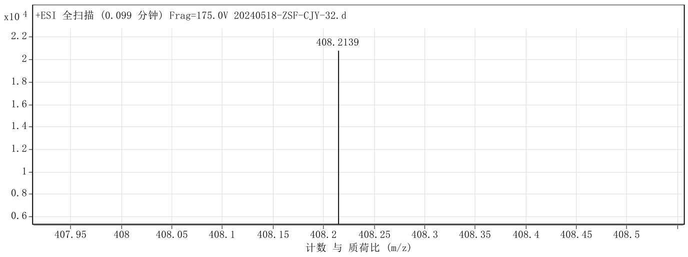

HPLC: AD-H column, hexane/^{i}PrOH = 97:3, 1.0 mL/min, t_R = 7.43 min (minor), 7.96 min (major).

Chiral HPLC spectra of (*rac*)-**3a** and **3a**

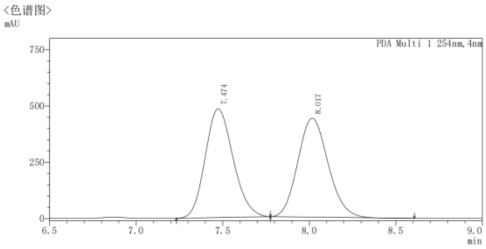

<峰表>
PDA Ch1 254nm

峰号	保留时间	面积	高度	高度%	面积%
1	7.474	5413418	482764	52.371	50.359
2	8.017	5336161	439060	47.629	49.641
总计		10749580	921824	100.000	100.000

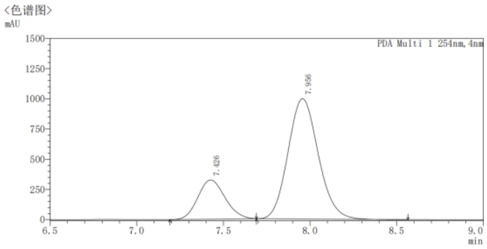

<峰表>
PDA Ch1 254nm

峰号	保留时间	面积	高度	高度%	面积%
1	7.426	3542832	325070	24.581	22.645
2	7.956	12102041	997362	75.419	77.355
总计		15644873	1322432	100.000	100.000

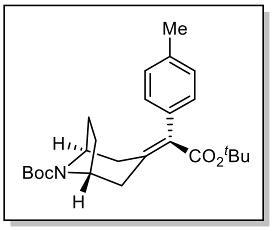

***tert*-butyl-*(1R,5S,Z)*-3-(2-(tert-butoxy)-2-oxo-1-(p-tolyl)ethylidene)-8-azabicyclo[3.2.1]octane-8-carboxylate (3b)**

16.0 mg, 39% yield, 97% ee, colorless oil, R_f = 0.53 (PE/EtOAc = 10:1).

^{1}H NMR (500 MHz, $CDCl_3$) δ 7.12 (d, *J* = 7.7 Hz, 2H), 7.08 (d, *J* = 7.6 Hz, 2H), 4.23 (s, 1H), 4.11 (d, *J* = 52.8 Hz, 1H), 2.71 (d, *J* = 14.7 Hz, 1H), 2.50 (dd, *J* = 57.3, 12.6 Hz, 1H), 2.33 (s, 3H), 2.17 (d, *J* = 14.6 Hz, 1H), 1.95 – 1.73 (m, 3H), 1.48 (s, 2H), 1.46 (s, 9H), 1.43 (s, 9H).

^{13}C NMR (125 MHz, $CDCl_3$) δ 168.5, 153.5, 137.5, 136.9, 135.2, 133.9, 128.9, 128.9, 81.2, 79.4, 54.3, 53.7, 37.9, 37.3, 36.6, 29.7, 28.7, 28.5, 28.4, 28.4, 28.3, 28.1, 27.7, 21.2.

$[\alpha]_D^{28}$ = -81.3 (c = 0.11, CH_2Cl_2).

HRMS (ESI) ($[M + H]^+$) Calcd. for $[C_{25}H_{36}NO_4]^+$: 414.2639, Found. 414.2638.

HRMS spectrum of **3b**

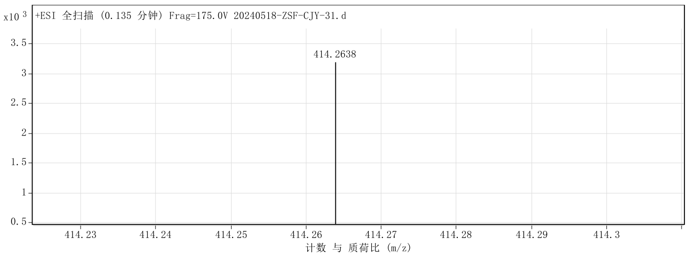

HPLC: INA column, hexane/^{i}PrOH = 97:3, 1.0 mL/min, t_R = 4.43 min (major), 4.89 min (minor).

Chiral HPLC spectra of (*rac*)-**3b** and **3b**

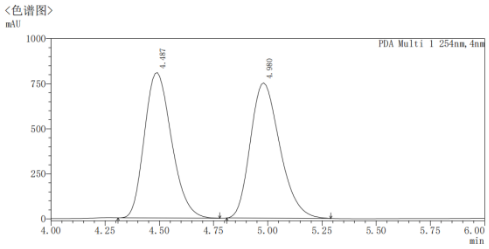

<峰表>
PDA Ch1 254nm

峰号	保留时间	面积	高度	高度%	面积%
1	4.487	7018553	805992	51.815	49.739
2	4.980	7092301	749541	48.185	50.261
总计		14110854	1555533	100.000	100.000

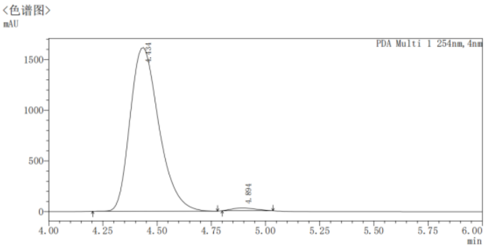

<峰表>
PDA Ch1 254nm

峰号	保留时间	面积	高度	高度%	面积%
1	4.434	14919170	1613326	98.334	98.616
2	4.894	209340	27333	1.666	1.384
总计		15128509	1640659	100.000	100.000

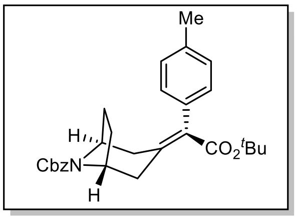

benzyl-*(1R,5S,Z)*-3-(2-(*tert*-butoxy)-2-oxo-1-(p-tolyl)ethylidene)-8-azabicyclo[3.2.1]octane-8-carboxylate (3c)

34.3 mg, 77% yield, 97% ee, colorless oil, R_f = 0.41 (PE/EtOAc = 10:1).

^{1}H NMR (400 MHz, $CDCl_3$) δ 7.46 – 7.30 (m, 5H), 7.14 (d, *J* = 8.0 Hz, 2H), 7.08 (d, *J* = 8.2 Hz, 2H), 5.17 (s, 2H), 4.42 (d, *J* = 18.5 Hz, 1H), 4.25 (d, *J* = 19.8 Hz, 1H), 2.76 (dd, *J* = 14.6, 2.6 Hz, 1H), 2.50 (d, *J* = 2.6 Hz, 1H), 2.41 – 2.29 (m, 4H), 2.27 – 2.17 (m, 2H), 2.00 – 1.78 (m, 3H), 1.44 (s, 9H).

^{13}C NMR (100 MHz, $CDCl_3$) δ 168.4, 153.6, 137.0, 136.9, 135.6, 133.7, 129.4, 129.0, 128.9, 128.5, 128.0, 127.9, 81.3, 72.1, 66.8, 54.1, 38.1, 37.6, 36.9, 28.1, 21.2.

$[\alpha]_D^{28}$ = -72.2 (c = 0.20, CH_2Cl_2).

HRMS (ESI) ($[M + Na]^+$) Calcd. for $[C_{28}H_{33}NaNO_4]^+$: 470.2302, Found. 470.2302.

HRMS spectrum of **3c**

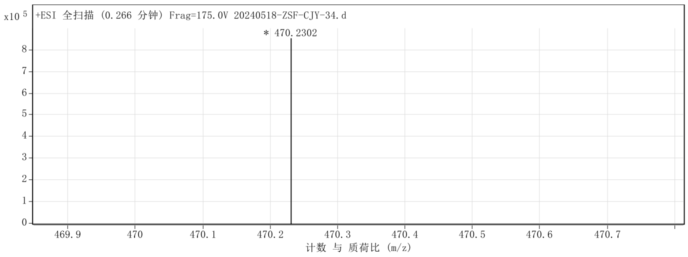

HPLC: INA column, hexane/^{i}PrOH = 97:3, 1.0 mL/min, t_R = 8.87 min (minor), 10.57 min (major).

Chiral HPLC spectra of (*rac*)-**3c** and **3c**

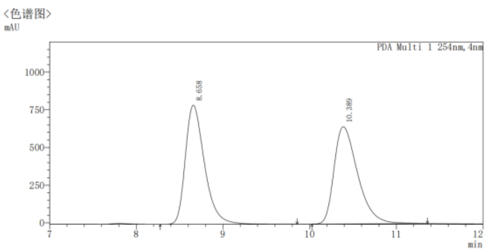

<峰表>
PDA Ch1 254nm

峰号	保留时间	高度	面积	高度%	面积%
1	8.658	790127	12142685	55.049	49.856
2	10.389	645193	12212745	44.951	50.144
总计		1435320	24355430	100.000	100.000

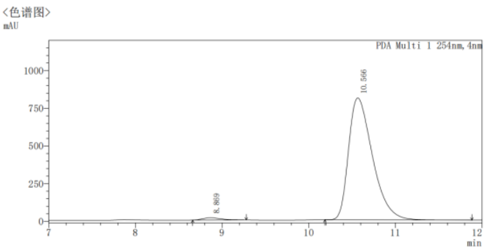

<峰表>
PDA Ch1 254nm

峰号	保留时间	高度	面积	高度%	面积%
1	8.869	15239	221883	1.852	1.411
2	10.566	807750	15498115	98.148	98.589
总计		822989	15719998	100.000	100.000

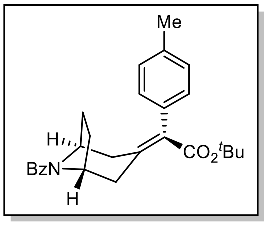

***tert*-butyl-*(Z)*-2-(*(1R,5S)*-8-benzoyl-8-azabicyclo[3.2.1]octan-3-ylidene)-2-(p-tolyl)acetate (3d)**

31.6 mg, 77% yield, 99% ee, colorless oil, R_f = 0.42 (PE/EtOAc = 3:1).

^{1}H NMR (400 MHz, CDCl$_3$) δ 7.57 – 7.36 (m, 5H), 7.22 – 7.05 (m, 4H), 4.86 (d, J = 65.4 Hz, 1H), 4.13 (d, J = 67.5 Hz, 1H), 2.83 (dd, J = 51.6, 14.0 Hz, 1H), 2.61 – 2.45 (m, 1H), 2.35 (d, J = 15.9 Hz, 3H), 2.23 (d, J = 7.5 Hz, 1H), 2.09 – 1.84 (m, 4H), 1.63 (d, J = 5.1 Hz, 1H), 1.45 (d, J = 20.2 Hz, 9H).

^{13}C NMR (100 MHz, CDCl$_3$) δ 168.3, 168.2, 168.2, 168.1, 137.1, 136.4, 136.3, 135.9, 133.7, 133.5, 130.1, 129.0, 128.8, 128.8, 128.5, 128.5, 127.1, 81.4, 81.3, 57.0, 56.9, 52.3, 52.2, 39.2, 38.7, 37.6, 37.0, 28.9, 28.6, 28.1, 27.9, 27.5, 27.2, 21.2.

$[\alpha]_D^{28}$ = -110.1 (c = 0.26, CH_2Cl_2).

HRMS (ESI) ($[M + Na]^+$) Calcd. for $[C_{27}H_{31}NaNO_3]^+$: 440.2196, Found. 440.2196.

HRMS spectrum of **3d**

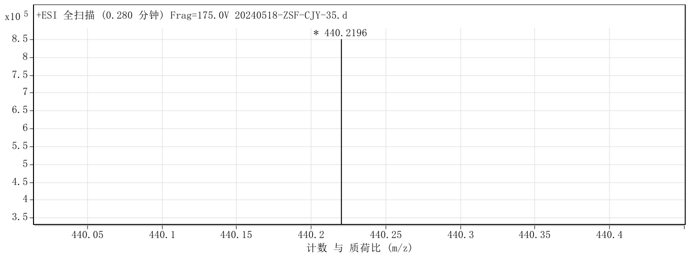

HPLC: AD-H column, hexane/^{i}PrOH = 95:5, 1.0 mL/min, t_R = 23.14 min (minor), 24.72 min (major).

Chiral HPLC spectra of (*rac*)-**3d** and **3d**

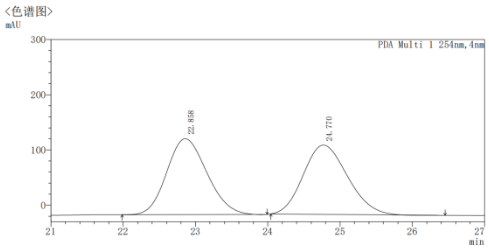

<峰表>
PDA Ch1 254nm

峰号	保留时间	面积	高度	高度%	面积%
1	22.858	5315693	137462	52.292	49.884
2	24.770	5340354	125412	47.708	50.116
总计		10656047	262874	100.000	100.000

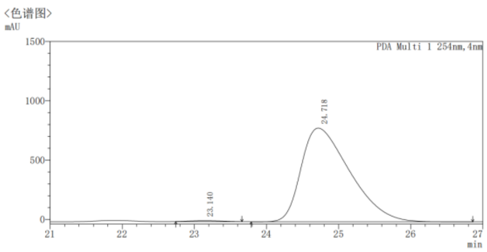

<峰表>
PDA Ch1 254nm

峰号	保留时间	面积	高度	高度%	面积%
1	23.140	224177	7459	0.936	0.602
2	24.718	37021033	789714	99.064	99.398
总计		37245210	797173	100.000	100.000

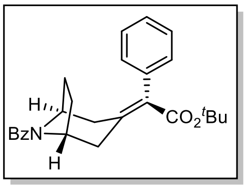

***tert*-butyl *(Z)*-2-(*(1R,5S)*-8-benzoyl-8-azabicyclo[3.2.1]octan-3-ylidene)-2-phenylacetate (3e)**

23.1 mg, 57% yield, 99% ee, colorless oil, R_f = 0.52 (PE/EtOAc = 5:1).

^{1}H NMR (500 MHz, $CDCl_3$) δ 7.34 (ddt, *J* = 51.5, 34.2, 10.9 Hz, 7H), 7.20 – 7.08 (m, 3H), 4.78 (d, *J* = 84.8 Hz, 1H), 4.05 (d, *J* = 82.0 Hz, 1H), 2.71 (dd, *J* = 26.7, 14.7 Hz, 1H), 2.48 (d, *J* = 14.8 Hz, 1H), 2.31 (dd, *J* = 80.5, 14.8 Hz, 1H), 2.19 – 2.06 (m, 1H), 1.99 – 1.75 (m, 3H), 1.56 – 1.43 (m, 1H), 1.36 (d, *J* = 25.4 Hz, 9H).

^{13}C NMR (125 MHz, $CDCl_3$) δ 168.2, 168.0, 137.3, 137.1, 136.7, 136.3, 136.0, 130.0, 128.9, 128.5, 128.3, 127.4, 127.1, 81.5, 81.4, 57.0, 56.9, 52.3, 52.2, 39.1, 38.8, 37.6, 37.1, 29.7, 28.9, 28.6, 28.1, 28.1, 27.9, 27.5, 27.2.

$[\alpha]_D^{28}$ = -122.6 (c = 0.15, CH_2Cl_2).

HRMS (ESI) ($[M + Na]^+$) Calcd. for $[C_{26}H_{29}NaNO_3]^+$: 426.2039, Found. 426.2039.

HRMS spectrum of **3e**

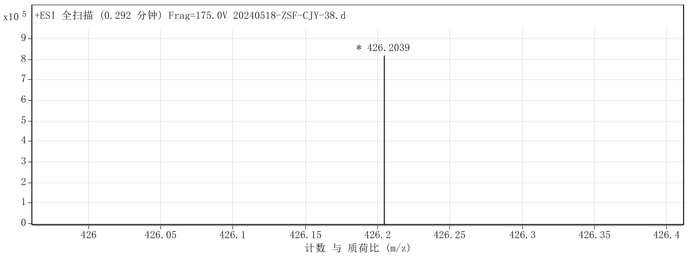

HPLC: INA column, hexane/^{i}PrOH = 97:3, 1.0 mL/min, t_R = 22.63 min (minor), 24.59 min (major).

Chiral HPLC spectra of (*rac*)-**3e** and **3e**

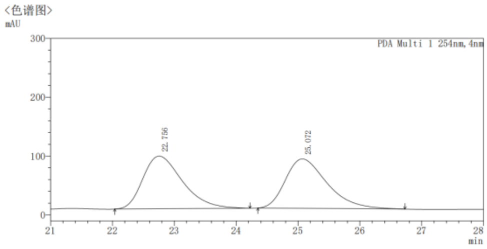

<峰表>
PDA Ch1 254nm

峰号	保留时间	高度	面积	高度%	面积%
1	22.756	89527	3986133	51.614	50.298
2	25.072	83928	3938855	48.386	49.702
总计		173456	7924987	100.000	100.000

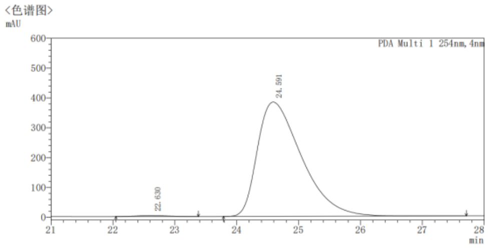

<峰表>
PDA Ch1 254nm

峰号	保留时间	高度	面积	高度%	面积%
1	22.630	3125	109728	0.809	0.574
2	24.591	383229	19012923	99.191	99.426
总计		386355	19122651	100.000	100.000

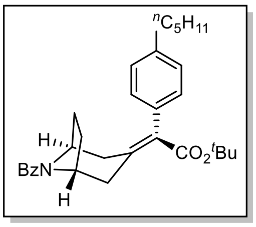

tert-butyl-(Z)-2-((1R,5S)-8-benzoyl-8-azabicyclo[3.2.1]octan-3-ylidene)-2-(4-hexylphenyl)acetate (3f)

34.6 mg, 71% yield, 97% ee, colorless oil, R_f = 0.48 (PE/EtOAc = 3:1).

^{1}H NMR (400 MHz, $CDCl_3$) δ 7.64 – 7.37 (m, 5H), 7.23 – 7.02 (m, 4H), 4.85 (d, *J* = 64.8 Hz, 1H), 4.11 (d, *J* = 61.8 Hz, 1H), δ 2.87 (d, *J* = 14.7 Hz, 1H), 2.74 (d, *J* = 13.3 Hz, 1H), 2.64 – 2.56 (m, 2H), 2.40 (dd, *J* = 44.9, 14.5 Hz, 1H), 2.22 (s, 1H), 1.91 (s, 3H), 1.62 (d, *J* = 22.0 Hz, 4H), 1.43 (d, *J* = 19.4 Hz, 9H), 1.37 – 1.29 (m, 3H), 0.89 (t, *J* = 7.0 Hz, 3H).

^{13}C NMR (125 MHz, $CDCl_3$) δ 168.3, 168.1, 142.2, 136.3, 136.0, 133.8, 133.7, 130.0, 128.8, 128.5, 128.3, 127.1, 81.4, 81.3, 57.0, 56.9, 52.2, 39.2, 38.7, 37.6, 37.0, 35.7, 31.6, 31.0, 29.7, 28.9, 28.6, 28.1, 27.5, 27.2, 22.5, 14.0.

$[\alpha]_D^{28}$ = -101.9 (c = 0.22, CH_2Cl_2).

HRMS (ESI) ($[M + H]^+$) Calcd. for $[C_{26}H_{29}NO_3]^+$: 403.2142, Found. 403.2131.

HRMS spectrum of **3f**

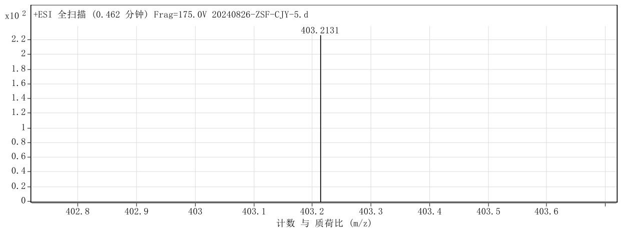

HPLC: AD-H column, hexane/^{i}PrOH = 95:5, 1.0 mL/min, t_R = 16.16 min (minor), 18.15 min (major).

Chiral HPLC spectra of (*rac*)-**3f** and **3f**

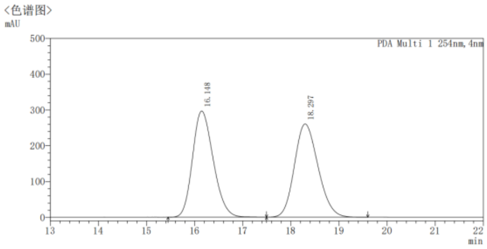

<峰表>
PDA Ch1 254nm

峰号	保留时间	面积	高度	高度%	面积%
1	16.148	8916203	296711	53.251	50.050
2	18.297	8898513	260486	46.749	49.950
总计		17814716	557196	100.000	100.000

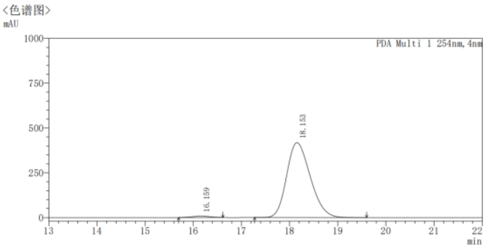

<峰表>
PDA Ch1 254nm

峰号	保留时间	面积	高度	高度%	面积%
1	16.159	205218	7672	1.803	1.419
2	18.153	14257500	417829	98.197	98.581
总计		14462718	425500	100.000	100.000

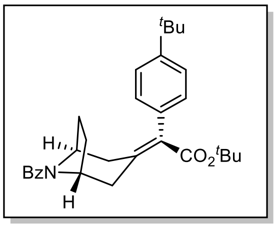

***tert*-butyl-*(Z)*-2-(*(1R,5S)*-8-benzoyl-8-azabicyclo[3.2.1]octan-3-ylidene)-2-(4-(tert-butyl)phenyl)acetate (3g)**

33.6 mg, 73% yield, 97% ee, colorless oil, R_f = 0.53 (PE/EtOAc = 5:1).

^{1}H NMR (400 MHz, $CDCl_3$) δ 7.42 (ddt, *J* = 49.7, 18.1, 8.5 Hz, 7H), 7.22 – 6.99 (m, 2H), 4.80 (d, 1H), 4.13 (d, *J* = 61.8 Hz, 1H), 2.75 (t, 1H), 2.73 (dd, *J* = 117.8, 14.2 Hz, 1H), 2.50 – 2.34 (m, 1H), 2.24 (s, 1H), 2.13 – 1.81 (m, 3H), 1.72 – 1.52 (m, 1H), 1.47 (d, *J* = 20.0 Hz, 9H), 1.33 (d, *J* = 15.6 Hz, 9H).

^{13}C NMR (100 MHz, $CDCl_3$) δ 168.3, 168.1, 150.2, 136.3, 136.2, 136.0, 130.0, 128.5, 128.4, 127.1, 125.2, 81.3, 57.0, 56.9, 52.3, 39.2, 38.7, 37.7, 37.0, 34.5, 31.3, 28.9, 28.6, 28.1, 27.5, 27.2.

$[\alpha]_D^{28}$ = -100.4 (c = 0.24, CH_2Cl_2).

HRMS (ESI) ($[M + Na]^+$) Calcd. for $[C_{30}H_{37}NaNO_3]^+$: 482.2665, Found. 482.2665.

HRMS spectrum of **3g**

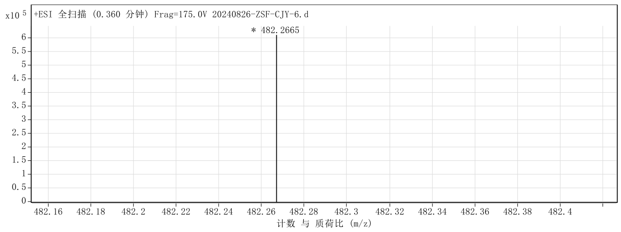

HPLC: AD-H column, hexane/^{i}PrOH = 97:3, 1.0 mL/min, t_R = 21.72 min (major), 23.94 min (minor).

Chiral HPLC spectra of (*rac*)-**3g** and **3g**

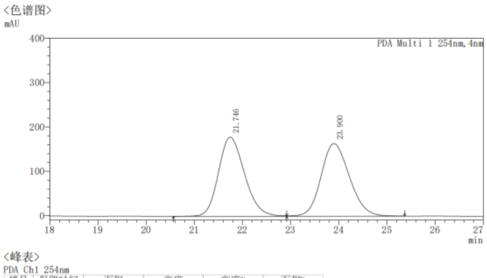

<峰表>
PDA Ch1 254nm

峰号	保留时间	面积	高度	高度%	面积%
1	21.746	6709399	178330	52.150	50.056
2	23.900	6694278	163625	47.850	49.944
总计		13403677	341954	100.000	100.000

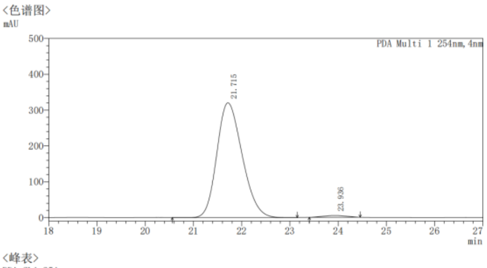

<峰表>
PDA Ch1 254nm

峰号	保留时间	面积	高度	高度%	面积%
1	21.715	11684320	320304	98.449	98.568
2	23.936	169768	5047	1.551	1.432
总计		11854088	325351	100.000	100.000

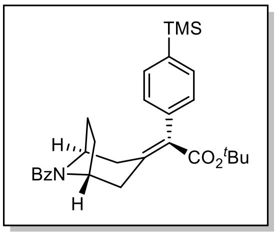

***tert*-butyl-*(Z)*-2-(*(1R,5S)*-8-benzoyl-8-azabicyclo[3.2.1]octan-3-ylidene)-2-(4-(trimethylsilyl)phenyl)acetate (3h)**

37.0 mg, 78% yield, 99% ee, colorless oil, R_f = 0.55 (PE/EtOAc = 5:1).

^{1}H NMR (400 MHz, CDCl$_3$) δ 7.46 (tt, *J* = 21.1, 6.6 Hz, 7H), 7.19 (t, *J* = 8.5 Hz, 2H), 4.79 (d, 1H), 4.14 (d, *J* = 65.5 Hz, 1H), 2.78 (d, *J* = 14.6 Hz, 1H), 2.75 (dd, *J* = 129.6, 14.8 Hz, 1H), 2.38 (dd, 1H), 2.23 (q, *J* = 14.6 Hz, 1H), 2.06 – 1.79 (m, 3H), 1.70 – 1.55 (m, 1H), 1.46 (d, *J* = 19.9 Hz, 9H), 0.28 (d, *J* = 14.6 Hz, 9H).

^{13}C NMR (100 MHz, CDCl$_3$) δ 169.3, 140.6, 138.1, 137.8, 137.4, 137.1, 134.4, 131.2, 129.6, 129.5, 129.3, 128.2, 128.2, 82.7, 82.6, 58.1, 58.0, 53.4, 40.3, 39.9, 38.8, 38.2, 30.0, 29.7, 29.2, 29.0, 28.6, 28.3, 0.0.

$[\alpha]_D^{28}$ = -143.7 (c = -143.7, CH_2Cl_2).

HRMS (ESI) ($[M + Na]^+$) Calcd. for $[C_{29}H_{37}NaNO_3Si]^+$: 498.2435, Found. 498.2430.

HRMS spectrum of **3h**

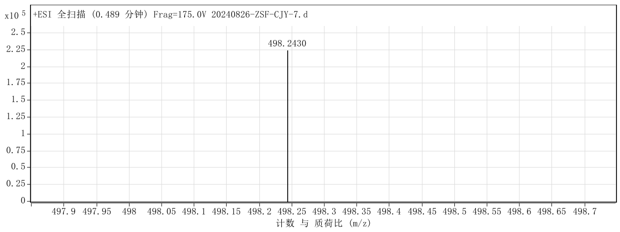

HPLC: INA column, hexane/^{i}PrOH = 99:1, 0.8 mL/min, t_R = 31.86 min (minor), 32.84 min (major).

Chiral HPLC spectra of (*rac*)-**3h** and **3h**

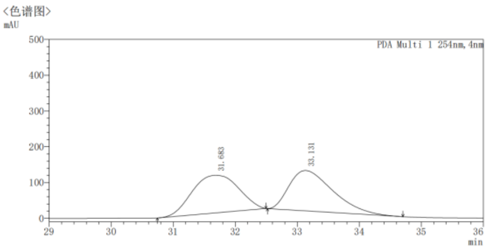

<峰表>
PDA Ch1 254nm

峰号	保留时间	面积	高度	高度%	面积%
1	31.683	5471228	104809	48.164	49.605
2	33.131	5558472	112801	51.836	50.395
总计		11029700	217609	100.000	100.000

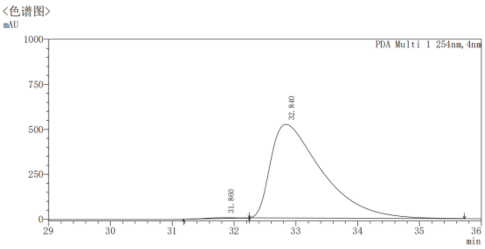

<峰表>
PDA Ch1 254nm

峰号	保留时间	面积	高度	高度%	面积%
1	31.860	119357	4371	0.834	0.396
2	32.840	30019019	519634	99.166	99.604
总计		30138376	524005	100.000	100.000

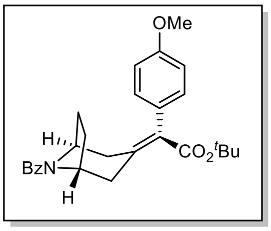

***tert*-butyl-*(Z)*-2-(*(1R,5S)*-8-benzoyl-8-azabicyclo[3.2.1]octan-3-ylidene)-2-(4-methoxyphenyl)acetate (3i)**

32.1 mg, 74% yield, 99% ee, colorless oil, R_f = 0.43 (PE/EtOAc = 5:1).

^{1}H NMR (400 MHz, $CDCl_3$) δ 7.46 (ddt, *J* = 23.7, 17.1, 7.3 Hz, 5H), 7.14 (t, *J* = 8.8 Hz, 2H), 6.88 (dd, *J* = 18.5, 8.2 Hz, 2H), 4.86 (d, *J* = 60.6 Hz, 1H), 4.28 – 3.97 (m, 1H), 3.82 (d, *J* = 14.9 Hz, 3H), 2.81 (dd, *J* = 48.1, 14.7 Hz, 1H), 2.52 (dd, *J* = 42.4, 14.6 Hz, 1H), 2.24 (t, 1H), 1.90 (dd, *J* = 17.2, 10.9 Hz, 4H), 1.59 (d, *J* = 9.8 Hz, 1H), 1.45 (d, *J* = 20.2 Hz, 9H).

^{13}C NMR (100 MHz, $CDCl_3$) δ 168.4, 168.1, 158.8, 136.2, 135.6, 130.1, 128.5, 127.1, 113.7, 113.6, 81.5, 81.3, 57.0, 56.9, 55.2, 52.3, 52.2, 39.2, 38.7, 37.7, 37.0, 28.9, 28.6, 28.1, 27.9, 27.5, 27.2.

$[\alpha]_D^{28}$ = -144.3 (c = 0.22, CH_2Cl_2).

HRMS (ESI) ($[M + Na]^+$) Calcd. for $[C_{27}H_{31}NaNO_4]^+$: 456.2145, Found. 456.2147.

HRMS spectrum of **3i**

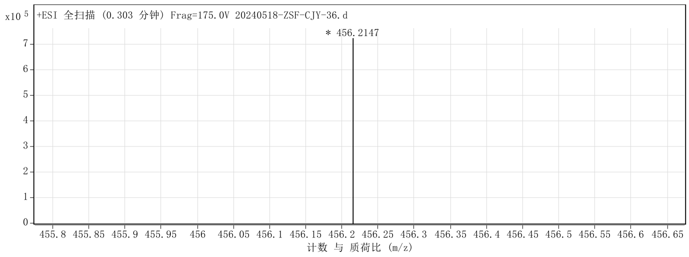

HPLC: AD-H column, hexane/^{i}PrOH = 95:5, 1.0 mL/min, t_R = 37.77 min (major), 40.12 min (minor).

Chiral HPLC spectra of (*rac*)-**3i** and **3i**

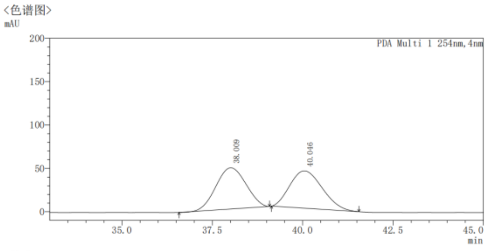

〈峰表〉
PDA Ch1 254nm

峰号	保留时间	面积	高度	高度%	面积%
1	38.009	2741533	47512	52.445	50.441
2	40.046	2693642	43083	47.555	49.559
总计		5435174	90594	100.000	100.000

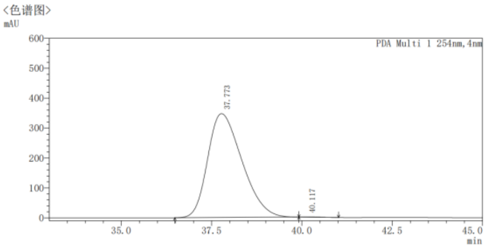

〈峰表〉
PDA Ch1 254nm

峰号	保留时间	面积	高度	高度%	面积%
1	37.773	22937772	347329	99.894	99.939
2	40.117	14000	368	0.106	0.061
总计		22951772	347697	100.000	100.000

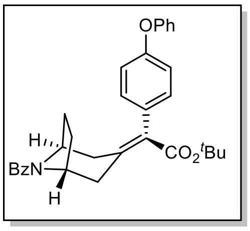

***tert*-butyl-*(Z)*-2-(*(1R,5S)*-8-benzoyl-8-azabicyclo[3.2.1]octan-3-ylidene)-2-(4-phenoxyphenyl)acetate (3j)**

26.1 mg, 53% yield, 93% ee, colorless oil, R_f = 0.63 (PE/EtOAc = 5:1).

^{1}H NMR (400 MHz, $CDCl_3$) δ 7.51 (q, *J* = 6.7 Hz, 2H), 7.47 – 7.39 (m, 3H), 7.40 – 7.31 (m, 2H), 7.16 (q, *J* = 7.8 Hz, 3H), 7.10 – 6.92 (m, 4H), 4.89 (d, *J* = 56.1 Hz, 1H), 4.16 (d, *J* = 54.1 Hz, 1H), 2.79 (s, 1H), 2.75 (dd, *J* = 130.1, 14.5 Hz, 1H), 2.53 – 2.33 (m, 1H), 2.25 (s, 1H), 2.11 – 1.80 (m, 3H), 1.62 (t, *J* = 9.4 Hz, 1H), 1.47 (d, *J* = 19.6 Hz, 9H).

^{13}C NMR (100 MHz, $CDCl_3$) δ 168.2, 168.1, 156.8, 136.3, 135.3, 130.4, 130.1, 129.8, 128.5, 127.1, 123.6, 119.3, 118.3, 81.4, 57.0, 56.9, 52.2, 39.2, 38.8, 37.6, 37.1, 28.9, 28.6, 28.1, 27.9, 27.2, 26.5.

$[\alpha]_D^{28}$ = -118.6 (c = 0.16, CH_2Cl_2).

HRMS (ESI) ($[M + Na]^+$) Calcd. for $[C_{32}H_{33}NaNO_4]^+$: 518.2302, Found. 518.2302.

HRMS spectrum of **3j**

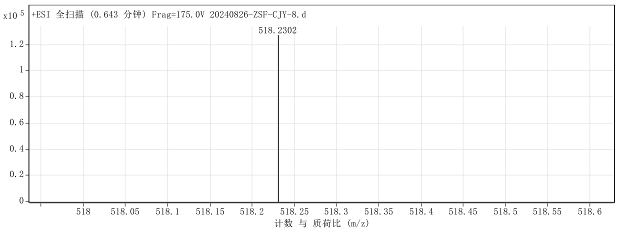

HPLC: AD-H column, hexane/^{i}PrOH = 95:5, 1.0 mL/min, t_R = 31.26 min (minor), 48.28 min (major).

Chiral HPLC spectra of (*rac*)-**3j** and **3j**

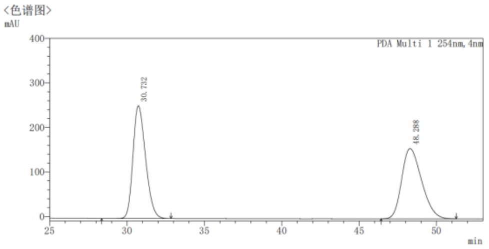

<峰表>
PDA Ch1 254nm

峰号	保留时间	面积	高度	高度%	面积%
1	30.732	14340367	253413	61.605	50.295
2	48.288	14172084	157939	38.395	49.705
总计		28512451	411352	100.000	100.000

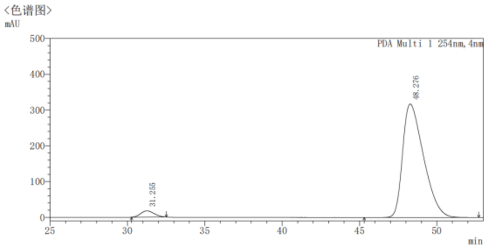

<峰表>
PDA Ch1 254nm

峰号	保留时间	面积	高度	高度%	面积%
1	31.255	1070064	17580	5.251	3.470
2	48.276	29768412	317215	94.749	96.530
总计		30838476	334795	100.000	100.000

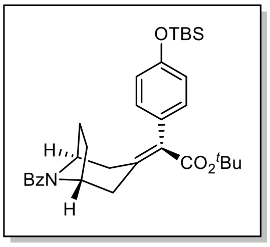

***tert*-butyl-*(Z)*-2-(*(1R,5S)*-8-benzoyl-8-azabicyclo[3.2.1]octan-3-ylidene)-2-(4-((tert-butyldimethylsilyl)oxy)phenyl)acetate (3k)**

35.2 mg, 66% yield, 97% ee, colorless oil, R_f = 0.45 (PE/EtOAc = 5:1).

^{1}H NMR (400 MHz, $CDCl_3$) δ 7.61 – 7.36 (m, 5H), 7.06 (t, *J* = 7.8 Hz, 2H), 6.80 (dd, *J* = 18.3, 8.0 Hz, 2H), 4.87 (d, *J* = 61.3 Hz, 1H), 4.13 (d, *J* = 57.1 Hz, 1H), 2.77 (d, *J* = 12.0 Hz, 1H), 2.72 (dd, *J* = 124.6, 14.6 Hz, 1H), 2.41 (dd, *J* = 40.2, 14.7 Hz, 1H), 2.24 (s, 1H), 2.06 – 1.83 (m, 3H), 1.62 (d, *J* = 7.7 Hz, 1H), 1.44 (d, *J* = 19.7 Hz, 9H), 1.00 (d, *J* = 8.3 Hz, 9H), 0.22 (d, *J* = 13.1 Hz, 6H).

^{13}C NMR (100 MHz, $CDCl_3$) δ 168.3, 168.1, 155.0, 136.3, 135.7, 130.0, 128.5, 127.1, 119.8, 81.4, 81.2, 56.9, 52.1, 39.2, 38.7, 37.6, 37.0, 28.9, 28.6, 28.1, 27.5, 27.2, 25.7, 18.2, -4.4.

$[\alpha]_D^{28}$ = -94.8 (c = 0.25, CH_2Cl_2).

HRMS (ESI) ($[M + Na]^+$) Calcd. for $[C_{32}H_{43}NaNO_4Si]^+$: 556.2853, Found. 556.2853.

HRMS spectrum of **3k**

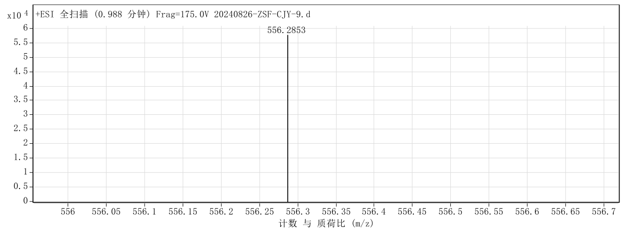

HPLC: AD-H column, hexane/^{i}PrOH = 97:3, 1.0 mL/min, t_R = 13.79 min (major), 15.45 min (minor).

Chiral HPLC spectra of (*rac*)-**3k** and **3k**

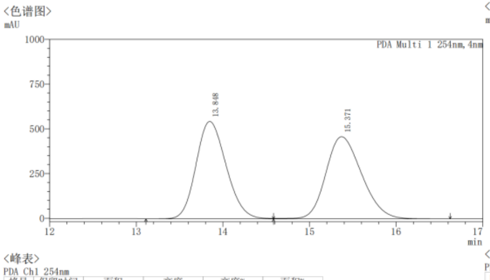

<峰表>
PDA Ch1 254nm

峰号	保留时间	面积	高度	高度%	面积%
1	13.848	13172312	544418	54.245	49.890
2	15.371	13230498	459213	45.755	50.110
总计		26402810	1003631	100.000	100.000

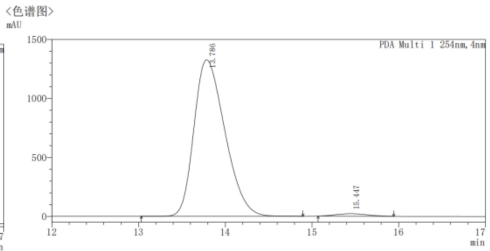

<峰表>
PDA Ch1 254nm

峰号	保留时间	面积	高度	高度%	面积%
1	13.786	32885276	1326264	98.446	98.414
2	15.447	529914	20929	1.554	1.586
总计		33415190	1347194	100.000	100.000

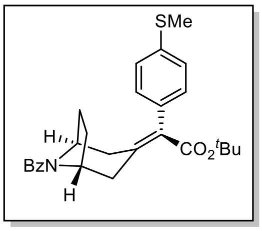

***tert*-butyl-*(Z)*-2-(*(1R,5S)*-8-benzoyl-8-azabicyclo[3.2.1]octan-3-ylidene)-2-(4-(methylthio)phenyl)acetate (3l)**

21.6 mg, 48% yield, 98% ee, colorless oil, R_f = 0.58 (PE/EtOAc = 5:1).

^{1}H NMR (400 MHz, $CDCl_3$) δ 7.47 (ddd, *J* = 34.8, 14.6, 7.2 Hz, 5H), 7.22 (dd, *J* = 16.2, 7.8 Hz, 2H), 7.14 (d, *J* = 7.2 Hz, 2H), 4.87 (d, *J* = 64.1 Hz, 1H), 4.14 (d, *J* = 61.3 Hz, 1H), 2.84 – 2.71 (m, 1H), 2.74 (dd, *J* = 131.4, 14.8 Hz, 1H), 2.50 (d, *J* = 13.7 Hz, 3H), 2.27 (dt, *J* = 39.7, 13.4 Hz, 1H), 1.91 (dd, *J* = 26.1, 14.9 Hz, 3H), 1.74 (s, 1H), 1.58 (d, *J* = 11.7 Hz, 1H), 1.45 (d, *J* = 19.3 Hz, 9H).

^{13}C NMR (100 MHz, $CDCl_3$) δ 168.2, 168.0, 135.4, 130.1, 129.4, 128.5, 127.1, 126.2, 80.1, 56.9, 52.2, 39.2, 38.8, 37.6, 37.1, 28.9, 28.6, 28.1, 27.5, 27.2, 15.6.

$[\alpha]_D^{28}$ = -135.5 (c = 0.17, CH_2Cl_2).

HRMS (ESI) ($[M + H]^+$) Calcd. for $[C_{27}H_{32}NO_3S]^+$: 450.2098, Found. 450.2096.

HRMS spectrum of **3l**

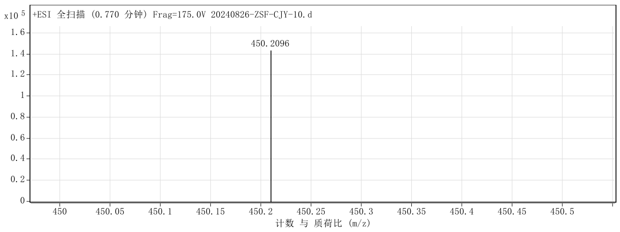

HPLC: REGIS column, hexane/^{i}PrOH = 85:15, 1.0 mL/min, t_R = 48.14 min (minor), 54.28 min (major).

Chiral HPLC spectra of (*rac*)-**3l** and **3l**

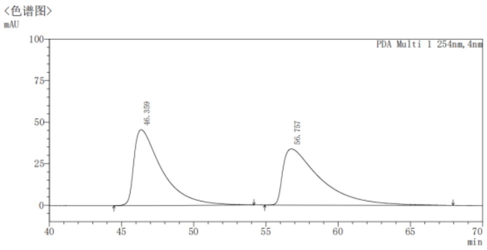

<峰表>
PDA Ch1 254nm

峰号	保留时间	面积	高度	高度%	面积%
1	46.359	6159600	45651	57.427	50.021
2	56.757	6154426	33843	42.573	49.979
总计		12314025	79494	100.000	100.000

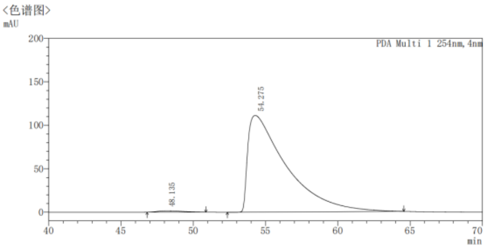

<峰表>
PDA Ch1 254nm

峰号	保留时间	面积	高度	高度%	面积%
1	48.135	173891	1487	1.318	0.833
2	54.275	20713016	111330	98.682	99.167
总计		20886907	112817	100.000	100.000

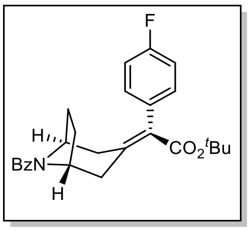

***tert*-butyl-*(Z)*-2-(*(1R,5S)*-8-benzoyl-8-azabicyclo[3.2.1]octan-3-ylidene)-2-(4-fluorophenyl)acetate (3m)**

30.5 mg, 72% yield, 95% ee, colorless oil, R_f = 0.33 (PE/EtOAc = 5:1).

^{1}H NMR (500 MHz, $CDCl_3$) δ 7.64 – 7.36 (m, 5H), 7.19 (t, *J* = 6.9 Hz, 2H), 7.04 (dt, *J* = 17.8, 8.7 Hz, 2H), 4.88 (d, *J* = 63.9 Hz, 1H), 4.15 (d, *J* = 59.8 Hz, 1H), 2.89 (dd, *J* = 42.5, 14.7 Hz, 1H), 2.67 (dd, *J* = 76.0, 14.8 Hz, 1H), 2.31 (dd, *J* = 130.5, 14.5 Hz, 1H), 2.37 – 2.21 (m, 1H), 2.07 – 1.84 (m, 3H), 1.57 (d, *J* = 12.1 Hz, 1H), 1.45 (d, *J* = 19.9 Hz, 9H).

^{13}C NMR (125 MHz, $CDCl_3$) δ 168.2, 168.2, 167.8, 162.08 (d, *J* = 245.7 Hz), 138.2, 137.9, 136.2, 134.9, 130.68 (d, *J* = 6.2 Hz), 130.1, 128.5, 127.10 (d, *J* = 6.1 Hz), 115.31 (dd, *J* = 22.9, 3.6 Hz). 81.7, 81.5, 56.9, 56.9, 52.2, 52.1, 39.1, 38.8, 37.6, 37.1, 29.0, 28.6, 28.1, 27.6, 27.2.

^{19}F NMR (376 MHz, $CDCl_3$) δ -62.8, -114.6, -114.7.

$[\alpha]_D^{28}$ = -62.0 (c = 0.21, CH_2Cl_2).

HRMS (ESI) ($[M + Na]^+$) Calcd. for $[C_{26}H_{28}NaFNO_3]^+$: 444.1945, Found. 444.1945.

HRMS spectrum of **3m**

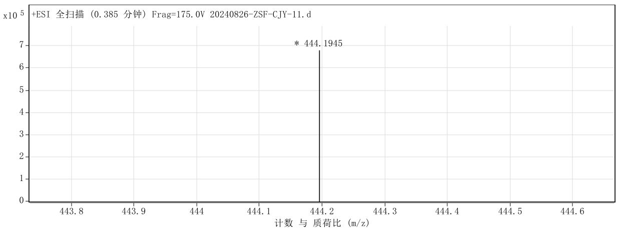

HPLC: AD-H column, hexane/^{i}PrOH = 97:3, 1.0 mL/min, t_R = 24.74 min (minor), 31.82 min (major).

Chiral HPLC spectra of (*rac*)-**3m** and **3m**

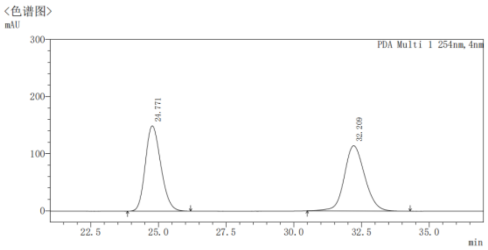

<峰表>
PDA Ch1 254nm

峰号	保留时间	面积	高度	高度%	面积%
1	24.771	5944411	149367	56.662	49.817
2	32.209	5988025	114244	43.338	50.183
总计		11932436	263611	100.000	100.000

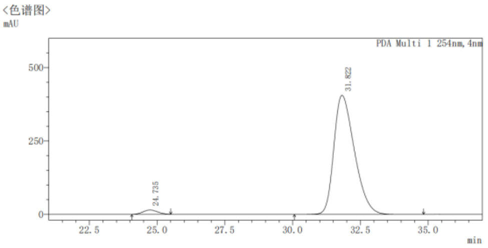

<峰表>
PDA Ch1 254nm

峰号	保留时间	面积	高度	高度%	面积%
1	24.735	550302	14728	3.502	2.504
2	31.822	21423194	405828	96.498	97.496
总计		21973496	420556	100.000	100.000

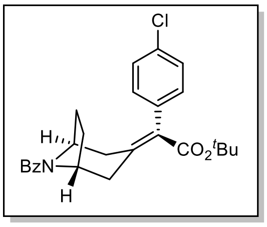

***tert*-butyl-*(Z)*-2-(*(1R,5S)*-8-benzoyl-8-azabicyclo[3.2.1]octan-3-ylidene)-2-(4-chlorophenyl)acetate (3n)**

36.9 mg, 84% yield, 98% ee, colorless oil, R_f = 0.47 (PE/EtOAc = 5:1).

^{1}H NMR (400 MHz, CDCl$_3$) δ 7.46 (ddt, *J* = 23.5, 16.4, 7.2 Hz, 5H), 7.31 (d, *J* = 19.5 Hz, 2H), 7.14 (t, *J* = 7.7 Hz, 2H), 4.79 (d, 1H), 4.16 (d, *J* = 63.8 Hz, 1H), 2.88 (dd, 1H), 2.67 (dd, *J* = 72.5, 14.8 Hz, 1H), δ 2.33 – 2.20 (m, 1H), 2.28 (dd, 1H), 1.93 (ddd, *J* = 42.0, 14.9, 9.1 Hz, 3H), 1.57 (t, *J* = 11.2 Hz, 1H), 1.44 (d, *J* = 20.4 Hz, 9H).

^{13}C NMR (100 MHz, CDCl$_3$) δ 168.2, 167.5, 138.7, 138.4, 136.1, 135.2, 135.1, 134.8, 133.4, 130.4, 130.2, 128.5, 127.1, 81.8, 81.6, 56.9, 56.8, 52.2, 52.1, 39.1, 38.9, 37.6, 37.2, 29.0, 28.6, 28.1, 27.6, 27.2.

$[\alpha]_D^{28}$ = -144.2 (c = 0.27, CH_2Cl_2).

HRMS (ESI) ($[M + Na]^+$) Calcd. for $[C_{26}H_{28}NaClNO_3]^+$: 460.1650, Found. 460.1650.

HRMS spectrum of **3n**

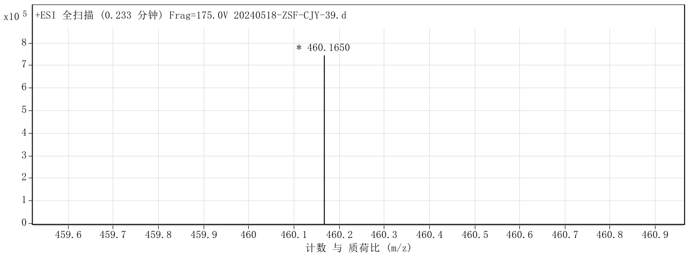

HPLC: AD-H column, hexane/^{i}PrOH = 97:3, 1.0 mL/min, t_R = 40.14 min (minor), 46.10 min (major).

Chiral HPLC spectra of (*rac*)-**3n** and **3n**

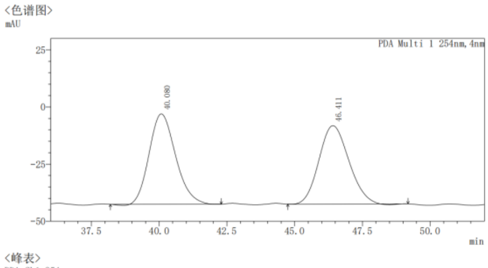

<峰表>
PDA Ch1 254nm

峰号	保留时间	高度	面积	高度%	面积%
1	40.080	39551	2676089	53.541	50.501
2	46.411	34320	2622957	46.459	49.499
总计		73871	5299046	100.000	100.000

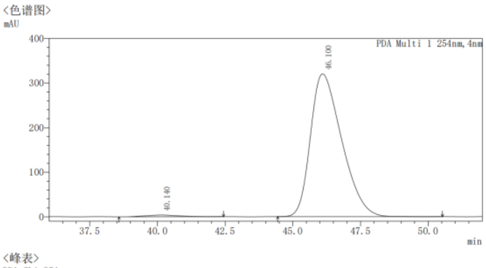

<峰表>
PDA Ch1 254nm

峰号	保留时间	高度	面积	高度%	面积%
1	40.140	3795	300117	1.171	1.159
2	46.100	320181	25600036	98.829	98.841
总计		323975	25900153	100.000	100.000

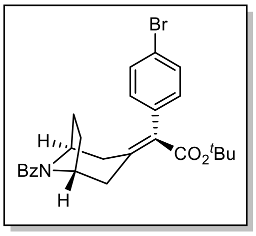

***tert*-butyl-*(Z)*-2-(*(1R,5S)*-8-benzoyl-8-azabicyclo[3.2.1]octan-3-ylidene)-2-(4-bromophenyl)acetate (3o)**

40.8 mg, 84% yield, 98% ee, colorless oil, R_f = 0.46 (PE/EtOAc = 5:1).

^{1}H NMR (400 MHz, $CDCl_3$) δ 7.61 – 7.34 (m, 7H), 7.08 (t, *J* = 7.1 Hz, 2H), 4.87 (d, *J* = 63.8 Hz, 1H), 4.15 (d, *J* = 60.1 Hz, 1H), 3.05 – 2.82 (m, 1H), 2.66 (dd, *J* = 71.4, 14.8 Hz, 1H), 2.30 (dd, *J* = 130.7, 14.5 Hz, 1H), 2.34 – 2.20 (m, 1H), 2.11 – 1.78 (m, 3H), 1.55 (d, *J* = 13.1 Hz, 1H), 1.44 (d, *J* = 19.5 Hz, 9H).

^{13}C NMR (100 MHz, $CDCl_3$) δ 168.2, 167.4, 136.1, 134.8, 131.5, 130.7, 130.1, 128.5, 127.1, 121.6, 81.7, 56.9, 52.1, 39.1, 38.9, 37.6, 37.2, 37.2, 28.6, 28.1, 27.9, 27.6, 27.2.

$[\alpha]_D^{28}$ = -131.2 (c = 0.31, CH_2Cl_2).

HRMS (ESI) ($[M + Na]^+$) Calcd. for $[C_{26}H_{28}NaBrNO_3]^+$: 504.1145, Found. 504.1145.

HRMS spectrum of **3o**

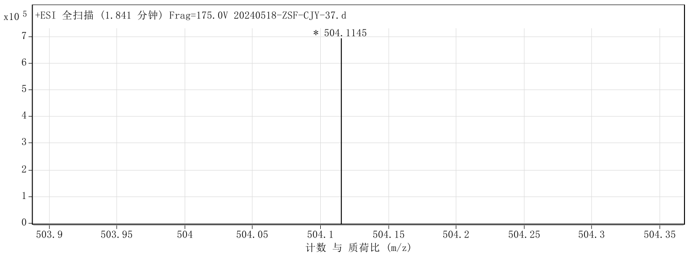

HPLC: INA column, hexane/^{i}PrOH = 97:3, 1.0 mL/min, t_R = 28.15 min (minor), 29.70 min (major).

Chiral HPLC spectra of (*rac*)-**3o** and **3o**

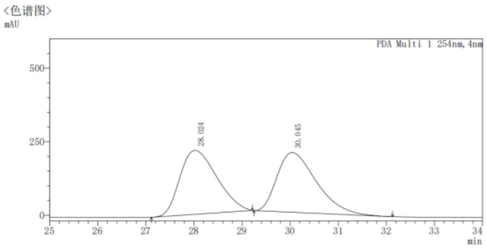

<峰表>
PDA Ch1 254nm

峰号	保留时间	面积	高度	高度%	面积%
1	28.024	11615524	217517	51.681	50.070
2	30.045	11582935	203363	48.319	49.930
总计		23198459	420880	100.000	100.000

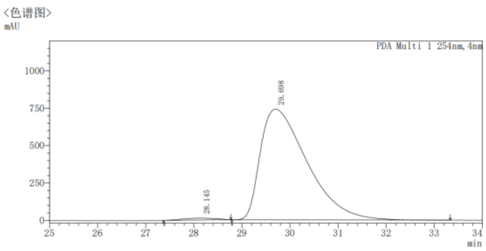

<峰表>
PDA Ch1 254nm

峰号	保留时间	面积	高度	高度%	面积%
1	28.145	600561	12702	1.687	1.185
2	29.698	50075521	740181	98.313	98.815
总计		50676081	752883	100.000	100.000

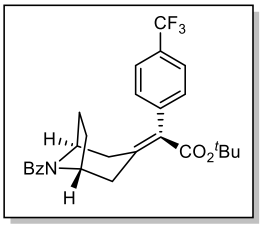

***tert*-butyl-*(Z)*-2-(*(1R,5S)*-8-benzoyl-8-azabicyclo[3.2.1]octan-3-ylidene)-2-(4-(trifluoromethyl)phenyl)acetate (3p)**

37.1 mg, 79% yield, 97% ee, colorless oil, R_f = 0.39 (PE/EtOAc = 5:1).

^{1}H NMR (400 MHz, $CDCl_3$) δ 7.72 – 7.57 (m, 2H), 7.55 – 7.38 (m, 5H), 7.37 – 7.31 (m, 2H), 4.88 (d, *J* = 68.7 Hz, 1H), 4.25 (d, 1H), 2.98 (dd, *J* = 44.0, 14.9 Hz, 1H), 2.70 (dd, *J* = 73.3, 14.8 Hz, 1H), 2.29 (dd, *J* = 162.9, 15.5 Hz, 1H), 2.25 (t, *J* = 17.0 Hz, 1H), 2.04 – 1.83 (m, 3H), 1.56 (d, *J* = 12.5 Hz, 1H), 1.44 (d, *J* = 20.2 Hz, 9H).

^{13}C NMR (125 MHz, $CDCl_3$) δ 168.3, 168.2, 167.1, 140.61 (d, *J* = 15.5 Hz), 139.76 (d, *J* = 28.8 Hz)136.1, 134.7, 134.7, 130.2, 129.7, 129.4, 128.5, 127.10 (d, *J* = 8.1 Hz), 125.3, 123.00 (d, *J* = 4.7 Hz). 82.0, 81.8, 56.9, 56.8, 52.2, 52.1, 39.1, 39.0, 37.5, 37.3, 29.0, 28.7, 28.1, 27.6, 27.2.

^{19}F NMR (376 MHz, $CDCl_3$) δ -62.5.

$[\alpha]_D^{28}$ = -77.1 (c = 0.27, CH_2Cl_2).

HRMS (ESI) ($[M + Na]^+$) Calcd. for $[C_{27}H_{28}NaF_3NO_3]^+$: 494.1913, Found. 494.1912.

HRMS spectrum of **3p**

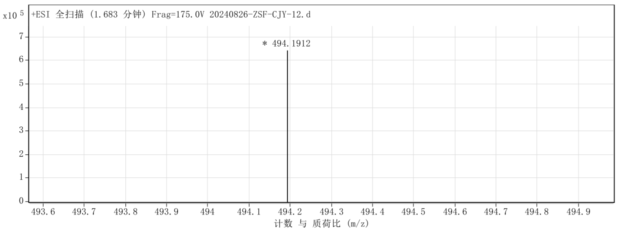

HPLC: AD-H column, hexane/^{i}PrOH = 99:1, 1.0 mL/min, t_R = 66.79 min (minor), 70.21 min (major).

Chiral HPLC spectra of (*rac*)-**3p** and **3p**

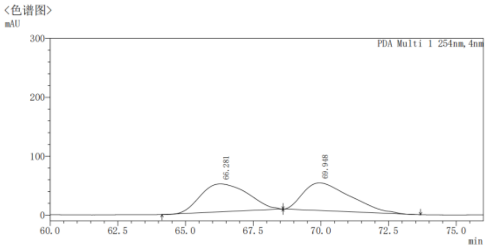

<峰表>
PDA Ch1 254nm

峰号	保留时间	面积	高度	高度%	面积%
1	66.281	5736420	47557	50.347	50.030
2	69.948	5729551	46902	49.653	49.970
总计		11465971	94458	100.000	100.000

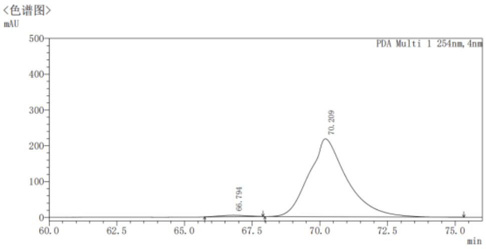

<峰表>
PDA Ch1 254nm

峰号	保留时间	面积	高度	高度%	面积%
1	66.794	283174	3963	1.789	1.310
2	70.209	21335757	217503	98.211	98.690
总计		21618932	221466	100.000	100.000

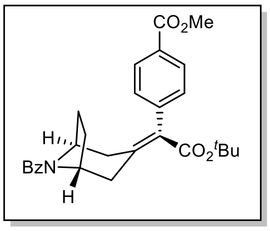

methyl-4-(*(Z)*-1-(*(1R,5S)*-8-benzoyl-8-azabicyclo[3.2.1]octan-3-ylidene)-2-(*tert*-butoxy)-2-oxoethyl)benzoate (3q)

18.4 mg, 40% yield, 95% ee, colorless oil, R_f = 0.29 (PE/EtOAc = 5:1).

^{1}H NMR (400 MHz, CDCl$_3$) δ 8.11 – 7.96 (m, 2H), 7.47 (ddt, *J* = 24.2, 17.0, 6.8 Hz, 5H), 7.29 (d, *J* = 6.8 Hz, 2H), 4.87 (d, *J* = 71.7 Hz, 1H), 4.16 (d, *J* = 66.9 Hz, 1H), 3.93 (d, *J* = 8.2 Hz, 3H), 2.97 (dd, *J* = 39.0, 14.9 Hz, 1H), 2.69 (d, 1H), 2.25 (t, *J* = 13.5 Hz, 1H), 2.30 (dd, *J* = 147.0, 14.8 Hz, 1H), 2.05 – 1.84 (m, 3H), 1.64 – 1.53 (m, 1H), 1.43 (d, *J* = 20.3 Hz, 9H).

^{13}C NMR (100 MHz, CDCl$_3$) δ 168.3, 167.2, 166.8, 141.5, 139.6, 139.1, 136.1, 135.1, 130.2, 129.6, 129.1, 128.5, 127.1, 81.8, 56.9, 52.2, 39.0, 37.5, 37.3, 28.6, 28.0, 27.6, 27.2.

$[\alpha]_D^{28}$ = -55.2 (c = 0.13, CH_2Cl_2).

HRMS (ESI) ($[M + Na]^+$) Calcd. for $[C_{28}H_{31}NaNO_5]^+$: 484.2094, Found. 484.2094.

HRMS spectrum of **3q**

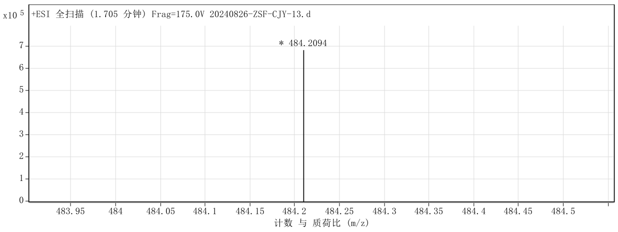

HPLC: AD-H column, hexane/^{i}PrOH = 90:10, 1.0 mL/min, t_R = 9.04 min (minor), 11.53 min (major).

Chiral HPLC spectra of (*rac*)-**3q** and **3q**

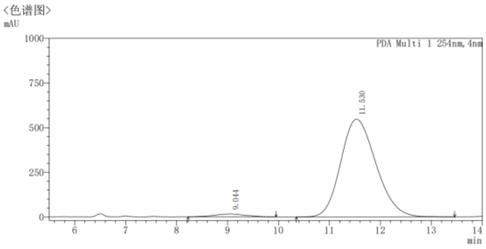

<峰表>

PDA Ch1 254nm

峰号	保留时间	面积	高度	面积%	高度%
1	9.044	694217	15988	2.566	2.840
2	11.530	26357831	546962	97.434	97.160
总计		27052048	562950	100.000	100.000

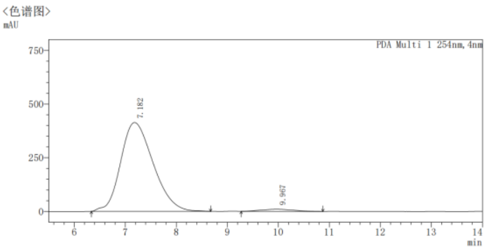

<峰表>

PDA Ch1 254nm

峰号	保留时间	面积	高度	面积%	高度%
1	7.182	18357706	413185	97.633	97.646
2	9.967	445112	9959	2.367	2.354
总计		18802818	423144	100.000	100.000

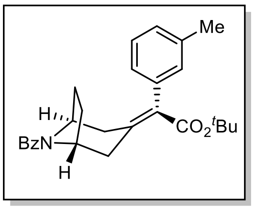

***tert*-butyl-*(Z)*-2-(*(1R,5S)*-8-benzoyl-8-azabicyclo[3.2.1]octan-3-ylidene)-2-(*m*-tolyl)acetate (3r)**

29.1 mg, 70% yield, 98% ee, colorless oil, R_f = 0.34 (PE/EtOAc = 5:1).

^{1}H NMR (400 MHz, $CDCl_3$) δ 7.64 – 7.37 (m, 5H), 7.27 – 7.17 (m, 1H), 7.10 (dd, *J* = 17.4, 7.6 Hz, 1H), 7.03 (t, *J* = 8.2 Hz, 2H), 4.87 (d, *J* = 68.8 Hz, 1H), 4.14 (d, *J* = 64.0 Hz, 1H), 2.84 – 2.71 (m, 1H), 2.74 (dd, *J* = 126.0, 14.7 Hz, 1H), 2.47 (d, *J* = 14.6 Hz, 1H), 2.35 (d, *J* = 13.2 Hz, 3H), 2.21 (t, *J* = 11.8 Hz, 1H), 1.93 (q, *J* = 15.9, 13.5 Hz, 3H), 1.60 (q, *J* = 13.7, 10.1 Hz, 1H), 1.46 (d, *J* = 20.2 Hz, 9H).

^{13}C NMR (100 MHz, $CDCl_3$) δ 168.2, 137.9, 136.6, 136.4, 136.3, 136.1, 130.0, 129.6, 129.5, 128.5, 128.4, 128.2, 127.1, 126.0, 81.4, 56.9, 52.3, 39.2, 38.8, 37.6, 37.1, 29.7, 28.9, 28.6, 28.1, 27.2, 21.4.

$[\alpha]_D^{28}$ = -127.8 (c = 0.24, CH_2Cl_2).

HRMS (ESI) ($[M + Na]^+$) Calcd. for $[C_{27}H_{31}NaNO_3]^+$: 440.2196, Found. 440.2197.

HRMS spectrum of **3r**

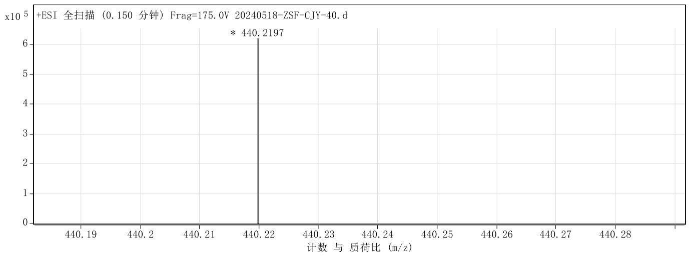

HPLC: AD-H column, hexane/^{i}PrOH = 97:3, 1.0 mL/min, t_R = 22.59 min (minor), 23.62 min (major).

Chiral HPLC spectra of (*rac*)-**3r** and **3r**

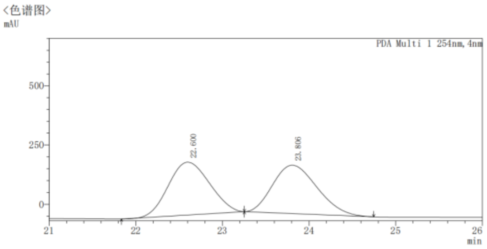

<峰表>
PDA Ch1 254nm

峰号	保留时间	高度	面积	高度%	面积%
1	22.600	223020	7384080	52.198	50.358
2	23.806	204234	7279099	47.802	49.642
总计		427254	14663180	100.000	100.000

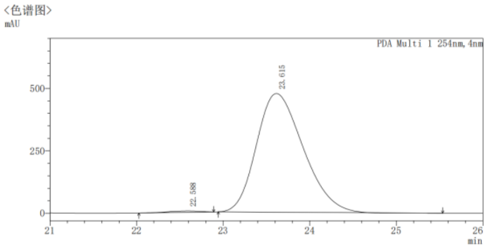

<峰表>
PDA Ch1 254nm

峰号	保留时间	高度	面积	高度%	面积%
1	22.588	5635	140698	1.172	0.783
2	23.615	475129	17835225	98.828	99.217
总计		480764	17975923	100.000	100.000

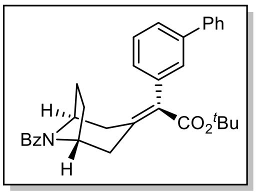

***tert*-butyl-*(Z)*-2-([1,1'-biphenyl]-3-yl)-2-(*(1R,5S)*-8-benzoyl-8-azabicyclo[3.2.1]octan-3-ylidene)acetate (3s)**

25.4 mg, 53% yield, 96% ee, colorless oil, R_f = 0.45 (PE/EtOAc = 5:1).

^{1}H NMR (500 MHz, $CDCl_3$) δ 7.65 – 7.57 (m, 2H), 7.54 (d, J = 6.5 Hz, 2H), 7.51 – 7.43 (m, 6H), 7.39 (dp, J = 11.0, 6.3, 5.5 Hz, 3H), 7.21 (t, J = 9.0 Hz, 1H), 4.89 (d, J = 85.7 Hz, 1H), 4.16 (d, J = 86.7 Hz, 1H), 2.82 (dd, J = 26.5, 14.8 Hz, 1H), 2.81 (dd, J = 157.6, 14.7 Hz, 1H), 2.46 (dd, J = 47.3, 14.7 Hz, 1H), 2.34 – 2.21 (m, 1H), 2.11 – 1.85 (m, 3H), 1.60 (dd, J = 20.4, 11.4 Hz, 1H), 1.47 (d, J = 25.9 Hz, 9H).

^{13}C NMR (125 MHz, $CDCl_3$) δ 168.2, 168.0, 141.2, 140.8, 137.6, 137.4, 137.2, 137.0, 136.3, 136.2, 135.9, 130.1, 128.8, 128.8, 128.5, 127.8, 127.7, 127.5, 127.1, 126.2, 81.6, 81.5, 57.0, 56.9, 52.3, 39.2, 38.9, 37.6, 37.2, 29.7, 29.0, 28.6, 28.1, 27.6, 27.2.

$[\alpha]_D^{28}$ = -121.9 (c = 0.21, CH_2Cl_2).

HRMS (ESI) ($[M + Na]^+$) Calcd. for $[C_{32}H_{33}NaNO_3]^+$: 502.2352, Found. 502.2348.

HRMS spectrum of **3s**

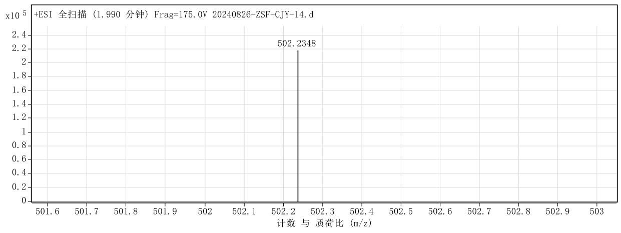

HPLC: AD-H column, hexane/^{i}PrOH = 95:5, 1.0 mL/min, t_R = 14.87 min (minor), 16.18 min (major).

Chiral HPLC spectra of (*rac*)-**3s** and **3s**

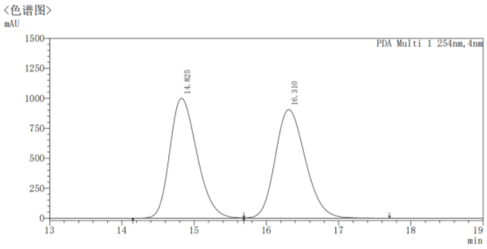

<峰表>
PDA Ch1 254nm

峰号	保留时间	面积	高度	高度%	面积%
1	14.825	26088629	999440	52.478	49.838
2	16.310	26258368	905037	47.522	50.162
总计		52346997	1904477	100.000	100.000

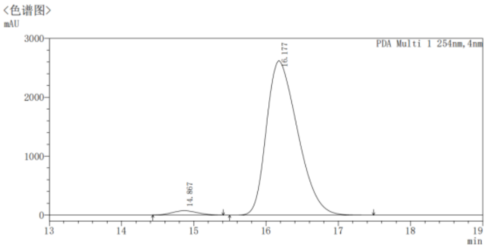

<峰表>
PDA Ch1 254nm

峰号	保留时间	面积	高度	高度%	面积%
1	14.867	1795585	73933	2.741	2.234
2	16.177	78597302	2623418	97.259	97.766
总计		80392887	2697351	100.000	100.000

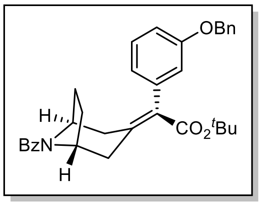

***tert*-butyl-*(Z)*-2-(*(1R,5S)*-8-benzoyl-8-azabicyclo[3.2.1]octan-3-ylidene)-2-(3-(benzyloxy)phenyl)acetate (3t)**

38.9 mg, 76% yield, 99% ee, colorless oil, R_f = 0.46 (PE/EtOAc = 5:1).

^{1}H NMR (400 MHz, $CDCl_3$) δ 7.65 – 7.47 (m, 2H), 7.50 – 7.38 (m, 6H), 7.35 (t, *J* = 7.3 Hz, 2H), 7.28 (d, *J* = 6.5 Hz, 1H), 7.02 – 6.87 (m, 1H), 6.81 (dd, *J* = 14.4, 6.4 Hz, 2H), 5.08 (d, *J* = 8.1 Hz, 2H), 4.86 (d, *J* = 66.1 Hz, 1H), 4.12 (d, *J* = 75.4 Hz, 1H), 2.84 – 2.70 (m, 1H), 2.60 (dd, *J* = 233.0, 14.7 Hz, 1H), 2.51 (dd, *J* = 28.5, 13.6 Hz, 1H), 2.23 – 2.09 (m, 1H), 1.92 (dt, *J* = 27.8, 9.9 Hz, 3H), 1.65 – 1.51 (m, 1H), 1.45 (d, *J* = 20.8 Hz, 9H).

^{13}C NMR (100 MHz, $CDCl_3$) δ 168.2, 168.1, 167.9, 158.6, 158.5, 137.9, 137.3, 137.0, 136.9, 136.2, 135.8, 130.1, 129.4, 129.1, 128.6, 128.6, 128.5, 128.0, 127.6, 127.5, 127.4, 127.1, 121.7, 121.5, 115.4, 114.2, 114.1, 69.9, 57.0, 56.9, 52.3, 52.2, 39.1, 38.7, 37.6, 37.1, 28.9, 28.6, 28.1, 27.9, 27.5, 27.2.

$[\alpha]_D^{28}$ = -85.8 (c = 0.29, CH_2Cl_2).

HRMS (ESI) ($[M + Na]^+$) Calcd. for $[C_{33}H_{35}NaNO_4]^+$: 532.2458, Found. 532.2458.

HRMS spectrum of **3t**

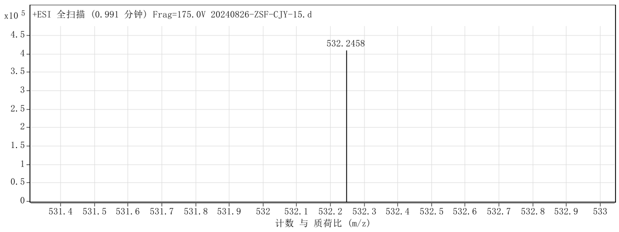

HPLC: INA column, hexane/^{i}PrOH = 97:3, 0.8 mL/min, t_R = 48.77 min (major).

Chiral HPLC spectra of (*rac*)-**3t** and **3t**

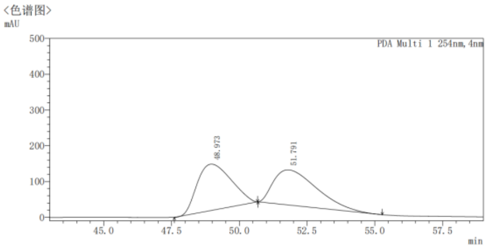

<峰表>

PDA Ch1 254nm

峰号	保留时间	面积	高度	高度%	面积%
1	48.973	11280040	129413	56.917	50.671
2	51.791	10981200	97960	43.083	49.329
总计		22261240	227373	100.000	100.000

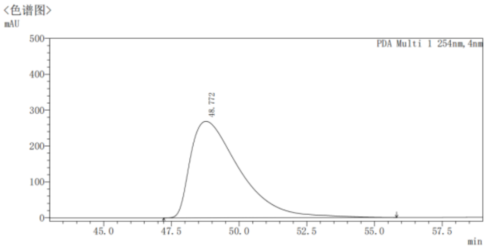

<峰表>

PDA Ch1 254nm

峰号	保留时间	面积	高度	高度%	面积%
1	48.772	34097651	269308	100.000	100.000
总计		34097651	269308	100.000	100.000

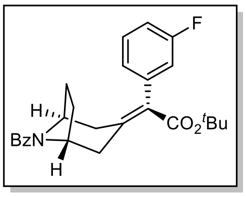

***tert*-butyl-*(Z)*-2-(*(1R,5S)*-8-benzoyl-8-azabicyclo[3.2.1]octan-3-ylidene)-2-(3-fluorophenyl)acetate (3u)**

32.6 mg, 77% yield, 96% ee, colorless oil, R_f = 0.41 (PE/EtOAc = 5:1).

^{1}H NMR (500 MHz, CDCl$_3$) δ 7.47 (ddd, J = 33.5, 15.7, 7.3 Hz, 5H), 7.30 (d, J = 15.2 Hz, 1H), 7.11 – 6.87 (m, 3H), 4.88 (d, J = 62.9 Hz, 1H), 4.16 (d, J = 59.4 Hz, 1H), 2.92 (dd, J = 41.8, 14.8 Hz, 1H), 2.68 (dd, J = 71.9, 14.8 Hz, 1H), 2.35 – 2.22 (m, 1H), 2.32 (dd, J = 128.2, 14.9 Hz, 1H), 2.08 – 1.82 (m, 3H), 1.58 (d, J = 12.8 Hz, 1H), 1.45 (d, J = 19.9 Hz, 9H).

^{13}C NMR (125 MHz, CDCl$_3$) δ 168.2, 168.2, 167.4, 163.6, 161.6, 138.9, 138.9, 138.8, 138.7, 138.6, 136.2, 136.1 (d, J = 4.2 Hz), 134.8, 130.1, 129.9, 129.8 (d, J = 8.2 Hz), 128.5, 127.1, 124.8 (d, J = 6.5 Hz), 116.1, 115.9, 114.5, 114.3 (d, J = 20.5 Hz). 81.8, 81.7, 56.9, 56.8, 52.2, 52.1, 39.1, 38.9, 37.5, 37.2, 29.0, 28.6, 28.1, 27.6, 27.2.

^{19}F NMR (376 MHz, CDCl$_3$) δ -113.0.

$[\alpha]_D^{28}$ = -54.4 (c = 0.23, CH_2Cl_2).

HRMS (ESI) ($[M + Na]^+$) Calcd. for $[C_{26}H_{28}NaFNO_3]^+$: 444.1945, Found. 444.1941.

HRMS spectrum of **3u**

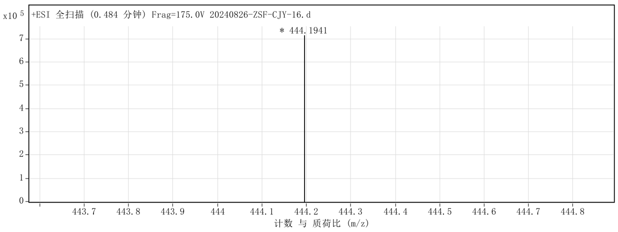

HPLC: AD-H column, hexane/^{i}PrOH = 97:3, 1.0 mL/min, t_R = 20.15 min (minor), 23.01 min (major).

Chiral HPLC spectra of (*rac*)-**3u** and **3u**

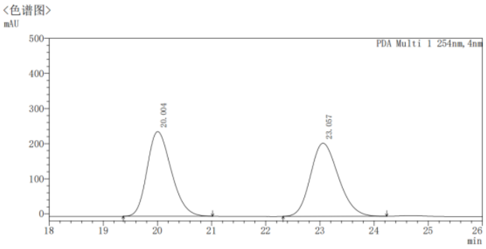

<峰表>
PDA Ch1 254nm

峰号	保留时间	面积	高度	高度%	面积%
1	20.004	7492024	241266	53.677	50.059
2	23.057	7474215	208213	46.323	49.941
总计		14966240	449479	100.000	100.000

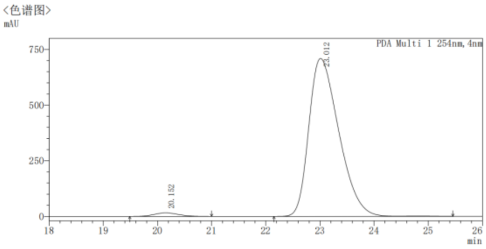

<峰表>
PDA Ch1 254nm

峰号	保留时间	面积	高度	高度%	面积%
1	20.152	490317	16003	2.205	1.763
2	23.012	27316307	709682	97.795	98.237
总计		27806624	725685	100.000	100.000

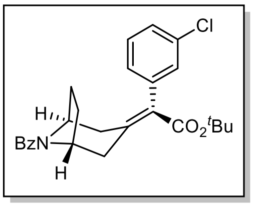

***tert*-butyl-*(Z)*-2-(*(1R,5S)*-8-benzoyl-8-azabicyclo[3.2.1]octan-3-ylidene)-2-(3-chlorophenyl)acetate (3v)**

20.8 mg, 48% yield, 96% ee, colorless oil, R_f = 0.51 (PE/EtOAc = 5:1).

^{1}H NMR (500 MHz, $CDCl_3$) δ 7.47 (ddd, *J* = 32.6, 14.8, 7.0 Hz, 5H), 7.26 (dt, *J* = 19.9, 7.2 Hz, 3H), 7.09 (t, *J* = 4.4 Hz, 1H), 4.88 (d, *J* = 62.4 Hz, 1H), 4.16 (d, *J* = 58.0 Hz, 1H), 2.91 (dd, *J* = 40.5 Hz, 1H), 2.68 (dd, *J* = 66.4, 14.8 Hz, 1H), 2.31 (dd, *J* = 132.3, 14.6 Hz, 1H), 2.27 (d, *J* = 15.4 Hz, 1H), 1.95 (ddd, *J* = 32.6, 24.8, 15.9 Hz, 3H), 1.58 (d, *J* = 13.5 Hz, 1H), 1.45 (d, *J* = 20.1 Hz, 9H).

^{13}C NMR (125 MHz, $CDCl_3$) δ 168.3, 168.2, 167.3, 139.2, 138.9, 138.6, 138.5, 136.1, 134.6, 134.1, 130.2, 129.6, 129.1, 128.5, 127.6, 127.1, 81.9, 81.7, 56.9, 56.8, 52.1, 39.1, 38.9, 37.5, 37.3, 29.0, 28.6, 28.1, 28.1, 27.6, 27.2.

$[\alpha]_D^{28}$ = -117.7 (c = 0.16, CH_2Cl_2).

HRMS (ESI) ($[M + Na]^+$) Calcd. for $[C_{26}H_{28}NaClNO_3]^+$: 460.1650, Found. 460.1650.

HRMS spectrum of **3v**

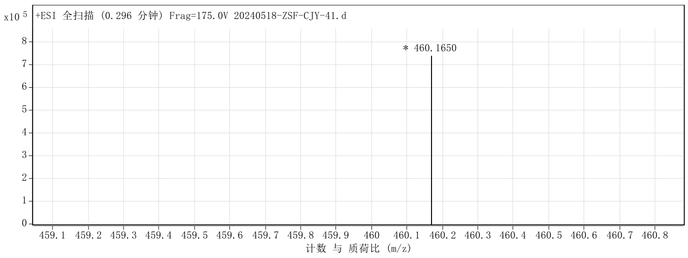

HPLC: AD-H column, hexane/^{i}PrOH = 98:2, 0.8 mL/min, t_R = 40.35 min (minor), 44.22 min (major).

Chiral HPLC spectra of (*rac*)-**3v** and **3v**

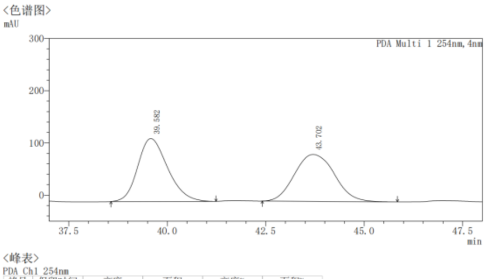

<峰表>
PDA Ch1 254nm

峰号	保留时间	高度	面积	高度%	面积%
1	39.582	120564	6360263	57.290	50.141
2	43.702	89881	6324587	42.710	49.859
总计		210445	12684851	100.000	100.000

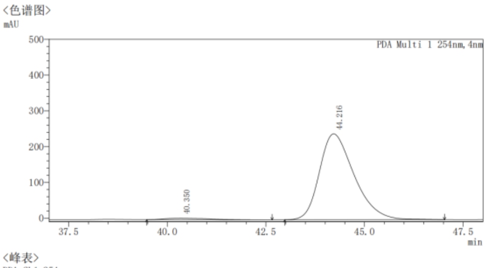

<峰表>
PDA Ch1 254nm

峰号	保留时间	高度	面积	高度%	面积%
1	40.350	4319	317327	1.769	2.113
2	44.216	239827	14697052	98.231	97.887
总计		244146	15014379	100.000	100.000

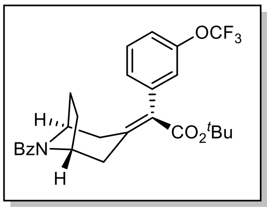

***tert*-butyl-*(Z)*-2-(*(1R,5S)*-8-benzoyl-8-azabicyclo[3.2.1]octan-3-ylidene)-2-(3-(trifluoromethoxy)phenyl)acetate (3w)**

35.1 mg, 72% yield, 97% ee, colorless oil, R_f = 0.49 (PE/EtOAc = 5:1).

^{1}H NMR (500 MHz, CDCl$_3$) δ 7.45 (tdd, *J* = 26.8, 15.9, 7.3 Hz, 6H), 7.14 (dq, *J* = 15.7, 8.3 Hz, 3H), 4.88 (d, *J* = 61.9 Hz, 1H), 4.17 (d, *J* = 60.1 Hz, 1H), 2.97 (dd, *J* = 40.9, 14.7 Hz, 1H), 2.69 (dd, *J* = 65.0, 14.9 Hz, 1H), 2.31 (dd, *J* = 142.1, 14.8 Hz, 1H), 2.27 (d, *J* = 14.7 Hz, 1H), 2.07 – 1.82 (m, 3H), 1.64 – 1.51 (m, 1H), 1.44 (d, *J* = 19.3 Hz, 9H).

^{13}C NMR (125 MHz, CDCl$_3$) δ 168.3, 168.2, 167.1, 149.1, 139.67 (d, *J* = 45.4 Hz), 138.80 (d, *J* = 15.2 Hz), 136.1, 134.5, 130.1, 129.7, 128.5, 127.5, 127.1, 121.6, 121.5, 119.88 (d, *J* = 11.7 Hz), 119.4, 81.9, 81.7, 56.9, 52.2, 52.1, 39.1, 38.9, 37.5, 37.3, 29.0, 28.6, 28.0, 27.6, 27.2.

^{19}F NMR (376 MHz, CDCl$_3$) δ -57.9.

$[\alpha]_D^{28}$ = -102.4 (c = 0.25, CH_2Cl_2).

HRMS (ESI) ($[M + Na]^+$) Calcd. for $[C_{27}H_{28}NaF_3NO_4]^+$: 510.1862, Found. 510.1859.

HRMS spectrum of **3w**

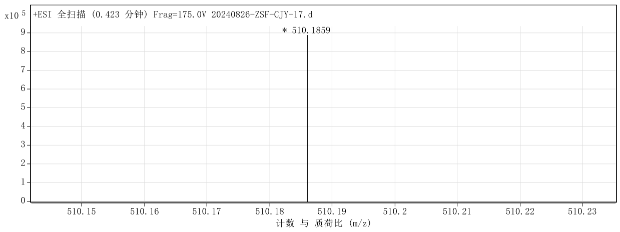

HPLC: INA column, hexane/^{i}PrOH = 95:5, 1.0 mL/min, t_R = 9.47 min (minor), 10.17 min (major).

Chiral HPLC spectra of (*rac*)-**3w** and **3w**

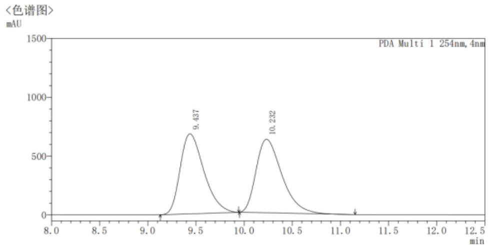

<峰表>
PDA Ch1 254nm

峰号	保留时间	面积	高度	高度%	面积%
1	9.437	11681005	679409	52.124	50.048
2	10.232	11658769	624044	47.876	49.952
总计		23339774	1303452	100.000	100.000

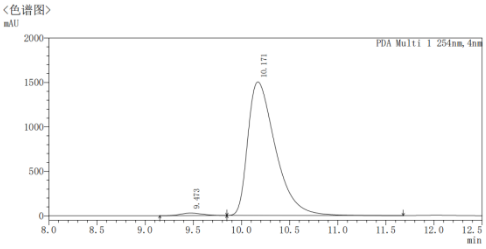

<峰表>
PDA Ch1 254nm

峰号	保留时间	面积	高度	高度%	面积%
1	9.473	464340	28901	1.887	1.526
2	10.171	29972450	1503063	98.113	98.474
总计		30436790	1531963	100.000	100.000

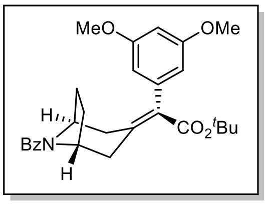

***tert*-butyl-*(Z)*-2-(*(1R,5S)*-8-benzoyl-8-azabicyclo[3.2.1]octan-3-ylidene)-2-(3,5-dimethoxyphenyl)acetate (3x)**

34.7 mg, 75% yield, 97% ee, colorless oil, R_f = 0.35 (PE/EtOAc = 5:1).

^{1}H NMR (400 MHz, CDCl$_3$) δ 7.45 (ddq, *J* = 23.5, 14.8, 7.0 Hz, 5H), 6.38 (t, *J* = 11.9 Hz, 3H), 4.87 (d, *J* = 61.2 Hz, 1H), 4.14 (d, *J* = 58.4 Hz, 1H), 3.78 (d, *J* = 9.7 Hz, 6H), 2.75 (d, *J* = 13.4 Hz, 1H), 2.73 (dd, *J* = 116.4, 14.7 Hz, 1H), 2.42 (dd, *J* = 27.1, 14.7 Hz, 1H), 2.25 (s, 1H), 2.05 – 1.78 (m, 3H), 1.61 (d, *J* = 13.6 Hz, 1H), 1.46 (d, *J* = 19.3 Hz, 9H).

^{13}C NMR (100 MHz, CDCl$_3$) δ 168.2, 167.8, 160.6, 137.0, 136.2, 135.9, 130.1, 128.5, 127.1, 107.0, 99.7, 99.1, 81.4, 56.9, 56.9, 55.4, 52.3, 39.2, 38.8, 37.6, 37.1, 28.9, 28.6, 28.6, 28.1, 27.5, 27.5, 27.2, 27.2.

$[\alpha]_D^{28}$ = -30.0 (c = 0.05, CH_2Cl_2).

HRMS (ESI) ($[M + Na]^+$) Calcd. for $[C_{28}H_{33}NaNO_5]^+$: 486.2251, Found. 486.2245.

HRMS spectrum of **3x**

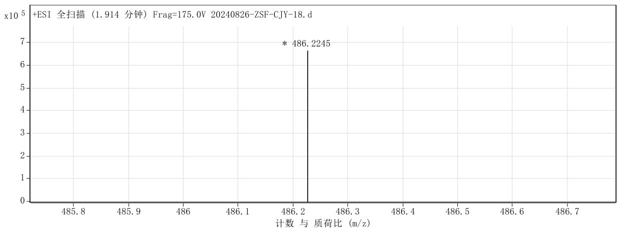

HPLC: INA column, hexane/^{i}PrOH = 95:5, 1.0 mL/min, t_R = 17.27 min (minor), 19.06 min (major).

Chiral HPLC spectra of (*rac*)-**3x** and **3x**

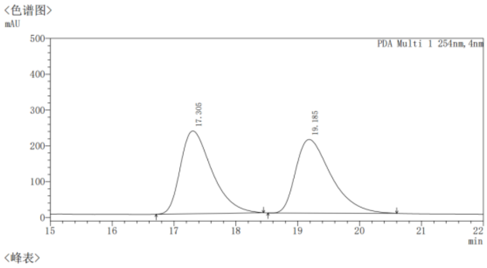

<峰表>

PDA Ch1 254nm

峰号	保留时间	面积	高度	高度%	面积%
1	17.305	8181148	231284	52.920	49.994
2	19.185	8183275	205763	47.080	50.006
总计		16364422	437046	100.000	100.000

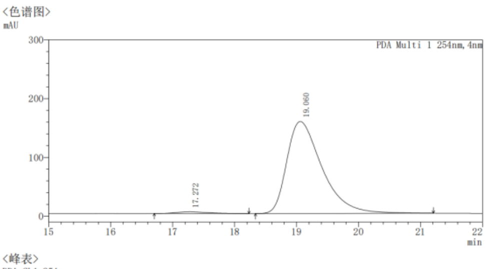

<峰表>

PDA Ch1 254nm

峰号	保留时间	面积	高度	高度%	面积%
1	17.272	102074	2865	1.798	1.628
2	19.060	6167641	156467	98.202	98.372
总计		6269715	159333	100.000	100.000

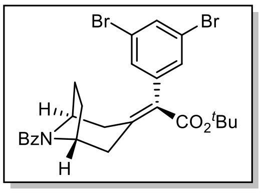

***tert*-butyl-*(Z)*-2-(*(1R,5S)*-8-benzoyl-8-azabicyclo[3.2.1]octan-3-ylidene)-2-(3,5-dibromophenyl)acetate (3y)**

42.6 mg, 76% yield, 97% ee, colorless oil, R_f = 0.53 (PE/EtOAc = 5:1).

^{1}H NMR (500 MHz, $CDCl_3$) δ 7.61 (d, *J* = 13.7 Hz, 1H), 7.54 – 7.48 (m, 2H), 7.45 (d, *J* = 8.8 Hz, 3H), 7.35 – 7.29 (m, 2H), 4.86 (d, 1H), 4.24 (d, 1H), 2.96 (dd, *J* = 36.1, 14.8 Hz, 1H), 2.70 (dd, *J* = 46.7, 14.8 Hz, 1H), 2.28 (d, *J* = 14.2 Hz, 1H), 2.23 (dd, 1H), 2.07 – 1.92 (m, 2H), 1.90 – 1.73 (m, 1H), 1.56 (d, *J* = 10.1 Hz, 1H), 1.45 (d, *J* = 19.7 Hz, 9H).

^{13}C NMR (125 MHz, $CDCl_3$) δ 168.3, 168.2, 166.6, 141.2, 140.6, 140.3, 136.0, 133.2, 133.0, 130.8, 130.2, 129.0, 128.5, 127.1, 122.8, 82.1, 56.7, 52.1, 39.1, 37.4, 29.7, 29.1, 28.6, 28.1, 27.7, 27.2.

$[\alpha]_D^{28}$ = -69.0 (c = 0.33, CH_2Cl_2).

HRMS (ESI) ($[M + Na]^+$) Calcd. for $[C_{26}H_{27}NaBr_2NO_3]^+$: 582.0250, Found. 582.0229.

HRMS spectrum of **3y**

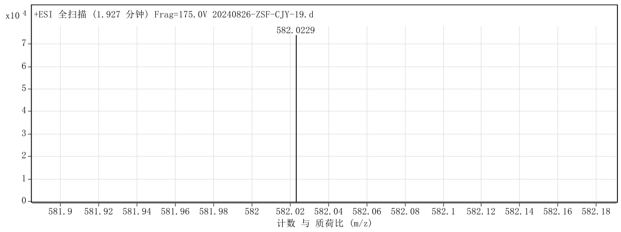

HPLC: INA column, hexane/^{i}PrOH = 99:1, 1.0 mL/min, t_R = 25.89 min (minor), 27.88 min (major).

Chiral HPLC spectra of (*rac*)-**3y** and **3y**

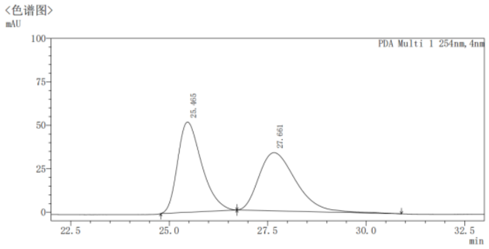

<峰表>
PDA Ch1 254nm

峰号	保留时间	面积	高度	高度%	面积%
1	25.465	2217668	51958	60.803	50.207
2	27.661	2199393	33495	39.197	49.793
总计		4417062	85454	100.000	100.000

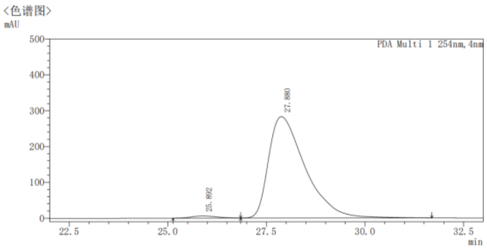

<峰表>
PDA Ch1 254nm

峰号	保留时间	面积	高度	高度%	面积%
1	25.892	269539	6106	2.111	1.456
2	27.880	18248225	283079	97.889	98.544
总计		18517764	289185	100.000	100.000

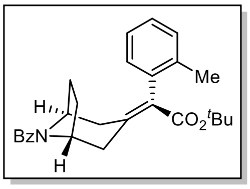

***tert*-butyl-*(Z)*-2-(*(1R,5S)*-8-benzoyl-8-azabicyclo[3.2.1]octan-3-ylidene)-2-(o-tolyl)acetate (3z)**

39.2 mg, 94% yield, 94% ee, colorless oil, R_f = 0.54 (PE/EtOAc = 5:1).

^{1}H NMR (500 MHz, CDCl$_3$) δ 7.61 – 7.36 (m, 5H), 7.24 – 6.92 (m, 4H), 4.87 (d, *J* = 88.5 Hz, 1H), 4.14 (d, *J* = 81.5 Hz, 1H), 3.48 – 2.95 (m, 1H), 2.84 – 2.48 (m, 1H), 2.48 – 2.36 (m, 1H), 2.25 (d, *J* = 52.8 Hz, 3H), 1.98 (dt, *J* = 39.8, 15.0 Hz, 4H), 1.62 (dd, *J* = 23.6, 11.5 Hz, 1H), 1.54 – 1.33 (m, 9H).

^{13}C NMR (125 MHz, CDCl$_3$) δ 168.1, 167.5, 167.0, 136.7, 136.3, 134.8, 130.1, 129.9, 128.9, 128.4, 127.5, 127.1, 125.7, 81.0, 57.0, 56.7, 52.2, 51.9, 38.4, 37.2, 29.2, 29.0, 28.6, 28.6, 28.1, 27.9, 27.3, 27.1, 19.7, 19.5.

$[\alpha]_D^{28}$ = -67.5 (c = 0.29, CH_2Cl_2).

HRMS (ESI) ([M + Na]$^+$) Calcd. for [$C_{27}H_{31}NaNO_3$]$^+$: 440.2196, Found. 440.2188.

HRMS spectrum of **3z**

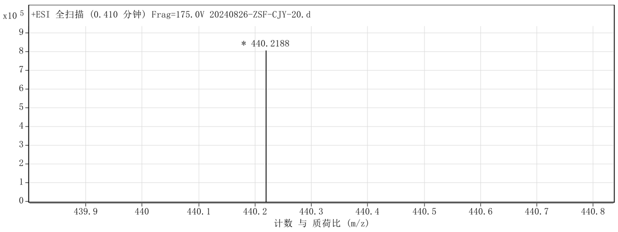

HPLC: AD-H column, hexane/^{i}PrOH = 97:3, 1.0 mL/min, t_R = 18.04 min (major), 19.56 min (minor).

Chiral HPLC spectra of (*rac*)-**3z** and **3z**

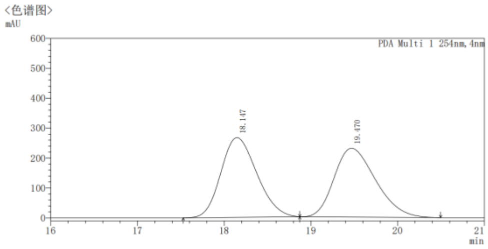

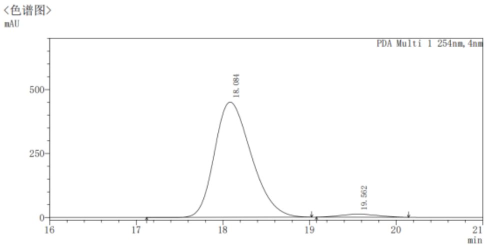

<峰表>
PDA Ch1 254nm

峰号	保留时间	面积	高度	高度%	面积%
1	18.147	7680207	266254	53.654	50.151
2	19.470	7633958	229986	46.346	49.849
总计		15314166	496240	100.000	100.000

<峰表>
PDA Ch1 254nm

峰号	保留时间	面积	高度	高度%	面积%
1	18.084	13290691	449886	97.440	97.399
2	19.562	354860	11820	2.560	2.601
总计		13645551	461706	100.000	100.000

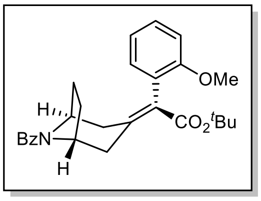

***tert*-butyl-*(Z)*-2-(*(1R,5S)*-8-benzoyl-8-azabicyclo[3.2.1]octan-3-ylidene)-2-(2-methoxyphenyl)acetate (3aa)**

21.7 mg, 50% yield, 98% ee, colorless oil, R_f = 0.52 (PE/EtOAc = 3:1).

^{1}H NMR (400 MHz, CDCl$_3$) δ 7.61 – 7.35 (m, 5H), 7.33 – 7.22 (m, 1H), 7.08 (s, 1H), 6.97 – 6.84 (m, 2H), 4.88 (d, *J* = 75.2 Hz, 1H), 4.13 (d, *J* = 69.4 Hz, 1H), 3.95 – 3.75 (m, 3H), 3.33 (s, 1H), 2.81 – 2.49 (m, 1H), 2.25 (d, *J* = 14.8 Hz, 1H), 1.97 (d, *J* = 20.0 Hz, 3H), 1.80 – 1.62 (m, 2H), 1.55 – 1.31 (m, 9H).

^{13}C NMR (100 MHz, CDCl$_3$) δ 168.1, 167.2, 157.0, 141.8, 136.4, 131.0, 130.0, 128.9, 128.4, 127.1, 120.3, 114.6, 110.7, 80.5, 57.1, 55.4, 52.1, 28.6, 28.0, 27.9, 27.4, 27.2.

$[\alpha]_D^{28}$ = -82.1 (c = 0.12, CH_2Cl_2).

HRMS (ESI) ($[M + Na]^+$) Calcd. for $[C_{27}H_{31}NaNO_4]^+$: 456.2145, Found. 456.2138.

HRMS spectrum of **3aa**

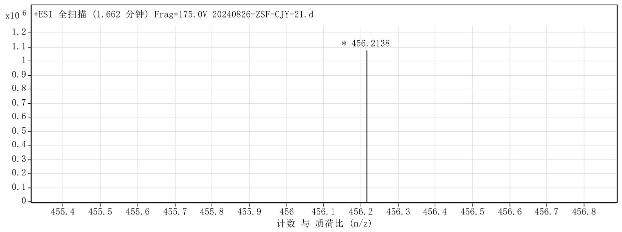

HPLC: AD-H column, hexane/^{i}PrOH = 94:6, 0.6 mL/min, t_R = 25.80 min (minor), 26.71 min (major).

Chiral HPLC spectra of (*rac*)-**3aa** and **3aa**

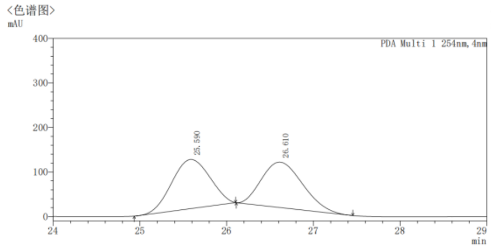

<峰表>
PDA Ch1 254nm

峰号	保留时间	面积	高度	高度%	面积%
1	25.590	3364405	110257	51.944	49.995
2	26.610	3365019	102005	48.056	50.005
总计		6729424	212262	100.000	100.000

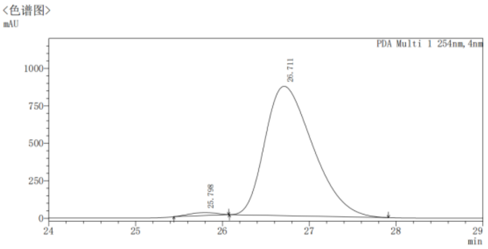

<峰表>
PDA Ch1 254nm

峰号	保留时间	面积	高度	高度%	面积%
1	25.798	433477	19554	2.213	1.288
2	26.711	33228304	863959	97.787	98.712
总计		33661780	883514	100.000	100.000

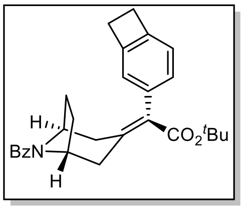

***tert*-butyl-*(Z)*-2-(*(1R,5S)*-8-benzoyl-8-azabicyclo[3.2.1]octan-3-ylidene)-2-(bicyclo[4.2.0]octa-1(6),2,4-trien-3-yl)acetate (3ab)**

26.1 mg, 61% yield, 98% ee, colorless oil, R_f = 0.52 (PE/EtOAc = 3:1).

^{1}H NMR (500 MHz, CDCl$_3$) δ 7.62 – 7.32 (m, 5H), 7.00 (d, *J* = 15.7 Hz, 2H), 6.88 (d, *J* = 11.8 Hz, 1H), 4.85 (d, *J* = 81.9 Hz, 1H), 4.11 (d, *J* = 78.9 Hz, 1H), 3.16 (d, *J* = 20.8 Hz, 4H), 2.79 – 2.68 (m, 1H), δ 2.69 (dd, *J* = 161.5, 14.7 Hz, 1H), 2.39 (dd, *J* = 57.9, 14.6 Hz, 1H), 2.21 (s, 1H), 2.07 – 1.79 (m, 3H), 1.68 – 1.51 (m, 1H), 1.44 (d, *J* = 25.5 Hz, 9H).

^{13}C NMR (125 MHz, CDCl$_3$) δ 168.4, 168.1, 145.7, 145.1, 136.8, 136.3, 135.9, 135.2, 135.0, 130.0, 128.5, 128.4, 127.5, 127.1, 123.0, 122.4, 81.4, 81.3, 57.0, 57.0, 52.3, 39.2, 38.7, 37.6, 37.0, 29.5, 29.5, 28.9, 28.5, 28.1, 28.1, 27.9, 27.5, 27.1.

$[\alpha]_D^{28}$ = -137.4 (c = 0.21, CH_2Cl_2).

HRMS (ESI) ([M + Na]$^+$) Calcd. for $[C_{28}H_{31}NaNO_3]^+$: 452.2196, Found. 452.2187.

HRMS spectrum of **3ab**

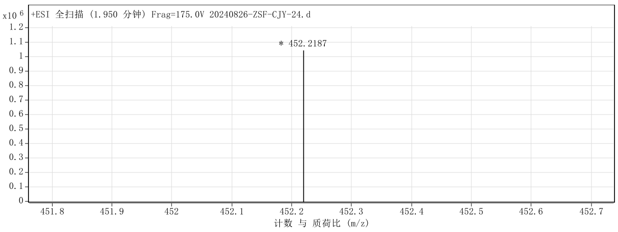

HPLC: AD-H column, hexane/^{i}PrOH = 95:5, 1.0 mL/min, t_R = 14.69 min (minor), 16.10 min (major).

Chiral HPLC spectra of (*rac*)-**3ab** and **3ab**

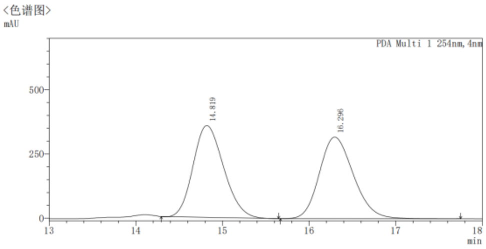

<峰表>
PDA Ch1 254nm

峰号	保留时间	面积	高度	高度%	面积%
1	14.819	8789734	357537	52.958	50.320
2	16.296	8677822	317592	47.042	49.680
总计		17467556	675129	100.000	100.000

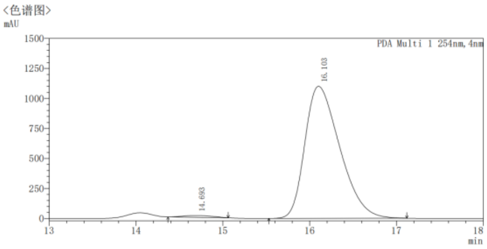

<峰表>
PDA Ch1 254nm

峰号	保留时间	面积	高度	高度%	面积%
1	14.693	351974	14738	1.322	1.131
2	16.103	30781370	1100151	98.678	98.869
总计		31133344	1114889	100.000	100.000

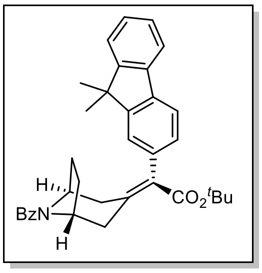

***tert*-butyl-*(Z)*-2-(*(1R,5S)*-8-benzoyl-8-azabicyclo[3.2.1]octan-3-ylidene)-2-(9,9-dimethyl-9H-fluoren-2-yl)acetate (3ac)**

39.2 mg, 76% yield, 97% ee, colorless oil, R_f = 0.62 (PE/EtOAc = 5:1).

^{1}H NMR (400 MHz, CDCl$_3$) δ 7.69 (dd, *J* = 19.6, 7.8 Hz, 2H), 7.53 (dt, *J* = 11.4, 5.5 Hz, 2H), 7.44 (dd, *J* = 14.0, 6.1 Hz, 4H), 7.34 (d, *J* = 5.3 Hz, 2H), 7.29 (s, 1H), 7.19 (dd, *J* = 17.5, 7.8 Hz, 1H), 4.90 (d, *J* = 71.6 Hz, 1H), 4.17 (d, *J* = 60.9 Hz, 1H), 2.84 (t, 1H), 2.82 (dd, *J* = 108.6, 14.8 Hz, 1H), 2.49 (t, *J* = 17.6 Hz, 1H), 2.32 (s, 1H), 2.10 – 1.88 (m, 3H), 1.79 – 1.57 (m, 1H), 1.63 – 1.39 (m, 15H).

^{13}C NMR (100 MHz, CDCl$_3$) δ 168.2, 153.7, 138.8, 138.5, 136.9, 136.5, 136.3, 135.4, 130.1, 128.5, 127.8, 127.4, 127.1, 127.0, 123.4, 123.1, 122.6, 120.0, 119.7, 81.4, 57.0, 52.3, 46.9, 39.3, 38.8, 37.7, 37.2, 28.6, 28.1, 27.2, 27.1.

$[\alpha]_D^{28}$ = -165.4 (c = 0.29, CH_2Cl_2).

HRMS (ESI) ($[M + H]^+$) Calcd. for $[C_{35}H_{38}NO_3]^+$: 520.2846, Found. 520.2847.

HRMS spectrum of **3ac**

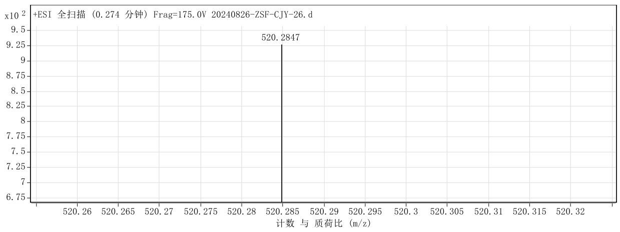

HPLC: OD-H column, hexane/^{i}PrOH = 97:3, 1.0 mL/min, t_R = 18.41 min (major), 22.98 min (minor).

Chiral HPLC spectra of (*rac*)-**3ac** and **3ac**

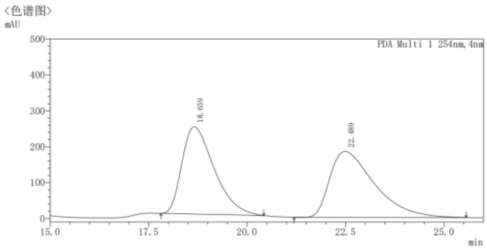

<峰表>
PDA Ch1 254nm

峰号	保留时间	面积	高度	高度%	面积%
1	18.659	13856900	243196	56.956	48.806
2	22.489	14534745	183791	43.044	51.194
总计		28391645	426986	100.000	100.000

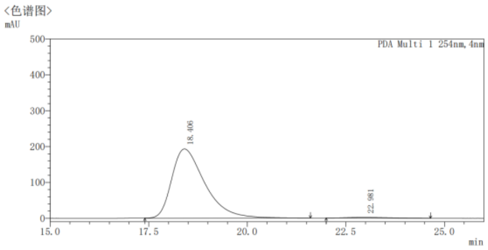

<峰表>
PDA Ch1 254nm

峰号	保留时间	面积	高度	高度%	面积%
1	18.406	11358413	193251	98.798	98.568
2	22.981	165034	2352	1.202	1.432
总计		11523447	195603	100.000	100.000

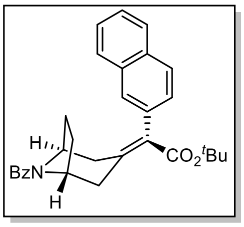

***tert*-butyl-*(Z)*-2-(*(1R,5S)*-8-benzoyl-8-azabicyclo[3.2.1]octan-3-ylidene)-2-(naphthalen-2-yl)acetate (3ad)**

30.0 mg, 66% yield, 99% ee, colorless oil, R_f = 0.61 (PE/EtOAc = 5:1).

^{1}H NMR (500 MHz, CDCl$_3$) δ 7.83 (q, *J* = 8.6 Hz, 3H), 7.69 (d, *J* = 8.3 Hz, 1H), 7.60 – 7.45 (m, 5H), 7.44 – 7.32 (m, 3H), 4.89 (d, *J* = 86.0 Hz, 1H), 4.15 (d, *J* = 83.1 Hz, 1H), 2.86 (dd, *J* = 28.2, 14.7 Hz, 1H), 2.83 (dd, *J* = 138.1, 14.7 Hz, 1H), 2.44 – 2.27 (m, 1H), 2.38 (dd, *J* = 121.9, 14.4 Hz, 1H), 1.95 (dt, *J* = 18.1, 6.7 Hz, 3H), 1.69 – 1.54 (m, 1H), 1.45 (d, *J* = 21.0 Hz, 9H).

^{13}C NMR (125 MHz, CDCl$_3$) δ 168.3, 168.1, 168.0, 137.9, 137.6, 136.3, 136.2, 135.9, 134.2, 134.1, 133.2, 132.5, 130.1, 128.5, 128.5, 127.9, 127.8, 127.7, 127.2, 127.1, 126.2, 126.2, 81.6, 81.5, 57.1, 56.9, 52.3, 52.2, 39.2, 39.0, 37.7, 37.3, 29.0, 28.7, 28.1, 27.9, 27.6, 27.2.

$[\alpha]_D^{28}$ = -170.5 (c = 0.20, CH_2Cl_2).

HRMS (ESI) ([M + Na]$^+$) Calcd. for $[C_{30}H_{31}NaNO_3]^+$: 476.2196, Found. 476.2183.

HRMS spectrum of **3ad**

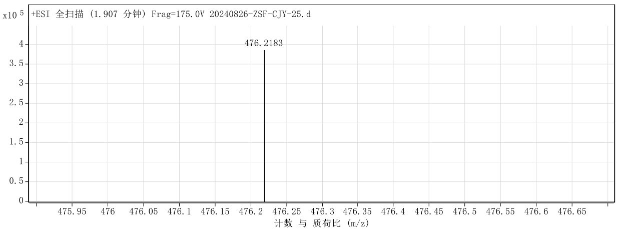

HPLC: OD-H column, hexane/^{i}PrOH = 98:2, 1.0 mL/min, t_R = 41,61 min (major), 49.72 min (minor).

Chiral HPLC spectra of (*rac*)-**3ad** and **3ad**

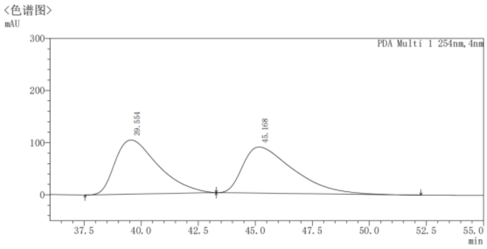

<峰表>
PDA Ch1 254nm

峰号	保留时间	面积	高度	高度%	面积%
1	39.554	13667959	103832	53.991	49.929
2	45.168	13706844	88483	46.009	50.071
总计		27374803	192315	100.000	100.000

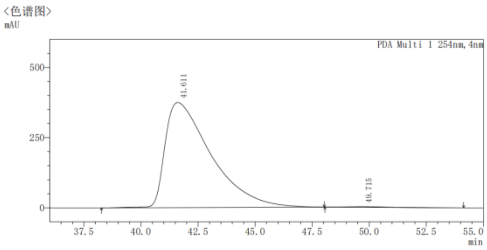

<峰表>
PDA Ch1 254nm

峰号	保留时间	面积	高度	高度%	面积%
1	41.611	52561286	374769	99.341	99.522
2	49.715	252555	2485	0.659	0.478
总计		52813841	377254	100.000	100.000

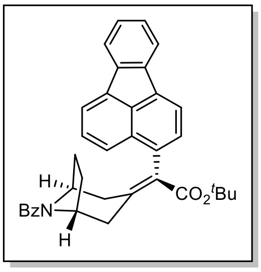

***tert*-butyl-*(Z)*-2-(*(1R,5S)*-8-benzoyl-8-azabicyclo[3.2.1]octan-3-ylidene)-2-(fluoranthen-3-yl)acetate (3ae)**

32.3 mg, 61% yield, 95% ee, colorless oil, R_f = 0.52 (PE/EtOAc = 3:1).

^{1}H NMR (500 MHz, CDCl$_3$) δ 7.90 (dt, *J* = 21.4, 9.6 Hz, 5H), 7.69 – 7.58 (m, 1H), 7.58 – 7.50 (m, 1H), 7.49 – 7.41 (m, 2H), 7.43 – 7.29 (m, 5H), 4.86 (d, *J* = 169.6 Hz, 1H), 4.40 – 3.87 (m, 1H), 3.57 – 2.99 (m, 1H), 2.55 (s, 1H), 2.47 – 2.17 (m, 1H), 2.15 – 1.80 (m, 3H), 1.65 (d, *J* = 6.5 Hz, 2H), 1.55 – 1.12 (m, 9H).

^{13}C NMR (125 MHz, $CDCl_3$) δ 168.2, 167.4, 139.6, 139.1, 137.2, 136.7, 136.3, 136.1, 135.3, 135.0, 133.5, 132.9, 132.7, 132.5, 130.1, 129.3, 129.2, 128.5, 128.4, 128.3, 128.2, 127.7, 127.2, 125.2, 124.8, 121.6, 121.5, 120.2, 119.8, 81.4, 56.9, 52.2, 39.7, 39.4, 39.1, 38.8, 38.1, 37.9, 37.5, 37.3, 28.9, 28.7, 28.0, 27.5, 27.3, 25.4.

$[\alpha]_D^{28}$ = -116.1 (c = 0.22, CH_2Cl_2).

HRMS (ESI) ($[M + H]^+$) Calcd. for $[C_{36}H_{34}NO_3]^+$: 528.2533, Found. 528.2546.

HRMS spectrum of **3ae**

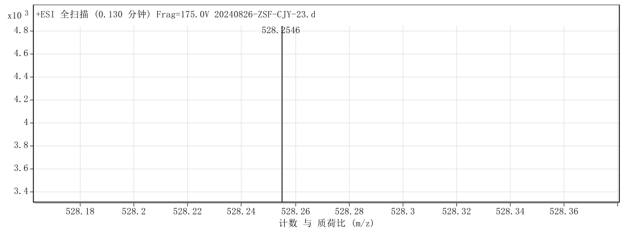

HPLC: AD-H column, hexane/^{i}PrOH = 95:5, 1.0 mL/min, t_R = 52.55 min (major), 65.99 min (minor).

Chiral HPLC spectra of (*rac*)-**3ae** and **3ae**

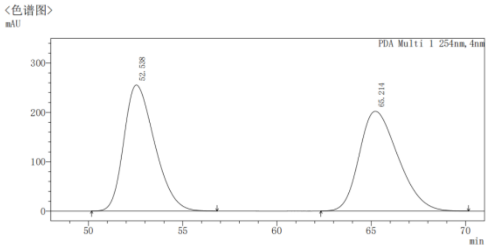

<峰表>
PDA Ch1 254nm

峰号	保留时间	面积	高度	高度%	面积%
1	52.538	29048656	255388	55.808	50.003
2	65.214	29044971	202231	44.192	49.997
总计		58093627	457620	100.000	100.000

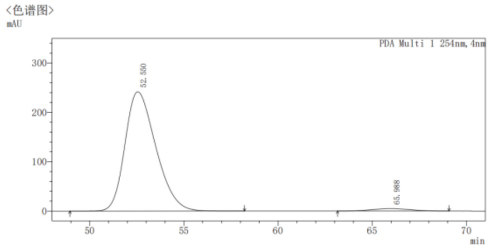

<峰表>
PDA Ch1 254nm

峰号	保留时间	面积	高度	高度%	面积%
1	52.550	27545771	241677	98.076	97.630
2	65.988	668730	4742	1.924	2.370
总计		28214501	246419	100.000	100.000

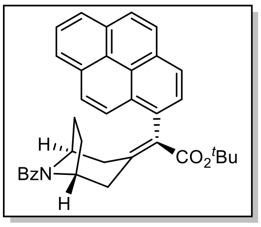

***tert*-butyl-*(Z)*-2-(*(1R,5S)*-8-benzoyl-8-azabicyclo[3.2.1]octan-3-ylidene)-2-(pyren-1-yl)acetate (3af)**

46,4 mg, 88% yield, 98% ee, green oil, R_f = 0.48 (PE/EtOAc = 3:1).

^{1}H NMR (500 MHz, $CDCl_3$) δ 8.37 – 7.89 (m, 9H), 7.78 – 7.39 (m, 4H), 7.35 – 7.26 (m, 1H), 4.69 (d, *J* = 370.8 Hz, 1H), 4.32 (dd, 1H), 3.65 – 3.10 (m, 1H), 3.05 – 2.68 (m, 1H), 2.67 – 2.39 (m, 1H), 2.33 – 2.02 (m, 2H), 1.99 – 1.69 (m, 3H), 1.61 – 0.83 (m, 9H).

^{13}C NMR (125 MHz, $CDCl_3$) δ 168.2, 167.7, 167.5, 142.8, 142.3, 140.8, 136.3, 134.7, 134.1, 132.6, 132.4, 131.3, 131.0, 130.8, 130.8, 130.1, 130.1, 129.3, 128.5, 128.4, 127.9, 127.8, 127.6, 127.5, 127.4,

127.3, 127.2, 127.1, 126.7, 126.1, 126.1, 125.3, 125.3, 125.2, 124.9, 124.7, 124.6, 56.9, 56.8, 52.3, 52.1, 39.9, 39.2, 39.0, 38.8, 38.3, 37.6, 37.3, 28.8, 28.1, 27.9, 27.6, 27.4.

$[\alpha]_D^{28}$ = -43.1 (c = 0.36, CH_2Cl_2).

HRMS (ESI) ($[M + Na]^+$) Calcd. for $[C_{36}H_{33}NaNO_3]^+$: 550.2352, Found. 550.2356.

HRMS spectrum of **3af**

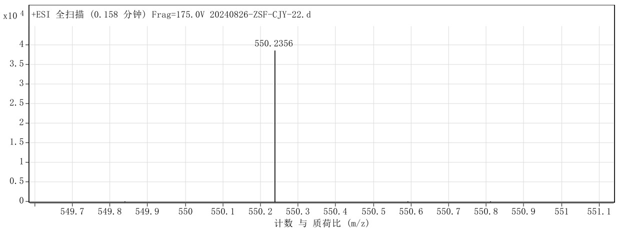

HPLC: INA column, hexane/^{i}PrOH = 90:10, 1.0 mL/min, t_R = 10.71 min (minor), 12.75 min (major).

Chiral HPLC spectra of (*rac*)-**3af** and **3af**

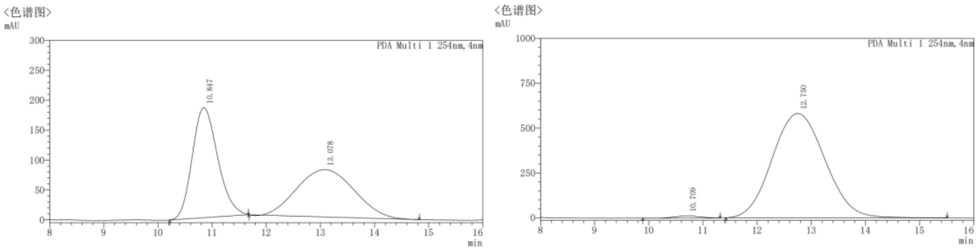

<峰表>
PDA Ch1 254nm

峰号	保留时间	面积	高度	高度%	面积%
1	10.847	6022006	184137	69.931	50.783
2	13.078	5836349	79177	30.069	49.217
总计		11858355	263314	100.000	100.000

<峰表>
PDA Ch1 254nm

峰号	保留时间	面积	高度	高度%	面积%
1	10.709	419031	12442	2.097	1.053
2	12.750	39365917	580850	97.903	98.947
总计		39784948	593292	100.000	100.000

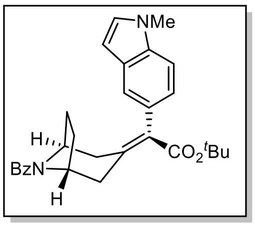

***tert*-butyl-*(Z)*-2-(*(1R,5S)*-8-benzoyl-8-azabicyclo[3.2.1]octan-3-ylidene)-2-(1-methyl-1H-indol-5-yl)acetate (3ag)**

20.1 mg, 44% yield, 99% ee, colorless oil, R_f = 0.46 (PE/EtOAc = 3:1).

^{1}H NMR (500 MHz, CDCl$_3$) δ 7.40 (s, 2H), 7.37 (s, 2H), 7.30 (d, J = 7.6 Hz, 1H), 7.19 (d, J = 1.1 Hz, 2H), 7.05 – 6.92 (m, 2H), 6.39 (d, J = 18.7 Hz, 1H), 4.77 (d, J = 102.3 Hz, 1H), 4.13 (d, 1H), 3.72 (d, J = 15.6 Hz, 3H), 2.68 (t, 1H), 2.64 (dd, J = 144.0, 14.6 Hz, 1H), 2.37 (dd, J = 45.8, 14.6 Hz, 1H), 2.19 (s, 1H), 1.98 – 1.75 (m, 3H), 1.37 (d, J = 25.6 Hz, 9H), 1.18 (s, 1H).

^{13}C NMR (125 MHz, CDCl$_3$) δ 167.9, 167.1, 135.9, 134.9, 134.0, 128.9, 128.3, 127.4, 127.3, 126.1, 125.8, 121.7, 120.2, 108.0, 100.1, 80.2, 80.1, 56.1, 56.0, 51.3, 38.2, 37.7, 36.7, 36.0, 31.9, 28.7, 27.9, 27.5, 27.1, 26.5, 26.1.

[α]$_D^{28}$ = -113.2 (c = 0.11, CH_2Cl_2).

HRMS (ESI) ([M + Na]$^+$) Calcd. for $[C_{29}H_{32}NaN_2O_3]^+$: 479.2305, Found. 479.2302.

HRMS spectrum of **3ag**

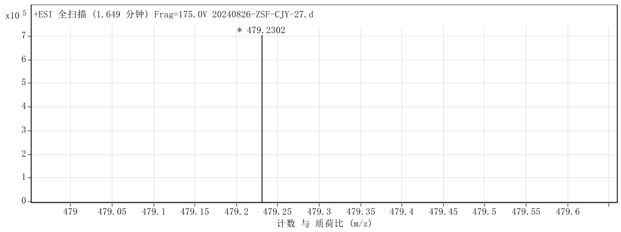

HPLC: AD-H column, hexane/^{i}PrOH = 92:8, 1.0 mL/min, t_R = 30.08 min (major), 31.71 min (minor).

Chiral HPLC spectra of (*rac*)-**3ag** and **3ag**

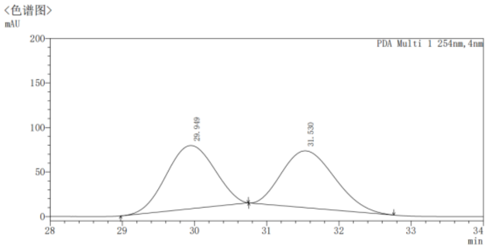

<峰表>
PDA Ch1 254nm

峰号	保留时间	面积	高度	高度%	面积%
1	29.949	3302231	71028	52.677	50.366
2	31.530	3254224	63810	47.323	49.634
总计		6556455	134838	100.000	100.000

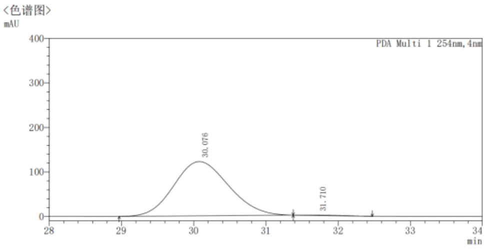

<峰表>
PDA Ch1 254nm

峰号	保留时间	面积	高度	高度%	面积%
1	30.076	6437436	121917	99.367	99.544
2	31.710	29490	776	0.633	0.456
总计		6466926	122694	100.000	100.000

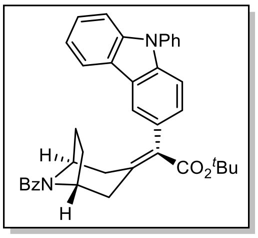

***tert*-butyl-*(Z)*-2-(*(1R,5S)*-8-benzoyl-8-azabicyclo[3.2.1]octan-3-ylidene)-2-(9-phenyl-9H-carbazol-3-yl)acetate (3ah)**

41.7 mg, 73% yield, 96% ee, colorless oil, R_f = 0.32 (PE/EtOAc = 5:1).

^{1}H NMR (500 MHz, $CDCl_3$) δ 8.15 (t, *J* = 6.1 Hz, 1H), 8.01 (d, *J* = 9.3 Hz, 1H), 7.72 – 7.55 (m, 5H), 7.54 – 7.37 (m, 8H), 7.34 – 7.29 (m, 2H), 4.91 (d, *J* = 82.3 Hz, 1H), 4.16 (d, *J* = 83.3 Hz, 1H), 2.84 (d, *J* = 15.2 Hz, 1H), 2.81 (dd, *J* = 110.6 Hz, 1H), 2.52 (dd, *J* = 30.4, 14.6 Hz, 1H), 2.34 (s, 1H), 2.00 (q, *J* = 16.5, 15.4 Hz, 3H), 1.67 (d, *J* = 10.8 Hz, 1H), 1.49 (d, *J* = 19.8 Hz, 9H).

^{13}C NMR (125 MHz, $CDCl_3$) δ 168.7, 168.2, 141.2, 140.1, 137.6, 136.5, 136.1, 130.0, 129.9, 128.5, 128.4, 127.6, 127.1, 126.2, 123.3, 123.2, 120.7, 120.3, 120.1, 109.9, 109.6, 81.3, 57.1, 57.0, 52.4, 39.3, 38.9, 38.3, 37.8, 37.2, 37.0, 29.0, 28.6, 28.2, 27.6, 27.2.

[α]$_D^{28}$ = -126.2 (c = 0.32, CH_2Cl_2).

HRMS (ESI) ([M + Na]$^+$) Calcd. for $[C_{38}H_{36}NaN_2O_3]^+$: 591.2618, Found. 591.2623.

HRMS spectrum of **3ah**

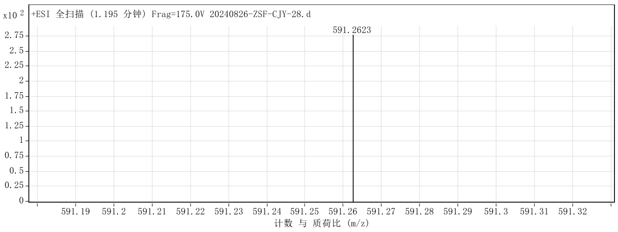

HPLC: AD-H column, hexane/^{i}PrOH = 93:7, 0.8 mL/min, t_R = 22.04 min (minor), 27.01 min (major).

Chiral HPLC spectra of (*rac*)-**3ah** and **3ah**

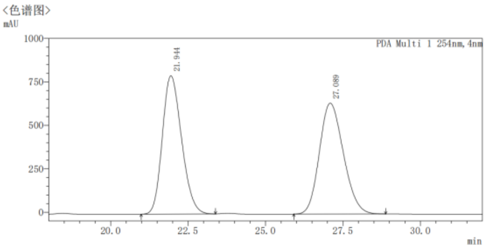

<峰表>
PDA Ch1 254nm

峰号	保留时间	面积	高度	高度%	面积%
1	21.944	34430100	795910	55.511	49.988
2	27.089	34446381	637888	44.489	50.012
总计		68876480	1433798	100.000	100.000

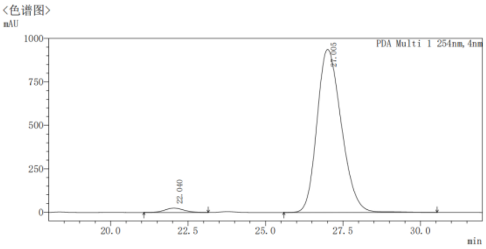

<峰表>
PDA Ch1 254nm

峰号	保留时间	面积	高度	高度%	面积%
1	22.040	1047832	24490	2.545	2.002
2	27.005	51296278	937795	97.455	97.998
总计		52344110	962285	100.000	100.000

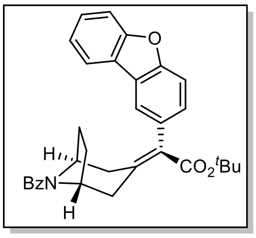

***tert*-butyl-*(Z)*-2-(*(1R,5S)*-8-benzoyl-8-azabicyclo[3.2.1]octan-3-ylidene)-2-(dibenzo[b,d]furan-2-yl)acetate (3ai)**

29.0 mg, 59% yield, 98% ee, colorless oil, R_f = 0.47 (PE/EtOAc = 3:1).

^{1}H NMR (500 MHz, CDCl$_3$) δ 7.99 – 7.85 (m, 2H), 7.65 – 7.31 (m, 9H), 7.19 (d, *J* = 7.9 Hz, 1H), 4.88 (d, *J* = 96.1 Hz, 1H), 4.14 (d, *J* = 97.3 Hz, 1H), 2.93 (dd, *J* = 54.3, 14.4 Hz, 1H), 2.71 (dd, *J* = 90.7, 14.8 Hz, 1H), 2.37 (dd, *J* = 145.1, 14.7 Hz, 1H), 2.42 – 2.27 (m, 1H), 2.12 – 1.84 (m, 3H), 1.67 – 1.56 (m, 1H), 1.44 (d, *J* = 25.5 Hz, 9H).

^{13}C NMR (125 MHz, CDCl$_3$) δ 168.2, 167.8, 156.5, 156.1, 136.3, 135.8, 130.1, 128.5, 127.3, 127.1, 123.9, 123.5, 122.9, 120.6, 120.4, 112.2, 111.7, 81.7, 81.6, 57.0, 52.3, 39.2, 39.0, 37.6, 37.3, 29.0, 28.7, 28.1, 27.6, 27.2.

$[\alpha]_D^{28}$ = -153.8 (c = 0.29, CH_2Cl_2).

HRMS (ESI) ($[M + Na]^+$) Calcd. for $[C_{32}H_{31}NaNO_4]^+$: 516.2145, Found. 516.2142.

HRMS spectrum of **3ai**

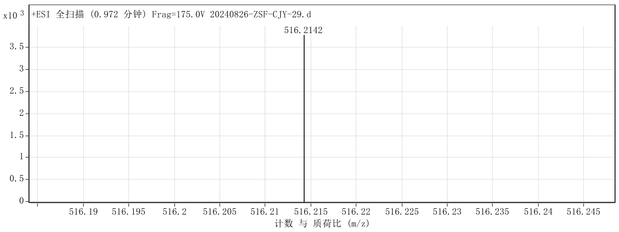

HPLC: AD-H column, hexane/^{i}PrOH = 90:10, 1.0 mL/min, t_R = 19.86 min (major), 22.38 min (minor).

Chiral HPLC spectra of (*rac*)-**3ai** and **3ai**

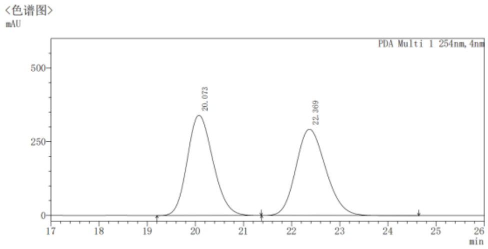

<峰表>
PDA Ch1 254nm

峰号	保留时间	面积	高度	高度%	面积%
1	20.073	12667880	340252	53.726	50.112
2	22.369	12611193	293053	46.274	49.888
总计		25279073	633305	100.000	100.000

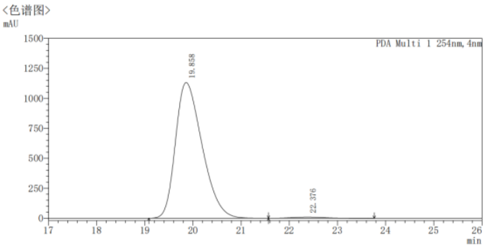

<峰表>
PDA Ch1 254nm

峰号	保留时间	面积	高度	高度%	面积%
1	19.858	43472373	1131531	99.182	99.106
2	22.376	392230	9334	0.818	0.894
总计		43864603	1140865	100.000	100.000

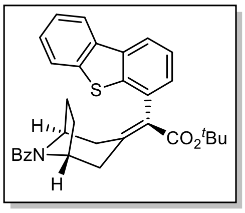

***tert*-butyl-*(Z)*-2-(*(1R,5S)*-8-benzoyl-8-azabicyclo[3.2.1]octan-3-ylidene)-2-(dibenzo[b,d]thiophen-4-yl)acetate (3aj)**

48.4 mg, 95% yield, 99% ee, colorless oil, R_f = 0.45 (PE/EtOAc = 3:1).

^{1}H NMR (500 MHz, $CDCl_3$) δ 8.31 – 8.06 (m, 2H), 7.93 – 7.82 (m, 1H), 7.63 – 7.31 (m, 9H), 4.86 (d, *J* = 133.6 Hz, 1H), 4.43 – 3.94 (m, 1H), 3.58 – 3.09 (m, 1H), 2.58 (ddd, *J* = 214.5, 29.4, 15.2 Hz, 1H), 2.71 – 2.50 (m, 1H), 2.21 – 1.80 (m, 5H), 1.55 – 1.14 (m, 9H).

^{13}C NMR (125 MHz, $CDCl_3$) δ 168.1, 166.7, 166.2, 139.4, 136.3, 135.8, 133.8, 132.8, 132.4, 132.3, 130.1, 128.5, 128.4, 127.8, 127.2, 126.8, 124.7, 124.5, 122.8, 121.8, 120.6, 81.6, 81.4, 56.6, 52.3, 52.0, 39.9, 38.9, 38.6, 37.4, 29.7, 29.0, 28.9, 28.7, 28.0, 27.9, 27.9, 27.6, 27.6, 27.3, 27.2.

$[\alpha]_D^{28}$ = -53.7 (c = 0.53, CH_2Cl_2).

HRMS (ESI) ($[M + Na]^+$) Calcd. for $[C_{32}H_{31}NaNO_3S]^+$: 532.1917, Found. 532.1911.

HRMS spectrum of **3aj**

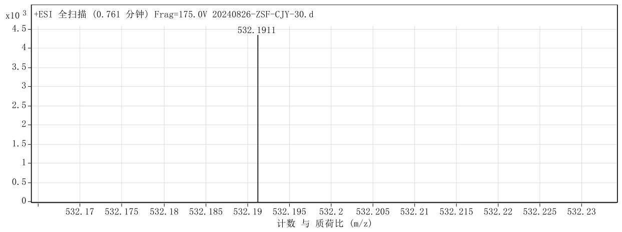

HPLC: INA column, hexane/^{i}PrOH = 97:3, 1.0 mL/min, t_R = 33.28 min (minor), 42.75 min (major).

Chiral HPLC spectra of (*rac*)-**3aj** and **3aj**

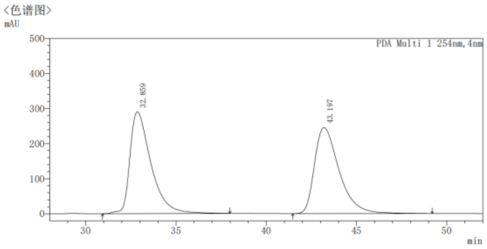

<峰表>
PDA Ch1 254nm

峰号	保留时间	面积	高度	高度%	面积%
1	32.859	23555102	290212	54.195	50.200
2	43.197	23367042	245288	45.805	49.800
总计		46922143	535501	100.000	100.000

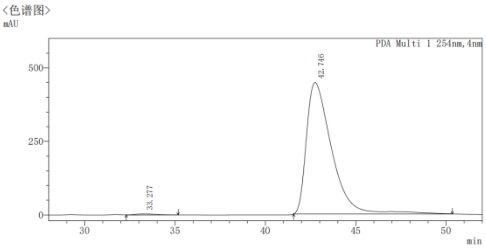

<峰表>
PDA Ch1 254nm

峰号	保留时间	面积	高度	高度%	面积%
1	33.277	274975	3526	0.783	0.629
2	42.746	43415356	446529	99.217	99.371
总计		43690330	450055	100.000	100.000

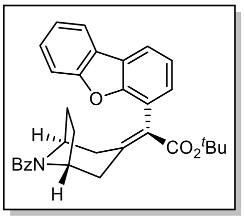

***tert*-butyl-*(Z)*-2-(*(1R,5S)*-8-benzoyl-8-azabicyclo[3.2.1]octan-3-ylidene)-2-(dibenzo[b,d]furan-4-yl)acetate (3ak)**

46.8 mg, 74% yield, 98% ee, colorless oil, R_f = 0.47 (PE/EtOAc = 3:1).

^{1}H NMR (500 MHz, CDCl$_3$) δ 7.92 (ddd, *J* = 30.1, 18.5, 7.8 Hz, 2H), 7.58 (d, *J* = 8.2 Hz, 1H), 7.53 (d, 1H), 7.46 (d, *J* = 6.6 Hz, 3H), 7.37 – 7.31 (m, 3H), 7.30 – 7.27 (m, 2H), 4.88 (d, *J* = 135.2 Hz, 1H), 4.14 (d, *J* = 130.4 Hz, 1H), 3.34 (dd, *J* = 45.3, 14.7 Hz, 1H), 2.77 (dd, *J* = 86.2, 14.9 Hz, 1H), 2.46 (dd, *J* = 123.3, 14.6 Hz, 1H), 2.26 – 2.06 (m, 1H), 2.09 – 1.98 (m, 2H), 1.87 (d, *J* = 17.0 Hz, 1H), 1.71 (dt, *J* = 7.9, 4.4 Hz, 1H), 1.37 (d, *J* = 32.1 Hz, 9H).

^{13}C NMR (125 MHz, CDCl$_3$) δ 168.2, 167.0, 156.1, 156.1, 153.6, 143.4, 143.1, 142.2, 136.2, 136.1, 130.1, 130.0, 128.5, 128.4, 128.0, 127.3, 127.3, 127.2, 127.1, 127.0, 126.8, 125.7, 124.7, 124.3, 124.2, 123.5, 122.8, 122.7, 122.7, 121.8, 120.9, 120.7, 120.1, 113.8, 112.0, 111.8, 81.5, 81.3, 57.0, 56.9, 52.3, 52.2, 39.8, 39.0, 38.3, 37.5, 29.7, 28.8, 28.1, 28.0, 27.9, 27.4.

$[\alpha]_D^{28}$ = -21.5 (c = 0.37, CH_2Cl_2).

HRMS (ESI) ([M + Na]$^+$) Calcd. for [$C_{32}H_{31}NaNO_4$]$^+$: 516.2145, Found. 516.2145.

HRMS spectrum of **3ak**

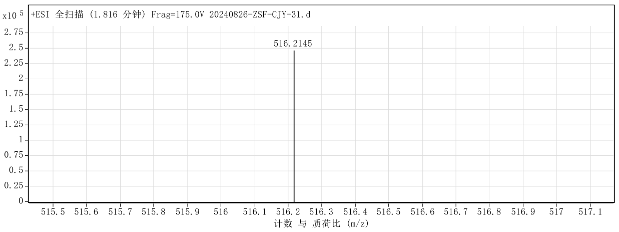

HPLC: AD-H column, hexane/^{i}PrOH = 90:10, 1.0 mL/min, t_R = 9.29 min (minor), 12.41 min (major).

Chiral HPLC spectra of (*rac*)-**3ak** and **3ak**

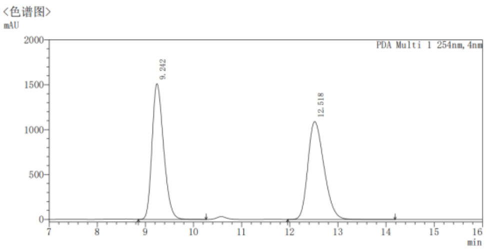

<峰表>
PDA Ch1 254nm

峰号	保留时间	面积	高度	高度%	面积%
1	9.242	25274529	1512159	58.117	49.926
2	12.518	25349841	1089778	41.883	50.074
总计		50624370	2601937	100.000	100.000

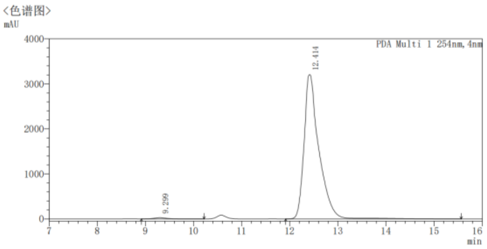

<峰表>
PDA Ch1 254nm

峰号	保留时间	面积	高度	高度%	面积%
1	9.299	495295	28448	0.879	0.713
2	12.414	68977984	3208249	99.121	99.287
总计		69473279	3236697	100.000	100.000

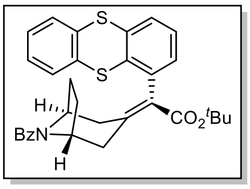

***tert*-butyl-*(Z)*-2-(*(1R,5S)*-8-benzoyl-8-azabicyclo[3.2.1]octan-3-ylidene)-2-(thianthren-1-yl)acetate (3al)**

52.0 mg, 97% yield, 99% ee, colorless oil, R_f = 0.43 (PE/EtOAc = 3:1).

^{1}H NMR (500 MHz, $CDCl_3$) δ 7.71 – 7.33 (m, 8H), 7.29 – 6.88 (m, 4H), 5.18 – 4.63 (m, 1H), 4.14 (d, *J* = 116.4 Hz, 1H), 3.58 (ddd, *J* = 164.5, 35.9, 15.2 Hz, 1H), 3.03 – 2.42 (m, 1H), 2.30 – 1.99 (m, 2H), 2.03 – 1.53 (m, 4H), 1.35 (dd, *J* = 25.7, 9.5 Hz, 9H).

^{13}C NMR (125 MHz, $CDCl_3$) δ 168.3, 168.1, 168.1, 166.1, 165.5, 147.0, 146.5, 144.5, 144.3, 138.5, 138.4, 136.2, 136.1, 136.0, 135.6, 135.4, 133.1, 130.1, 129.2, 128.8, 128.6, 128.5, 128.4, 128.1, 127.9, 127.8, 127.8, 127.7, 127.6, 127.2, 127.2, 127.1, 81.3, 81.1, 57.5, 56.9, 56.7, 56.5, 52.3, 52.1, 51.8, 40.8, 39.2, 38.6, 38.4, 37.7, 37.1, 36.9, 29.7, 29.2, 28.9, 28.0, 28.0, 27.9, 27.4.

$[\alpha]_D^{28}$ = -54.8 (c = 0.42, CH_2Cl_2).

HRMS (ESI) ($[M + Na]^+$) Calcd. for $[C_{32}H_{31}NaNO_3S_2]^+$: 564.1637, Found. 564.1637.

HRMS spectrum of **3al**

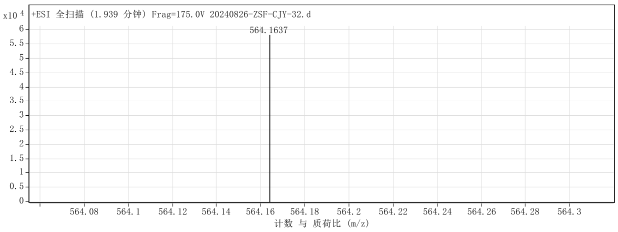

HPLC: INA column, hexane/^{i}PrOH = 97:3, 1.0 mL/min, t_R = 34.85 min (major), 42.49 min (minor).

Chiral HPLC spectra of (*rac*)-**3al** and **3al**

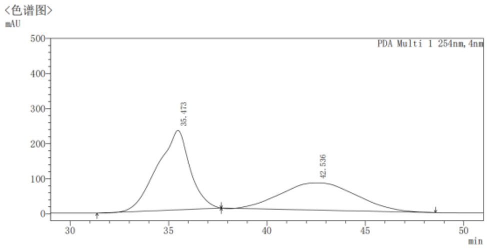

<峰表>
PDA Ch1 254nm

峰号	保留时间	面积	高度	高度%	面积%
1	35.473	26224104	226290	74.474	55.927
2	42.536	20666179	77561	25.526	44.073
总计		46890283	303851	100.000	100.000

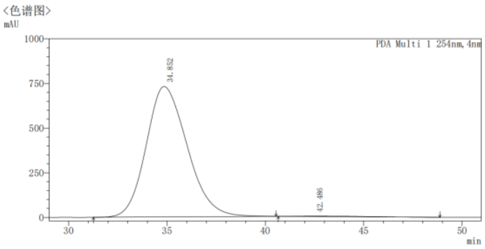

<峰表>
PDA Ch1 254nm

峰号	保留时间	面积	高度	高度%	面积%
1	34.852	106213404	730430	99.674	99.466
2	42.486	569916	2391	0.326	0.534
总计		106783321	732820	100.000	100.000

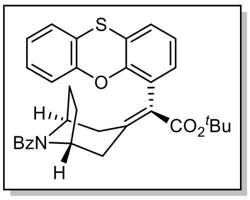

***tert*-butyl-*(Z)*-2-(*(1R,5S)*-8-benzoyl-8-azabicyclo[3.2.1]octan-3-ylidene)-2-(phenoxathiin-4-yl)acetate (3am)**

44.7 mg, 85% yield, 98% ee, colorless oil, R_f = 0.44 (PE/EtOAc = 3:1).

^{1}H NMR (500 MHz, $CDCl_3$) δ 7.64 – 7.32 (m, 5H), 7.18 – 6.81 (m, 7H), 4.89 (d, *J* = 106.2 Hz, 1H), 4.15 (d, *J* = 103.1 Hz, 1H), 3.75 – 3.28 (m, 1H), 3.21 – 2.33 (m, 2H), 2.24 (t, *J* = 17.3 Hz, 1H), 2.15 – 1.77 (m, 4H), 1.33 (d, *J* = 27.4 Hz, 9H).

^{13}C NMR (125 MHz, $CDCl_3$) δ 168.1, 166.3, 152.1, 149.4, 144.3, 136.2, 130.1, 129.3, 128.4, 127.7, 127.2, 127.1, 126.7, 126.2, 124.7, 124.0, 120.6, 120.4, 117.9, 81.1, 81.0, 72.8, 57.0, 52.3, 38.5, 36.9, 28.7, 28.1, 27.9, 27.5, 27.3.

$[\alpha]_D^{28}$ = -18.6 (c = 0.43, CH_2Cl_2).

HRMS (ESI) ($[M + Na]^+$) Calcd. for $[C_{32}H_{31}NaNO_4S]^+$: 548.1866, Found. 548.1866.

HRMS spectrum of **3am**

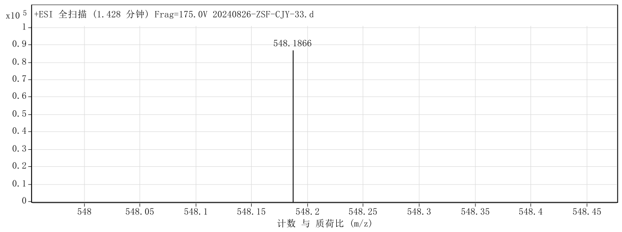

HPLC: AD-H column, hexane/^{i}PrOH = 90:10, 1.0 mL/min, t_R = 7.90 min (minor), 9.01 min (major).

Chiral HPLC spectra of (*rac*)-**3am** and **3am**

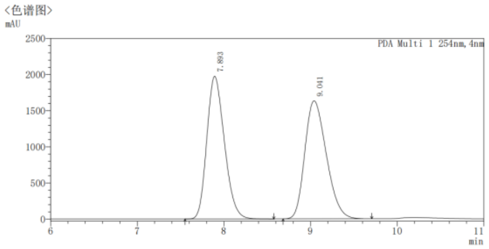

<峰表>
PDA Ch1 254nm

峰号	保留时间	面积	高度	高度%	面积%
1	7.893	27886247	1978498	54.772	49.704
2	9.041	28217881	1633744	45.228	50.296
总计		56104128	3612242	100.000	100.000

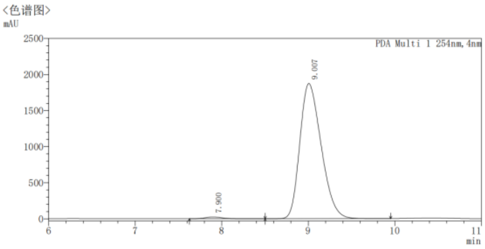

<峰表>
PDA Ch1 254nm

峰号	保留时间	面积	高度	高度%	面积%
1	7.900	340162	24638	1.298	1.047
2	9.007	32140205	1873777	98.702	98.953
总计		32480367	1898415	100.000	100.000

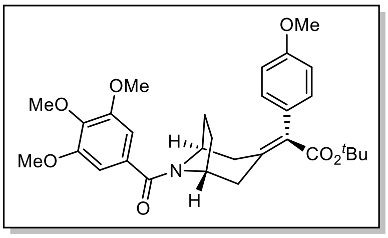

***tert*-butyl-*(Z)*-2-(4-methoxyphenyl)-2-(*(1R,5S)*-8-(3,4,5-trimethoxybenzoyl)-8-azabicyclo[3.2.1]octan-3-ylidene)acetate (3an)**

31.9 mg, 61% yield, 98% ee, colorless oil, R_f = 0.51 (PE/EtOAc = 1:1).

^{1}H NMR (500 MHz, $CDCl_3$) δ 7.12 (d, *J* = 8.0 Hz, 2H), 6.87 (dt, *J* = 14.1, 5.9 Hz, 2H), 6.79 – 6.66 (m, 2H), 4.82 (d, *J* = 76.8 Hz, 1H), 4.28 (d, 1H), 4.04 – 3.70 (m, 9H), 3.84 – 3.71 (m, 3H), 2.79 (dq, *J* = 23.7, 14.7 Hz, 1H), 2.51 (dd, *J* = 37.5, 14.6 Hz, 1H), 2.31 (d, *J* = 57.2 Hz, 1H), 1.91 (dd, *J* = 25.4, 13.3 Hz, 3H), 1.77 (s, 1H), 1.59 (t, *J* = 8.6 Hz, 1H), 1.56 – 1.25 (m, 9H).

^{13}C NMR (125 MHz, $CDCl_3$) δ 168.3, 168.0, 158.9, 153.2, 150.2, 139.6, 135.7, 131.6, 130.1, 128.8, 116.0, 114.7, 113.7, 104.6, 81.4, 60.9, 57.2, 56.3, 55.8, 55.2, 52.4, 39.2, 38.5, 37.6, 37.0, 28.6, 28.1, 27.9, 27.5, 27.1.

$[\alpha]_D^{28}$ = -104.6 (c = 0.22, CH_2Cl_2).

HRMS (ESI) ($[M + Na]^+$) Calcd. for $[C_{30}H_{37}NaNO_7]^+$: 546.2462, Found. 546.2465.

HRMS spectrum of **3an**

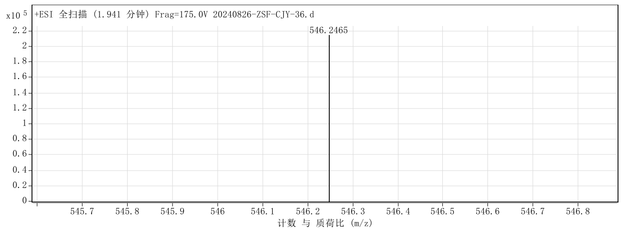

HPLC: AD-H column, hexane/^{i}PrOH = 90:10, 1.0 mL/min, t_R = 31.34 min (minor), 34.28 min (major).

Chiral HPLC spectra of (*rac*)-**3an** and **3an**

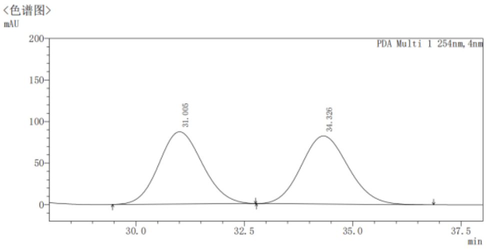

<峰表>
PDA Ch1 254nm

峰号	保留时间	面积	高度	高度%	面积%
1	31.005	6096959	86960	51.491	49.631
2	34.326	6187735	81924	48.509	50.369
总计		12284694	168884	100.000	100.000

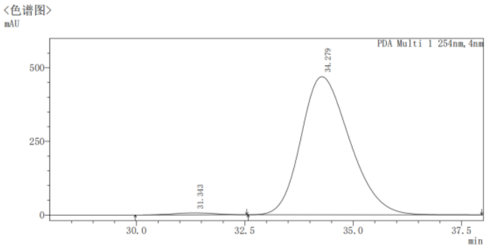

<峰表>
PDA Ch1 254nm

峰号	保留时间	面积	高度	高度%	面积%
1	31.343	432769	6556	1.377	1.164
2	34.279	36736939	469529	98.623	98.836
总计		37169708	476085	100.000	100.000

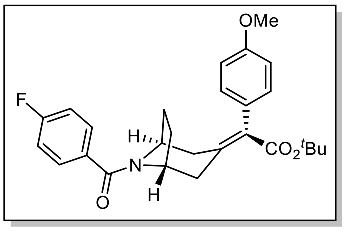

***tert*-butyl-*(Z)*-2-(*(1R,5S)*-8-(4-fluorobenzoyl)-8-azabicyclo[3.2.1]octan-3-ylidene)-2-(4-methoxyphenyl)acetate (3ao)**

12.1 mg, 27% yield, 97% ee, colorless oil, R_f = 0.55 (PE/EtOAc = 3:1).

^{1}H NMR (500 MHz, CDCl$_3$) δ 7.51 (s, 2H), 7.10 (s, 4H), 6.81 (d, *J* = 51.7 Hz, 2H), 4.82 (d, *J* = 79.2 Hz, 1H), 4.11 (d, *J* = 75.5 Hz, 1H), 3.80 (d, *J* = 10.1 Hz, 3H), 2.74 (t, *J* = 17.9 Hz, 1H), 2.69 (dd, *J* = 155.5, 14.7 Hz, 1H), 2.38 (dd, *J* = 46.5, 14.5 Hz, 1H), 2.22 (s, 1H), 2.00 – 1.82 (m, 3H), 1.67 – 1.50 (m, 1H), 1.50 – 1.32 (m, 9H).

^{13}C NMR (125 MHz, CDCl$_3$) δ 167.3, 166.2, 163.7, 161.7, 157.9, 135.0, 134.97 (d, *J* = 18.2 Hz), 131.26 (d, *J* = 2.7 Hz), 129.1, 128.5, 128.4, 115.0, 114.50 (d, *J* = 21.8 Hz), 113.8, 113.7, 112.7, 80.4, 56.1, 54.8, 54.2, 51.4, 38.1, 37.7, 36.5, 35.9, 27.5, 27.1, 26.5, 26.1.

^{19}F NMR (376 MHz, CDCl$_3$) δ -109.9.

$[\alpha]_D^{28}$ = -120.7 (c = 0.12, CH_2Cl_2).

HRMS (ESI) ([M + Na]$^+$) Calcd. for $[C_{27}H_{30}NaFNO_4]^+$: 474.2051, Found. 474.2060.

HRMS spectrum of **3ao**

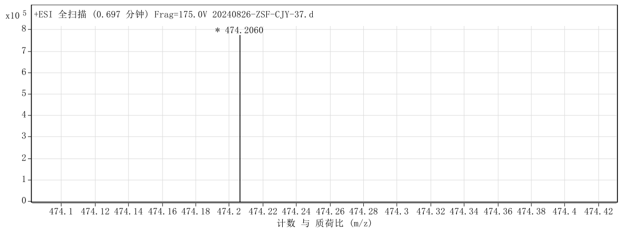

HPLC: INC column, hexane/^{i}PrOH = 90:10, 1.0 mL/min, t_R = 24.19 min (minor), 28.85 min (major).

Chiral HPLC spectra of (*rac*)-**3ao** and **3ao**

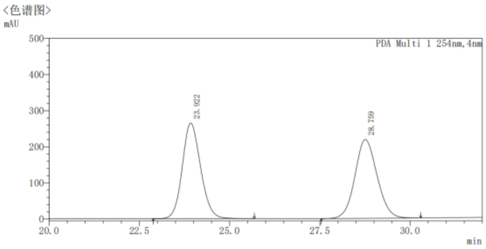

<峰表>
PDA Ch1 254nm

峰号	保留时间	面积	高度	高度%	面积%
1	23.922	9347950	264674	54.823	50.133
2	28.759	9298382	218106	45.177	49.867
总计		18646332	482780	100.000	100.000

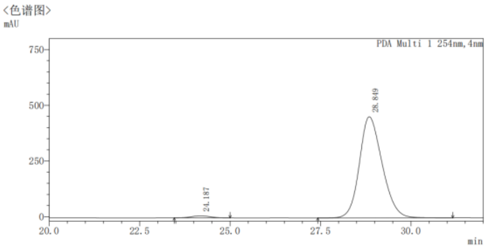

<峰表>
PDA Ch1 254nm

峰号	保留时间	面积	高度	高度%	面积%
1	24.187	330719	9478	2.044	1.639
2	28.849	19843325	454199	97.956	98.361
总计		20174044	463677	100.000	100.000

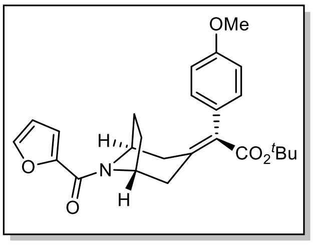

***tert*-butyl-*(Z)*-2-(*(1R,5S)*-8-(furan-2-carbonyl)-8-azabicyclo[3.2.1]octan-3-ylidene)-2-(4-methoxyphenyl)acetate (3ap)**

23.6 mg, 56% yield, 98% ee, colorless oil, R_f = 0.57 (PE/EtOAc = 3:1).

^{1}H NMR (400 MHz, $CDCl_3$) δ 7.49 (d, *J* = 18.6 Hz, 1H), 7.14 (dd, *J* = 9.8, 3.0 Hz, 3H), 6.97 – 6.81 (m, 2H), 6.50 (s, 1H), 4.94 (dd, *J* = 64.0, 39.8 Hz, 2H), 4.00 – 3.62 (m, 3H), 2.98 – 2.82 (m, 1H), 2.65 (d, *J* = 18.8 Hz, 1H), 2.42 (d, *J* = 28.8 Hz, 2H), 1.94 (d, 3H), 1.68 – 1.54 (m, 1H), 1.46 (s, 9H).

^{13}C NMR (125 MHz, $CDCl_3$) δ 167.4, 157.8, 154.9, 147.7, 142.7, 135.4, 134.4, 129.5, 129.1, 128.0, 115.3, 115.2, 112.7, 112.5, 110.4, 80.3, 54.2, 54.2, 52.2, 38.6, 38.2, 36.9, 36.3, 28.3, 28.0, 27.1, 26.9, 25.7, 25.3.

$[\alpha]_D^{28}$ = -143.0 (c = 0.19, CH_2Cl_2).

HRMS (ESI) ($[M + Na]^+$) Calcd. for $[C_{25}H_{29}NaNO_5]^+$: 446.1938, Found. 446.1941.

HRMS spectrum of **3ap**

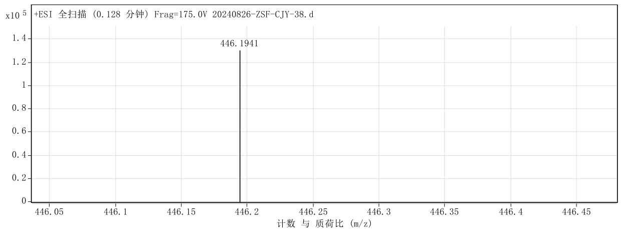

HPLC: OD-H column, hexane/^{i}PrOH = 97:3, 1.0 mL/min, t_R = 25.82 min (major), 31.10 min (minor).

Chiral HPLC spectra of (*rac*)-**3ap** and **3ap**

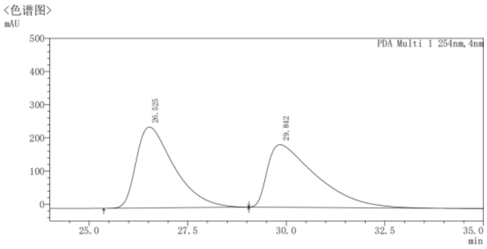

<峰表>
PDA Ch1 254nm

峰号	保留时间	面积	高度	高度%	面积%
1	26.525	16317418	243912	56.360	50.041
2	29.842	16290367	188860	43.640	49.959
总计		32607785	432773	100.000	100.000

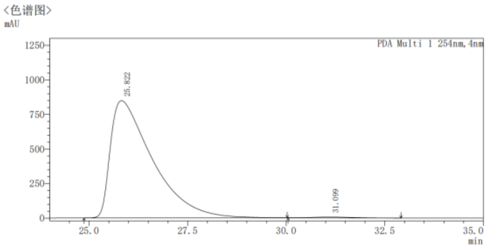

<峰表>
PDA Ch1 254nm

峰号	保留时间	面积	高度	高度%	面积%
1	25.822	63020363	848161	99.328	99.354
2	31.099	409746	5740	0.672	0.646
总计		63430109	853901	100.000	100.000

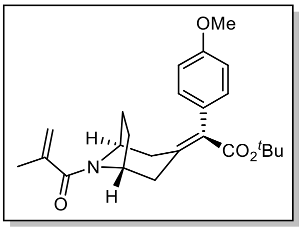

***tert*-butyl-*(Z)*-2-(*(1R,5S)*-8-methacryloyl-8-azabicyclo[3.2.1]octan-3-ylidene)-2-(4-methoxyphenyl)acetate (3aq)**

23.6 mg, 59% yield, 85% ee, colorless oil, R_f = 0.52 (PE/EtOAc = 3:1).

^{1}H NMR (500 MHz, $CDCl_3$) δ 7.11 (d, *J* = 8.2 Hz, 2H), 6.86 (d, *J* = 8.7 Hz, 2H), 5.16 (dd, *J* = 36.7, 23.0 Hz, 2H), 4.72 (d, *J* = 82.4 Hz, 1H), 4.29 (d, *J* = 81.4 Hz, 1H), 3.81 (s, 3H), 2.81 (t, *J* = 16.3 Hz, 1H), 2.47 – 2.36 (m, 1H), δ 2.40 (dd, *J* = 181.5, 14.3 Hz, 1H), 2.29 (d, *J* = 13.7 Hz, 1H), 1.97 (d, *J* = 10.8 Hz, 3H), 1.94 – 1.79 (m, 3H), 1.55 (d, *J* = 8.6 Hz, 1H), 1.54 – 1.18 (m, 9H).

^{13}C NMR (125 MHz, $CDCl_3$) δ 168.6, 168.3, 158.9, 141.0, 130.1, 115.7, 113.7, 82.8, 56.3, 55.2, 51.5, 39.4, 38.9, 37.6, 37.1, 29.0, 28.6, 28.1, 27.4, 27.0, 20.4.

$[\alpha]_D^{28}$ = -66.4 (c = 0.11, CH_2Cl_2).

HRMS (ESI) ($[M + Na]^+$) Calcd. for $[C_{24}H_{31}NaNO_4]^+$: 420.2145, Found. 420.2145.

HRMS spectrum of **3aq**

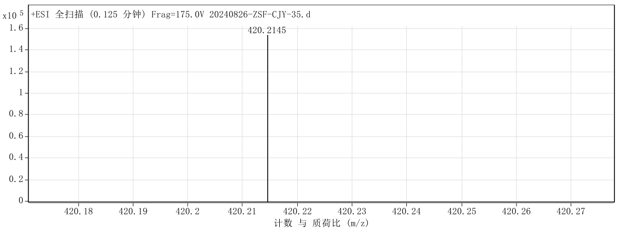

HPLC: AD-H column, hexane/^{i}PrOH = 95:5, 1.0 mL/min, t_R = 13.59 min (major), 16.66 min (minor).

Chiral HPLC spectra of (*rac*)-**3aq** and **3aq**

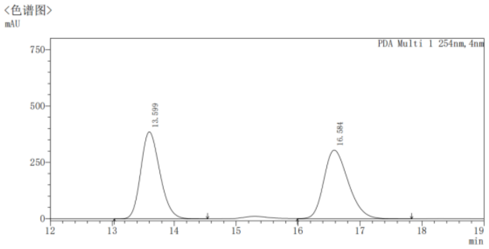

<峰表>
PDA Ch1 254nm

峰号	保留时间	面积	高度	高度%	面积%
1	13.599	8289665	386205	55.881	49.712
2	16.584	8385604	304911	44.119	50.288
总计		16675269	691116	100.000	100.000

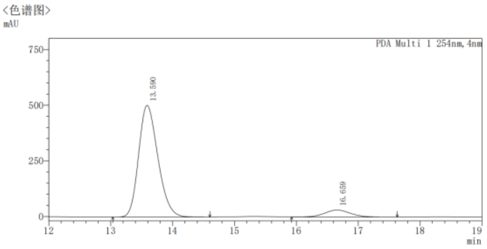

<峰表>
PDA Ch1 254nm

峰号	保留时间	面积	高度	高度%	面积%
1	13.590	10868366	501986	94.113	92.228
2	16.659	915850	31401	5.887	7.772
总计		11784216	533387	100.000	100.000

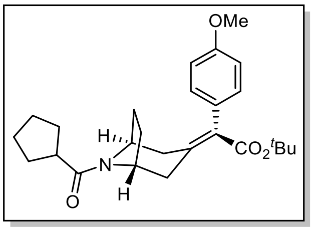

***tert*-butyl-*(Z)*-2-(*(1R,5S)*-8-(cyclopentanecarbonyl)-8-azabicyclo[3.2.1]octan-3-ylidene)-2-(4-methoxyphenyl)acetate (3ar)**

24.7 mg, 58% yield, 98% ee, colorless oil, R_f = 0.58 (PE/EtOAc = 3:1).

^{1}H NMR (500 MHz, CDCl$_3$) δ 7.11 (dd, *J* = 11.7, 8.7 Hz, 2H), 6.86 (dd, *J* = 8.8, 3.0 Hz, 2H), 4.71 (ddt, *J* = 79.3, 6.8, 3.0 Hz, 1H), 4.27 (ddt, *J* = 83.1, 6.0, 3.0 Hz, 1H), 3.80 (d, *J* = 1.3 Hz, 3H), 2.91 – 2.82 (m, 1H), 2.79 – 2.70 (m, 1H), 2.55 – 2.40 (m, 1H), 2.36 – 2.29 (m, 1H), 2.27 – 2.19 (m, 1H), 2.02 – 1.85 (m, 3H), 1.84 – 1.68 (m, 6H), 1.64 – 1.50 (m, 3H), 1.43 (s, 9H).

^{13}C NMR (125 MHz, CDCl$_3$) δ 172.4, 172.3, 168.4, 168.4, 158.8, 158.8, 136.8, 136.3, 135.3, 130.1, 130.1, 129.0, 128.9, 116.0, 114.7, 113.7, 81.4, 81.2, 55.2, 55.2, 54.7, 54.7, 51.8, 51.8, 42.2, 42.2, 39.4, 38.9, 37.8, 37.2, 30.6, 30.5, 30.3, 30.2, 29.3, 28.9, 28.1, 27.3, 27.0, 26.2, 26.2, 26.1, 26.1.

$[\alpha]_D^{28}$ = -144.2 (c = 0.20, CH_2Cl_2).

HRMS (ESI) ($[M + Na]^+$) Calcd. for $[C_{26}H_{35}NaNO_4]^+$: 448.2458, Found. 448.2464.

HRMS spectrum of **3ar**

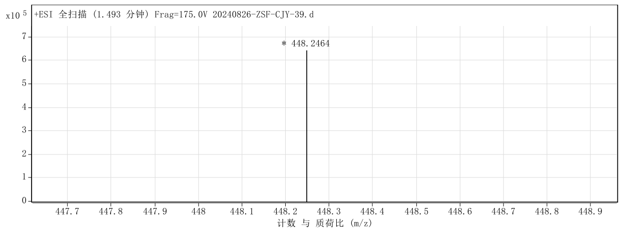

HPLC: AD-H column, hexane/^{i}PrOH = 90:10, 1.0 mL/min, t_R = 7.90 min (minor), 9.19 min (major).

Chiral HPLC spectra of (*rac*)-**3ar** and **3ar**

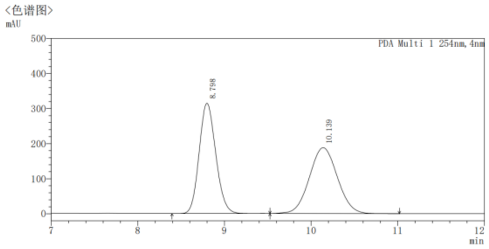

<峰表>
PDA Ch1 254nm

峰号	保留时间	面积	高度	高度%	面积%
1	8.798	4202630	314844	62.619	49.818
2	10.139	4233270	187951	37.381	50.182
总计		8435900	502795	100.000	100.000

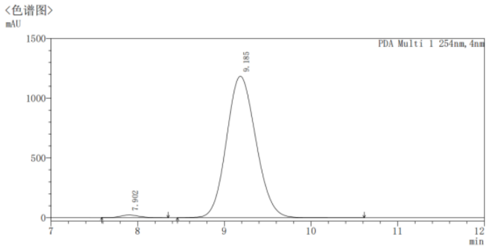

<峰表>
PDA Ch1 254nm

峰号	保留时间	面积	高度	高度%	面积%
1	7.902	321405	22472	1.863	1.140
2	9.185	27882640	1184061	98.137	98.860
总计		28204044	1206534	100.000	100.000

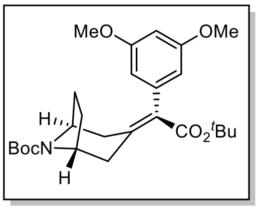

***tert*-butyl-*(1R,5S,Z)*-3-(2-(tert-butoxy)-1-(3,5-dimethoxyphenyl)-2-oxoethylidene)-8-azabicyclo[3.2.1]octane-8-carboxylate (3as)**

38.6 mg, 84% yield, 99% ee, colorless oil, R_f = 0.38 (PE/EtOAc = 10:1).

^{1}H NMR (500 MHz, CDCl$_3$) δ 6.37 (t, *J* = 2.2 Hz, 1H), 6.35 (s, 2H), 4.21 (d, *J* = 25.4 Hz, 1H), δ 4.21 (d, *J* = 124.4 Hz, 1H), 3.77 (s, 6H), 2.70 (d, *J* = 14.5 Hz, 1H), 2.60 – 2.29 (m, 2H), 2.21 (d, *J* = 11.5 Hz, 1H), 1.97 – 1.70 (m, 4H), 1.47 (s, 9H), 1.44 (s, 9H).

^{13}C NMR (125 MHz, CDCl$_3$) δ 168.0, 160.6, 153.5, 138.0, 135.2, 107.1, 105.5, 99.5, 99.3, 81.3, 79.5, 55.4, 55.3, 54.2, 53.7, 37.8, 37.4, 37.2, 36.7, 29.7, 28.8, 28.5, 28.4, 28.1, 28.0, 27.7.

$[\alpha]_D^{28}$ = -67.5 (c = 0.29, CH_2Cl_2).

HRMS (ESI) ($[M + H]^+$) Calcd. for $[C_{26}H_{38}NO_6]^+$: 460.2694, Found. 460.2694.

HRMS spectrum of **3as**

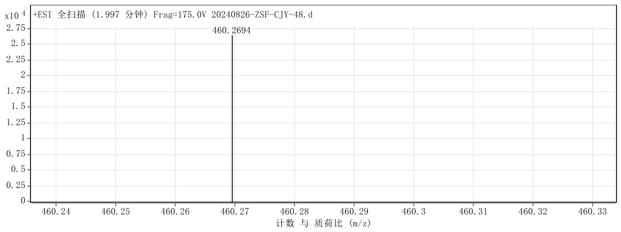

HPLC: AD-H column, hexane/^{i}PrOH = 99:1, 1.0 mL/min, t_R = 15.47 min (minor), 16.49 min (major).

Chiral HPLC spectra of (*rac*)-**3as** and **3as**

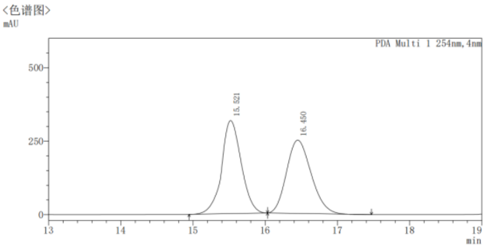

<峰表>
PDA Ch1 254nm

峰号	保留时间	面积	高度	高度%	面积%
1	15.521	5987527	316830	55.956	50.346
2	16.450	5905246	249381	44.044	49.654
总计		11892773	566211	100.000	100.000

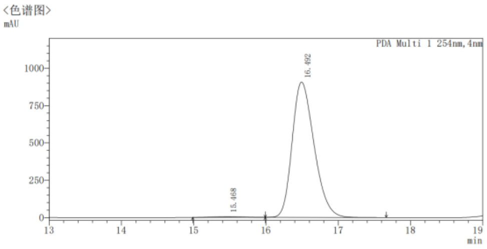

<峰表>
PDA Ch1 254nm

峰号	保留时间	面积	高度	高度%	面积%
1	15.468	141197	5081	0.557	0.732
2	16.492	19153183	907003	99.443	99.268
总计		19294381	912083	100.000	100.000

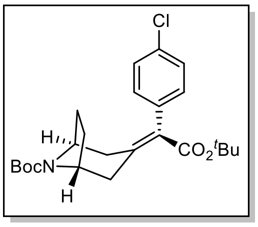

***tert*-butyl-*(1R,5S,Z)*-3-(2-(tert-butoxy)-1-(4-chlorophenyl)-2-oxoethylidene)-8-azabicyclo[3.2.1]octane-8-carboxylate (3at)**

35.7 mg, 82% yield, 99% ee, colorless oil, R_f = 0.41 (PE/EtOAc = 10:1).

^{1}H NMR (500 MHz, $CDCl_3$) δ 7.23 (d, *J* = 8.0 Hz, 2H), 7.06 (d, *J* = 7.9 Hz, 2H), 4.23 (d, *J* = 47.3 Hz, 1H), 4.07 (d, *J* = 47.1 Hz, 1H), 2.73 (d, *J* = 14.7 Hz, 1H), 2.43 (dd, *J* = 53.5, 14.4 Hz, 1H), 2.23 (dd, *J* = 67.4, 11.9 Hz, 1H), 2.03 (d, *J* = 14.7 Hz, 1H), 1.83 (t, *J* = 17.6 Hz, 2H), 1.68 (t, *J* = 9.4 Hz, 1H), 1.43 (s, 1H), 1.41 (s, 9H), 1.36 (s, 9H).

^{13}C NMR (125 MHz, $CDCl_3$) δ 166.7, 152.5, 138.7, 138.5, 134.5, 133.1, 132.2, 129.4, 127.5, 80.5, 78.5, 53.2, 52.6, 36.8, 36.5, 36.2, 35.8, 28.7, 27.5, 27.1, 26.7.

$[\alpha]_D^{28}$ = -137.7 (c = 0.26, CH_2Cl_2).

HRMS (ESI) ($[M + Na]^+$) Calcd. for $[C_{24}H_{32}NaClNO_4]^+$: 456.1912, Found. 456.1919.

HRMS spectrum of **3at**

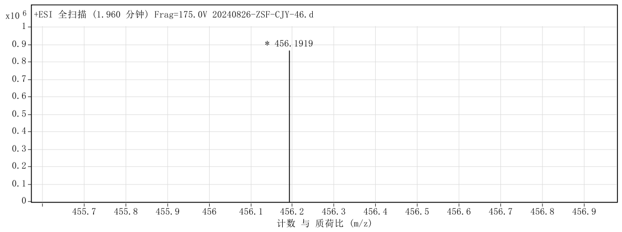

HPLC: AD-H column, hexane/^{i}PrOH = 99:1, 1.0 mL/min, t_R = 9.91 min (major), 10.53 min (minor).

Chiral HPLC spectra of (*rac*)-**3at** and **3at**

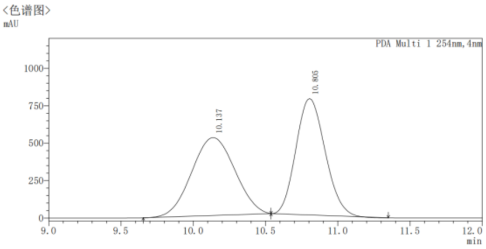

<峰表>
PDA Ch1 254nm

峰号	保留时间	面积	高度	高度%	面积%
1	10.137	10832407	520280	40.075	49.746
2	10.805	10942913	777997	59.925	50.254
总计		21775320	1298277	100.000	100.000

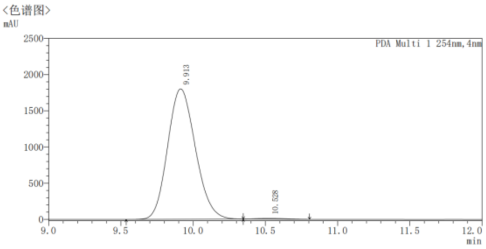

<峰表>
PDA Ch1 254nm

峰号	保留时间	面积	高度	高度%	面积%
1	9.913	24093824	1801707	99.415	99.419
2	10.528	140725	10609	0.585	0.581
总计		24234549	1812315	100.000	100.000

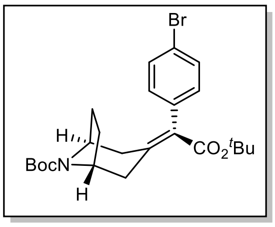

***tert*-butyl-*(1R,5S,Z)*-3-(1-(4-bromophenyl)-2-(tert-butoxy)-2-oxoethylidene)-8-azabicyclo[3.2.1]octane-8-carboxylate (3au)**

35.3 mg, 74% yield, >99% ee, colorless oil, R_f = 0.42 (PE/EtOAc = 10:1).

^{1}H NMR (500 MHz, $CDCl_3$) δ 7.46 (d, *J* = 8.1 Hz, 2H), 7.07 (d, *J* = 7.9 Hz, 2H), 4.25 (d, 1H), 4.14 (d, *J* = 48.7 Hz, 1H), 2.80 (d, *J* = 15.0 Hz, 1H), 2.50 (dd, *J* = 55.8, 14.2 Hz, 1H), 2.29 (dd, *J* = 69.0, 14.1 Hz, 1H), 2.10 (d, *J* = 14.7 Hz, 1H), 1.90 (s, 2H), 1.75 (t, *J* = 9.2 Hz, 2H), 1.48 (s, 9H), 1.43 (s, 9H).

^{13}C NMR (125 MHz, $CDCl_3$) δ 167.7, 153.5, 134.1, 131.4, 130.8, 121.4, 81.5, 79.6, 54.2, 53.6, 37.8, 37.4, 37.2, 36.8, 29.7, 28.5, 28.1, 28.1.

$[\alpha]_D^{28}$ = -64.0 (c = 0.35, CH_2Cl_2).

HRMS (ESI) ($[M + Na]^+$) Calcd. for $[C_{24}H_{32}NaBrNO_4]^+$: 500.1407, Found. 500.1407.

HRMS spectrum of **3au**

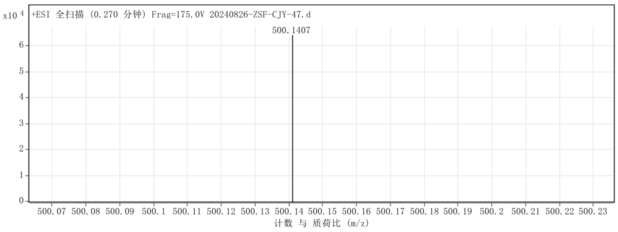

HPLC: INA column, hexane/^{i}PrOH = 97:3, 1.0 mL/min, t_R = 4.89 min (major).

Chiral HPLC spectra of (*rac*)-**3au** and **3au**

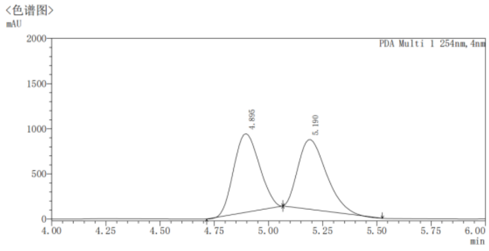

<峰表>
PDA Ch1 254nm

峰号	保留时间	面积	高度	高度%	面积%
1	4.895	7197693	871249	53.037	50.759
2	5.190	6982502	771457	46.963	49.241
总计		14180195	1642705	100.000	100.000

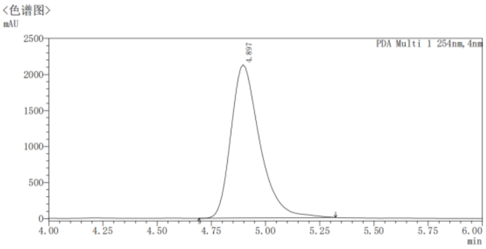

<峰表>
PDA Ch1 254nm

峰号	保留时间	面积	高度	高度%	面积%
1	4.897	19652254	2129204	100.000	100.000
总计		19652254	2129204	100.000	100.000

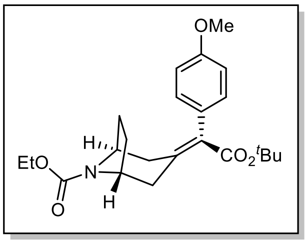

ethyl-*(1R,5S,Z)*-3-(2-(*tert*-butoxy)-1-(4-methoxyphenyl)-2-oxoethylidene)-8-azabicyclo[3.2.1]octane-8-carboxylate (3av)

11.7 mg, 29% yield, 98% ee, colorless oil, R_f = 0.56 (PE/EtOAc = 5:1).

^{1}H NMR (500 MHz, $CDCl_3$) δ 7.11 (d, *J* = 8.2 Hz, 2H), 6.86 (d, *J* = 8.6 Hz, 2H), 4.36 (d, *J* = 29.7 Hz, 1H), 4.23 (s, 1H), 4.16 (s, 2H), 3.80 (s, 3H), 2.72 (d, *J* = 14.6 Hz, 1H), 2.50 (d, *J* = 33.8 Hz, 1H), 2.34 (s, 1H), 2.20 (d, *J* = 14.4 Hz, 1H), 1.97 – 1.76 (m, 3H), 1.53 – 1.46 (m, 1H), 1.44 (s, 9H), 1.30 – 1.24 (m, 3H).

^{13}C NMR (125 MHz, $CDCl_3$) δ 168.5, 158.8, 154.0, 136.8, 135.1, 130.1, 129.0, 113.7, 81.2, 61.0, 55.2, 54.0, 53.9, 38.0, 37.4, 29.7, 28.1, 14.8.

$[\alpha]_D^{28}$ = -78.6 (c = 0.12, CH_2Cl_2).

HRMS (ESI) ($[M + Na]^+$) Calcd. for $[C_{23}H_{31}NaNO_5]^+$: 424.2094, Found. 424.2094.

HRMS spectrum of **3av**

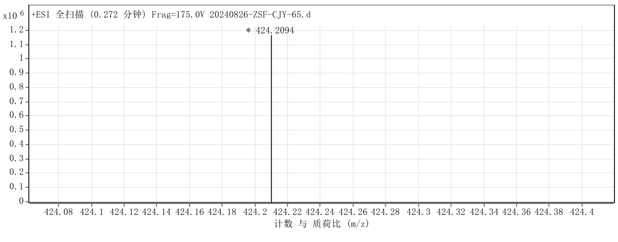

HPLC: INA column, hexane/^{i}PrOH = 90:10, 1.0 mL/min, t_R = 5.08 min (minor), 5.52 min (major).

Chiral HPLC spectra of (*rac*)-**3av** and **3av**

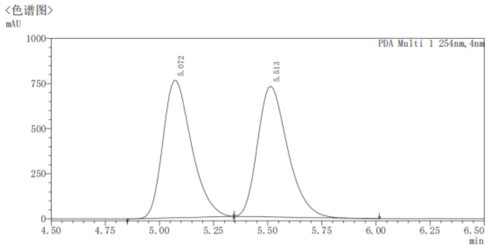

<峰表>
PDA Ch1 254nm

峰号	保留时间	面积	高度	高度%	面积%
1	5.072	6994697	759565	51.196	49.994
2	5.513	6996312	724080	48.804	50.006
总计		13991009	1483645	100.000	100.000

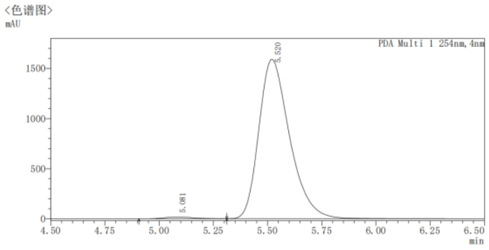

<峰表>
PDA Ch1 254nm

峰号	保留时间	面积	高度	高度%	面积%
1	5.081	175080	19289	1.200	1.099
2	5.520	15749298	1588304	98.800	98.901
总计		15924378	1607593	100.000	100.000

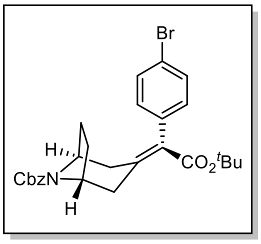

benzyl-*(1R,5S,Z)*-3-(1-(4-bromophenyl)-2-(*tert*-butoxy)-2-oxoethylidene)-8-azabicyclo[3.2.1]octane-8-carboxylate (3aw)

36.3 mg, 71% yield, 98% ee, colorless oil, R_f = 0.46 (PE/EtOAc = 5:1).

^{1}H NMR (500 MHz, $CDCl_3$) δ 7.46 (d, *J* = 8.1 Hz, 2H), 7.41 – 7.30 (m, 5H), 7.06 (d, *J* = 8.2 Hz, 2H), 5.16 (s, 2H), 4.34 (dd, *J* = 80.4, 20.4 Hz, 2H), 2.83 (d, *J* = 14.6 Hz, 1H), 2.51 (dd, *J* = 52.3, 13.6 Hz, 1H), 2.31 (dd, *J* = 67.2, 14.7 Hz, 1H), 2.13 (d, *J* = 14.5 Hz, 1H), 2.03 – 1.82 (m, 2H), 1.84 – 1.74 (m, 1H), 1.46 (d, *J* = 7.6 Hz, 1H), 1.42 (s, 9H).

^{13}C NMR (125 MHz, $CDCl_3$) δ 166.5, 152.6, 135.8, 134.8, 133.4, 130.4, 129.7, 127.5, 127.0, 126.9, 120.4, 80.5, 65.8, 53.0, 52.9, 37.0, 36.7, 36.3, 36.0, 28.7, 27.8, 27.5, 27.1, 27.0, 26.8, 26.7.

$[\alpha]_D^{28}$ = -121.7 (c = 0.31, CH_2Cl_2).

HRMS (ESI) ($[M + Na]^+$) Calcd. for $[C_{27}H_{30}NaBrNO_4]^+$: 534.1250, Found. 534.1250.

HRMS spectrum of **3aw**

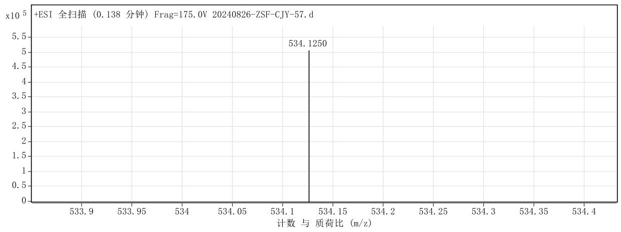

HPLC: INA column, hexane/^{i}PrOH = 97:3, 1.0 mL/min, t_R = 10.25 min (minor), 11.56 min (major).

Chiral HPLC spectra of (*rac*)-**3aw** and **3aw**

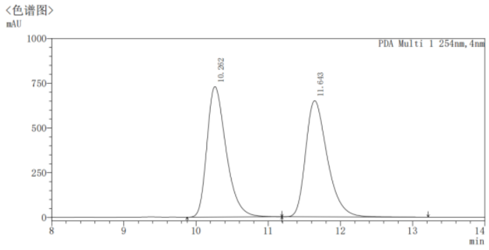

<峰表>
PDA Ch1 254nm

峰号	保留时间	面积	高度	高度%	面积%
1	10.262	13485991	729458	52.913	49.927
2	11.643	13525373	649145	47.087	50.073
总计		27011364	1378603	100.000	100.000

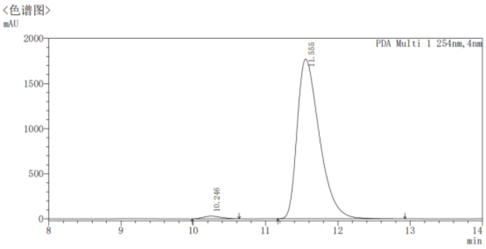

<峰表>
PDA Ch1 254nm

峰号	保留时间	面积	高度	高度%	面积%
1	10.246	522229	30593	1.700	1.363
2	11.555	37781263	1768967	98.300	98.637
总计		38303491	1799560	100.000	100.000

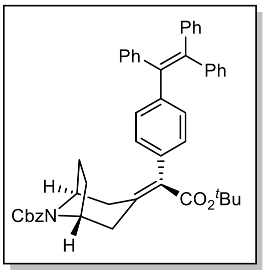

benzyl-*(1R,5S,Z)*-3-(2-(*tert*-butoxy)-2-oxo-1-(4-(1,2,2-triphenylvinyl)phenyl)ethylidene)-8-azabicyclo[3.2.1]octane-8-carboxylate (3ax)

45.3 mg, 66% yield, 99% ee, colorless oil, R_f = 0.48 (PE/EtOAc = 5:1).

^{1}H NMR (400 MHz, CDCl$_3$) δ 7.40 (d, *J* = 4.6 Hz, 4H), 7.38 – 7.32 (m, 1H), 7.13 (td, *J* = 6.3, 5.7, 2.1 Hz, 6H), 7.11 – 7.00 (m, 10H), 6.99 (s, 1H), 6.92 (d, *J* = 8.3 Hz, 2H), 5.19 (s, 2H), 4.43 (d, *J* = 17.6 Hz, 1H), 4.29 (d, *J* = 17.8 Hz, 1H), 2.87 – 2.77 (m, 1H), 2.51 (dd, *J* = 39.8, 14.4 Hz, 1H), 2.27 (d, *J* = 47.5 Hz, 1H), 2.15 (d, *J* = 14.6 Hz, 1H), 2.02 – 1.76 (m, 3H), 1.52 (dd, *J* = 9.6, 2.9 Hz, 1H), 1.43 (s, 9H).

^{13}C NMR (100 MHz, CDCl$_3$) δ 168.0, 143.7, 143.6, 143.4, 142.7, 141.3, 140.7, 136.9, 135.5, 134.9, 131.3, 131.3, 131.1, 128.5, 128.3, 128.0, 127.9, 127.7, 127.7, 127.5, 126.5, 81.2, 66.8, 54.1, 28.1.

$[\alpha]_D^{28}$ = -60.0 (c = 0.28, CH_2Cl_2).

HRMS (ESI) ($[M + Na]^+$) Calcd. for $[C_{47}H_{45}NaNO_4]^+$: 710.3241, Found. 710.3251.

HRMS spectrum of **3ax**

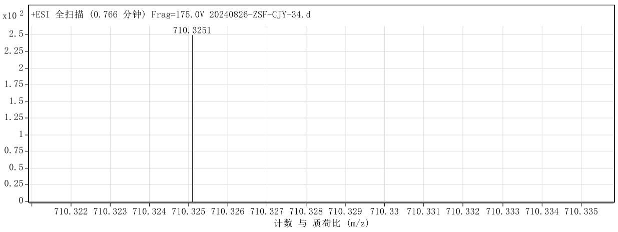

HPLC: AD-H column, hexane/^{i}PrOH = 95:5, 1.0 mL/min, t_R = 6.82 min (major), 11.05 min (minor).

Chiral HPLC spectra of (*rac*)-**3ax** and **3ax**

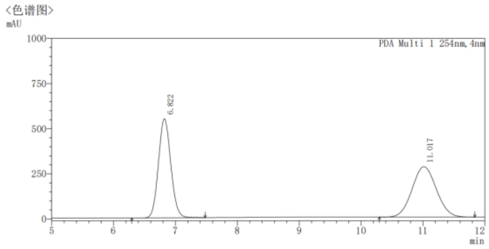

<峰表>
PDA Ch1 254nm

峰号	保留时间	面积	高度	高度%	面积%
1	6.822	7608395	548500	66.306	49.974
2	11.017	7616192	278723	33.694	50.026
总计		15224587	827223	100.000	100.000

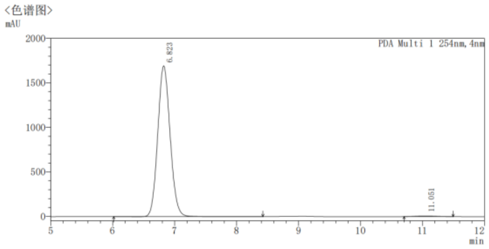

<峰表>
PDA Ch1 254nm

峰号	保留时间	面积	高度	高度%	面积%
1	6.823	23471163	1693947	99.621	99.343
2	11.051	155200	6439	0.379	0.657
总计		23626362	1700385	100.000	100.000

For the synthesis of **3ay**:

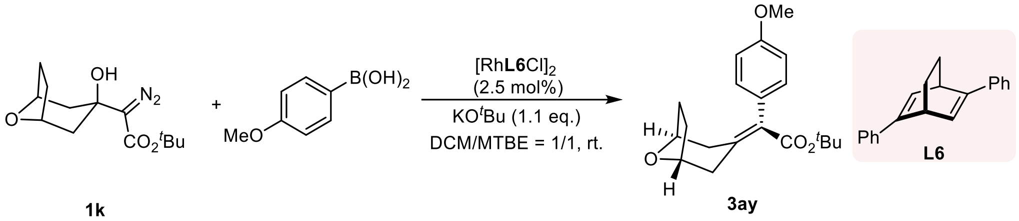

Under nitrogen atmosphere, to a solution of KO^{t}Bu (1.1 eq.) and 4-Methoxyphenylboronic acid (2.0 eq.) in DCM/MTBE (0.05 M), 2.5 mol% [Rh(**L6**)Cl]$_2$ (dissolved in 0.1 mL DCM) was added. Then, the α-diazoester **1k** (0.1 mmol dissolved in another 0.5 mL DCM) was introduced in one portion into the above system. The resulting mixture was stirred at room temperature for 12 h. After the reaction was completed, the reaction mixture was filtered through short silica gel, and then the solvent was removed under reduced pressure. The crude product was purified by flash column chromatography on silica gel with EtOAc–petroleum ether to yield the product **3ay**.

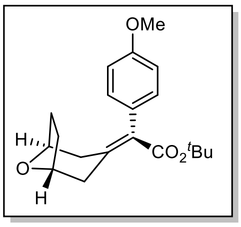

***tert*-butyl-*(Z)*-2-(*(1R,5S)*-8-oxabicyclo[3.2.1]octan-3-ylidene)-2-(4-methoxyphenyl)acetate (3ay)**

19.7 mg, 60% yield, 99% ee, colorless oil, R_f = 0.63 (PE/EtOAc = 3:1).

^{1}H NMR (500 MHz, CDCl$_3$) δ 7.18 – 7.08 (m, 2H), 6.91 – 6.82 (m, 2H), 4.51 (dd, *J* = 4.1, 2.2 Hz, 1H), 4.41 – 4.31 (m, 1H), 3.81 (s, 3H), 2.71 (d, *J* = 14.4 Hz, 1H), 2.54 (dd, *J* = 14.5, 3.6 Hz, 1H), 2.34 (dd, 1H), 2.12 (d, *J* = 14.5 Hz, 1H), 1.93 – 1.81 (m, 3H), 1.62 – 1.53 (m, 1H), 1.44 (s, 9H).

^{13}C NMR (125 MHz, CDCl$_3$) δ 168.4, 158.7, 137.8, 133.8, 130.2, 129.2, 113.6, 81.0, 75.4, 75.3, 55.2, 38.5, 38.1, 29.1, 28.7, 28.1.

$[\alpha]_D^{28}$ = -82.3 (c = 0.15, CH_2Cl_2).

HRMS (ESI) ($[M + Na]^+$) Calcd. for $[C_{20}H_{26}NaO_4]^+$: 353.1723, Found. 353.1728.

HRMS spectrum of **3ay**

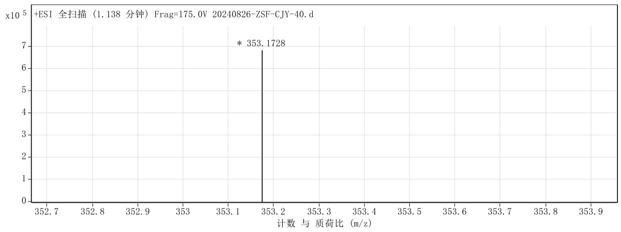

HPLC: AD-H column, hexane/^{i}PrOH = 99:1, 0.8 mL/min, t_R = 14.56 min (minor), 15.11 min (major).

Chiral HPLC spectra of (*rac*)-**3ay** and **3ay**

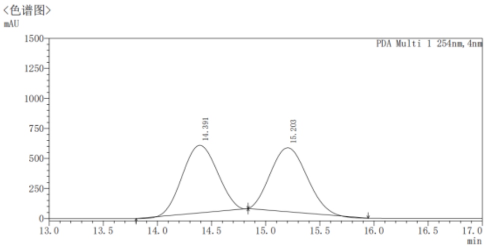

<峰表>
PDA Ch1 254nm

峰号	保留时间	面积	高度	高度%	面积%
1	14.391	12610414	561987	51.337	50.318
2	15.203	12450910	532717	48.663	49.682
总计		25061324	1094704	100.000	100.000

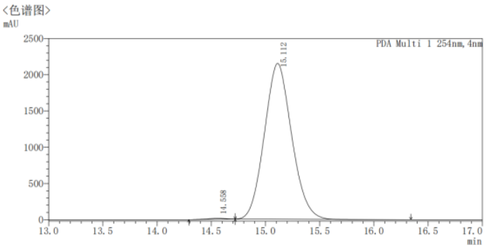

<峰表>
PDA Ch1 254nm

峰号	保留时间	面积	高度	高度%	面积%
1	14.558	153360	12752	0.589	0.407
2	15.112	37549895	2150884	99.411	99.593
总计		37703255	2163636	100.000	100.000

For the synthesis of **4**:

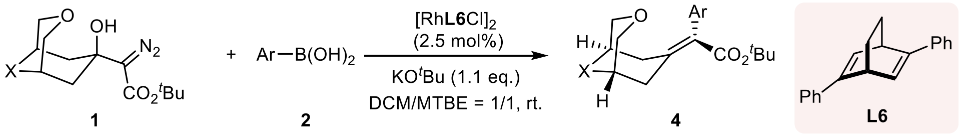

Under nitrogen atmosphere, to a solution of KO^{t}Bu (1.1 eq.) and $ArB(OH)_2$ (2.0 eq.) in DCM/MTBE (0.05 M), 2.5 mol% $[Rh(\mathbf{L6})Cl]_2$ (dissolved in 0.1 mL DCM) was added. Then, the α-diazoester **1** (0.1 mmol dissolved in another 0.5 mL DCM) was introduced in one portion into the above system. The resulting mixture was stirred at room temperature for 12 h. After the reaction was completed, the reaction mixture was filtered through short silica gel, and then the solvent was removed under reduced pressure. The crude product was purified by flash column chromatography on silica gel with EtOAc–petroleum ether to yield the products.

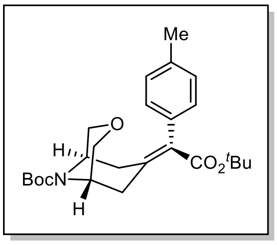

***tert*-butyl-*(1R,5S,E)*-7-(2-(tert-butoxy)-2-oxo-1-(p-tolyl)ethylidene)-3-oxa-9-azabicyclo[3.3.1]nonane-9-carboxylate (4a)**

34.3 mg, 80% yield, 93% ee, colorless oil, R_f = 0.52 (PE/EtOAc = 10:1).

^{1}H NMR (500 MHz, CDCl$_3$) δ 7.13 (d, *J* = 7.8 Hz, 2H), 7.08 (dd, *J* = 8.0, 4.4 Hz, 2H), 4.26 – 3.92 (m, 1H), 4.14 – 4.03 (m, 1H), 3.92 – 3.84 (m, 1H), 3.73 (tdd, *J* = 11.7, 9.1, 2.5 Hz, 1H), 3.69 – 3.63 (m, 1H), 3.39 – 3.25 (m, 1H), 2.55 (ddd, *J* = 25.2, 15.2, 5.3 Hz, 1H), 2.37 (d, *J* = 4.9 Hz, 1H), 2.34 (s, 3H), 2.33 – 2.19 (m, 1H), 1.87 – 1.72 (m, 1H), 1.47 (dd, *J* = 17.0, 2.3 Hz, 9H), 1.42 (d, *J* = 1.9 Hz, 9H).

^{13}C NMR (125 MHz, CDCl$_3$) δ 168.2, 168.0, 153.9, 153.8, 143.5, 143.4, 136.4, 129.2, 128.8, 80.7, 80.6, 80.2, 80.1, 71.1, 71.0, 70.8, 70.7, 69.8, 69.6, 50.2, 48.7, 48.6, 48.3, 46.6, 34.7, 34.5, 34.1, 34.0, 28.4, 28.4, 28.4, 28.2, 28.1, 21.2.

$[\alpha]_D^{28}$ = -46.9 (c = 0.24, CH_2Cl_2).

HRMS (ESI) ($[M + Na]^+$) Calcd. for $[C_{25}H_{35}NaNO_5]^+$: 452.2407, Found. 452.2402.

HRMS spectrum of **4a**

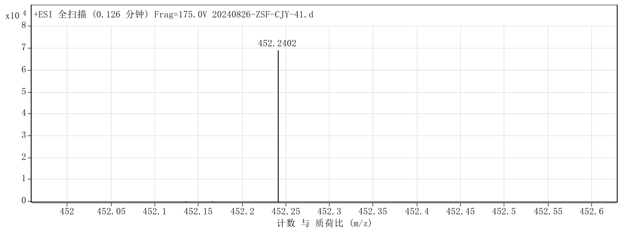

HPLC: INA column, hexane/^{i}PrOH = 97:3, 1.0 mL/min, t_R = 6.45 min (minor), 8.14 min (major).

Chiral HPLC spectra of (*rac*)-**4a** and **4a**

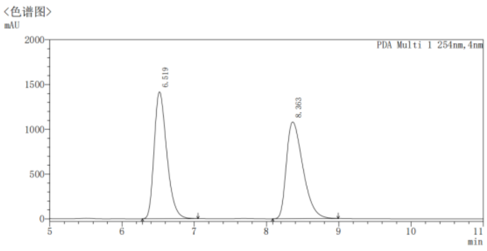

<峰表>
PDA Ch1 254nm

峰号	保留时间	面积	高度	高度%	面积%
1	6.519	17208052	1418273	56.735	50.006
2	8.363	17204218	1081537	43.265	49.994
总计		34412271	2499810	100.000	100.000

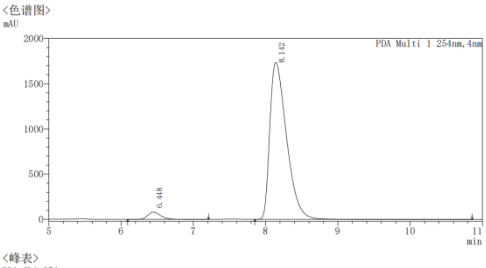

<峰表>
PDA Ch1 254nm

峰号	保留时间	面积	高度	高度%	面积%
1	6.448	1033174	81145	4.463	3.557
2	8.142	28014437	1736905	95.537	96.443
总计		29047611	1818050	100.000	100.000

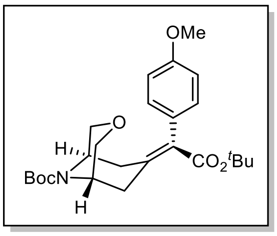

***tert*-butyl-*(1R,5S,E)*-7-(2-(tert-butoxy)-1-(4-methoxyphenyl)-2-oxoethylidene)-3-oxa-9-azabicyclo[3.3.1]nonane-9-carboxylate (4b)**

24.6 mg, 55% yield, 95% ee, colorless oil, R_f = 0.38 (PE/EtOAc = 10:1).

^{1}H NMR (500 MHz, $CDCl_3$) δ 7.13 (dd, *J* = 8.6, 3.9 Hz, 2H), 6.95 – 6.83 (m, 2H), 4.11 (dd, *J* = 14.2, 4.8 Hz, 1H), 4.10 (dd, *J* = 109.6, 5.0 Hz, 1H), 3.90 (dd, *J* = 15.8, 11.2 Hz, 1H), 3.83 (s, 3H), 3.74 (t, 1H), 3.68 (d, *J* = 11.3 Hz, 2H), 3.36 – 3.26 (m, 1H), 2.56 (ddd, *J* = 20.3, 15.1, 5.3 Hz, 1H), 2.42 (dd, *J* = 15.1, 4.5 Hz, 1H), 2.28 (ddd, *J* = 28.9, 15.2, 5.4 Hz, 1H), 1.49 (d, *J* = 13.0 Hz, 9H), 1.44 (d, *J* = 1.7 Hz, 9H).

^{13}C NMR (125 MHz, $CDCl_3$) δ 168.2, 168.1, 158.4, 153.9, 153.8, 143.6, 143.5, 130.5, 130.3, 128.7, 128.7, 113.5, 80.6, 80.6, 80.2, 80.1, 71.0, 70.8, 55.2, 55.2, 50.2, 50.2, 48.6, 34.6, 34.5, 34.1, 34.0, 28.4, 28.4, 28.2, 28.2.

$[\alpha]_D^{28}$ = -35.2 (c = 0.20, CH_2Cl_2).

HRMS (ESI) ($[M + Na]^+$) Calcd. for $[C_{25}H_{35}NaNO_6]^+$: 468.2356, Found. 468.2361.

HRMS spectrum of **4b**

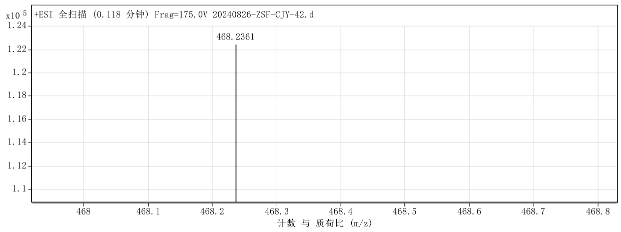

HPLC: AD-H column, hexane/^{i}PrOH = 97:3, 1.0 mL/min, t_R = 13.12 min (minor), 18.60 min (major).

Chiral HPLC spectra of (*rac*)-**4b** and **4b**

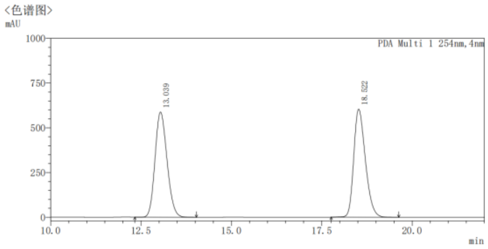

<峰表>
PDA Ch1 254nm

峰号	保留时间	面积	高度	高度%	面积%
1	13.039	13579852	588130	49.319	50.049
2	18.522	13553306	604367	50.681	49.951
总计		27133158	1192497	100.000	100.000

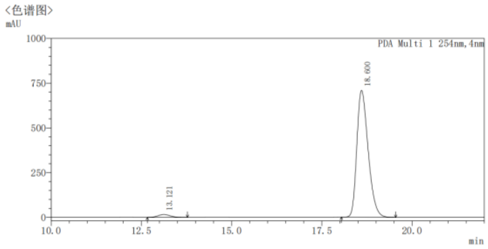

<峰表>
PDA Ch1 254nm

峰号	保留时间	面积	高度	高度%	面积%
1	13.121	364212	16020	2.210	2.305
2	18.600	15435961	708963	97.790	97.695
总计		15800173	724983	100.000	100.000

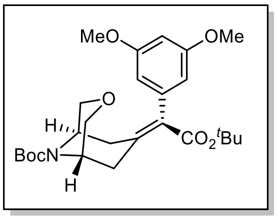

***tert*-butyl-*(1R,5S,E)*-7-(2-(tert-butoxy)-1-(3,5-dimethoxyphenyl)-2-oxoethylidene)-3-oxa-9-azabicyclo[3.3.1]nonane-9-carboxylate (4c)**

23.9 mg, 50% yield, 96% ee, colorless oil, R_f = 0.26 (PE/EtOAc = 10:1).

^{1}H NMR (500 MHz, CDCl$_3$) δ 6.47 – 6.25 (m, 3H), 4.10 (dd, *J* = 12.3, 4.7 Hz, 1H), 4.10 (dd, *J* = 136.4, 4.3 Hz, 1H), 3.88 (dd, *J* = 19.7, 11.3 Hz, 1H), 3.77 (d, 6H), 3.73 (d, *J* = 11.1 Hz, 1H), 3.71 – 3.65 (m, 2H), 3.30 (d, *J* = 15.2 Hz, 1H), 2.54 (ddd, *J* = 24.9, 15.1, 5.3 Hz, 1H), 2.43 (dd, *J* = 15.3, 5.3 Hz, 1H), 2.28 (ddd, *J* = 38.4, 15.3, 5.4 Hz, 1H), 1.50 – 1.44 (m, 9H), 1.43 (d, *J* = 1.8 Hz, 9H).

^{13}C NMR (125 MHz, CDCl$_3$) δ 167.7, 167.6, 160.5, 153.9, 153.8, 144.1, 144.0, 140.1, 139.9, 129.0, 128.9, 107.3, 107.3, 99.4, 99.3, 80.8, 80.7, 80.3, 80.2, 71.0, 71.0, 70.7, 70.7, 55.3, 50.2, 48.7, 48.6, 34.8, 34.4, 34.2, 34.0, 28.4, 28.4, 28.2, 28.2.

$[\alpha]_D^{28}$ = -30.2 (c = 0.19, CH_2Cl_2).

HRMS (ESI) ($[M + Na]^+$) Calcd. for $[C_{26}H_{37}NaNO_7]^+$: 498.2462, Found. 498.2468.

HRMS spectrum of **4c**

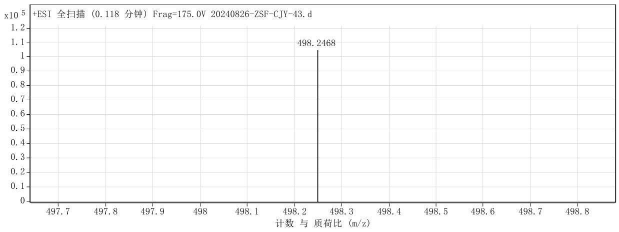

HPLC: INA column, hexane/^{i}PrOH = 97:3, 1.0 mL/min, t_R = 7.33 min (minor), 7.89 min (major).

Chiral HPLC spectra of (*rac*)-**4c** and **4c**

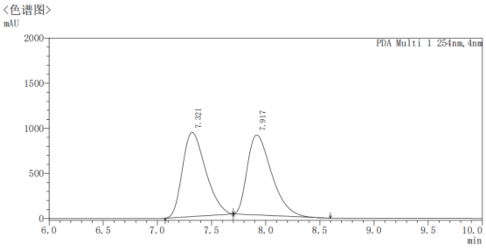

<峰表>
PDA Ch1 254nm

峰号	保留时间	面积	高度	高度%	面积%
1	7.321	13930334	931483	51.248	50.176
2	7.917	13832449	886128	48.752	49.824
总计		27762783	1817612	100.000	100.000

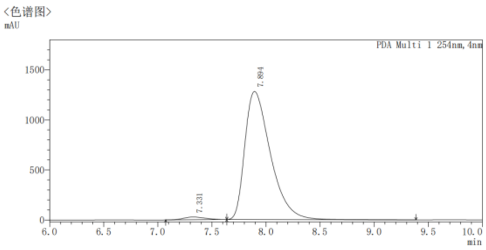

<峰表>
PDA Ch1 254nm

峰号	保留时间	面积	高度	高度%	面积%
1	7.331	365995	26557	2.030	1.706
2	7.894	21093619	1281369	97.970	98.294
总计		21459613	1307926	100.000	100.000

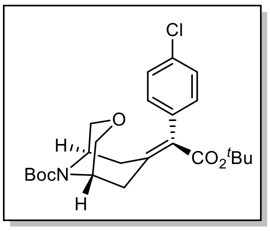

***tert*-butyl-*(1R,5S,E)*-7-(2-(tert-butoxy)-1-(4-chlorophenyl)-2-oxoethylidene)-3-oxa-9-azabicyclo[3.3.1]nonane-9-carboxylate (4d)**

25.8 mg, 57% yield, 94% ee, colorless oil, R_f = 0.46 (PE/EtOAc = 10:1).

^{1}H NMR (500 MHz, CDCl$_3$) δ 7.29 (d, *J* = 8.0 Hz, 2H), 7.12 (dd, *J* = 8.0, 5.3 Hz, 2H), 4.09 (d, *J* = 17.6 Hz, 1H), 4.09 (d, *J* = 140.0 Hz, 1H), 3.87 (dd, *J* = 19.6, 11.2 Hz, 1H), 3.71 (t, *J* = 11.1 Hz, 1H), 3.67 –

3.58 (m, 2H), 3.41 (dd, J = 15.4, 5.8 Hz, 1H), 2.53 (ddd, J = 24.4, 15.3, 5.3 Hz, 1H), 2.37 – 2.16 (m, 2H), 1.47 (d, J = 15.5 Hz, 9H), 1.39 (s, 9H).

^{13}C NMR (125 MHz, CDCl$_3$) δ 167.3, 167.2, 153.8, 153.7, 145.9, 145.7, 136.9, 136.7, 132.8, 130.8, 128.3, 127.9, 127.9, 116.7, 80.9, 80.9, 80.3, 80.3, 71.1, 70.8, 70.8, 50.2, 48.7, 48.6, 34.9, 34.4, 34.3, 34.0, 29.7, 28.4, 28.4, 28.1, 28.1.

$[\alpha]_D^{28}$ = -45.2 (c = 0.21, CH_2Cl_2).

HRMS (ESI) ($[M + Na]^+$) Calcd. for $[C_{24}H_{32}NaClNO_5]^+$: 472.1861, Found. 472.1861.

HRMS spectrum of **4d**

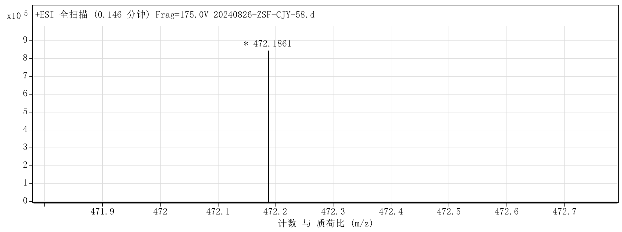

HPLC: INA column, hexane/^{i}PrOH = 97:3, 1.0 mL/min, t_R = 5.98 min (minor), 7.39 min (major).

Chiral HPLC spectra of (*rac*)-**4d** and **4d**

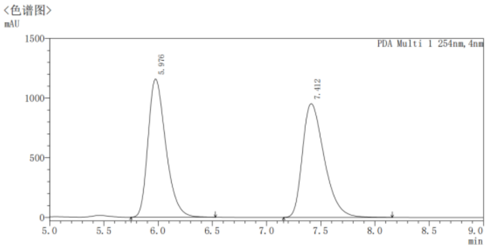

<峰表>
PDA Ch1 254nm

峰号	保留时间	面积	高度	高度%	面积%
1	5.976	13229422	1160239	54.856	49.764
2	7.412	13354825	954809	45.144	50.236
总计		26584247	2115048	100.000	100.000

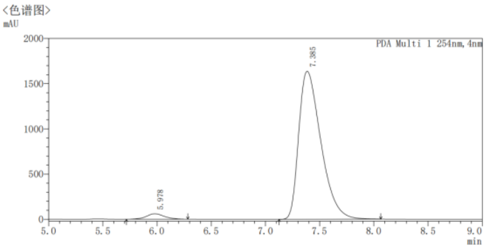

<峰表>
PDA Ch1 254nm

峰号	保留时间	面积	高度	高度%	面积%
1	5.978	701421	60535	3.565	2.928
2	7.385	23252665	1637516	96.435	97.072
总计		23954087	1698051	100.000	100.000

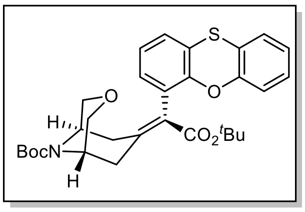

***tert*-butyl-*(1R,5S,E)*-7-(2-(tert-butoxy)-2-oxo-1-(phenoxathiin-4-yl)ethylidene)-3-oxa-9-azabicyclo[3.3.1]nonane-9-carboxylate (4e)**

48.8 mg, 91% yield, 97% ee, colorless oil, R_f = 0.47 (PE/EtOAc = 10:1).

^{1}H NMR (500 MHz, CDCl$_3$) δ 7.20 – 7.01 (m, 4H), 7.00 – 6.83 (m, 3H), 4.21 (d, J = 62.2 Hz, 1H), 4.00 (dd, J = 74.7, 19.1 Hz, 2H), 3.82 – 3.57 (m, 4H), 2.67 (s, 1H), 2.38 – 2.20 (m, 2H), 1.46 (d, J = 23.9 Hz, 9H), 1.32 (d, J = 5.6 Hz, 9H).

^{13}C NMR (125 MHz, CDCl$_3$) δ 166.6, 166.5, 153.8, 153.7, 152.3, 149.9, 129.6, 128.7, 128.6, 127.6, 126.5, 125.8, 124.5, 123.9, 120.6, 118.2, 80.4, 80.3, 80.2, 80.2, 71.2, 70.9, 50.3, 48.7, 48.7, 28.4, 28.4, 28.1, 28.1.

$[\alpha]_D^{28}$ = +32.0 (c = 0.39, CH_2Cl_2).

HRMS (ESI) ($[M + Na]^+$) Calcd. for $[C_{30}H_{35}NaNO_6S]^+$: 560.2077, Found. 560.2080.

HRMS spectrum of **4e**

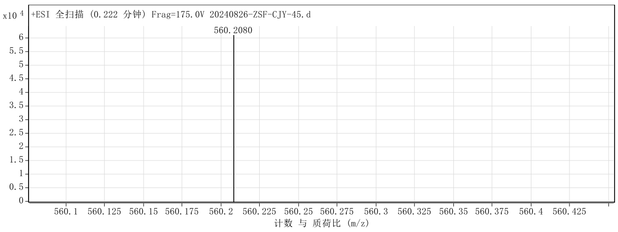

HPLC: INA column, hexane/^{i}PrOH = 97:3, 1.0 mL/min, t_R = 6.36 min (major), 7.13 min (minor).

Chiral HPLC spectra of (*rac*)-**4e** and **4e**

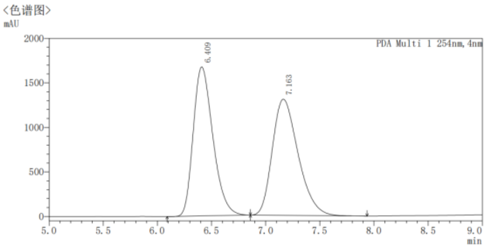

<峰表>
PDA Ch1 254nm

峰号	保留时间	面积	高度	高度%	面积%
1	6.409	21163165	1676828	56.246	49.430
2	7.163	21650960	1304398	43.754	50.570
总计		42814125	2981226	100.000	100.000

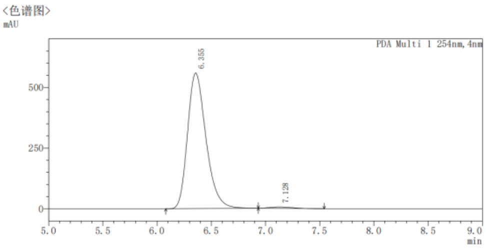

<峰表>
PDA Ch1 254nm

峰号	保留时间	面积	高度	高度%	面积%
1	6.355	6616498	559013	98.942	98.799
2	7.128	80402	5980	1.058	1.201
总计		6696900	564993	100.000	100.000

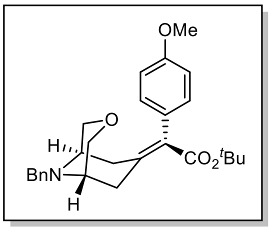

***tert*-butyl-*(E)*-2-(*(1R,5S)*-9-benzyl-3-oxa-9-azabicyclo[3.3.1]nonan-7-ylidene)-2-(4-methoxyphenyl)acetate (4f)**

14.4 mg, 33% yield, 96% ee, colorless oil, R_f = 0.32 (PE/EtOAc = 10:1).

^{1}H NMR (500 MHz, CDCl$_3$) δ 7.40 (d, J = 7.0 Hz, 2H), 7.34 (t, J = 7.4 Hz, 2H), 7.28 (d, J = 6.6 Hz, 1H), 7.16 (d, J = 8.6 Hz, 2H), 6.88 (d, J = 8.6 Hz, 2H), 3.97 – 3.86 (m, 1H), 3.91 (d, J = 4.2 Hz, 2H), 3.86 – 3.81 (m, 2H), 3.83 (s, 3H), 3.62 (d, J = 10.8 Hz, 1H), 3.14 (d, J = 14.6 Hz, 1H), 2.85 (dd, J = 16.1 Hz, 2H), 2.72 (d, J = 5.3 Hz, 1H), 2.52 (dd, J = 15.5, 5.2 Hz, 1H), 2.21 (d, J = 15.7 Hz, 1H), 1.44 (s, 9H).

^{13}C NMR (125 MHz, CDCl$_3$) δ 168.5, 158.3, 146.5, 138.7, 130.8, 130.6, 128.7, 128.7, 128.4, 128.3, 127.2, 127.1, 113.4, 80.3, 71.8, 71.7, 56.5, 55.2, 54.3, 54.1, 29.5, 29.3, 28.2, 28.1.

$[\alpha]_D^{28}$ = -17.9 (c = 0.12, CH_2Cl_2).

HRMS (ESI) ($[M + H]^+$) Calcd. for $[C_{27}H_{34}NO_4]^+$: 436.2483, Found. 436.2493.

HRMS spectrum of **4f**

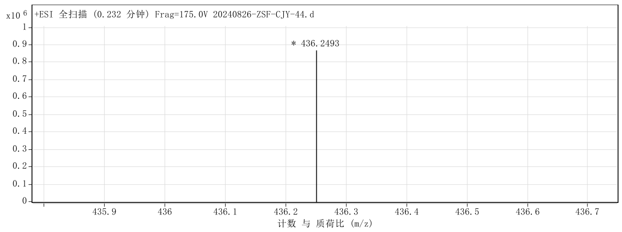

HPLC: AD-H column, hexane/^{i}PrOH = 97:3, 1.0 mL/min, t_R = 12.72 min (minor), 21.51 min (major).

Chiral HPLC spectra of (*rac*)-**4f** and **4f**

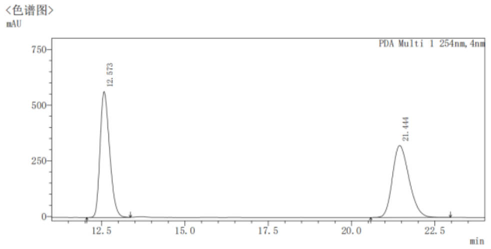

<峰表>
PDA Ch1 254nm

峰号	保留时间	面积	高度	高度%	面积%
1	12.573	11242228	565563	63.640	50.078
2	21.444	11207344	323126	36.360	49.922
总计		22449572	888689	100.000	100.000

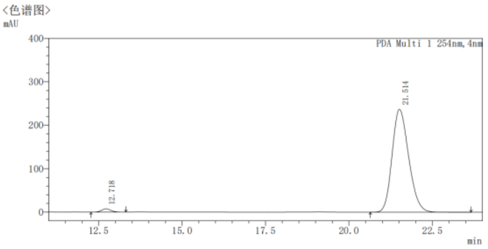

<峰表>
PDA Ch1 254nm

峰号	保留时间	面积	高度	高度%	面积%
1	12.718	150400	7701	3.149	1.806
2	21.514	8178440	236837	96.851	98.194
总计		8328840	244537	100.000	100.000

5. The gram-scale synthesis of products 3au and 3aw.

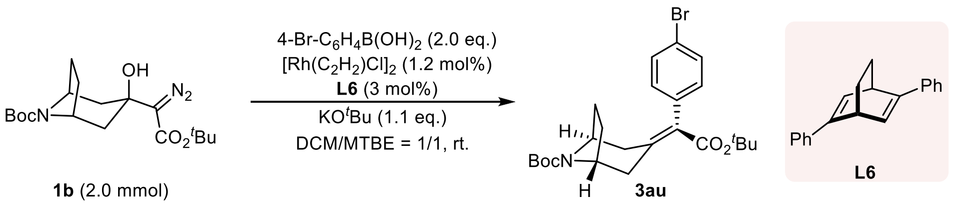

Under nitrogen atmosphere, to a 10 mL Schlenk tube was added $[Rh(C_2H_4)_2Cl]_2$ (9.3 mg, 1.2 mol%), **L6** (15.5 mg, 3 mol%), and then 2 mL DCM was added. The mixture was stirred for 1 h to prepare the $[Rh(\mathbf{L6})Cl]_2$ in situ. Then, $[Rh(\mathbf{L6})Cl]_2$ was added to a solution of KO^{t}Bu (246.4 mg, 1.1 eq.) and 4-bromophenylboronic acid (800 mg, 2.0 eq.) in DCM/MTBE (0.05 M). Then, the α-diazoester **1b** (2.0 mmol dissolved in another 10 mL DCM) was introduced in one portion into the above system. The resulting mixture was stirred at room temperature for 12 h. After the reaction was completed, the reaction mixture was filtered through short silica gel, and then the solvent was removed under reduced pressure. The crude product was purified by flash column chromatography on silica gel with EtOAc–petroleum ether to yield the product **3au** (93% yield, 98% ee).

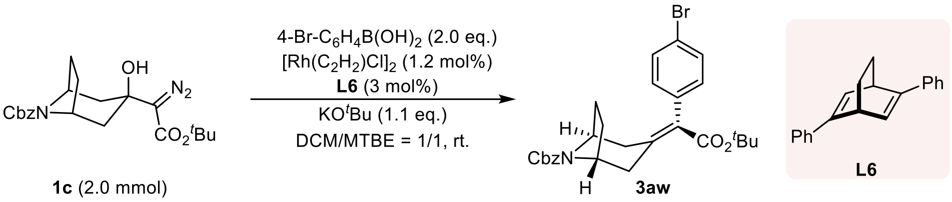

1c (2.0 mmol) **3aw**

Under nitrogen atmosphere, to a 10 mL Schlenk tube was added $[Rh(C_2H_4)_2Cl]_2$ ((9.3 mg, 1.2 mol%), **L6** (15.5 mg, 3 mol%), and then 2 mL DCM was added. The mixture was stirred for 1 h to prepare the [Rh(**L6**)Cl]$_2$ in situ. Then, [Rh(**L6**)Cl]$_2$ was added to a solution of KO*^{t}*Bu (246.4 mg, 1.1 eq.) and 4-bromophenylboronic acid (800 mg, 2.0 eq.) in DCM/MTBE (0.05 M). Then, the α-diazoester **1c** (2.0 mmol dissolved in another 10 mL DCM) was introduced in one portion into the above system. The resulting mixture was stirred at room temperature for 12 h. After the reaction was completed, the reaction mixture was filtered through short silica gel, and then the solvent was removed under reduced pressure. The crude product was purified by flash column chromatography on silica gel with EtOAc–petroleum ether to yield the product **3aw** (93% yield, 97% ee).

6. The procedure for the synthetic applications.

The procedure for the synthesis of **5**:

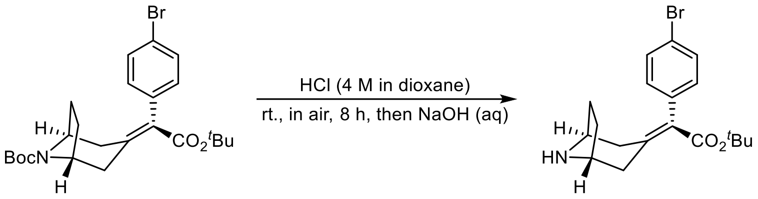

3au **5**

To the solution of **3au** (0.35 mmol) in dioxane (3 mL), 4 M HCl in dioxane (1 mL) was added. The resulting mixture was stirred at room temperature for 8 h. The reaction mixture was diluted with water and the pH was adjusted to 14 using 5 M NaOH. The aqueous layer was extracted with EtOAc. The combined organic phases were washed with brine, dried over Na_2SO_4, and then the solvent was removed under reduced pressure to give the product **5**.

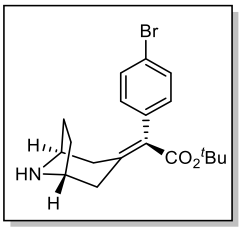

***tert*-butyl-*(Z)*-2-(*(1R,5S)*-8-azabicyclo[3.2.1]octan-3-ylidene)-2-(4-bromophenyl)acetate (5)**

44.9 mg, 44% yield, colorless solid, R_f = 0.22 (EtOAc), M.P. = 178.1 - 178.9 °C.

^{1}H NMR (400 MHz, CDCl$_3$) δ 7.51 – 7.43 (m, 2H), 7.11 – 7.03 (m, 2H), 3.72 (d, *J* = 3.5 Hz, 1H), 3.66 (s, 1H), 3.56 (dt, *J* = 6.5, 3.2 Hz, 1H), 2.88 (dt, *J* = 14.6, 2.4 Hz, 1H), 2.44 (dd, *J* = 14.7, 3.3 Hz, 1H), 2.24 (dd, *J* = 14.8, 3.3 Hz, 1H), 2.14 (dt, *J* = 14.6, 2.3 Hz, 1H), 1.88 – 1.71 (m, 3H), 1.54 – 1.45 (m, 1H), 1.43 (s, 9H).

^{13}C NMR (100 MHz, CDCl$_3$) δ 167.6, 140.7, 136.1, 133.0, 131.4, 130.9, 121.2, 81.3, 55.2, 39.0, 38.8, 29.1, 28.8, 28.1.

$[\alpha]_D^{28}$ = -15.8 (c = 0.12, H_2O).

HRMS (ES-TOF) ($[M + H]^+$) Calcd. for [$C_{19}H_{25}BrNO_2$]$^+$: 378.1063, Found. 378.1067.

HRMS spectrum of **5**

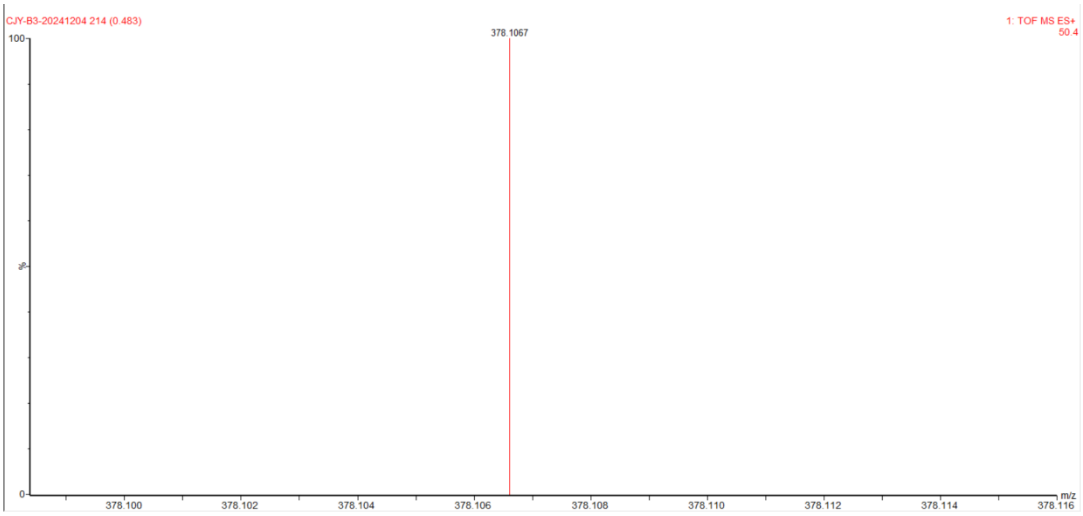

The procedure for the synthesis of **6**:

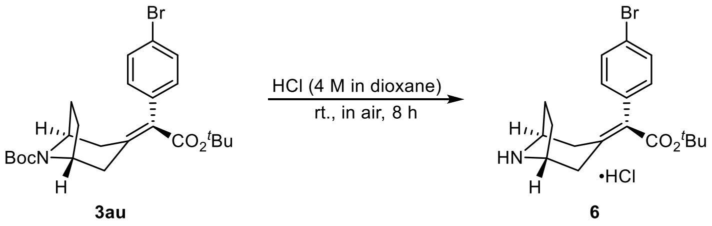

To the solution of **3au** (0.31 mmol) in dioxane (3 mL), 4 M HCl in dioxane (1 mL) was added. The resulting mixture was stirred at room temperature for 8 h. The mixture was concentrated under reduced pressure to give the product **6**.

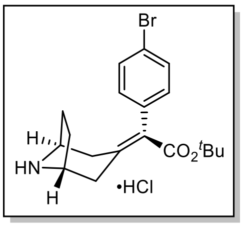

***tert*-butyl-*(Z)*-2-(*(1R,5S)*-8-azabicyclo[3.2.1]octan-3-ylidene)-2-(4-bromophenyl)acetate hydrochloride (6)**

115.0 mg, 90% yield, colorless solid, R_f = 0.19 (EtOAc), M.P. = 258.1 - 258.9 °C.

^{1}H NMR (400 MHz, $CDCl_3$) δ 7.51 – 7.43 (m, 2H), 7.11 – 7.03 (m, 2H), 3.72 (d, *J* = 3.5 Hz, 1H), 3.66 (s, 1H), 3.56 (dt, *J* = 6.5, 3.2 Hz, 1H), 2.88 (dt, *J* = 14.6, 2.4 Hz, 1H), 2.44 (dd, *J* = 14.7, 3.3 Hz, 1H), 2.24 (dd, *J* = 14.8, 3.3 Hz, 1H), 2.14 (dt, *J* = 14.6, 2.3 Hz, 1H), 1.88 – 1.71 (m, 3H), 1.54 – 1.45 (m, 1H), 1.43 (s, 9H).

^{13}C NMR (100 MHz, $CDCl_3$) δ 167.6, 140.7, 136.1, 133.0, 131.4, 130.9, 121.2, 81.3, 55.2, 39.0, 38.8, 29.1, 28.8, 28.1.

$[\alpha]_D^{28}$ = -15.0 (c = 0.10, H_2O).

The procedure for the synthesis of **7**:

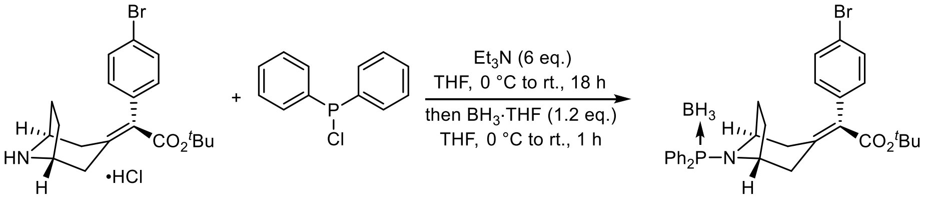

To an ice-cooled solution of **6** (0.1 mmol) in THF (1 mL), Et_3N (6 eq.) and chlorodiphenylphosphane (1.2 eq.) were added. After 30 min, the mixture was warmed to room temperature and stirred for 18 h. The mixture was filtered through short silica gel, and then the solvent was removed under reduced pressure. The residue was dissolved in THF (1 mL). After cooling to 0 °C, BH_3·THF (1.2 eq.) was added and resulting solution was stirred for 1 h at room temperature. The mixture was concentrated under reduced pressure. The crude product was purified by flash column chromatography to give the product **7** (eluent: PE/EtOAc = 3:1).

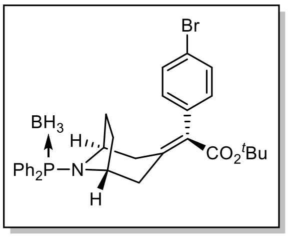

Compound 7: 15.0 mg, 26% yield, 97% ee, colorless oil, R_f = 0.18 (PE/EtOAc = 3:1).

^{1}H NMR (400 MHz, CDCl$_3$) δ 8.03 – 7.89 (m, 4H), 7.61 – 7.39 (m, 8H), 7.07 (d, J = 8.1 Hz, 2H), 3.86 (s, 1H), 3.69 (s, 1H), 2.89 – 2.68 (m, 2H), 2.50 (d, J = 13.7 Hz, 1H), 2.23 – 1.87 (m, 5H), 1.82 (t, 1H), 1.62 – 1.48 (m, 1H), 1.42 (s, 9H), 1.27 (s, 1H).

^{13}C NMR (100 MHz, CDCl$_3$) δ 167.5, 139.9, 135.9, 133.9, 132.8, 132.3, 132.3, 132.2, 132.2, 131.8, 131.4, 130.8, 128.7, 128.6, 121.3, 81.4, 56.0, 56.0, 39.1, 39.1, 38.9, 38.8, 30.3, 30.3, 30.0, 30.0, 29.7, 28.1.

^{31}P NMR (162 MHz, CDCl$_3$) δ 23.7.

$[\alpha]_D^{28}$ = -129.6 (c = 0.15, CH_2Cl_2).

HRMS (ESI) ($[M + H]^+$) Calcd. for $[C_{31}H_{36}BBrNO_2P]^+$: 576.1833, Found. 576.1828.

HRMS spectrum of **7**

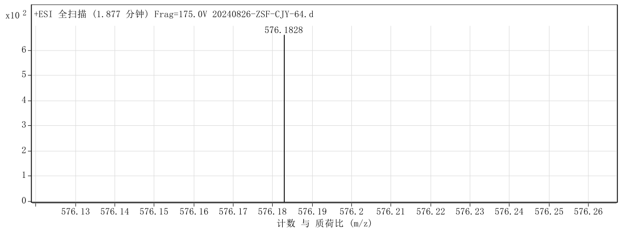

HPLC: INA column, hexane/^{i}PrOH = 80:20, 1.0 mL/min, t_R = 5.67 min (major), 7.24 min (minor).

Chiral HPLC spectra of (*rac*)-**7** and **7**

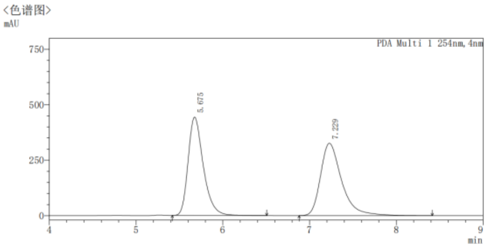

<峰表>
PDA Ch1 254nm

峰号	保留时间	面积	高度	高度%	面积%
1	5.675	5387871	443808	57.641	49.439
2	7.229	5510242	326139	42.359	50.561
总计		10898113	769947	100.000	100.000

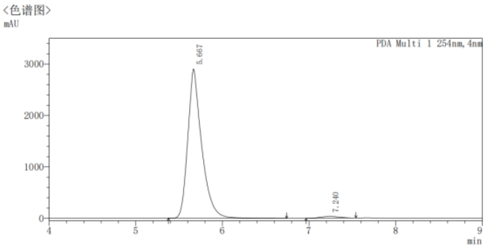

<峰表>
PDA Ch1 254nm

峰号	保留时间	面积	高度	高度%	面积%
1	5.667	32254974	2905633	98.871	98.542
2	7.240	477140	33173	1.129	1.458
总计		32732115	2938805	100.000	100.000

The procedure for the synthesis of **8**:

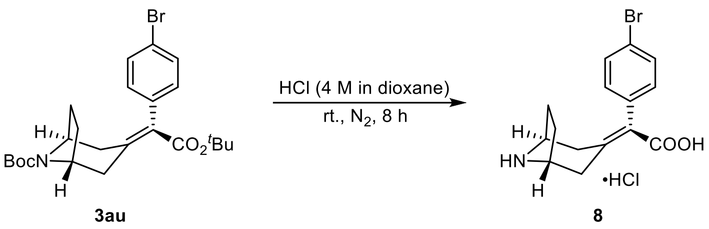

Under nitrogen atmosphere, to the solution of **3au** (1.1 mmol) in dioxane (5 mL), 4 M HCl in dioxane (2.5 mL) was added. The resulting mixture was stirred at room temperature for 8 h. The mixture was concentrated under reduced pressure. The crude product was washed by DCM (20 mL) to give the product **8**.

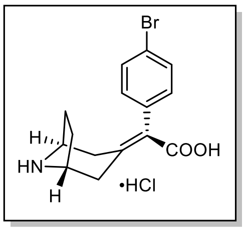

***(Z)*-2-(*(1R,5S)*-8-azabicyclo[3.2.1]octan-3-ylidene)-2-(4-bromophenyl)acetic acid hydrochloride (8)**

232.2 mg, 65% yield, colorless solid, R_f = 0.18 (EtOAc), M.P. = 251.8 - 253.3 °C.

^{1}H NMR (500 MHz, DMSO-*d*$_6$) δ 9.74 (d, *J* = 8.8 Hz, 1H), 9.54 (s, 1H), 7.57 (d, *J* = 8.4 Hz, 2H), 7.14 (d, *J* = 8.4 Hz, 2H), 4.08 (d, *J* = 3.4 Hz, 1H), 3.91 (d, *J* = 3.5 Hz, 1H), 3.08 (d, *J* = 15.4 Hz, 1H), 2.79 (d, *J* = 15.6 Hz, 1H), 2.63 (d, *J* = 15.6 Hz, 1H), 2.07 (d, *J* = 15.3 Hz, 1H), 2.04 – 1.83 (m, 2H), 1.76 (t, *J* = 11.3 Hz, 1H), 1.50 (t, *J* = 9.9 Hz, 1H).

^{13}C NMR (125 MHz, DMSO-*d*$_6$) δ 168.8, 138.2, 136.2, 134.5, 131.9, 131.7, 121.3, 54.7, 54.6, 35.0, 34.9, 26.8, 26.4.

$[\alpha]_D^{28}$ = -23.0 (c = 0.10, H_2O).

The procedure for the synthesis of **9**:[3]

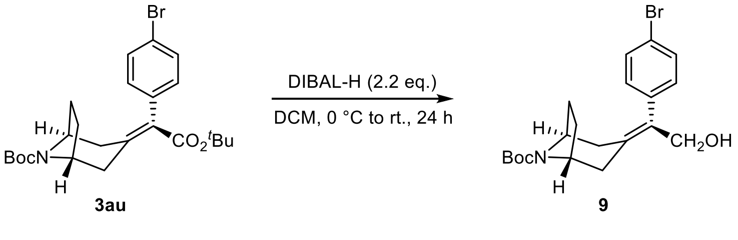

To a solution of **3au** (1.0 mmol) in DCM (2 mL), DIBAL-H (2.2 equiv., 1 M in hexane) was added dropwise at 0 °C. The resulting solution was warmed to room temperature and stirred for 24 h. After the reaction was completed, the mixture was quenched by the slow addition of water. The aqueous layer was

extracted with EtOAc. The combined organic phases were washed with brine, dried over Na_2SO_4, and concentrated under reduced pressure. The resulting crude product was purified by flash column chromatography to give the product **9** (eluent: PE/EtOAc = 3:1).

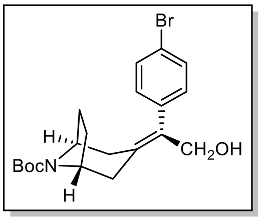

***tert*-butyl-*(1R,5S,Z)*-3-(1-(4-bromophenyl)-2-hydroxyethylidene)-8-azabicyclo[3.2.1]octane-8-carboxylate (9)**

337.8 mg, 83% yield, 96% ee, colorless oil, R_f = 0.35 (PE/EtOAc = 3:1).

^{1}H NMR (500 MHz, $CDCl_3$) δ 7.48 – 7.35 (m, 2H), 6.99 (d, *J* = 7.9 Hz, 2H), 4.48 – 4.20 (m, 3H), 4.05 (dd, *J* = 23.1, 7.6 Hz, 1H), 2.62 (s, 1H), 2.37 (dd, *J* = 33.9, 14.0 Hz, 1H), 2.26 – 2.09 (m, 2H), 2.06 – 1.97 (m, 1H), 1.95 – 1.76 (m, 2H), 1.60 (t, *J* = 9.4 Hz, 1H), 1.43 (s, 9H).

^{13}C NMR (125 MHz, $CDCl_3$) δ 153.5, 140.2, 137.3, 133.6, 131.5, 130.7, 120.7, 79.5, 62.2, 54.2, 53.5, 38.0, 37.3, 35.9, 35.2, 28.6, 28.5, 28.0.

$[\alpha]_D^{28}$ = -63.1 (c = 0.74, CH_2Cl_2).

HRMS (ESI) ($[M + Na]^+$) Calcd. for $[C_{23}H_{24}NaBrNO_3]^+$: 464.0832, Found. 464.0841.

HRMS spectrum of **9**

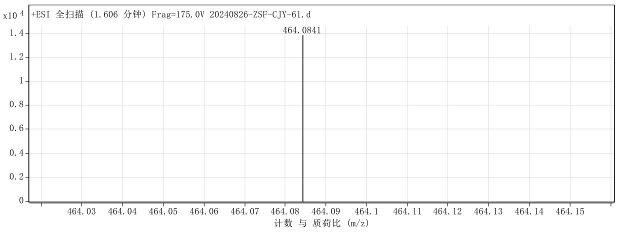

HPLC: AD-H column, hexane/^{i}PrOH = 90:10, 1.0 mL/min, t_R = 7.33 min (minor), 8.05 min (major).

Chiral HPLC spectra of (*rac*)-**9** and **9**

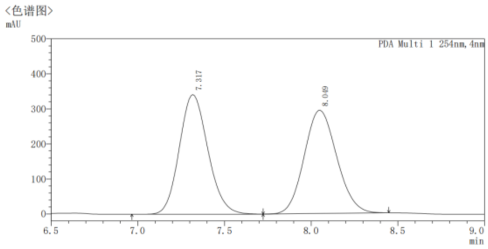

<峰表>
PDA Ch1 254nm

峰号	保留时间	面积	高度	高度%	面积%
1	7.317	3919313	341645	53.684	50.388
2	8.049	3859014	294755	46.316	49.612
总计		7778327	636400	100.000	100.000

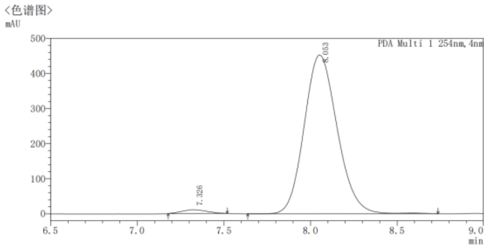

<峰表>
PDA Ch1 254nm

峰号	保留时间	面积	高度	高度%	面积%
1	7.326	105776	10424	2.249	1.731
2	8.053	6004746	453016	97.751	98.269
总计		6110522	463440	100.000	100.000

The procedure for the synthesis of **10**:

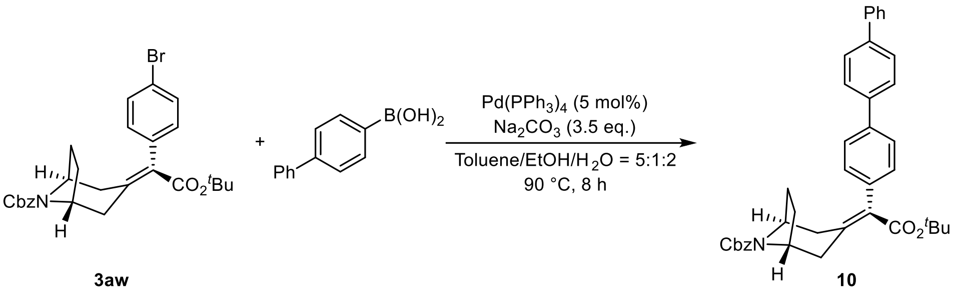

Under nitrogen atmosphere, add **3aw** (0.1 mmol), boric acid (2.0 eq.), $Pd(PPh_3)_4$ (5.0 mol%), Na_2CO_3 (3.5 eq.) and mixed toluene/EtOH/H_2O (5:1:2) to a 25 mL round-bottom flask equipped with condenser. The mixture was stirred at 90 °C for 8 h. After the reaction was completed, the mixture was quenched with H_2O and extracted with EtOAc. The combined organic phases were washed with brine, dried over Na_2SO_4, and concentrated under reduced pressure. The crude product was purified by flash column chromatography to give the product **10** (eluent: PE/EtOAc = 10:1).

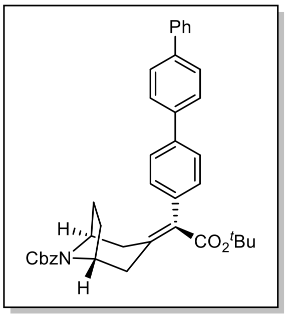

benzyl-*(1R,5S,Z)*-3-(1-([1,1':4',1''-terphenyl]-4-yl)-2-(tert-butoxy)-2-oxoethylidene)-8-azabicyclo[3.2.1]octane-8-carboxylate (10)

53.8 mg, 91% yield, 96% ee, colorless oil, R_f = 0.41 (PE/EtOAc = 10:1).

^{1}H NMR (400 MHz, $CDCl_3$) δ 7.72 (s, 4H), 7.68 (d, 2H), 7.66 (d, *J* = 8.0 Hz, 2H), 7.50 (t, *J* = 7.6 Hz, 2H), 7.45 – 7.37 (m, 6H), 7.33 (d, *J* = 7.9 Hz, 2H), 5.22 (s, 2H), 4.49 (d, *J* = 18.1 Hz, 1H), 4.33 (d, *J* = 19.5 Hz, 1H), 2.87 (dd, *J* = 13.9, 2.1 Hz, 1H), 2.61 (dd, *J* = 44.8, 16.6 Hz, 1H), 2.49 – 2.29 (m, 2H), 2.07 – 1.84 (m, 3H), 1.61 (t, *J* = 10.8 Hz, 1H), 1.51 (s, 9H).

^{13}C NMR (100 MHz, $CDCl_3$) δ 168.2, 153.7, 140.7, 140.2, 139.6, 139.5, 137.7, 136.9, 135.9, 135.3, 129.5, 128.9, 128.6, 128.0, 127.9, 127.5, 127.4, 127.4, 127.1, 126.9, 81.5, 66.8, 54.2, 38.3, 38.2, 37.5, 37.0, 28.9, 28.5, 28.2, 27.7, 27.6.

$[\alpha]_D^{28}$ = -65.7 (c = 0.46, CH_2Cl_2).

HRMS (ESI) ($[M + Na]^+$) Calcd. for $[C_{39}H_{37}NaNO_3]^+$: 590.2665, Found. 590.2662.

HRMS spectrum of **10**

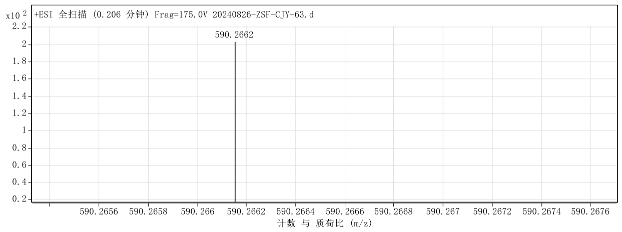

HPLC: AD-H column, hexane/^{i}PrOH = 95:5, 1.0 mL/min, t_R = 30.93 min (minor), 35.09 min (major).

Chiral HPLC spectra of (*rac*)-**10** and **10**

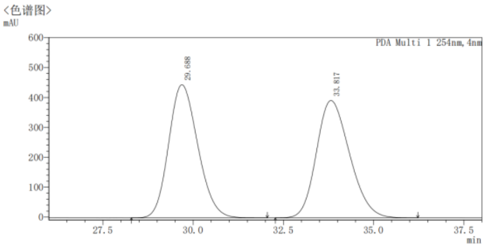

<峰表>
PDA Ch1 254nm

峰号	保留时间	面积	高度	高度%	面积%
1	29.688	25047758	445195	53.160	49.952
2	33.817	25095945	392269	46.840	50.048
总计		50143702	837464	100.000	100.000

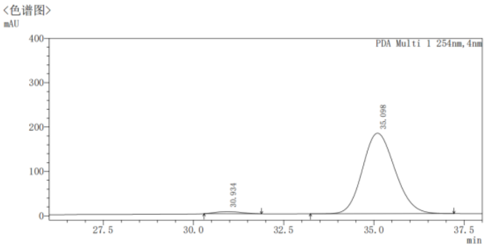

<峰表>
PDA Ch1 254nm

峰号	保留时间	面积	高度	高度%	面积%
1	30.934	238425	5017	2.691	2.055
2	35.098	11365023	181439	97.309	97.945
总计		11603448	186456	100.000	100.000

The procedure for the synthesis of **11**:

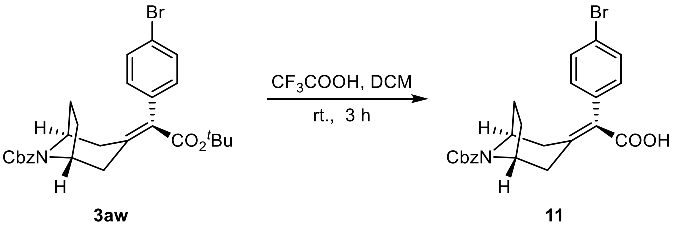

To a solution of **3aw** (0.63 mmol) in DCM (15 mL), 7.3 mL CF_3COOH was added in one portion. The resulting mixture was stirred at room temperature for 3 h. The mixture was concentrated under reduced pressure. The resulting crude product was purified by flash column chromatography to give the product **11** (eluent: PE/EtOAc = 1:1).

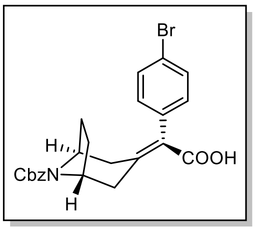

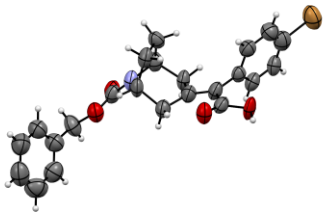

***(Z)*-2-(*(1R,5S)*-8-((benzyloxy)carbonyl)-8-azabicyclo[3.2.1]octan-3-ylidene)-2-(4-bromophenyl)acetic acid (11)**

257.8 mg, 90% yield, colorless solid, R_f = 0.29 (PE/EtOAc = 1:1), M.P. = 164.4 - 165.7 °C.

¹H NMR (500 MHz, CDCl₃) δ 10.01 (s, 1H), 7.48 (d, *J* = 8.0 Hz, 2H), 7.41 – 7.30 (m, 5H), 7.04 (d, *J* = 8.0 Hz, 2H), 5.17 (s, 2H), 4.44 (d, *J* = 24.4 Hz, 1H), 4.28 (d, *J* = 16.5 Hz, 1H), 3.29 (d, *J* = 15.2 Hz, 1H), 2.65 – 2.46 (m, 1H), 2.32 (dd, *J* = 61.8, 12.9 Hz, 1H), 2.13 (d, *J* = 15.0 Hz, 1H), 2.02 – 1.83 (m, 2H), 1.75 (t, *J* = 9.2 Hz, 1H), 1.47 (t, *J* = 11.1 Hz, 1H).

^{13}C NMR (125 MHz, $CDCl_3$) δ 172.3, 153.7, 147.8, 147.5, 139.5, 136.6, 135.8, 132.0, 131.7, 131.1, 129.9, 128.7, 128.6, 128.1, 127.9, 127.0, 121.8, 67.0, 53.9, 39.0, 38.2, 37.5, 29.7, 29.5, 28.9, 28.6, 28.1, 27.9.

$[\alpha]_D^{28}$ = -63.0 (c = 0.10, CH_2Cl_2).

The procedure for the synthesis of **12**:

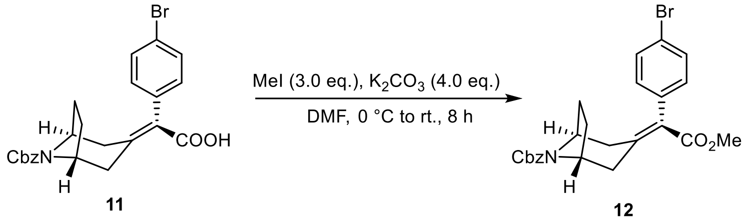

To a mixture of **11** (0.044 mmol) and K_2CO_3 (3.0 eq.) in DMF (1 mL), MeI (4.0 eq.) was added dropwise at 0 °C. The resulting mixture was stirred at room temperature for 8 h and then quenched with H_2O and extracted with DCM. The combined organic phases were washed with brine, dried over Na_2SO_4, and concentrated under reduced pressure. The resulting crude product was purified by flash column chromatography to give the product **12** (eluent: PE/EtOAc = 10:1).

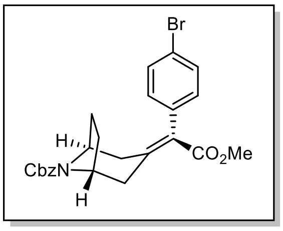

benzyl-*(1R,5S,Z)*-3-(1-(4-bromophenyl)-2-methoxy-2-oxoethylidene)-8-azabicyclo[3.2.1]octane-8-carboxylate (12)

21.1 mg, 99% yield, 98% ee, colorless oil, R_f = 0.51 (PE/EtOAc = 10:1).

¹H NMR (400 MHz, CDCl₃) δ 7.52 – 7.43 (m, 2H), 7.41 – 7.28 (m, 5H), 7.03 (d, *J* = 8.4 Hz, 2H), 5.16 (s, 2H), 4.35 (d, *J* = 63.6 Hz, 2H), 3.67 (s, 3H), 3.01 (d, *J* = 14.9 Hz, 1H), 2.69 – 2.44 (m, 1H), 2.39 – 2.21 (m, 1H), 2.13 (d, *J* = 14.5 Hz, 1H), 2.01 – 1.83 (m, 2H), 1.82 – 1.72 (m, 1H), 1.56 – 1.40 (m, 1H).

^{13}C NMR (125 MHz, CDCl$_3$) δ 168.1, 153.6, 143.8, 136.8, 135.8, 132.5, 131.6, 130.9, 128.5, 128.0, 127.9, 121.7, 66.9, 53.9, 52.0, 38.2, 37.5, 29.7, 28.9, 28.6, 28.1, 27.8.

$[\alpha]_D^{28}$ = -131.7 (c = 0.16, CH_2Cl_2).

HRMS (ESI) ($[M + Na]^+$) Calcd. for $[C_{24}H_{24}NaBrNO_4]^+$: 492.0781, Found. 492.0781.

HRMS spectrum of **12**

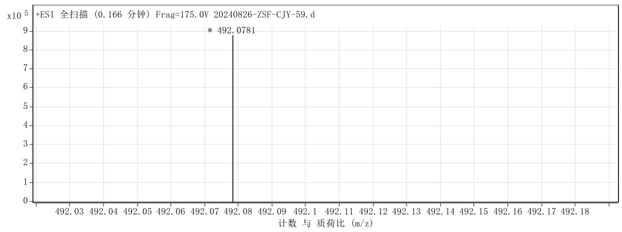

HPLC: INA column, hexane/^{i}PrOH = 97:3, 1.0 mL/min, t_R = 18.76 min (minor), 23.72 min (major).

Chiral HPLC spectra of (*rac*)-**12** and **12**

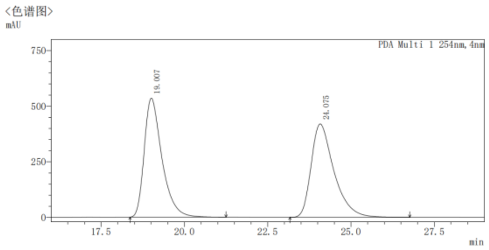

<峰表>
PDA Ch1 254nm

峰号	保留时间	面积	高度	高度%	面积%
1	19.007	19786563	535230	56.048	49.760
2	24.075	19977358	419727	43.952	50.240
总计		39763921	954957	100.000	100.000

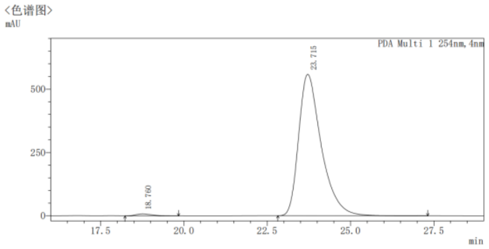

<峰表>
PDA Ch1 254nm

峰号	保留时间	面积	高度	高度%	面积%
1	18.760	276127	7893	1.395	1.038
2	23.715	26336737	557964	98.605	98.962
总计		26612864	565857	100.000	100.000

The procedure for the synthesis of **13**:[4]

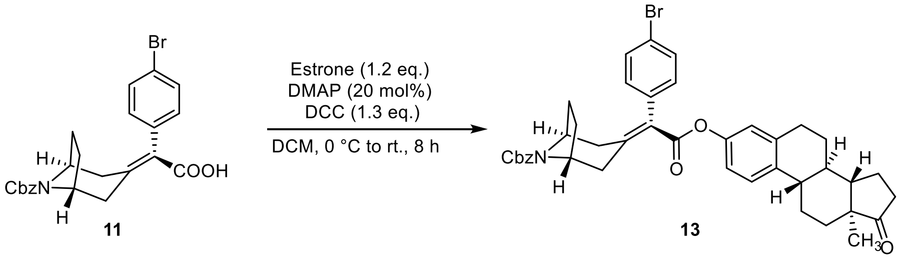

To a 25 mL shlenk tube was added **11** (0.05 mmol), Estrone (1.2 eq.), DMAP (20 mol%) and 1 mL DCM. Then, DCC (0.065 mmol dissolved in 0.05 mL DCM) was added at 0 °C. The resulting solution was warmed to room temperature and stirred for 8 h. The reaction mixture was filtered through short silica gel, and then the solvent was removed under reduced pressure. The crude product was purified by flash column chromatography to give the product **13** (eluent: PE/EtOAc = 3:1).

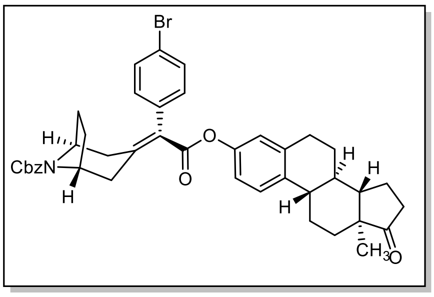

benzyl-*(1R,5S,Z)*-3-(1-(4-bromophenyl)-2-(((*8R,9S,13S,14S*)-13-methyl-17-oxo-7,8,9,11,12,13,14,15,16,17-decahydro-6*H*-cyclopenta[*a*]phenanthren-3-yl)oxy)-2-oxoethylidene)-8-azabicyclo[3.2.1]octane-8-carboxylate (13)

32.0 mg, 91% yield, >20:1 dr, colorless oil, R_f = 0.57 (PE/EtOAc = 3:1).

^{1}H NMR (400 MHz, $CDCl_3$) δ 7.52 (d, *J* = 8.4 Hz, 2H), 7.43 – 7.29 (m, 5H), 7.21 – 7.04 (m, 2H), 6.78 (dd, *J* = 8.5, 2.5 Hz, 1H), 6.73 (d, *J* = 2.6 Hz, 1H), 6.68 – 6.56 (m, 1H), 5.17 (s, 2H), 4.39 (d, *J* = 61.8 Hz, 2H), 3.19 (d, *J* = 15.1 Hz, 1H), 2.98 – 2.78 (m, 3H), 2.50 (dd, *J* = 18.9, 8.6 Hz, 1H), 2.43 – 2.35 (m, 1H), 2.41 – 2.32 (m, 2H), 2.31 – 2.20 (m, 2H), 2.15 (q, *J* = 9.7, 9.2 Hz, 2H), 2.07 – 1.97 (m, 3H), 1.82 (dd, *J* = 15.1, 5.9 Hz, 1H), 1.74 – 1.40 (m, 7H), 0.90 (s, 3H).

^{13}C NMR (125 MHz, $CDCl_3$) δ 166.3, 154.0, 153.7, 148.4, 138.1, 137.9, 137.6, 136.7, 135.5, 132.1, 132.0, 131.8, 131.7, 131.6, 131.0, 128.6, 128.6, 128.1, 127.9, 126.4, 126.4, 121.9, 121.3, 118.5, 115.4, 112.9, 67.0, 54.0, 53.9, 50.4, 48.1, 48.0, 44.1, 44.0, 38.4, 38.0, 35.9, 35.9, 33.9, 31.6, 31.5, 29.7, 29.5, 29.4, 26.6, 26.3, 26.0, 25.8, 25.6, 24.9, 21.6, 21.6, 21.1, 13.9, 13.8.

$[\alpha]_D^{28}$ = +49.5 (c = 0.22, CH_2Cl_2).

HRMS (ESI) ([M + Na]$^+$) Calcd. for $[C_{41}H_{42}NaBrNO_5]^+$: 730.2138, Found. 730.2126.

HRMS spectrum of **13**

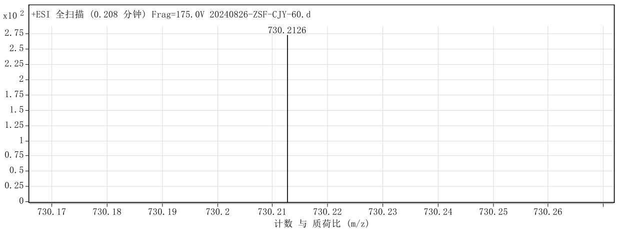

The procedure for the synthesis of **14**:

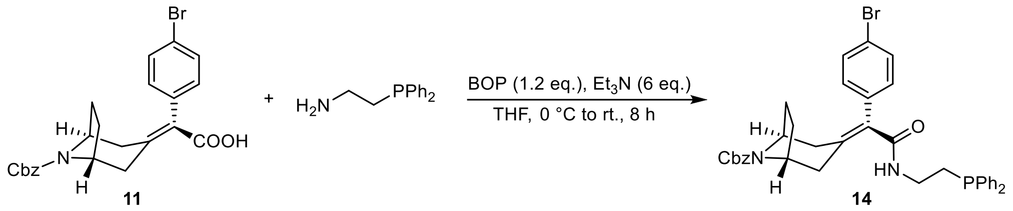

To a mixture of **11** (1.2 eq.), BOP (1.2 eq.) and Et_3N (6.0 eq.) in THF (3 mL), 2-(diphenylphosphanyl)ethan-1-amine (0.1 mmol, dissolved in 1 mL THF) was added the mixture at 0 °C. The resulting solution was warmed to room temperature and stirred for 8 h. After the reaction was completed, the mixture was quenched with saturated aqueous solution of $NaHCO_3$ and extracted with EtOAc. The combined organic phases were washed with brine, dried over Na_2SO_4, and concentrated under reduced pressure. The resulting crude product was purified by flash column chromatography to give the product **14** (eluent: PE/EtOAc = 5:1).

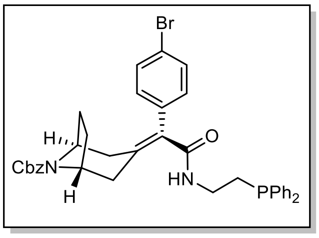

benzyl-*(1R,5S,Z)*-3-(1-(4-bromophenyl)-2-((2-(diphenylphosphanyl)ethyl)amino)-2-oxoethylidene)-8-azabicyclo[3.2.1]octane-8-carboxylate (14)

51.9 mg, 78% yield, 98% ee, colorless oil, R_f = 0.38 (PE/EtOAc = 5:1).

^{1}H NMR (500 MHz, $CDCl_3$) δ 7.57 (t, 2H), 7.56 – 7.52 (m, 1H), 7.49 – 7.41 (m, 2H), 7.40 – 7.35 (m, 5H), 7.31 – 7.26 (m, 5H), 7.25 – 7.21 (m, 2H), 6.98 (dd, *J* = 22.4, 8.1 Hz, 2H), 6.90 – 6.81 (m, 1H), 5.08 (s, 2H), 4.29 (d, *J* = 27.8 Hz, 1H), 4.17 (d, *J* = 18.9 Hz, 1H), 3.62 – 3.44 (m, 2H), 2.63 (d, *J* = 14.6 Hz, 1H), 2.38 (dt, *J* = 12.5, 6.6 Hz, 2H), 2.16 (t, *J* = 7.3 Hz, 1H), 2.03 (d, *J* = 14.4 Hz, 1H), 1.86 – 1.72 (m, 3H), 1.46 – 1.33 (m, 1H).

^{13}C NMR (125 MHz, $CDCl_3$) δ 169.0, 168.4, 153.6, 136.9, 135.8, 135.8, 132.8, 132.7, 132.6, 132.6, 132.5, 132.2, 132.2, 131.9, 131.9, 131.8, 131.7, 130.7, 130.7, 130.6, 130.6, 130.6, 130.5, 129.0, 128.9, 128.9, 128.9, 128.8, 128.6, 128.6, 128.5, 128.0, 127.9, 121.8, 66.8, 66.8, 54.1, 54.0, 38.0, 37.4, 37.1, 36.9, 33.9, 33.9, 29.7, 29.2, 28.9, 28.6, 28.5.

^{31}P NMR (162 MHz, $CDCl_3$) δ 33.3, 33.1.

$[\alpha]_D^{28}$ = -58.0 (c = 0.46, CH_2Cl_2).

HRMS (ESI) ($[M + Na]^+$) Calcd. for $[C_{37}H_{36}NaBrN_2O_3P]^+$: 689.1539, Found. 689.1536.

HRMS spectrum of **14**

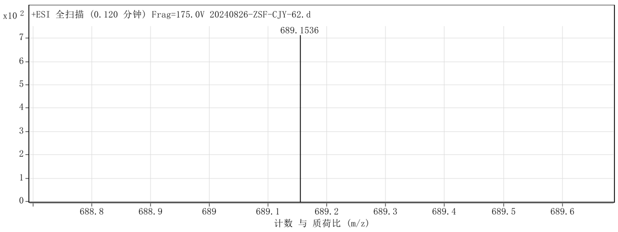

HPLC: INA column, hexane/^{i}PrOH = 85:15, 1.0 mL/min, t_R = 29.39 min (minor), 36.23 min (major).

Chiral HPLC spectra of (*rac*)-**14** and **14**

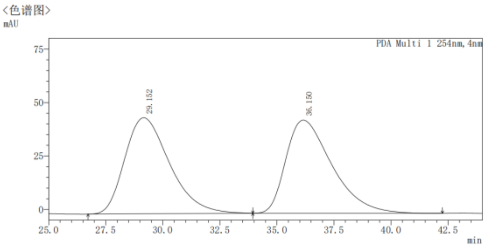

<峰表>
PDA Ch1 254nm

峰号	保留时间	面积	高度	高度%	面积%
1	29.152	6140567	44859	50.766	50.160
2	36.150	6101492	43505	49.234	49.840
总计		12242059	88364	100.000	100.000

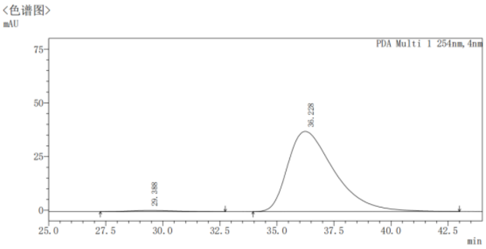

<峰表>
PDA Ch1 254nm

峰号	保留时间	面积	高度	高度%	面积%
1	29.388	68119	505	1.335	1.270
2	36.228	5297072	37366	98.665	98.730
总计		5365192	37872	100.000	100.000

7. Racemization experiments

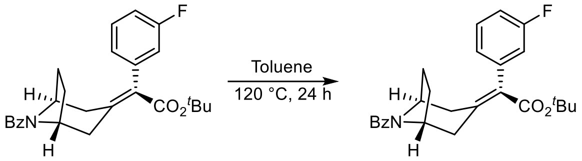

3u, 96% ee   96% ee

A solution of **3u** (10 mg, 96% ee) in toluene (1 mL) was heated at 120 °C for 24 h. Then, 0.1 mL of the solution was taken and prepared into an isopropanol solution directly. The enantiomeric excess was determined by HPLC. The results show that the chiral skeleton is very stable.

HPLC: AD-H column, hexane/^{i}PrOH = 97:3, 1.0 mL/min, t_R = 22.23 min (minor), 25.37 min (major).

Chiral HPLC spectrum of **3u**

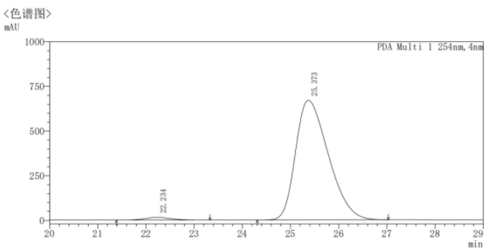

<峰表>
PDA Ch1 254nm

峰号	保留时间	面积	高度	高度%	面积%
1	22.234	572337	14959	2.186	1.775
2	25.373	31670189	669500	97.814	98.225
总计		32242526	684459	100.000	100.000

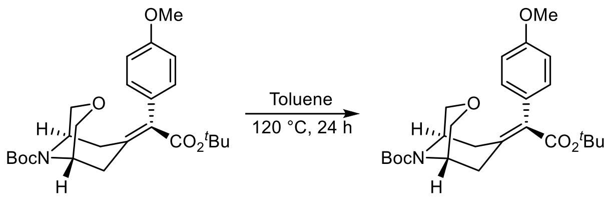

4b, 95% ee　　　　95% ee

A solution of **4b** (10 mg, 95% ee) in toluene (1 mL) was heated at 120 °C for 24 h. Then, 0.1 mL of the solution was taken and was prepared into an isopropanol solution directly. The enantiomeric excess was determined by HPLC. The results show that the chiral skeleton is very stable.

HPLC: AD-H column, hexane/^{i}PrOH = 97:3, 1.0 mL/min, t_R = 13.00 min (minor), 18.64 min (major).

Chiral HPLC spectrum of **4b**

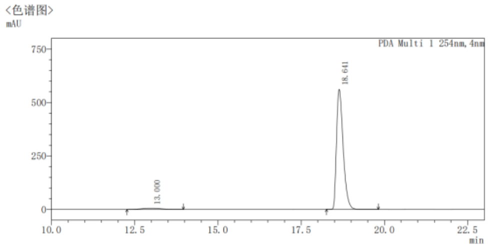

<峰表>
PDA Ch1 254nm

峰号	保留时间	面积	高度	高度%	面积%
1	13.000	194818	4649	0.822	2.442
2	18.641	7782437	560699	99.178	97.558
总计		7977255	565349	100.000	100.000

8. The X-ray diffraction analysis

Experimental Procedure Single crystals of **1b** and **11** were recrystallized by DCM/EtOAc solvent system. A suitable crystal was selected and measured on a Agilent SuperNova, Dual, Cu at zero, AtlasS2 diffractometer. The crystal was kept at 100.0(2) K during data collection.

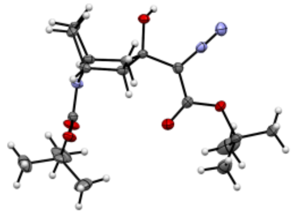

Table S2 Crystal data and structure refinement for **1b**.

CCDC	2407660
Identification code	CAJ_BOC
Empirical formula	$C_{18}H_{29}N_3O_5$
Formula weight	367.44
Temperature/K	150.00
Crystal system	triclinic
Space group	P-1
a/Å	12.2455(5)
b/Å	12.4726(5)
c/Å	16.2505(7)
α/°	82.984(2)
β/°	68.911(2)
γ/°	60.912(2)
Volume/Å^3	2019.43(15)
Z	4
ρ_{calc}g/cm^3	1.209
μ/mm^{-1}	0.728
F(000)	792.0
Crystal size/mm^3	0.15 × 0.1 × 0.08
Radiation	CuKα (λ = 1.54178)
2Θ range for data collection/°	8.128 to 136.372
Index ranges	$-14 \leq h \leq 14$, $-14 \leq k \leq 15$, $-19 \leq l \leq 19$
Reflections collected	57351
Independent reflections	7364 [R_{int} = 0.0837, R_{sigma} = 0.0471]
Data/restraints/parameters	7364/6/483
Goodness-of-fit on F^2	1.111
Final R indexes [I>=2σ (I)]	R_1 = 0.0548, wR_2 = 0.1419

Final R indexes [all data]	R_1 = 0.0696, wR_2 = 0.1484
Largest diff. peak/hole / e Å^{-3}	0.49/-0.38

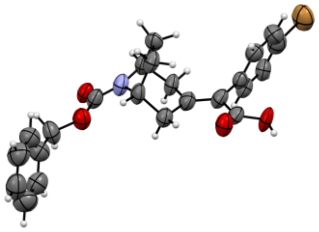

Table S3 Crystal data and structure refinement for **11**.

CCDC	2378186
Identification code	CJY
Empirical formula	$C_{23}H_{22}BrNO_4$
Formula weight	456.32
Temperature/K	200.00
Crystal system	monoclinic
Space group	$P2_1$
a/Å	6.3008(4)
b/Å	8.5061(5)
c/Å	19.2716(12)
α/°	90
β/°	96.221(3)
γ/°	90
Volume/Å^3	1026.78(11)
Z	2
ρ_{calc}g/cm^3	1.476
μ/mm^{-1}	2.975
F(000)	468.0
Crystal size/mm^3	0.12 × 0.1 × 0.08
Radiation	CuKα (λ = 1.54178)
2Θ range for data collection/°	4.612 to 135.98
Index ranges	$-7 \le h \le 7, 0 \le k \le 10, 0 \le l \le 23$
Reflections collected	1995
Independent reflections	1995 [R_{int} = 0.0703, R_{sigma} = 0.1016]
Data/restraints/parameters	1995/19/263
Goodness-of-fit on F^2	0.849
Final R indexes [I>=2σ (I)]	R_1 = 0.0800, wR_2 = 0.1894
Final R indexes [all data]	R_1 = 0.1128, wR_2 = 0.2066
Largest diff. peak/hole / e Å^{-3}	1.07/-0.58
Flack parameter	0.11(6)

9. References

[1] Bollinger, S. et al. Novel pyridylmethylamines as highly selective 5-HT1A superagonists. *J. Med. Chem.* **53**, 7167-7179 (2010).

[2] Li, W.-Y. et al. External photocatalyst-free visible light-promoted 1,3-addition of perfluoroalkyl iodides to vinyldiazoacetates. *CCS Chem.* **4**, 638-649 (2022).

[3] Qiu, S.-Q. et al. Asymmetric construction of an aryl-akene axis by Palladium-catalyzed Suzuki-Miyaura coupling reaction. *Angew. Chem. Int. Ed.* **61**, e202211211 (2022).

[4] Wang, Y.-S. et al. Expedient synthesis of axially and centrally chiral diaryl ethers via Cobalt-catalyzed photoreductive desymmetrization. *ACS Catal.* **14**, 4638-4647 (2024).

10. The NMR spectra

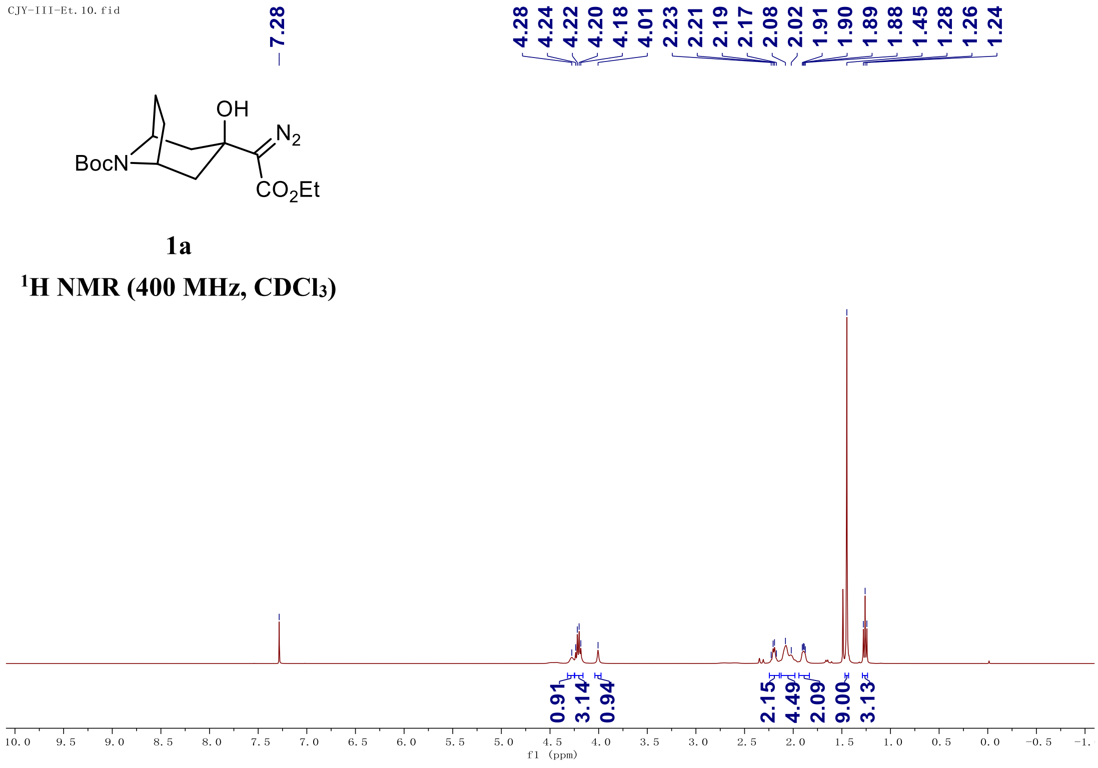

Figure S1 ^{1}H NMR (400 MHz, $CDCl_3$) spectrum for **1a**

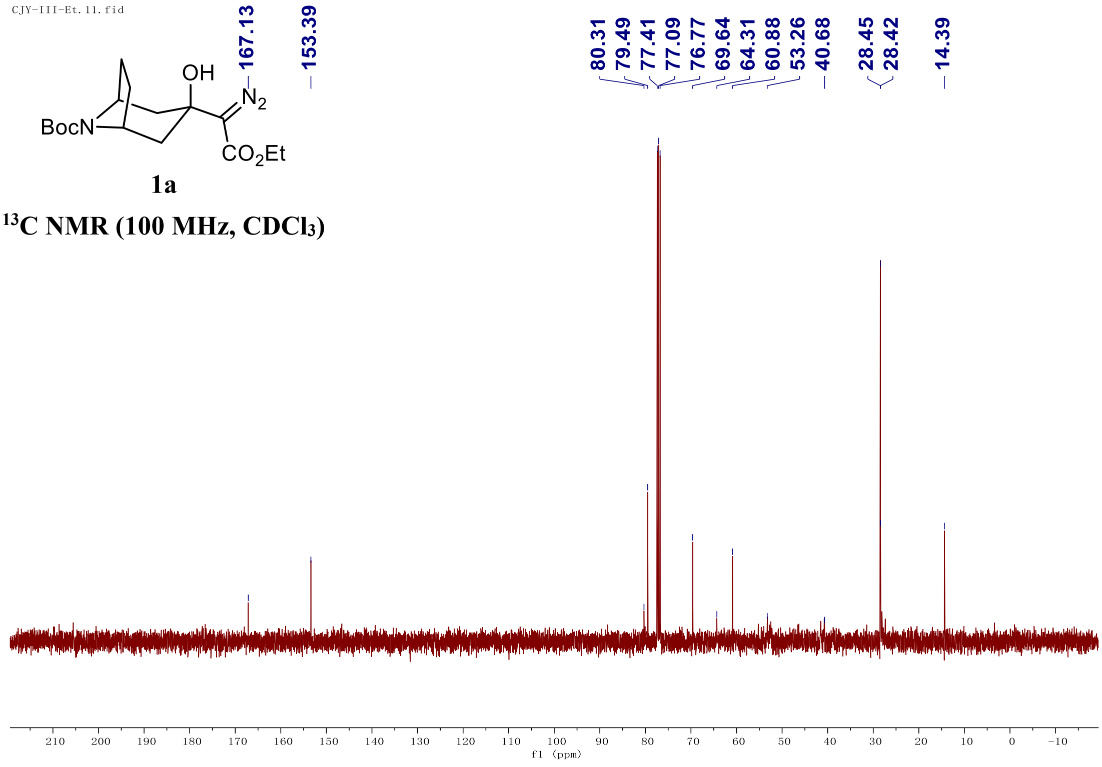

Figure S2 ^{13}C NMR (100 MHz, $CDCl_3$) spectrum for **1a**

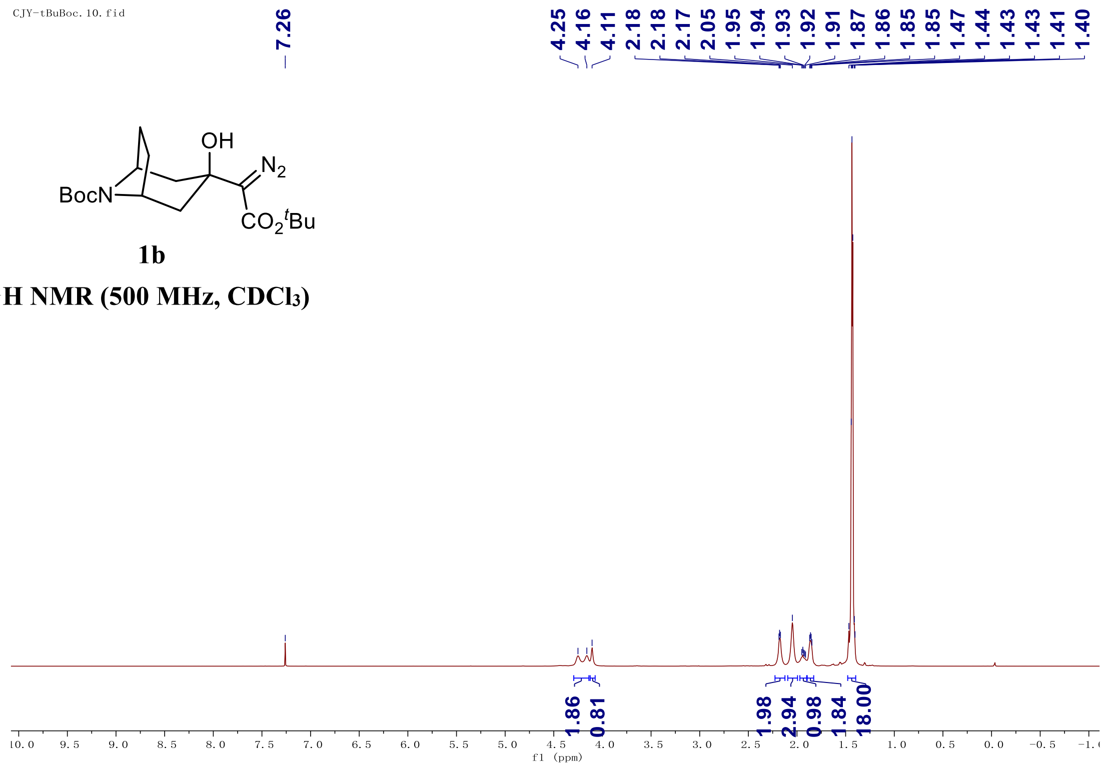

Figure S3 ^{1}H NMR (500 MHz, $CDCl_3$) spectrum for **1b**

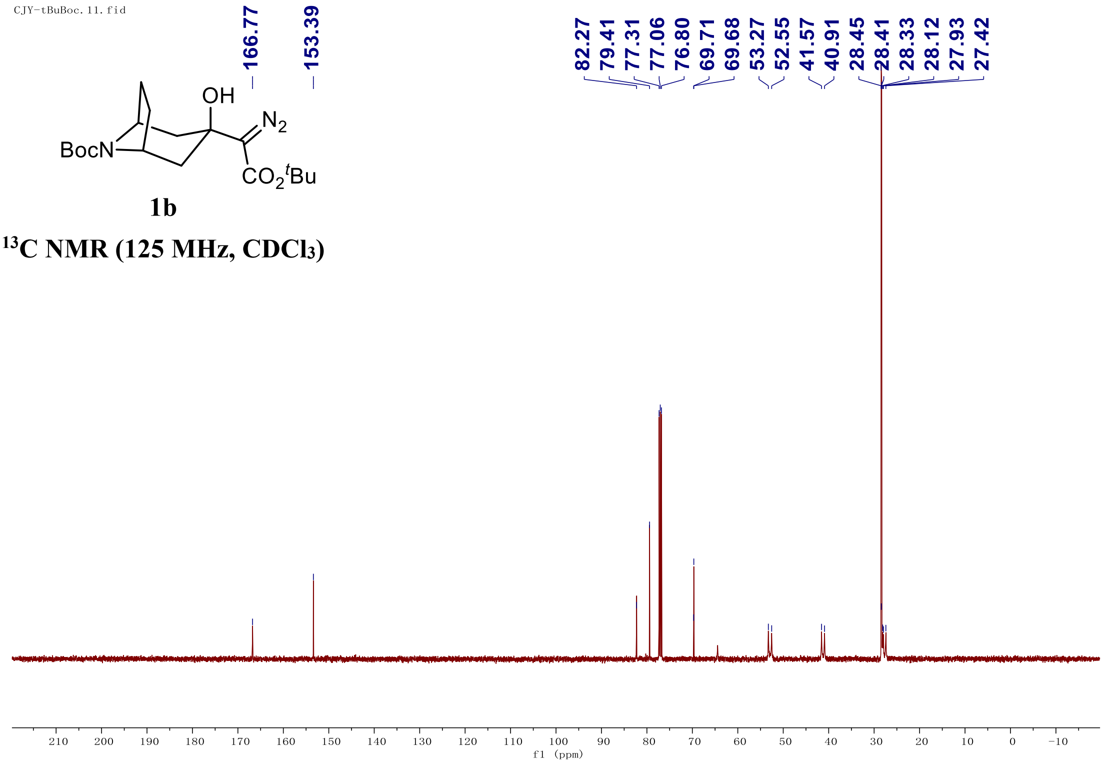

Figure S4 ^{13}C NMR (125 MHz, $CDCl_3$) spectrum for **1b**

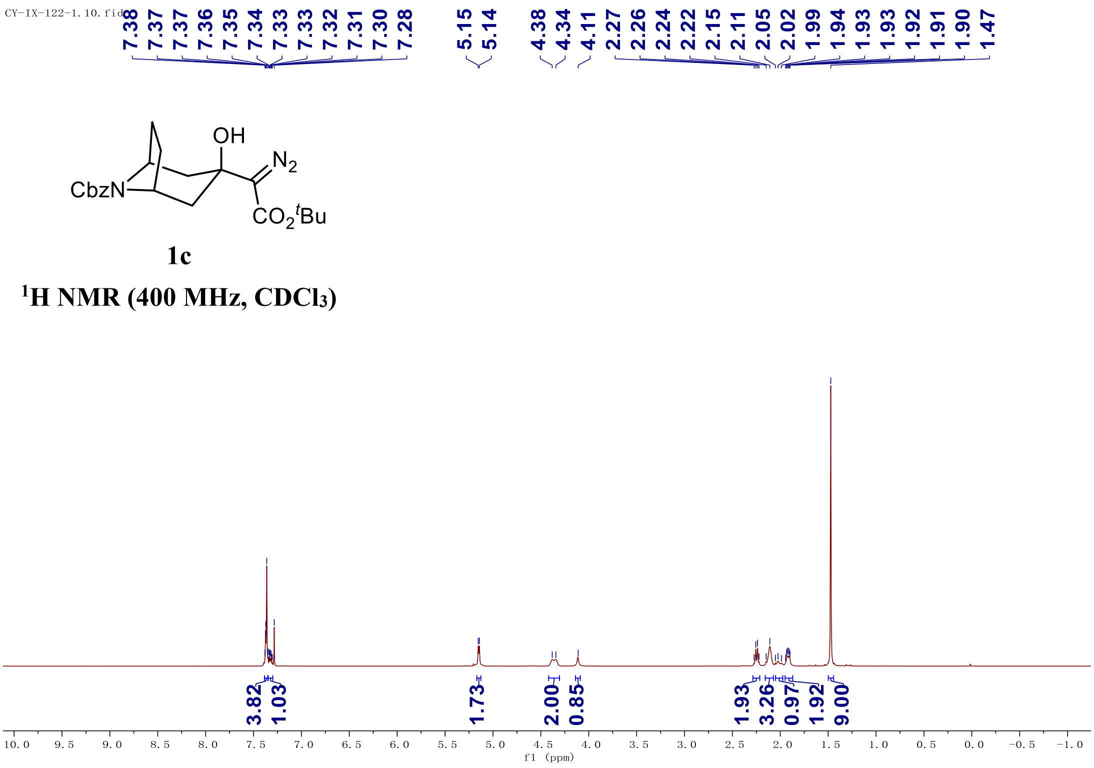

Figure S5 ^{1}H NMR (400 MHz, CDCl$_3$) spectrum for **1c**

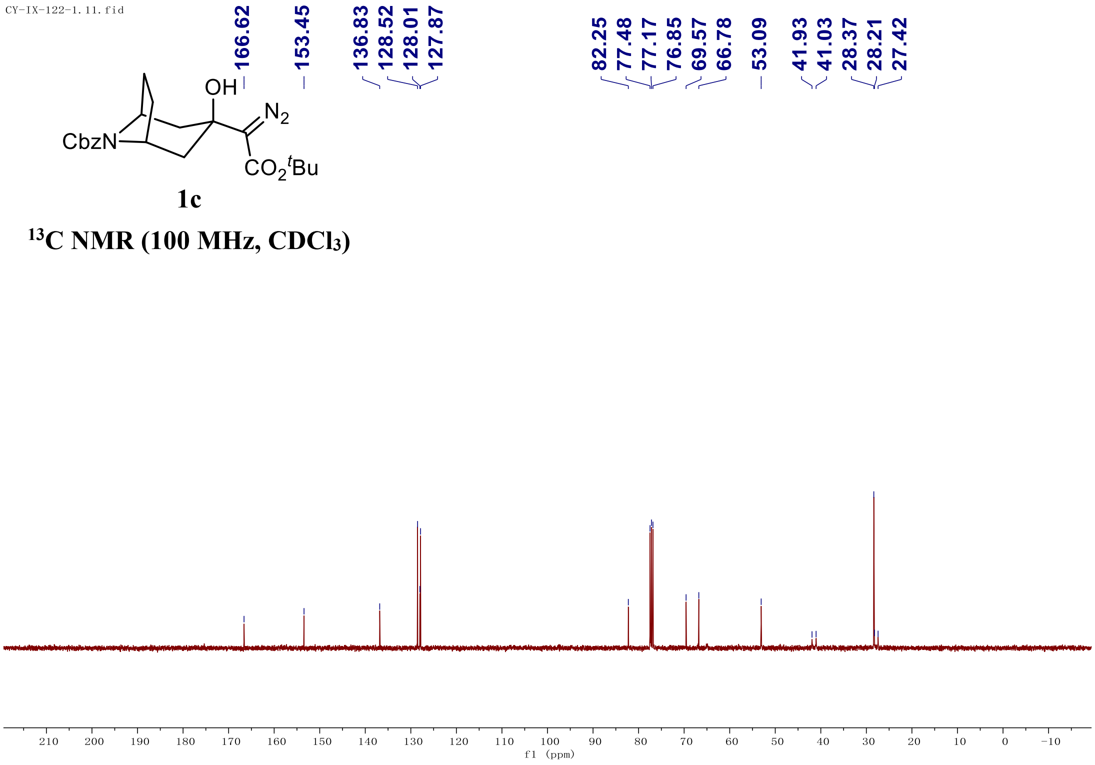

Figure S6 ^{13}C NMR (100 MHz, CDCl$_3$) spectrum for **1c**

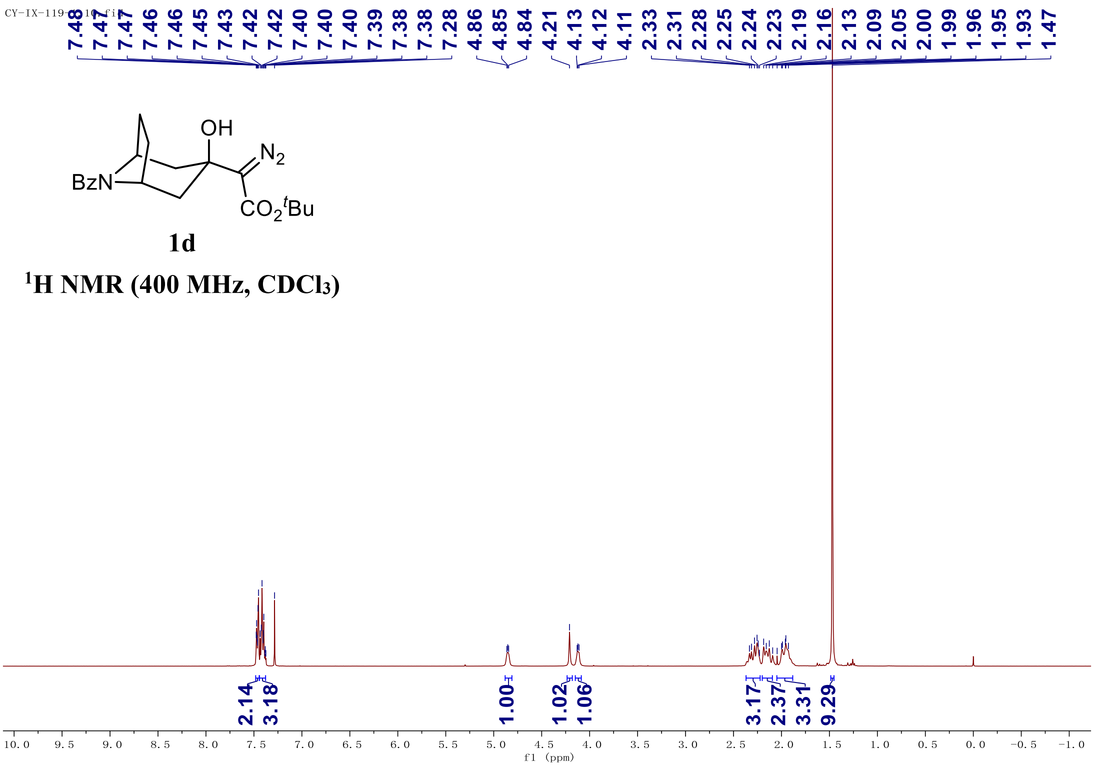

Figure S7 ^1H NMR (400 MHz, $CDCl_3$) spectrum for **1d**

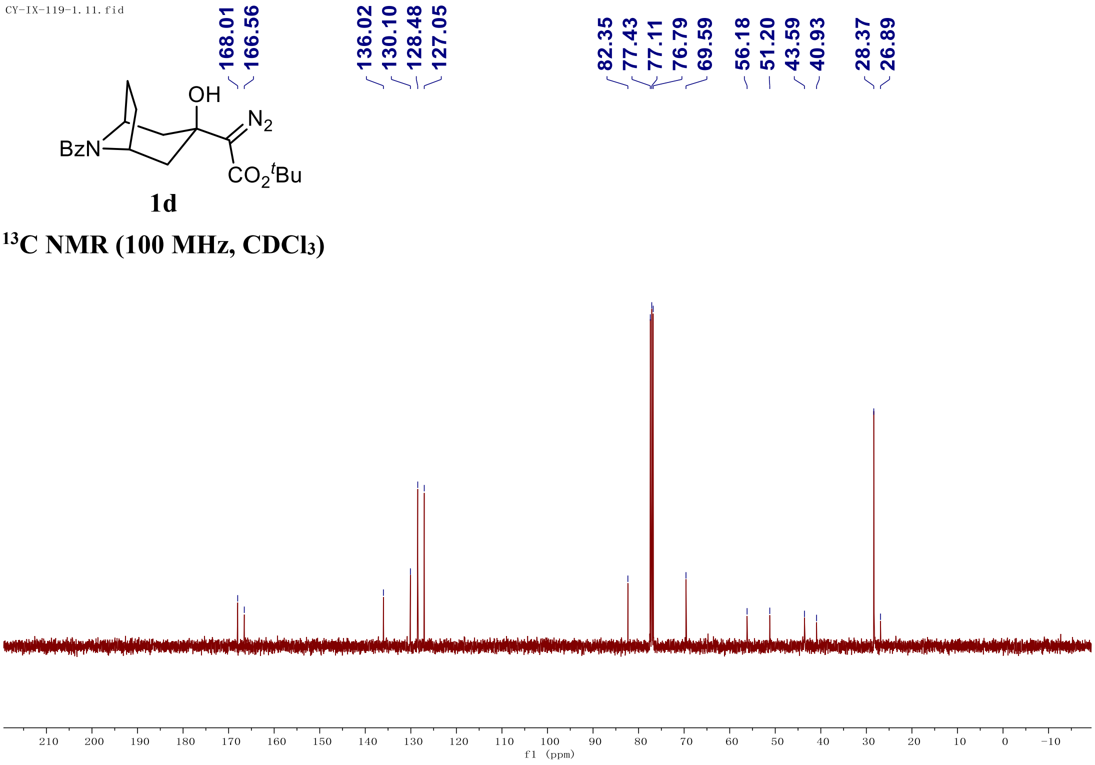

Figure S8 ^{13}C NMR (100 MHz, $CDCl_3$) spectrum for **1d**

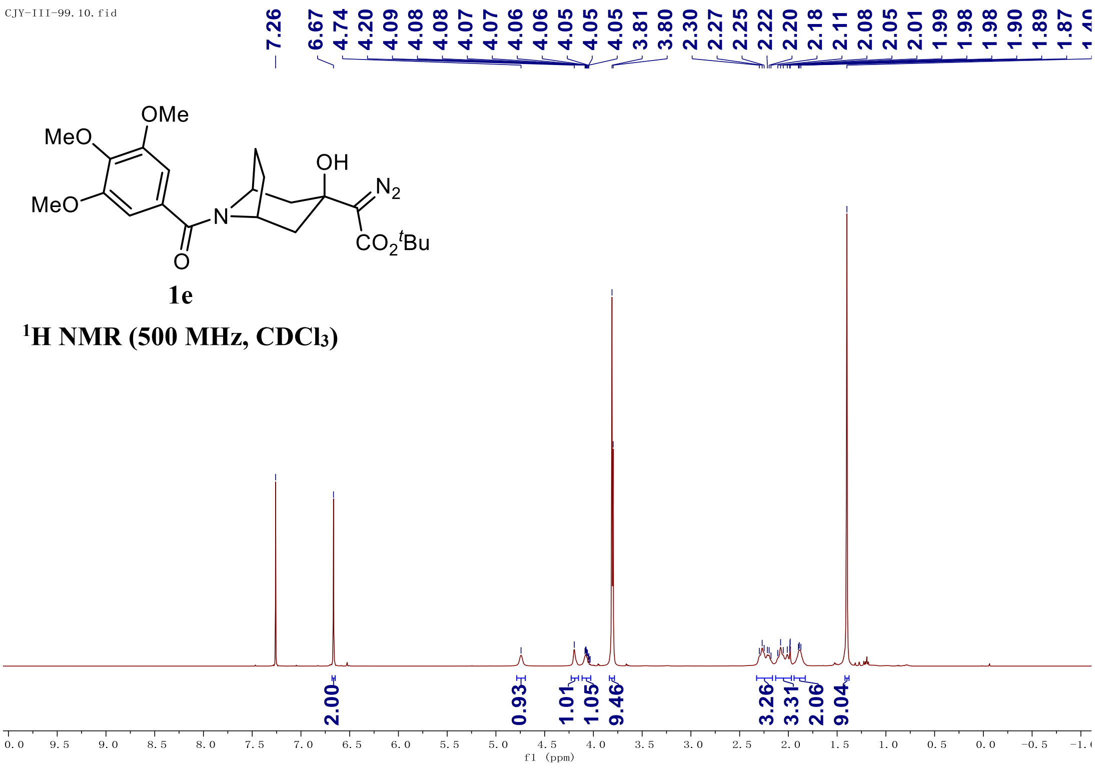

Figure S9 ^{1}H NMR (500 MHz, $CDCl_3$) spectrum for **1e**

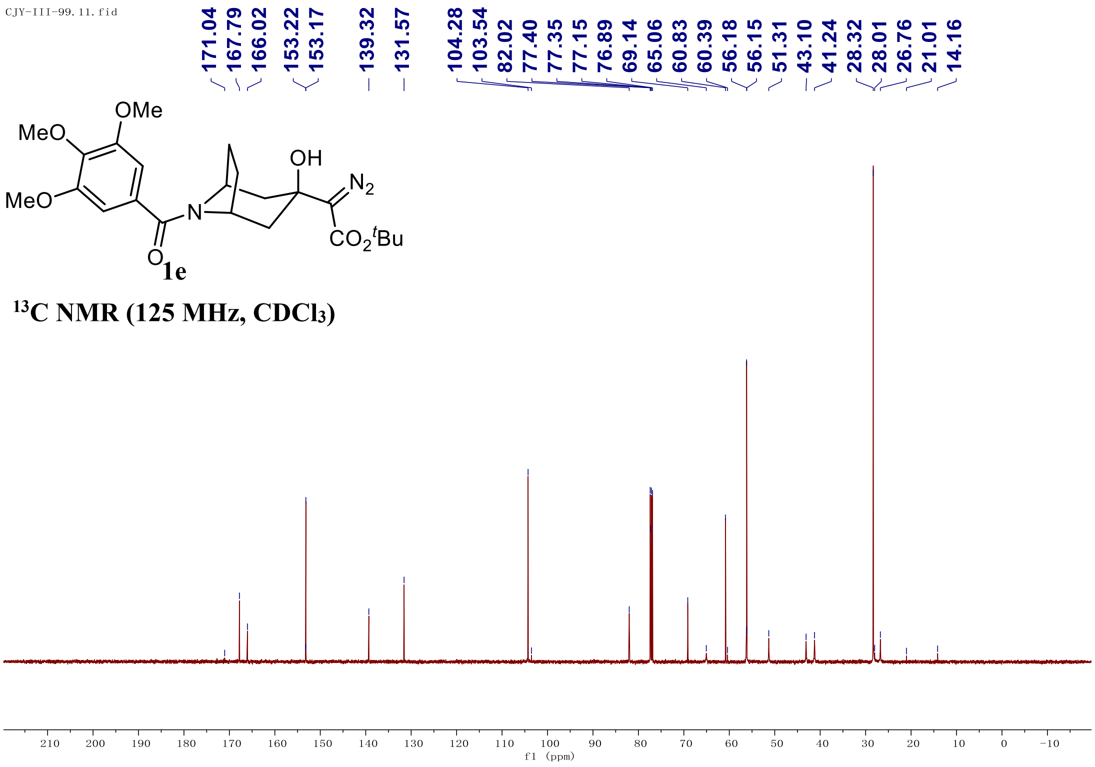

Figure S10 ^{13}C NMR (125 MHz, $CDCl_3$) spectrum for **1e**

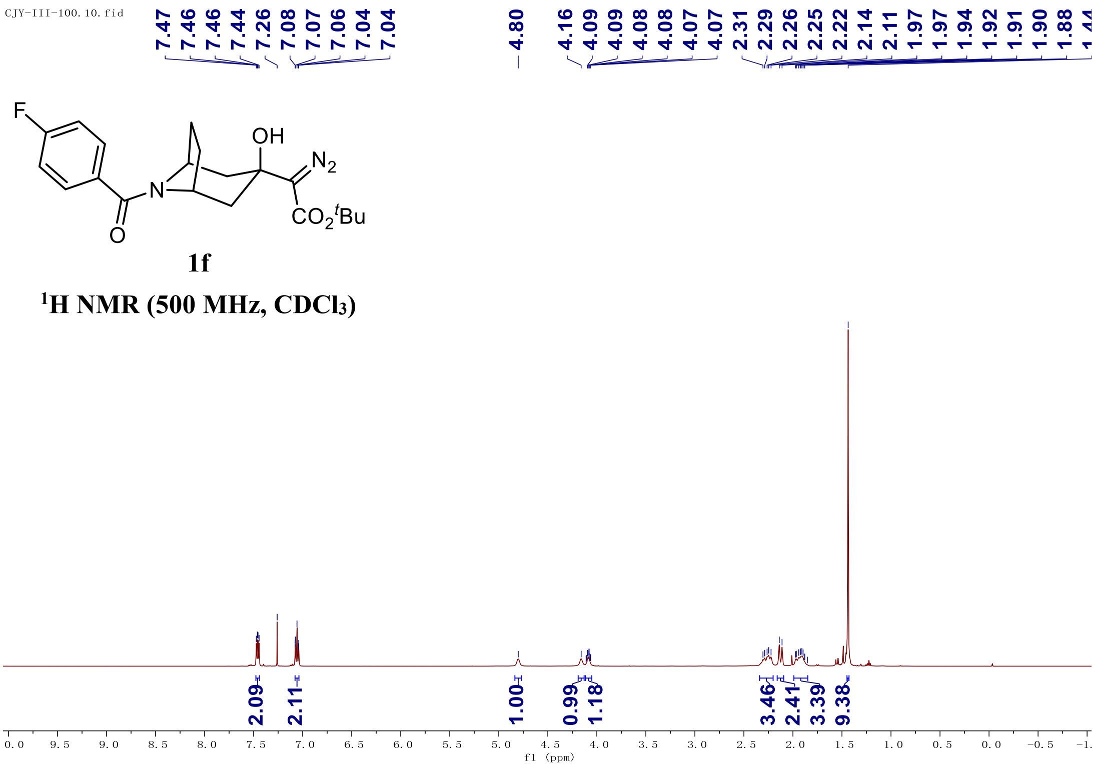

Figure S11 ^{1}H NMR (500 MHz, $CDCl_3$) spectrum for **1f**

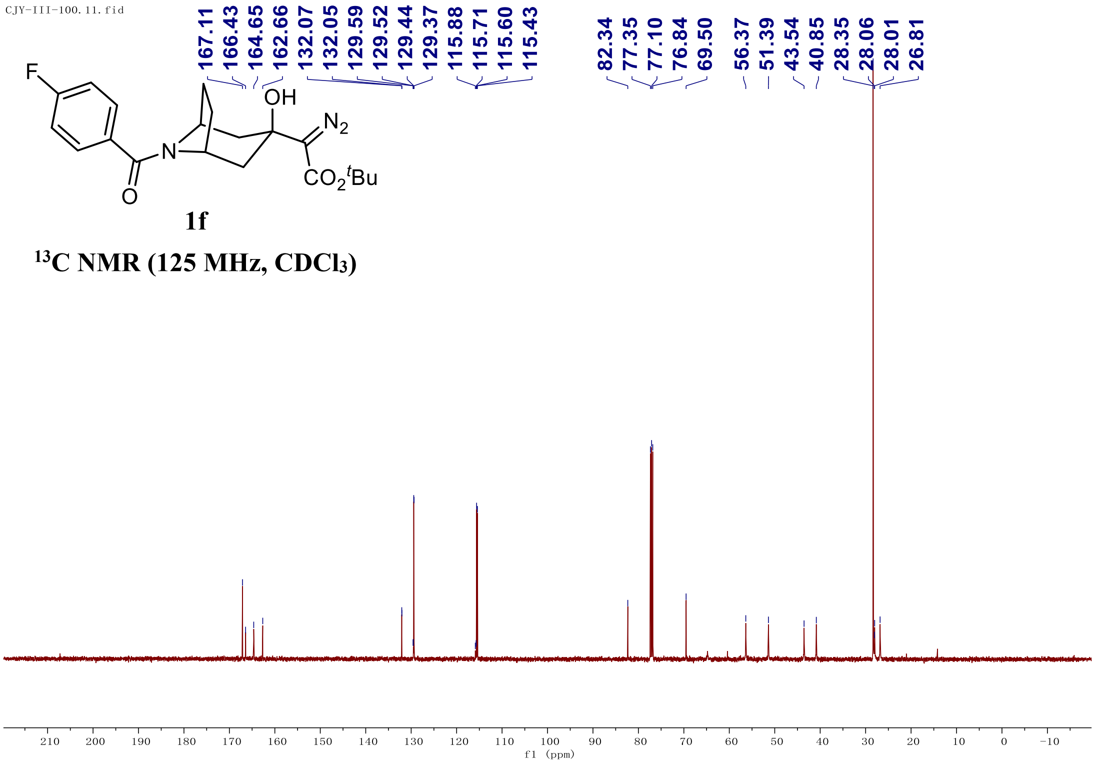

Figure S12 ^{13}C NMR (125 MHz, $CDCl_3$) spectrum for **1f**

mlh-III-100.10.fid

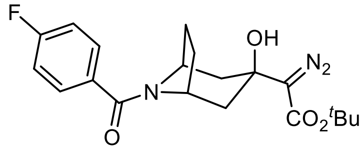

1f

^{19}F NMR (376 MHz, $CDCl_3$)

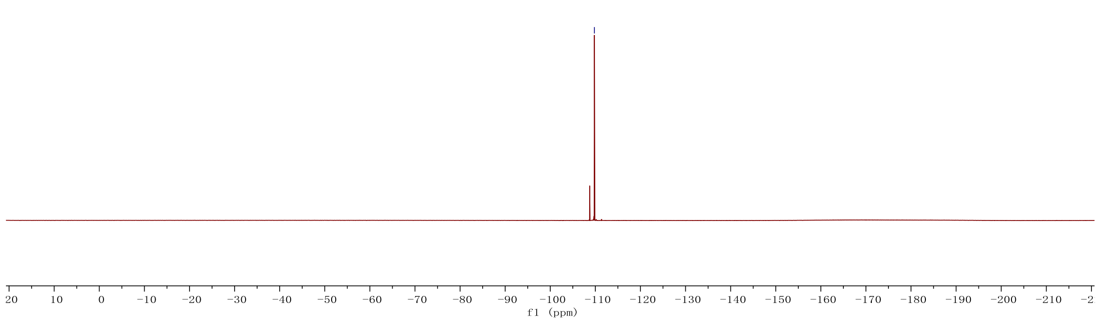

Figure S13 ^{19}F NMR (376 MHz, $CDCl_3$) spectrum for **1f**

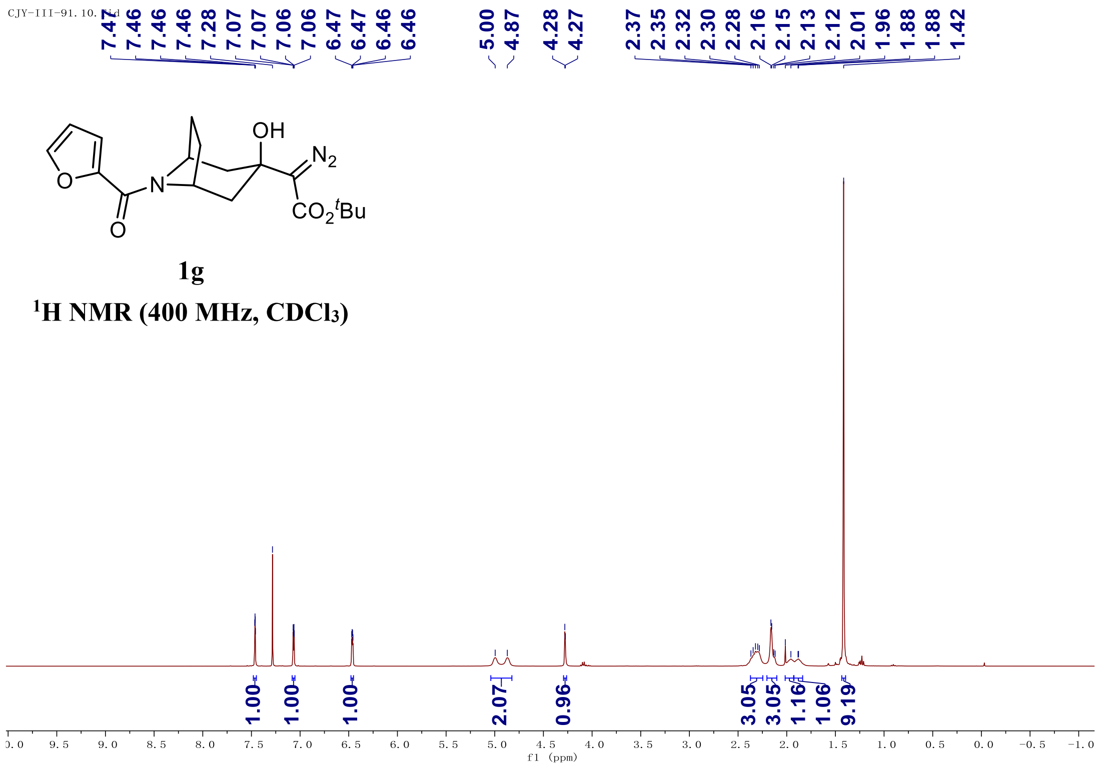

Figure S14 ^{1}H NMR (400 MHz, CDCl$_3$) spectrum for **1g**

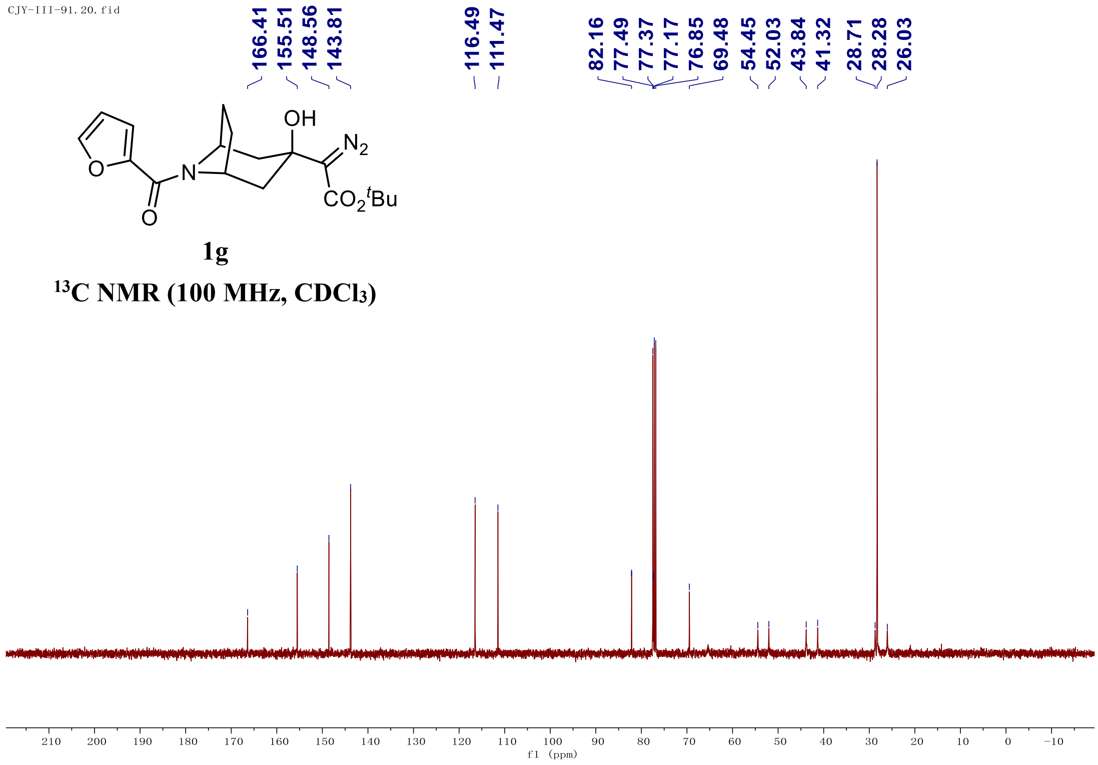

Figure S15 ^{13}C NMR (100 MHz, CDCl$_3$) spectrum for **1g**

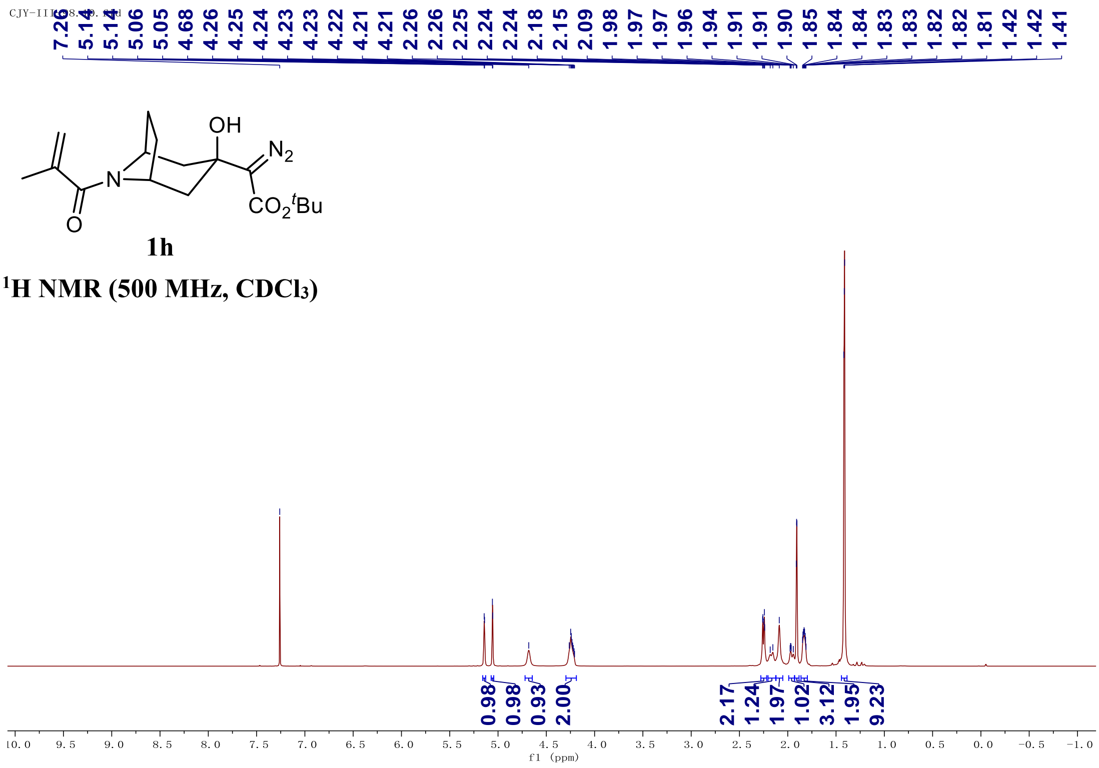

Figure S16 ^{1}H NMR (500 MHz, $CDCl_3$) spectrum for **1h**

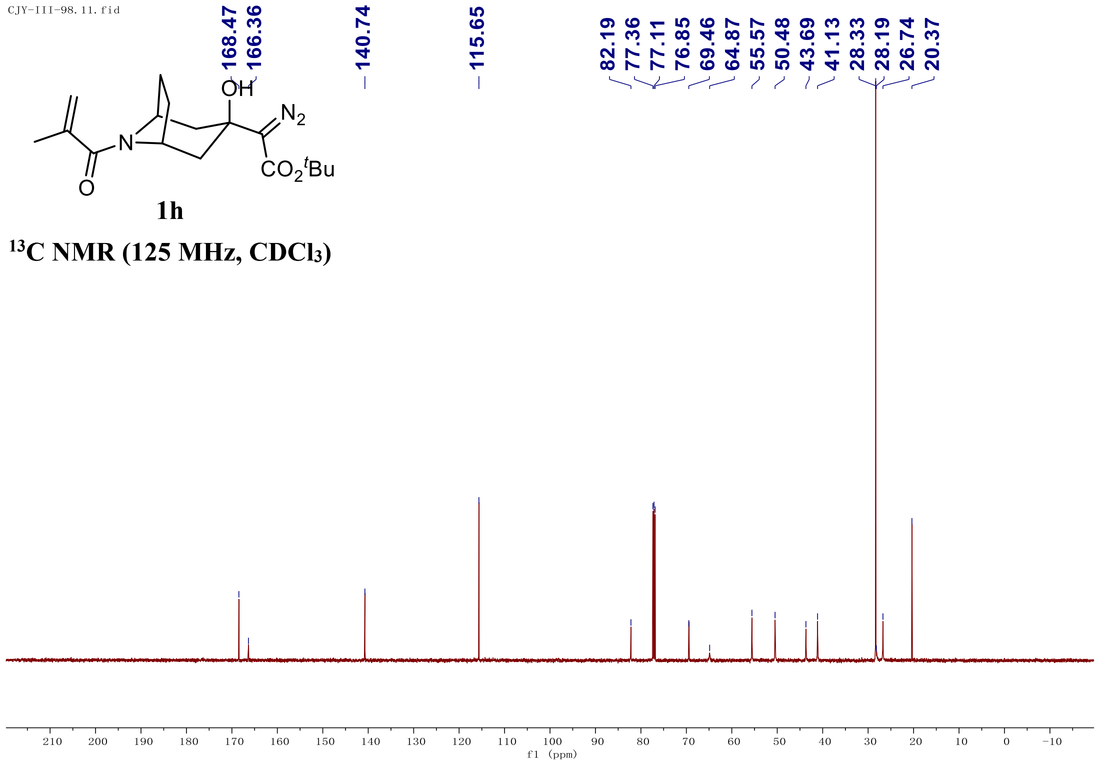

Figure S17 ^{13}C NMR (125 MHz, $CDCl_3$) spectrum for **1h**

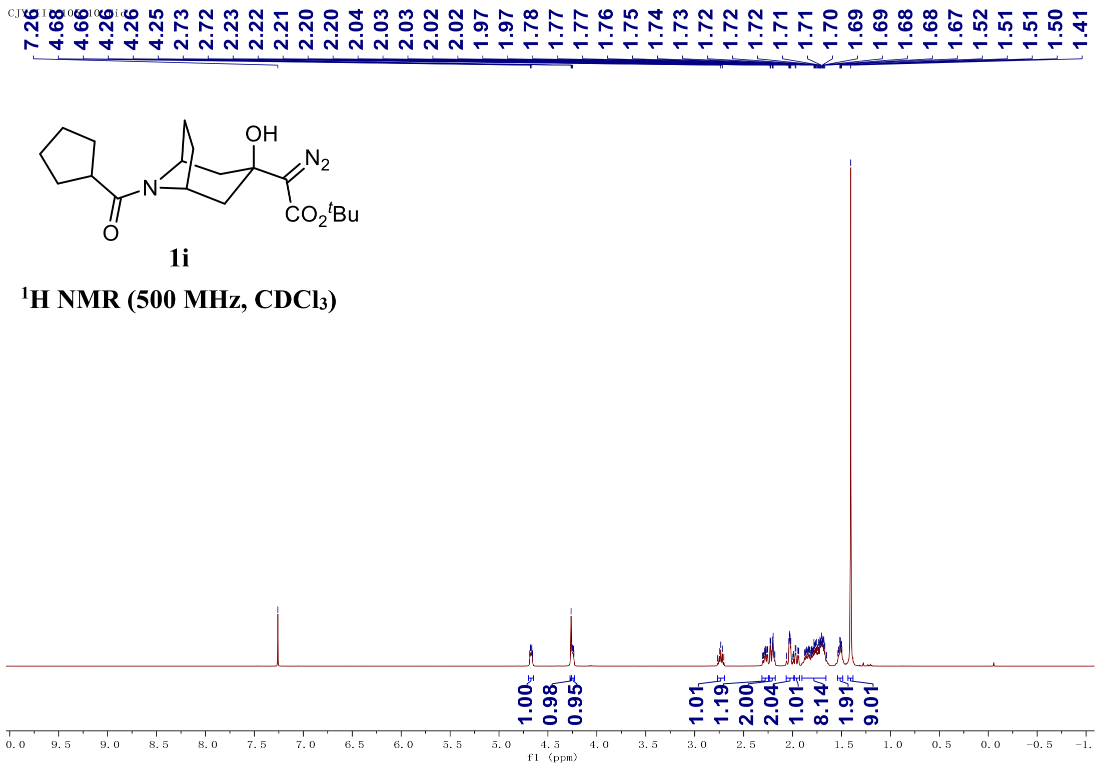

Figure S18 ^1H NMR (500 MHz, $CDCl_3$) spectrum for **1i**

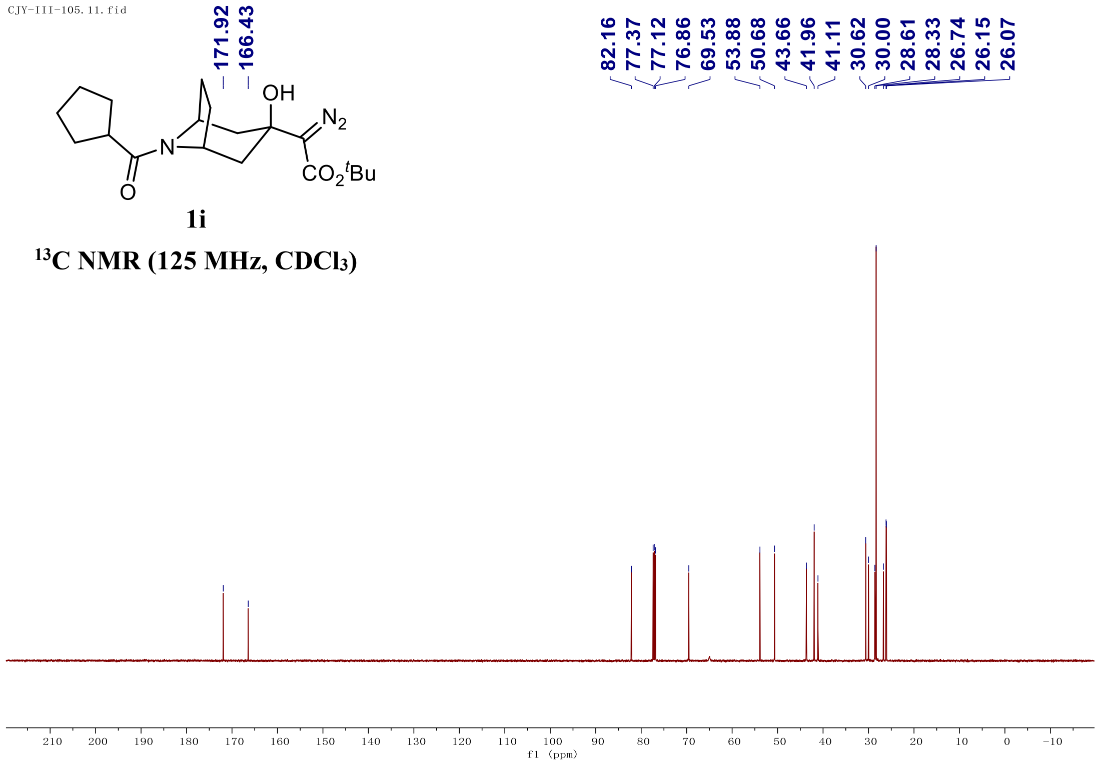

Figure S19 ^{13}C NMR (125 MHz, $CDCl_3$) spectrum for **1i**

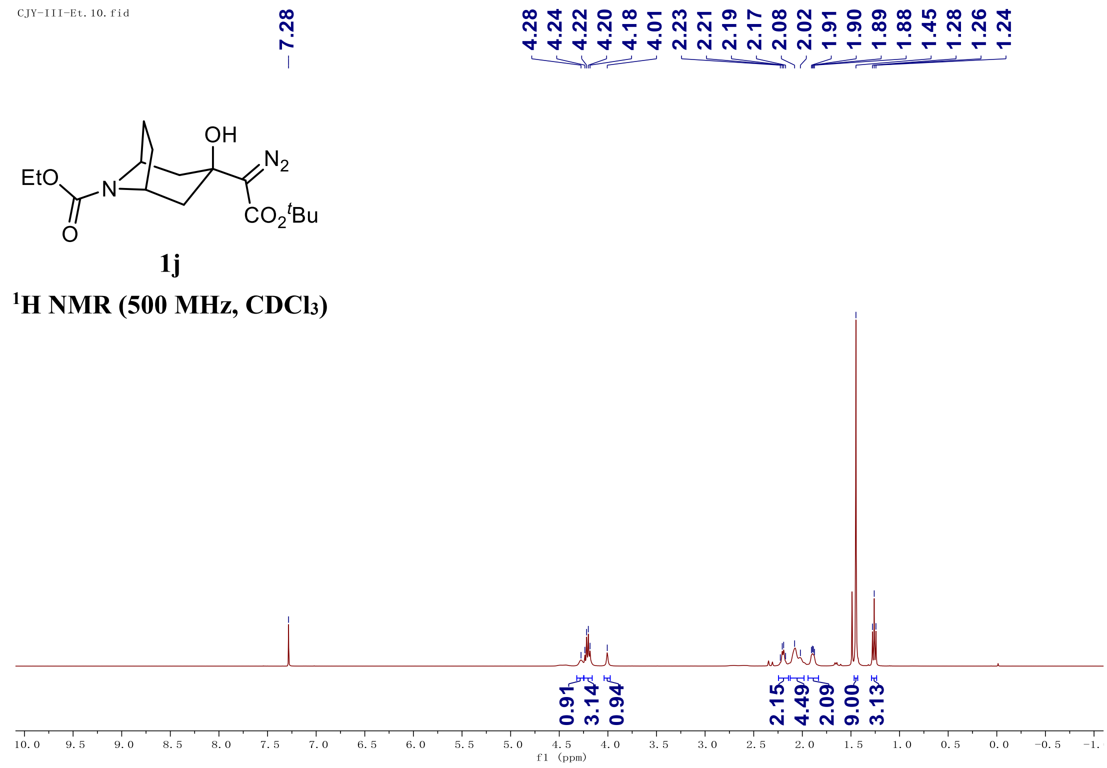

Figure S20 ^1H NMR (500 MHz, $CDCl_3$) spectrum for **1j**

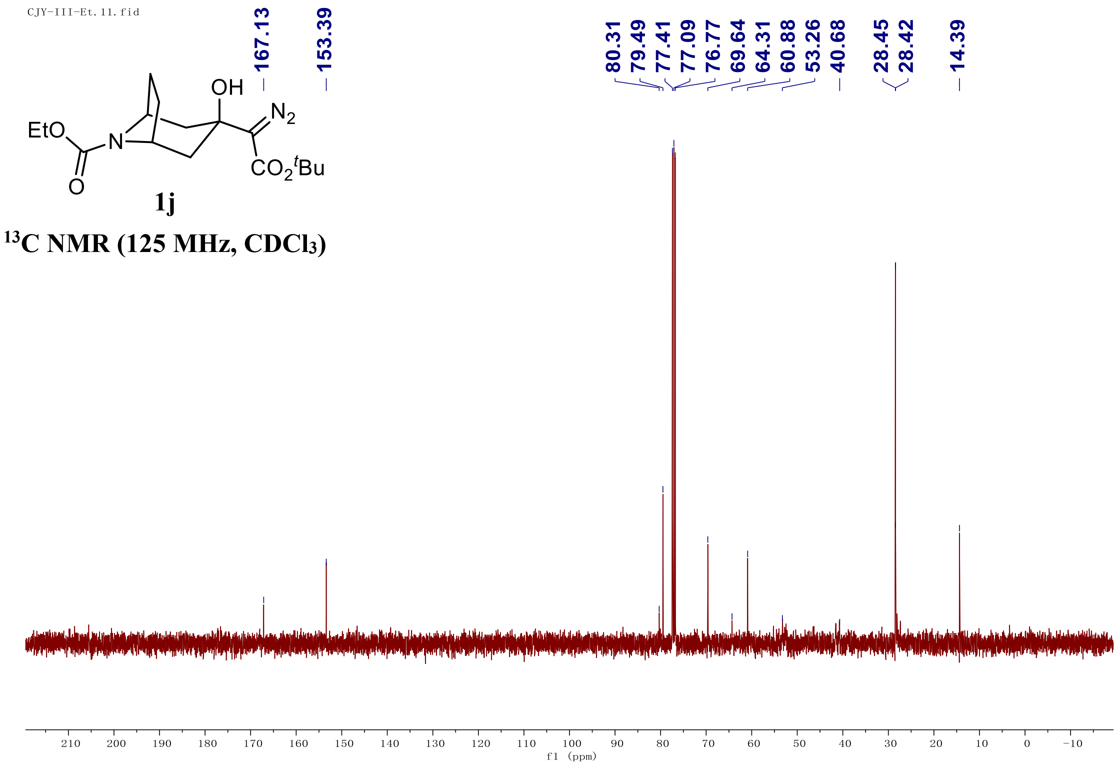

Figure S21 ^{13}C NMR (125 MHz, $CDCl_3$) spectrum for **1j**

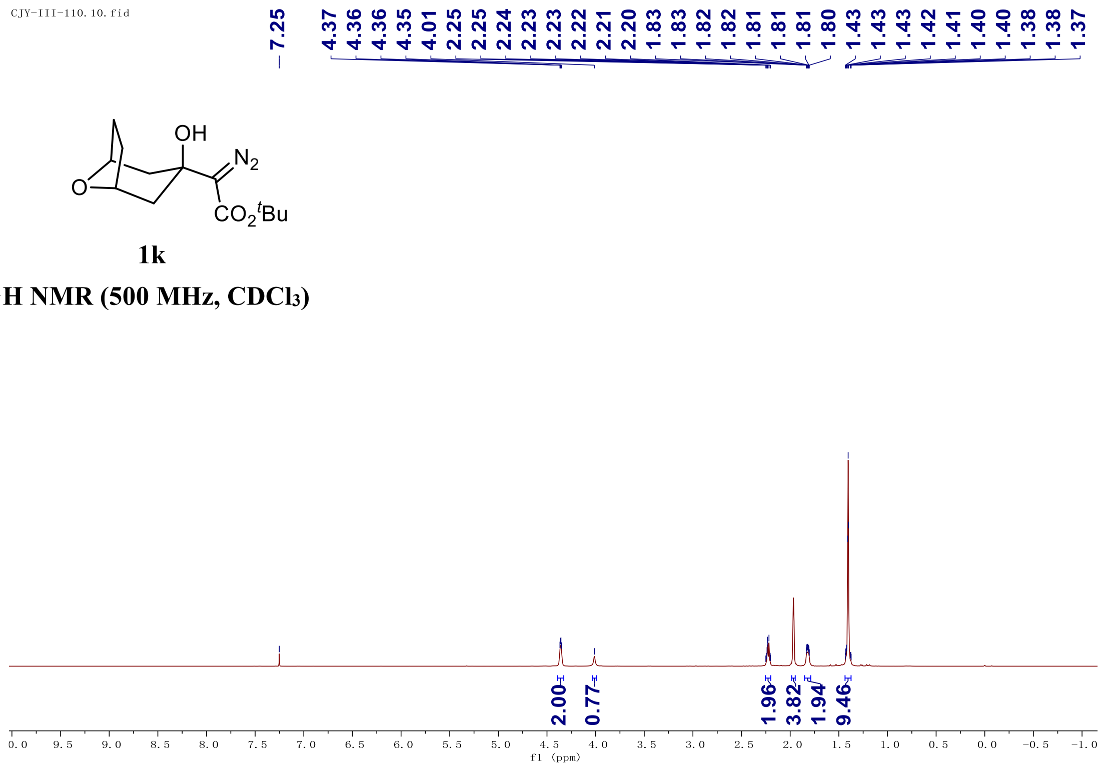

Figure S22 ^{1}H NMR (500 MHz, $CDCl_3$) spectrum for **1k**

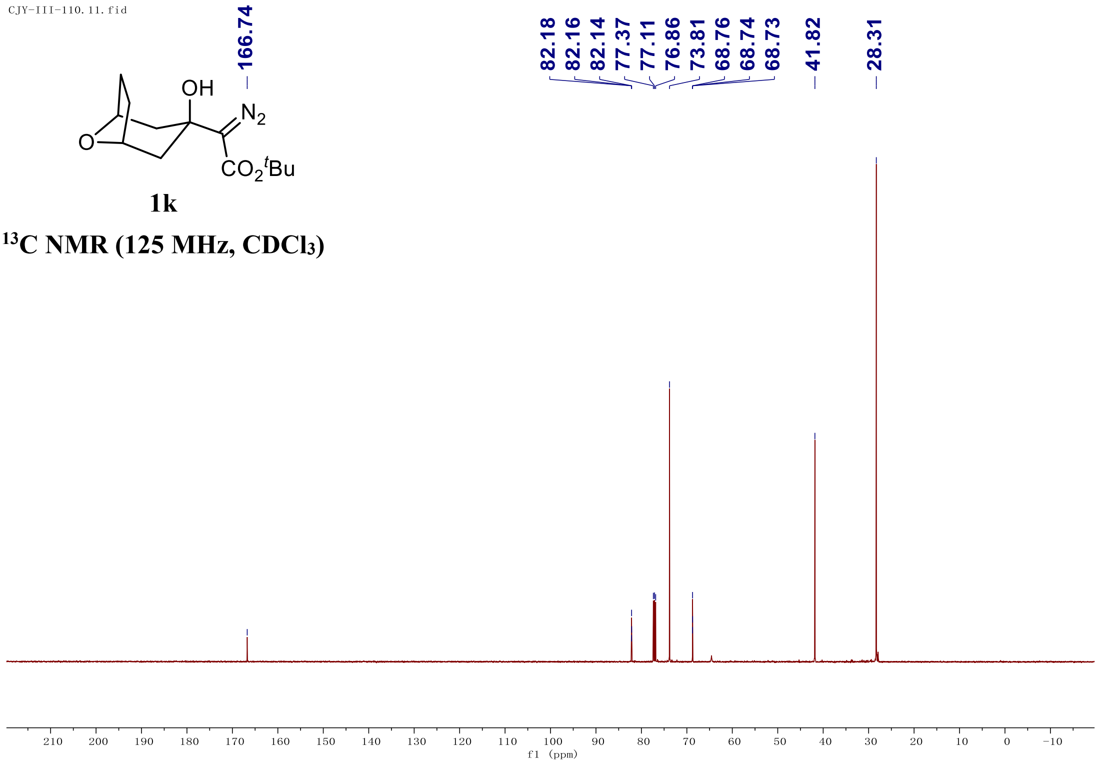

Figure S23 ^{13}C NMR (125 MHz, $CDCl_3$) spectrum for **1k**

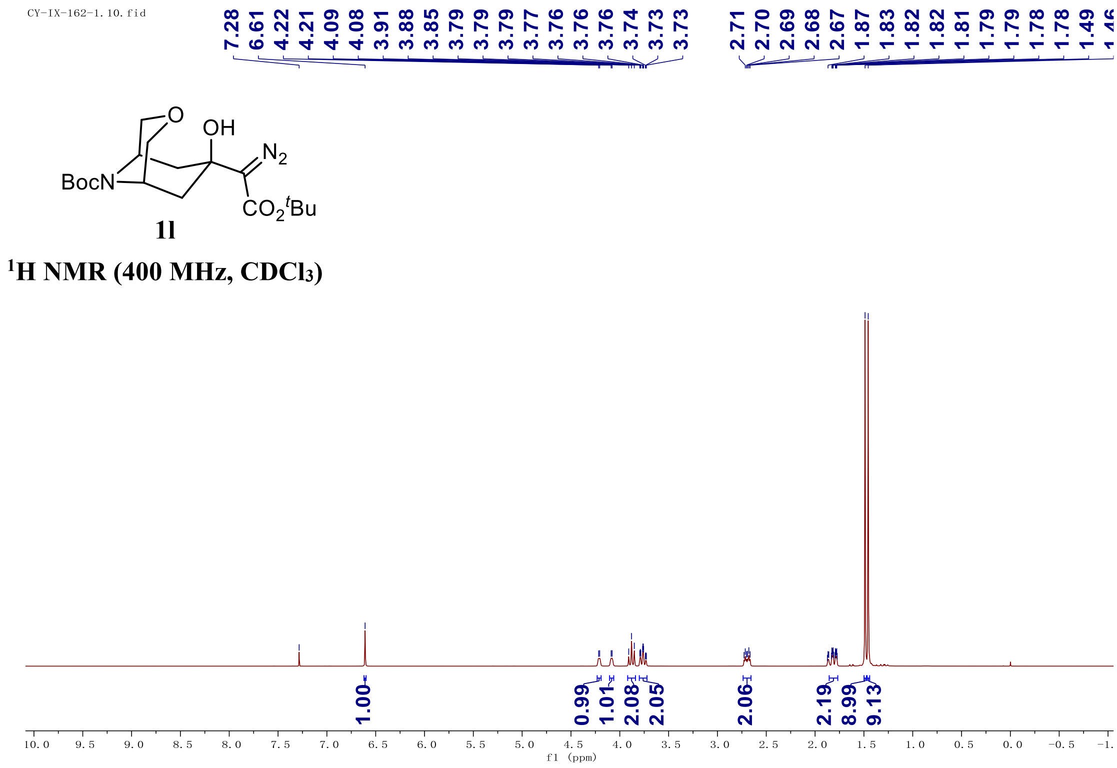

Figure S24 ^{1}H NMR (400 MHz, $CDCl_3$) spectrum for **1l**

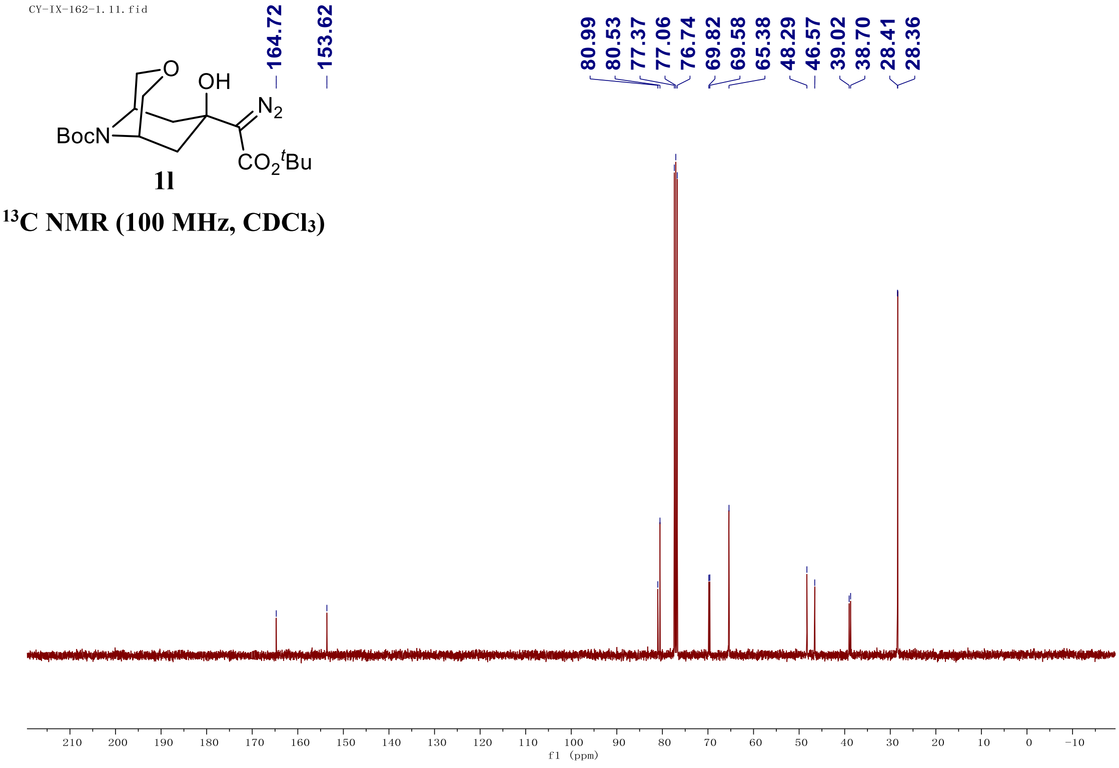

Figure S25 ^{13}C NMR (100 MHz, $CDCl_3$) spectrum for **1l**

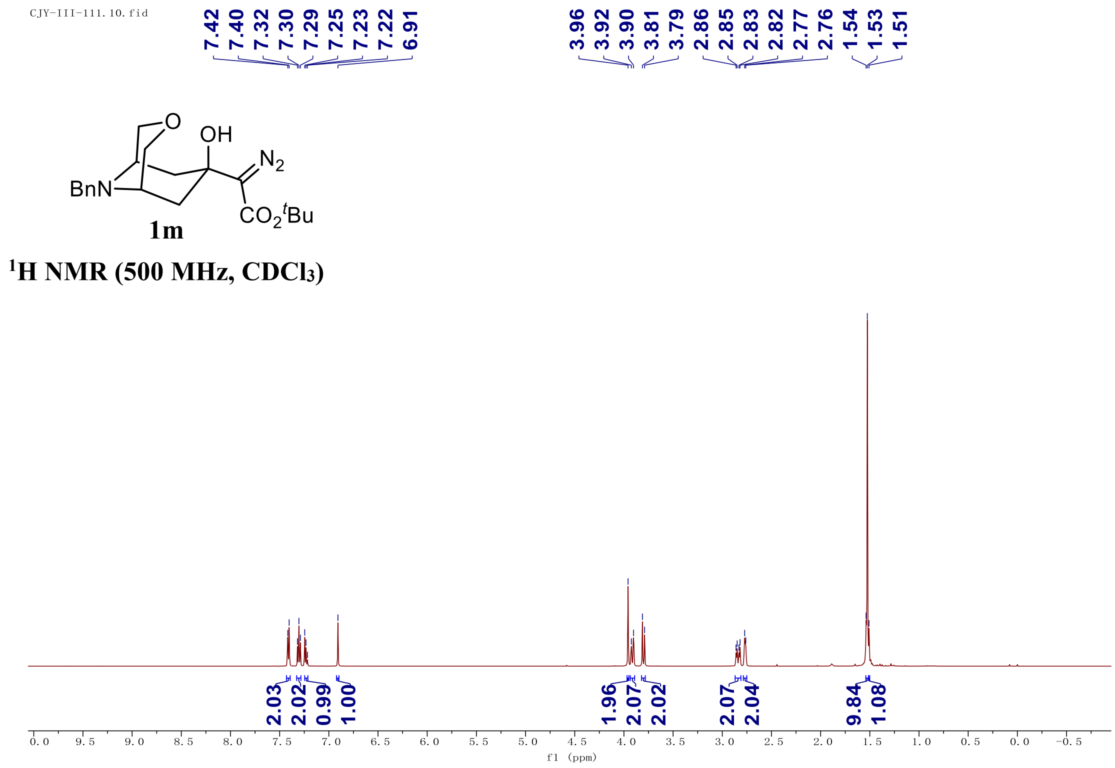

Figure S26 ^{1}H NMR (500 MHz, $CDCl_3$) spectrum for **1m**

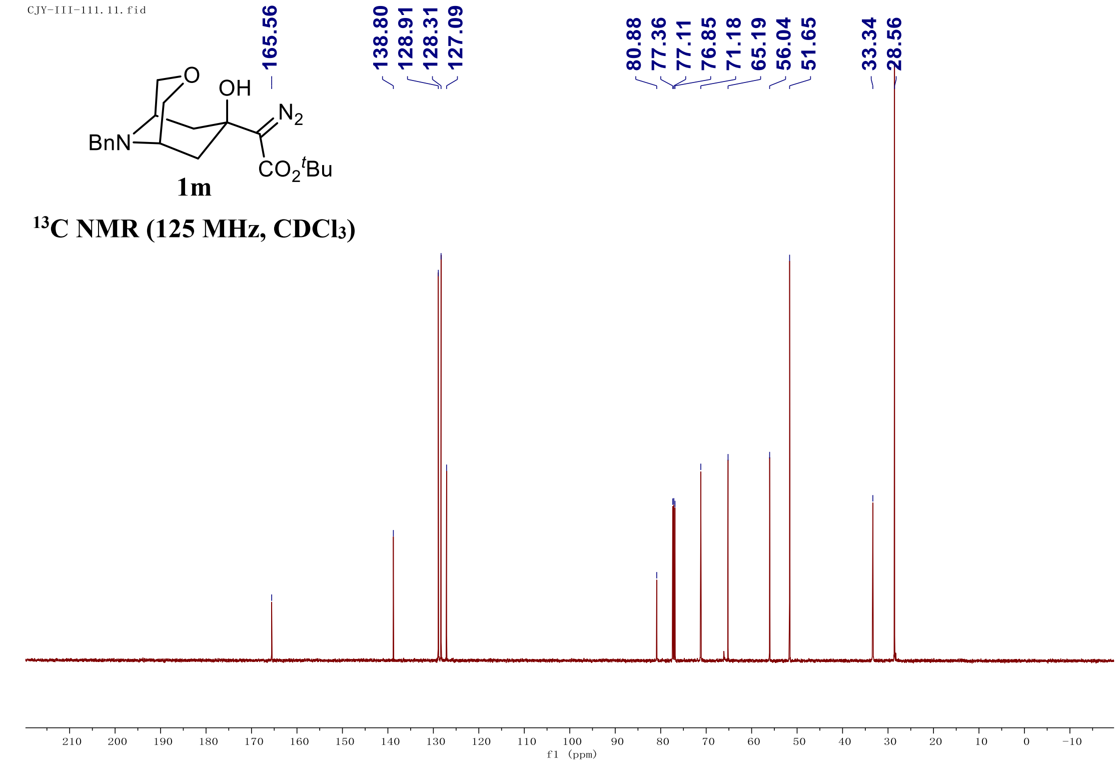

Figure S27 ^{13}C NMR (125 MHz, $CDCl_3$) spectrum for **1m**

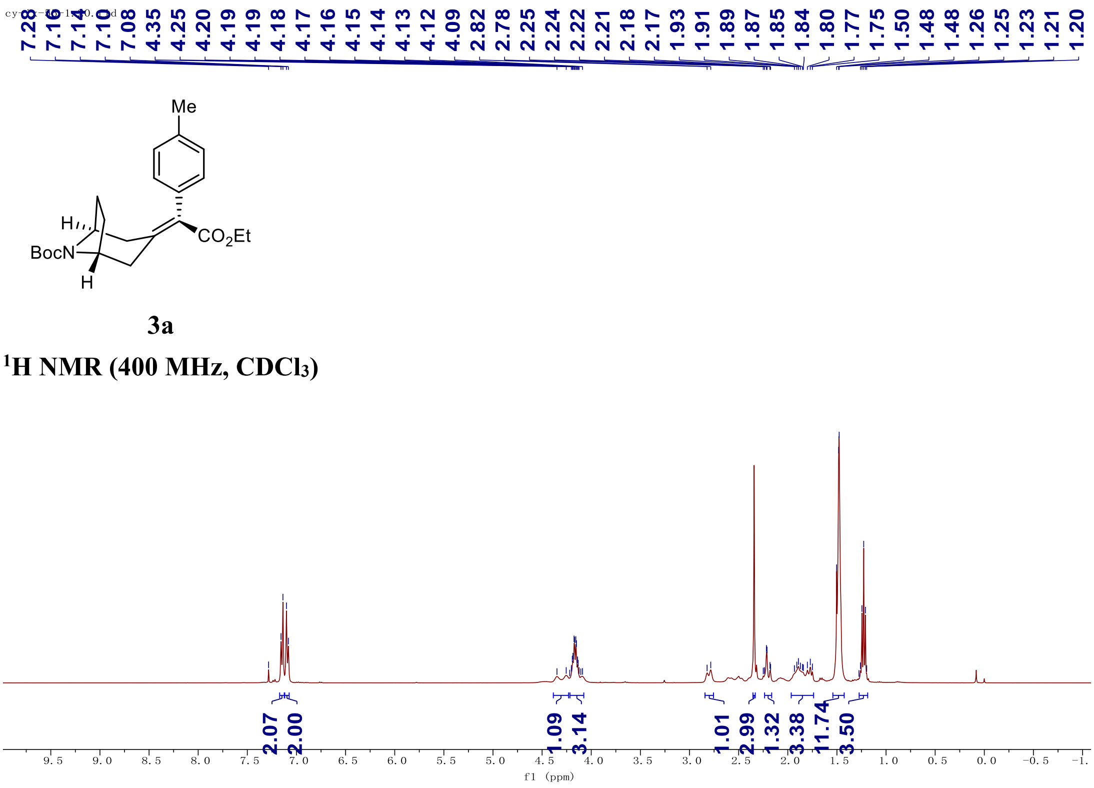

Figure S28 ^{1}H NMR (400 MHz, $CDCl_3$) spectrum for **3a**

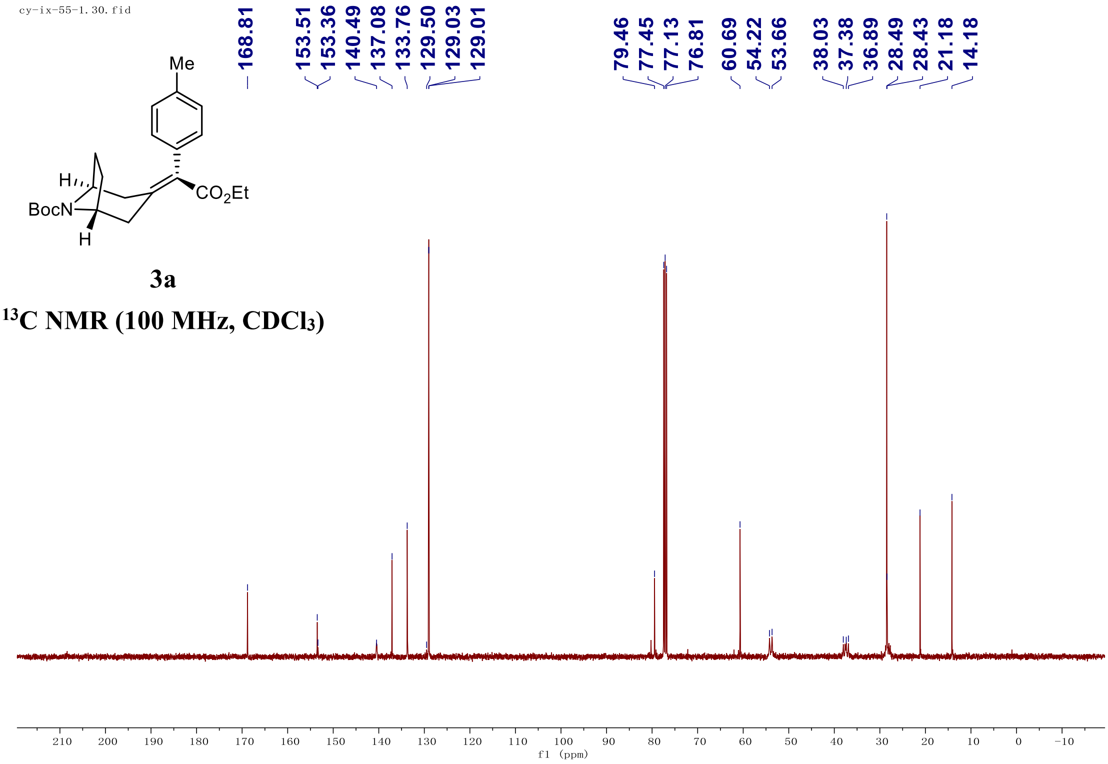

Figure S29 ^{13}C NMR (100 MHz, $CDCl_3$) spectrum for **3a**

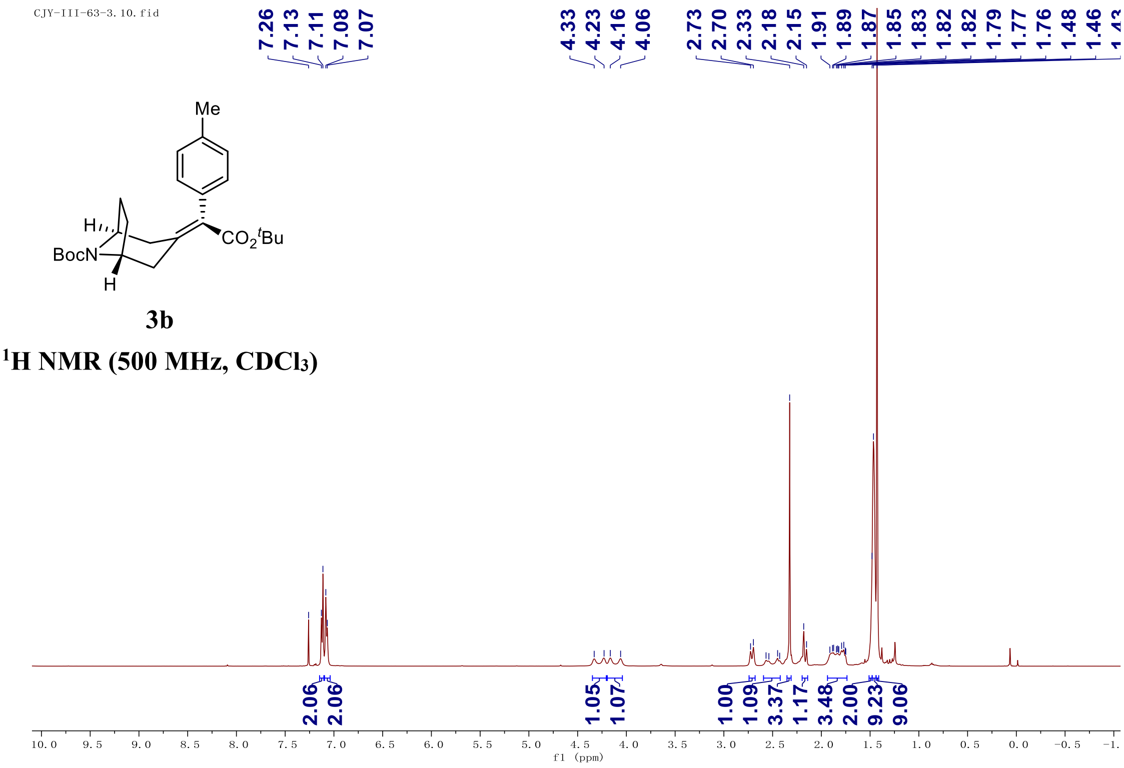

Figure S30 ^{1}H NMR (500 MHz, $CDCl_3$) spectrum for **3b**

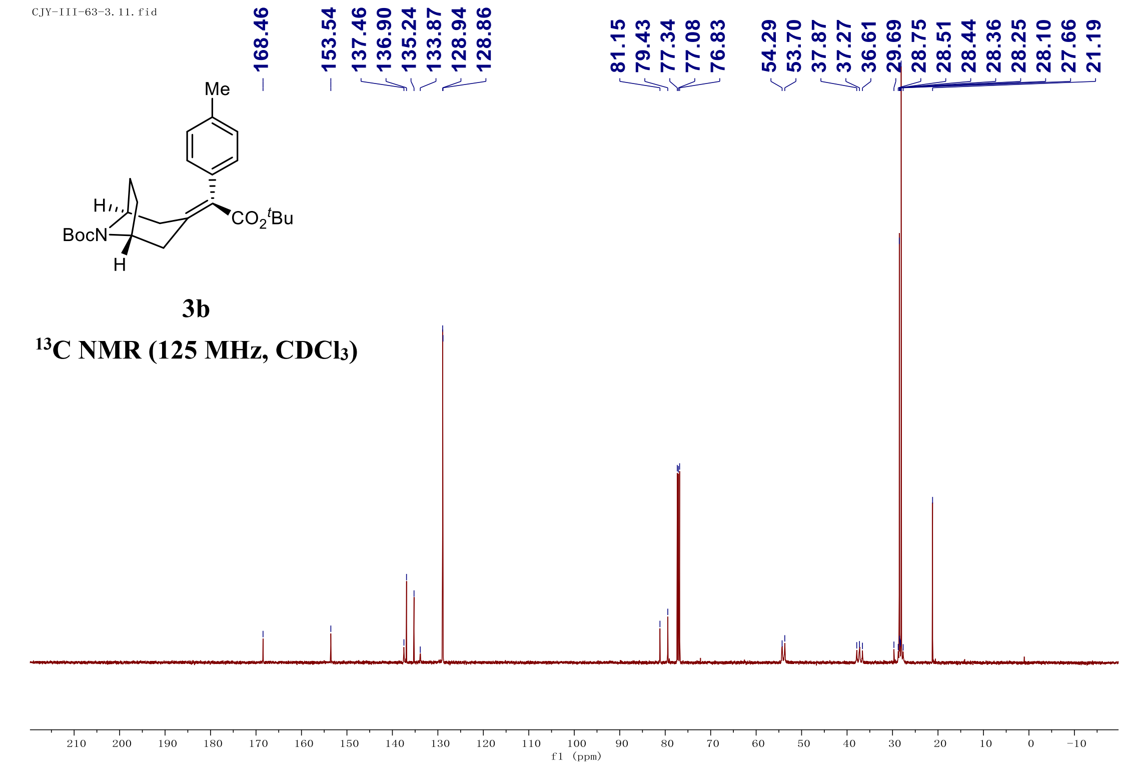

Figure S31 ^{13}C NMR (125 MHz, $CDCl_3$) spectrum for **3b**

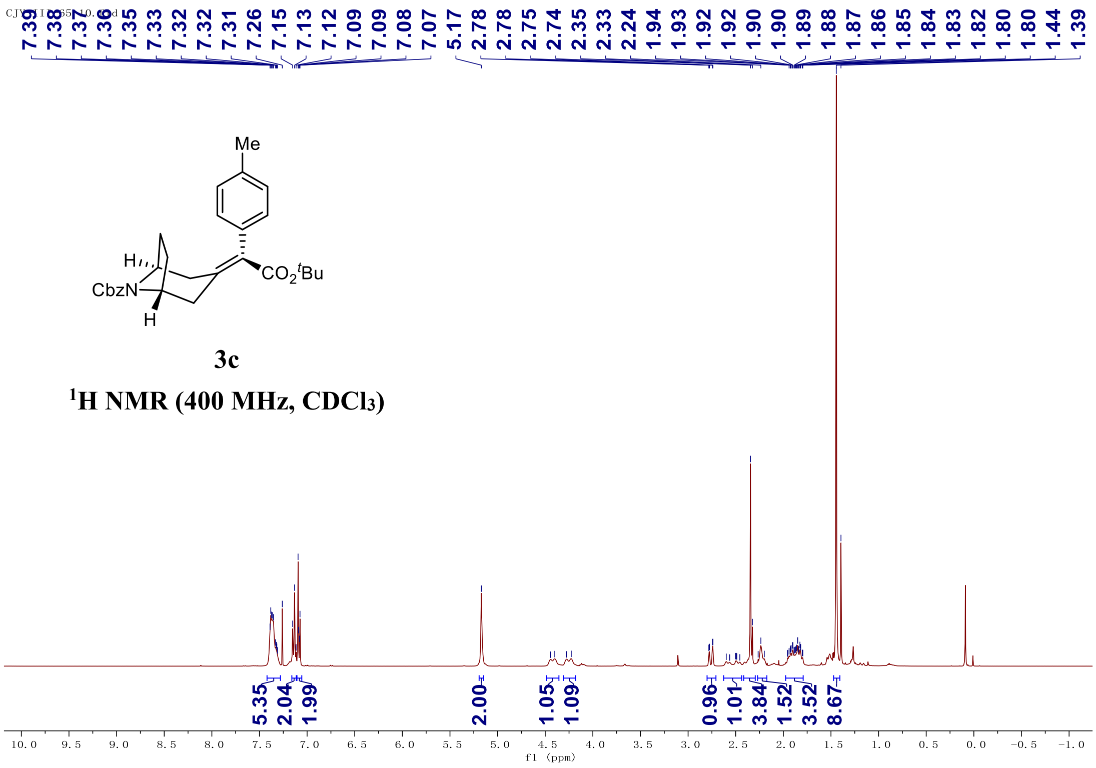

Figure S32 ^{1}H NMR (400 MHz, CDCl$_3$) spectrum for **3c**

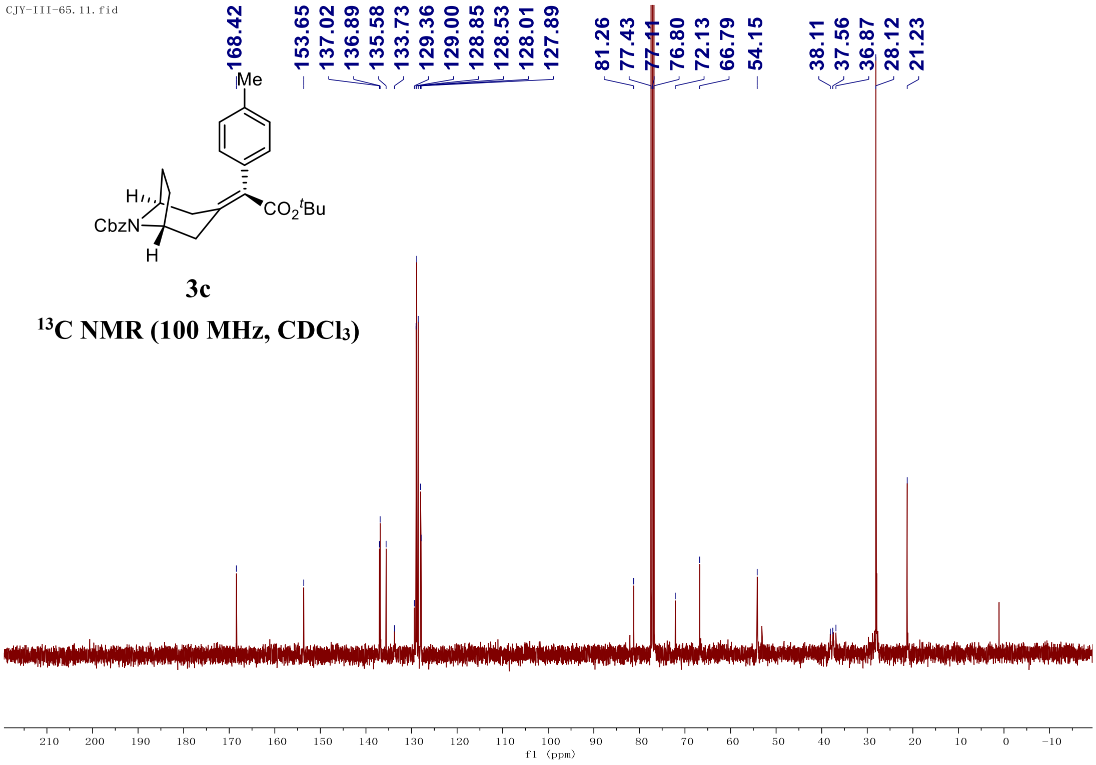

Figure S33 ^{13}C NMR (100 MHz, CDCl$_3$) spectrum for **3c**

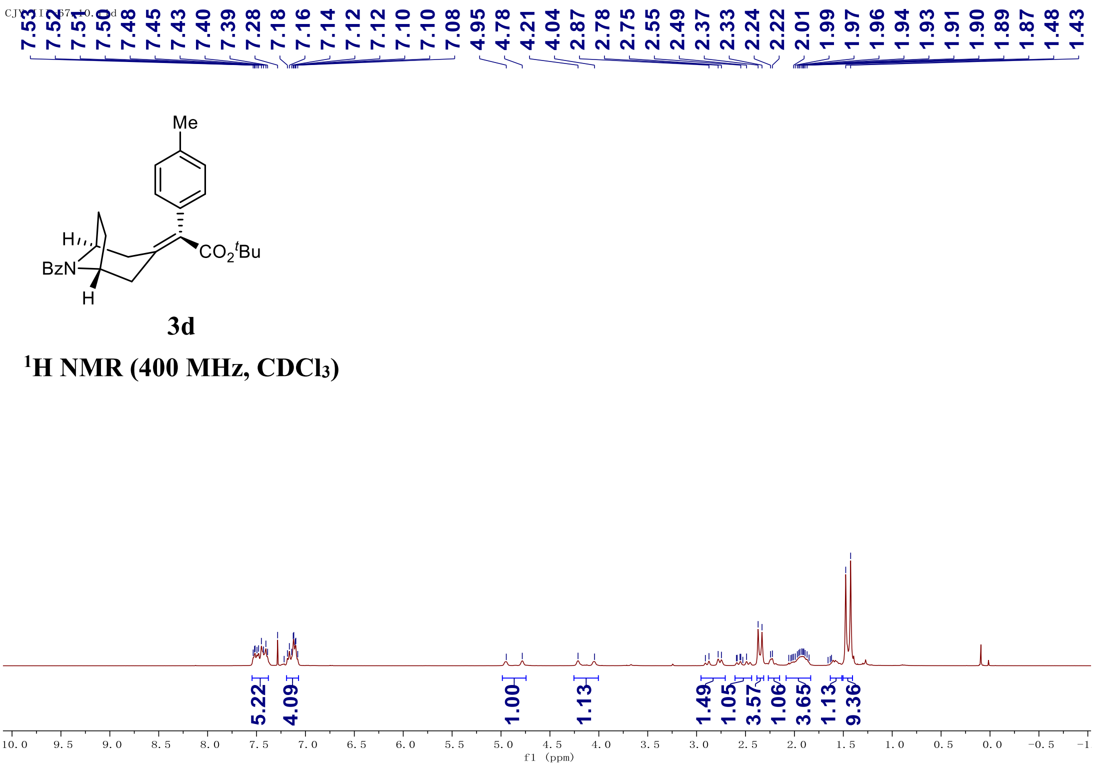

Figure S34 ^{1}H NMR (400 MHz, $CDCl_3$) spectrum for **3d**

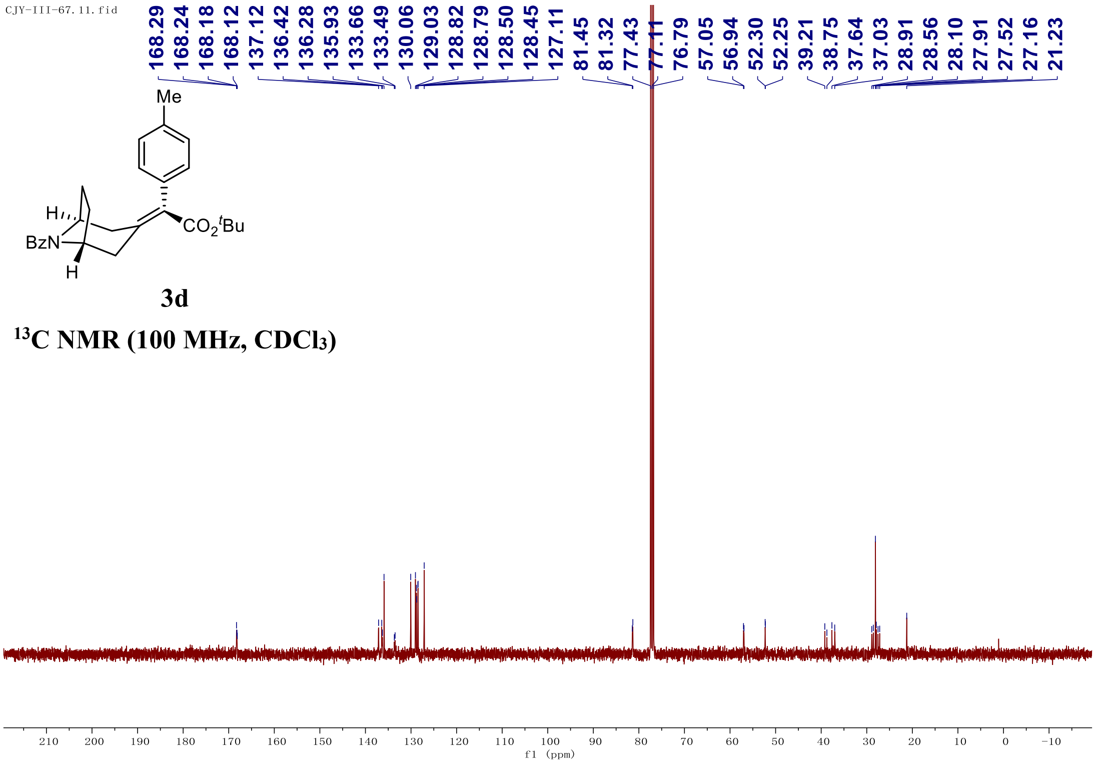

Figure S35 ^{13}C NMR (100 MHz, $CDCl_3$) spectrum for **3d**

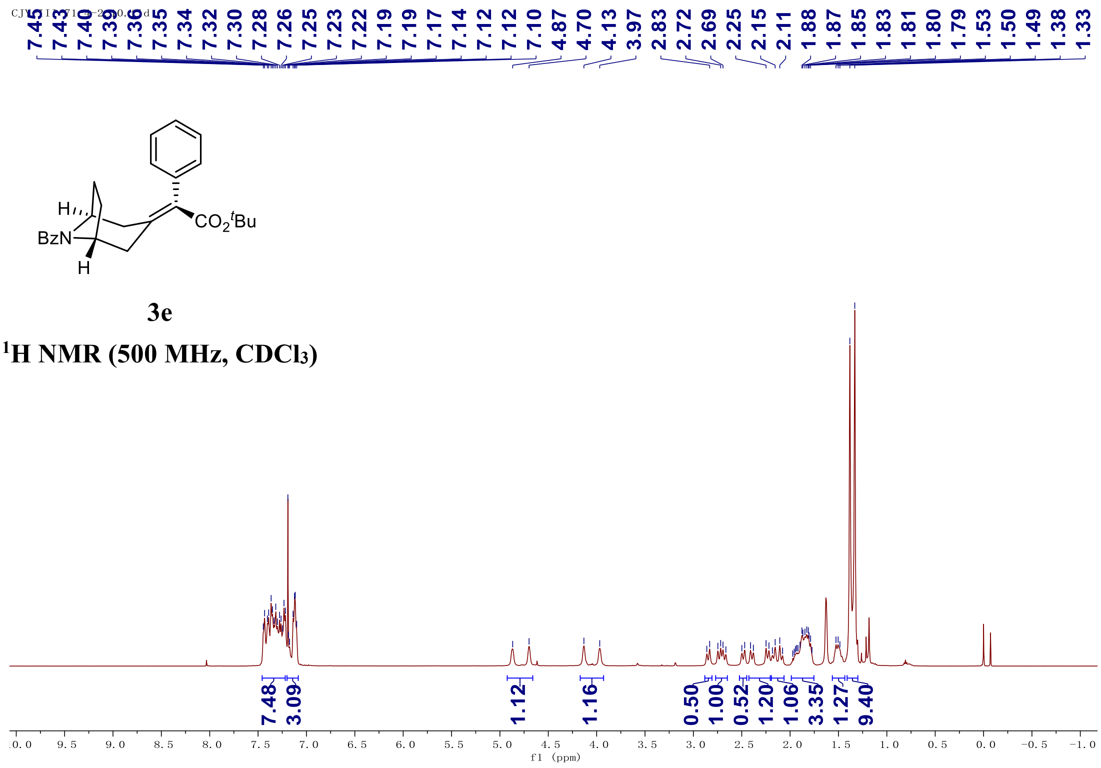

Figure S36 ^1H NMR (500 MHz, $CDCl_3$) spectrum for **3e**

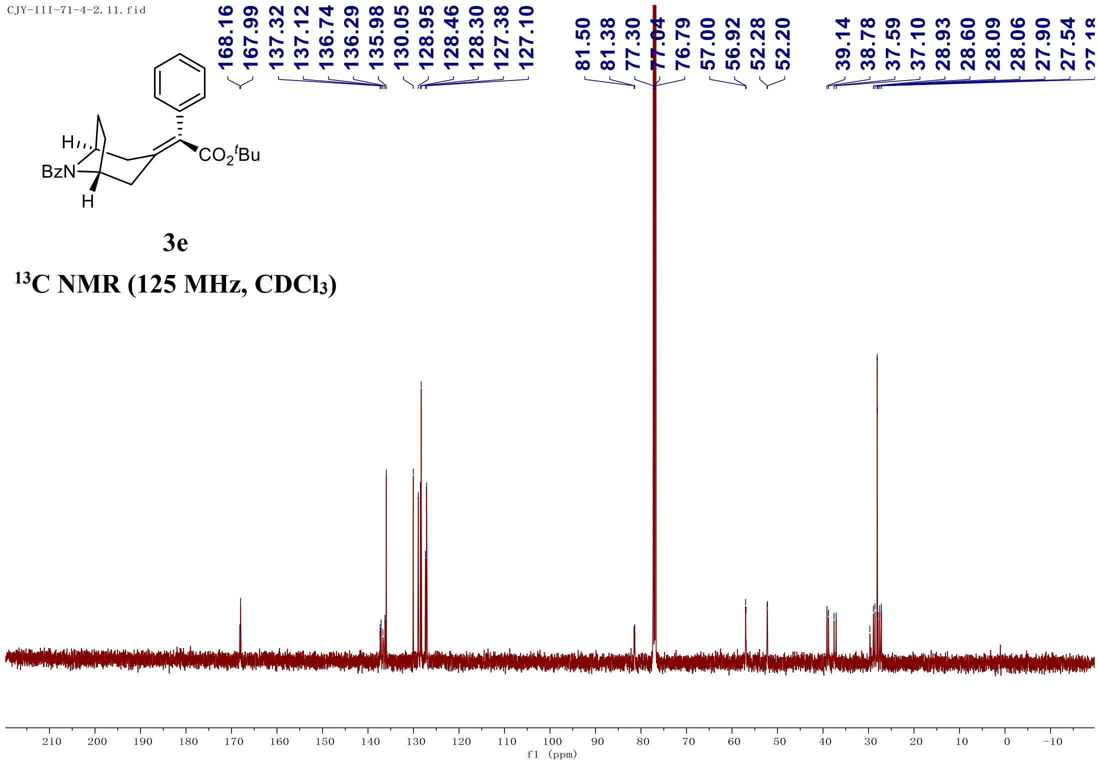

Figure S37 ^{13}C NMR (125 MHz, $CDCl_3$) spectrum for **3e**

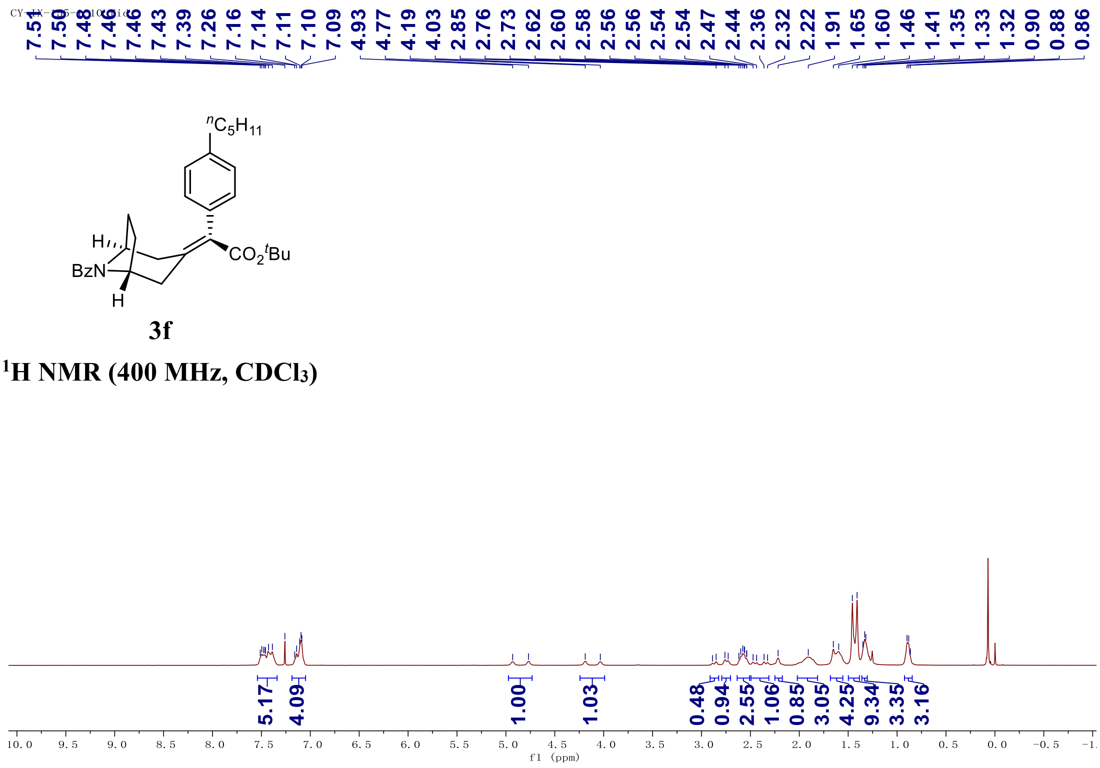

Figure S38 ^{1}H NMR (400 MHz, $CDCl_3$) spectrum for **3f**

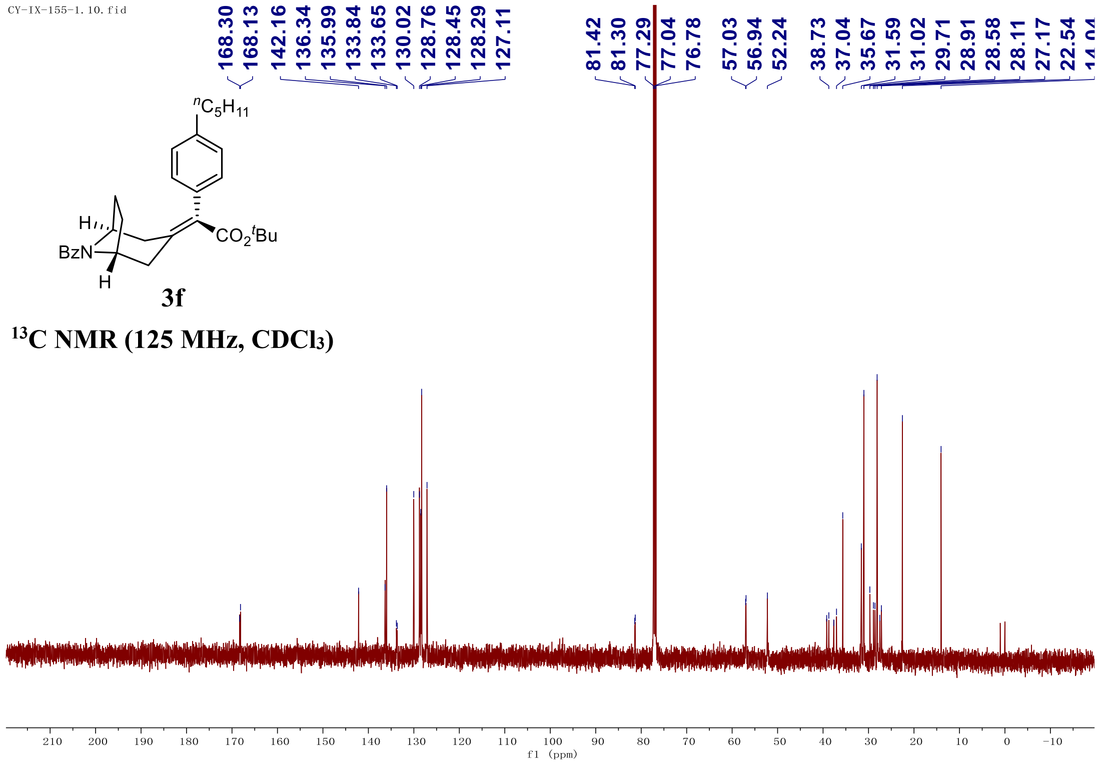

Figure S39 ^{13}C NMR (125 MHz, $CDCl_3$) spectrum for **3f**

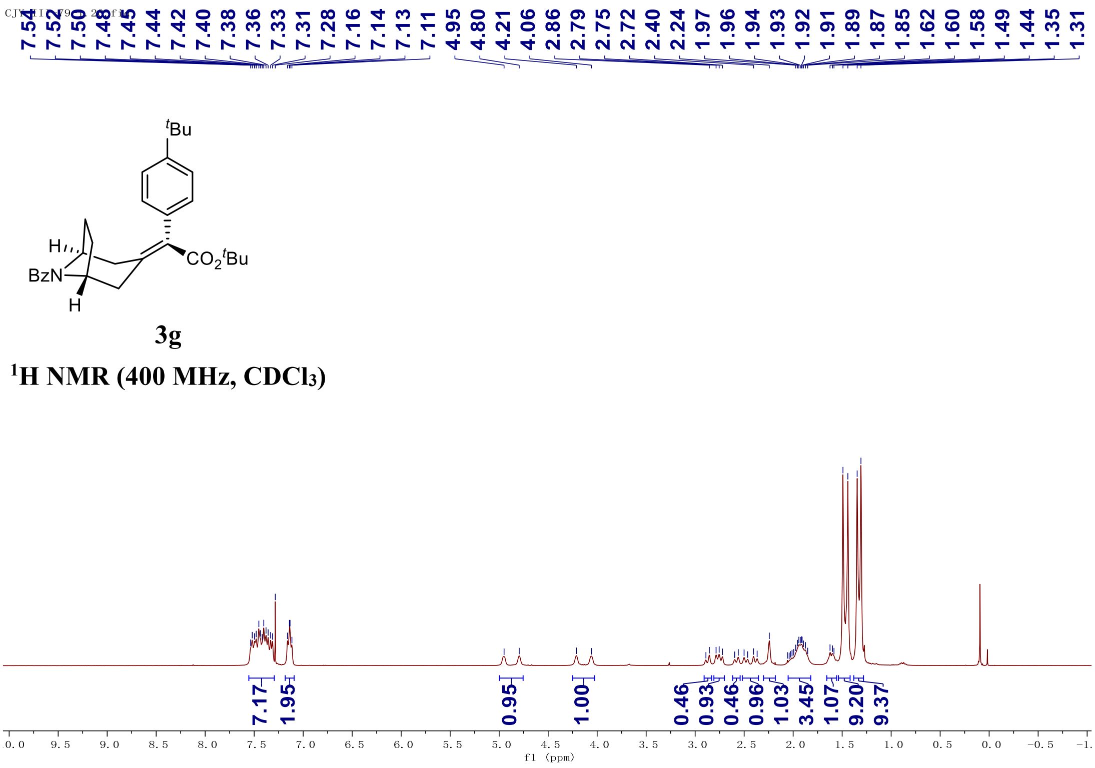

Figure S40 ^{1}H NMR (400 MHz, $CDCl_3$) spectrum for **3g**

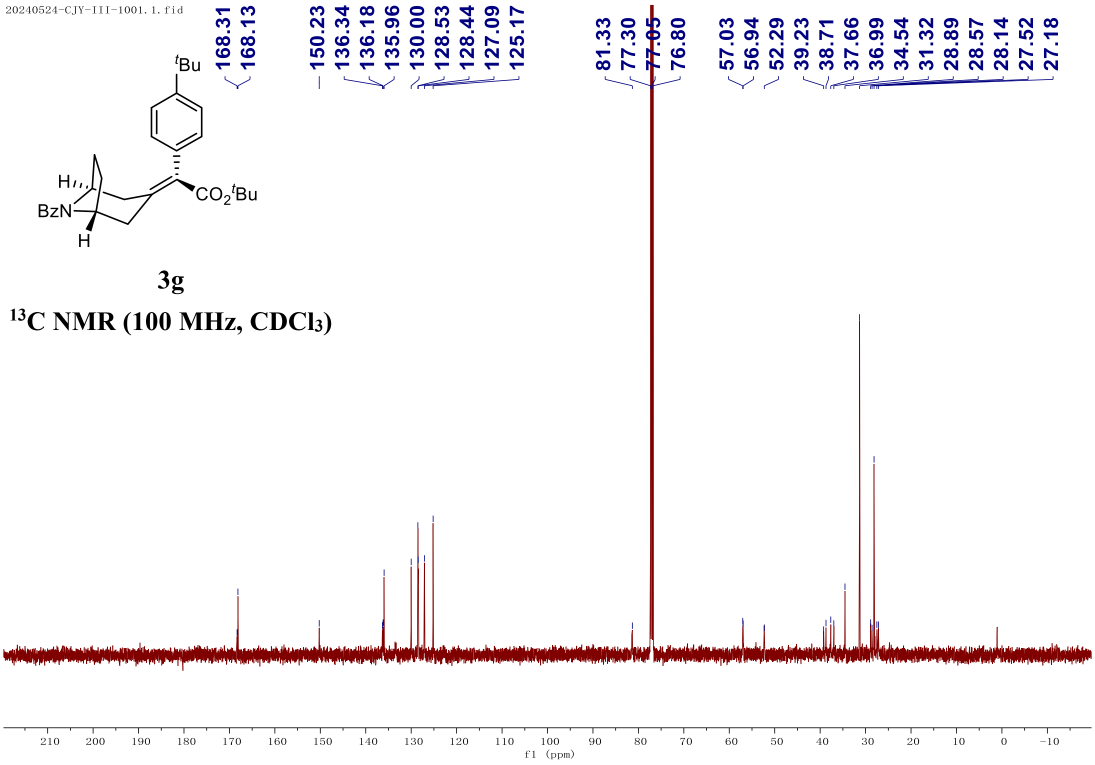

Figure S41 ^{13}C NMR (100 MHz, $CDCl_3$) spectrum for **3g**

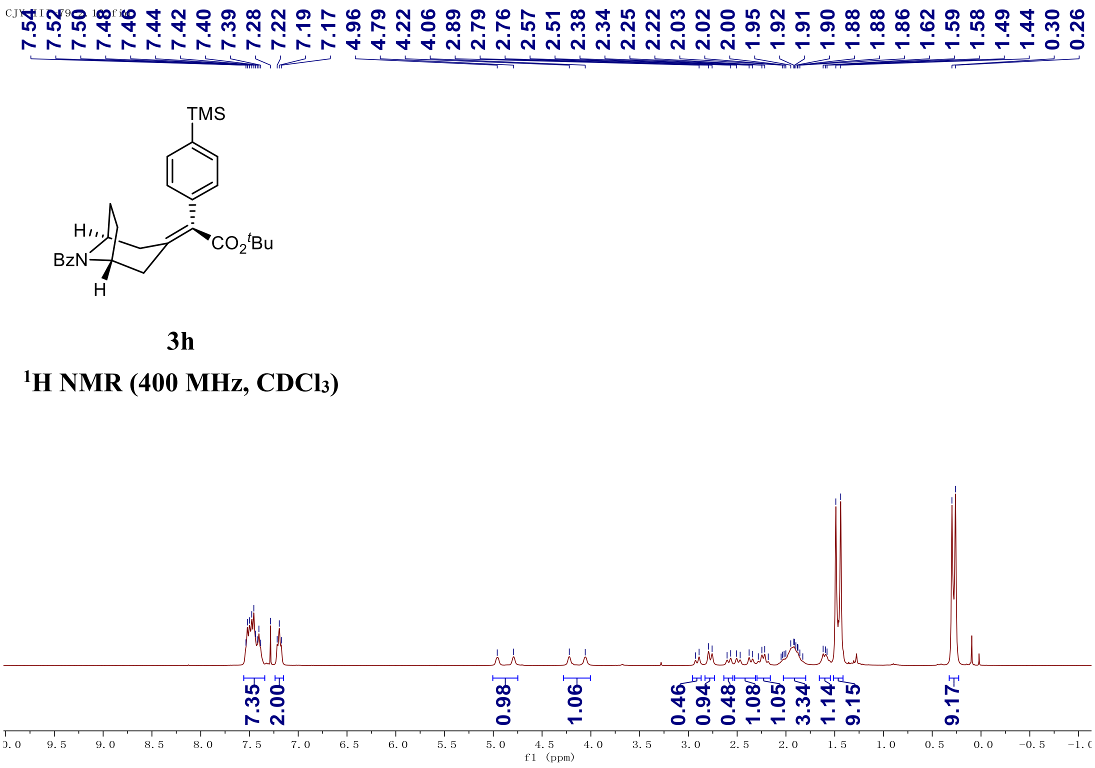

Figure S42 ^{1}H NMR (400 MHz, $CDCl_3$) spectrum for **3h**

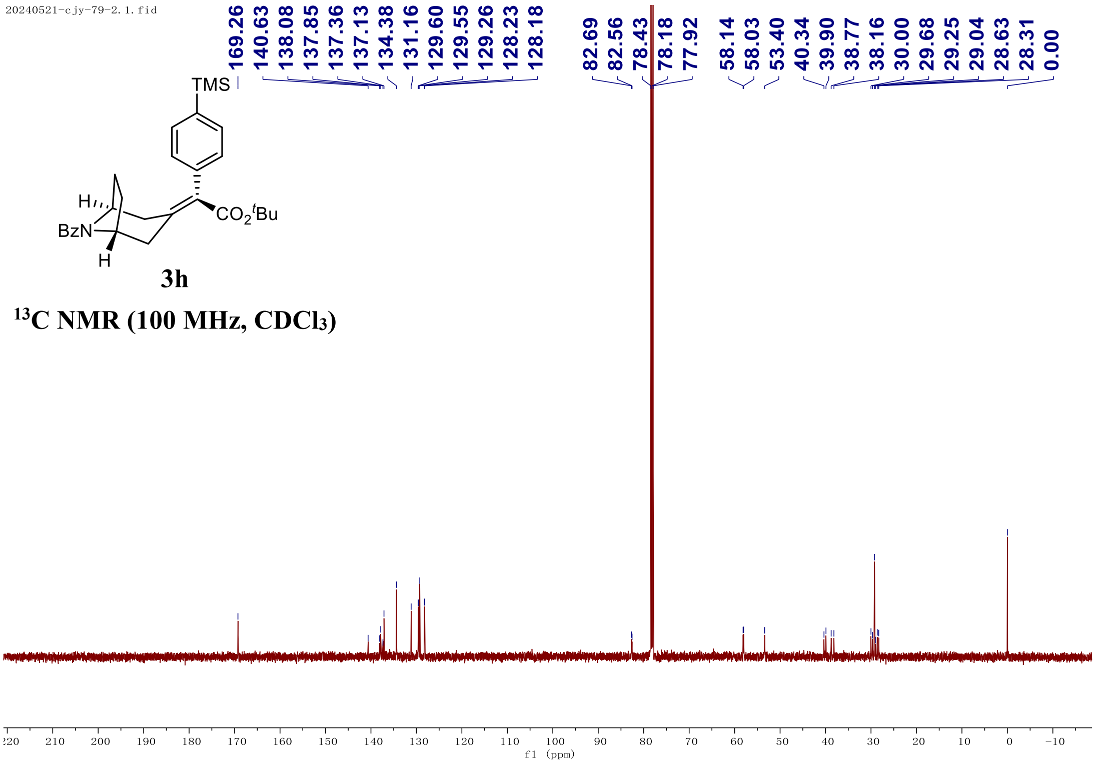

Figure S43 ^{13}C NMR (100 MHz, $CDCl_3$) spectrum for **3h**

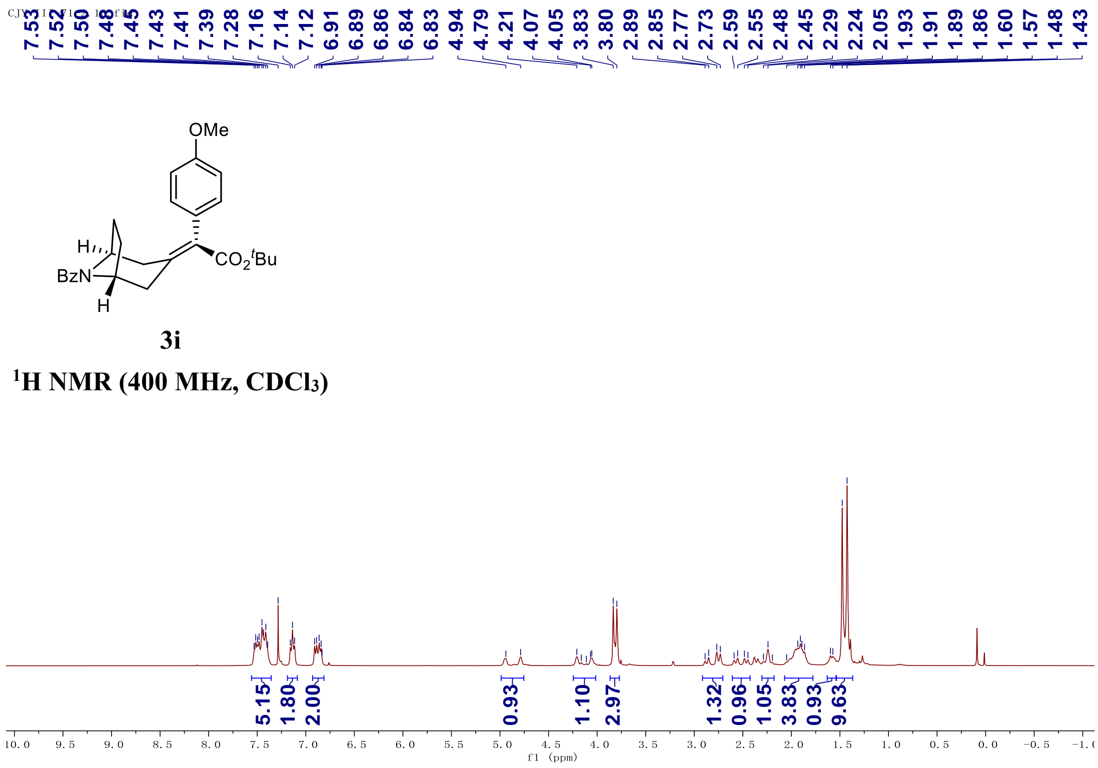

Figure S44 ^{1}H NMR (400 MHz, $CDCl_3$) spectrum for **3i**

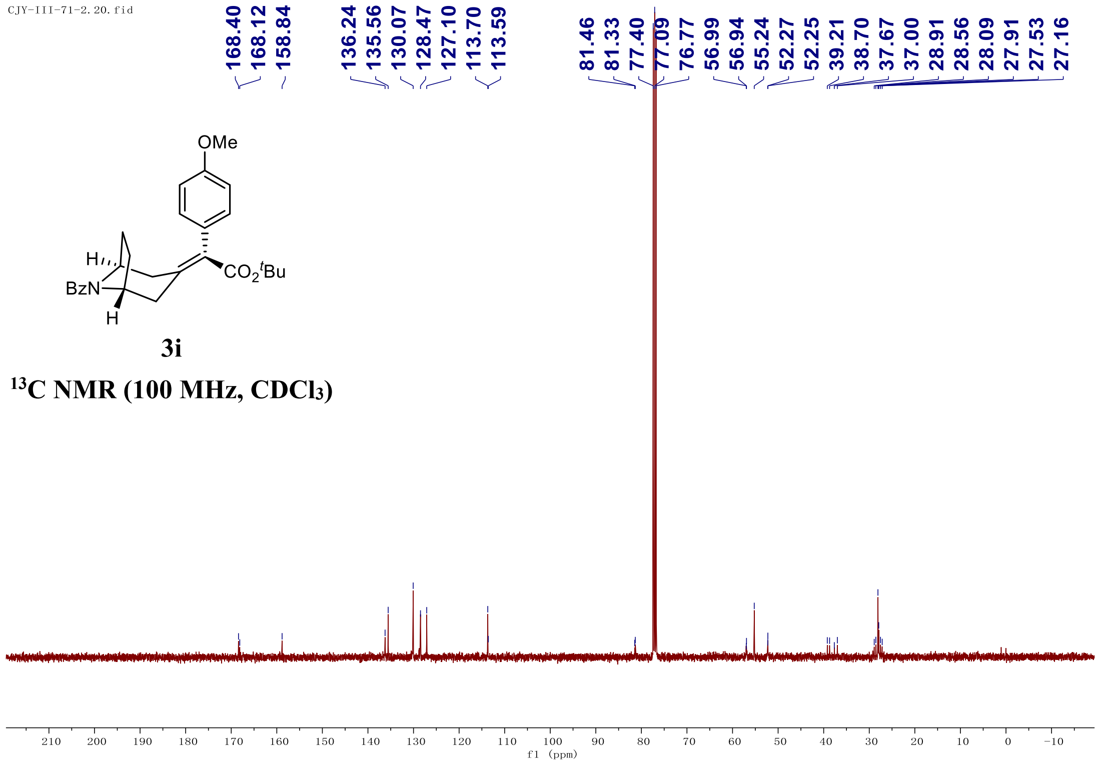

Figure S45 ^{13}C NMR (100 MHz, $CDCl_3$) spectrum for **3i**

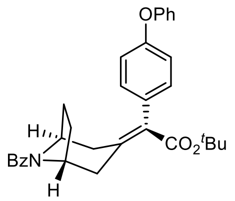

3j

^{1}H NMR (400 MHz, $CDCl_3$)

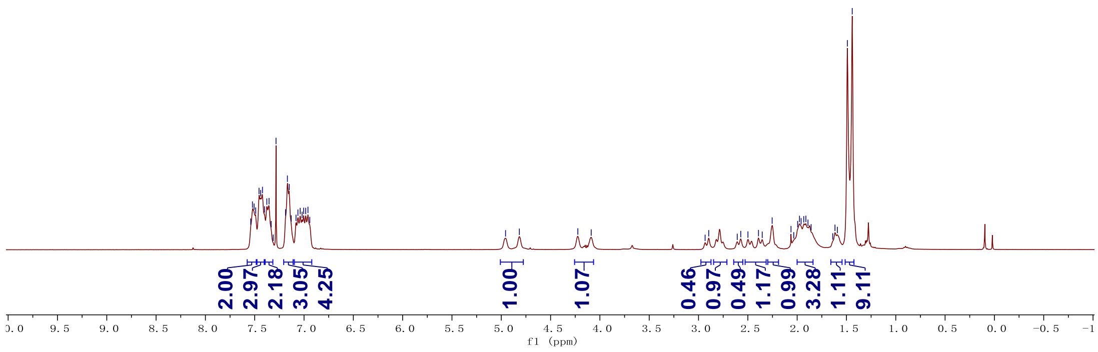

Figure S46 ^{1}H NMR (400 MHz, $CDCl_3$) spectrum for **3j**

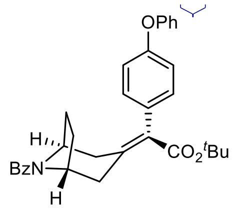

3j

^{13}C NMR (100 MHz, $CDCl_3$)

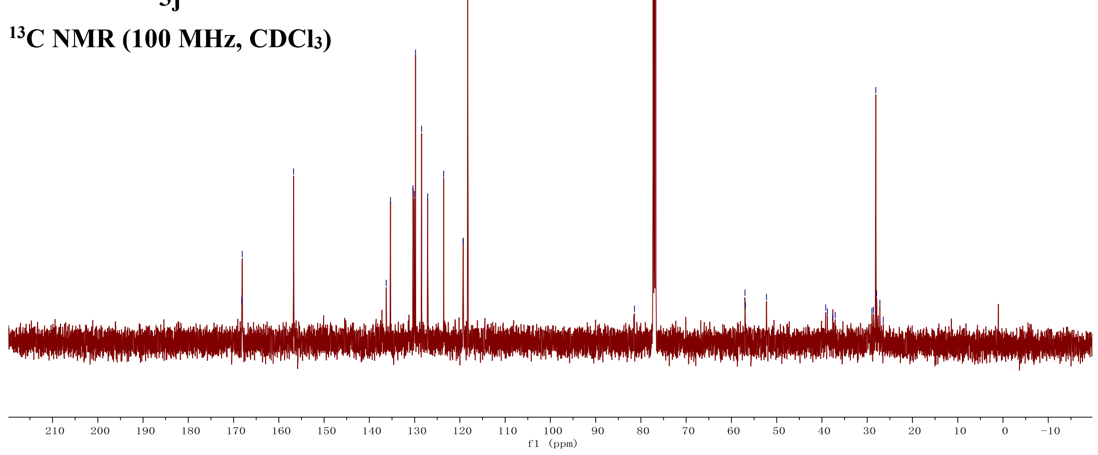

Figure S47 ^{13}C NMR (100 MHz, $CDCl_3$) spectrum for **3j**

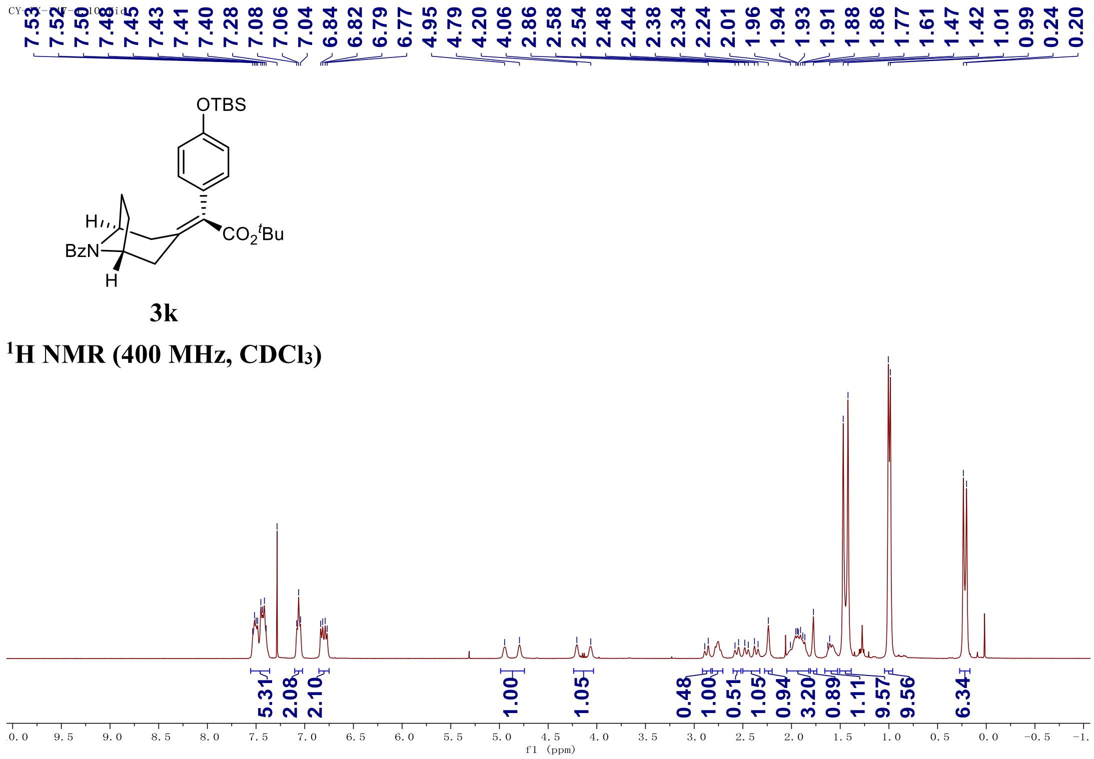

Figure S48 ^{1}H NMR (400 MHz, $CDCl_3$) spectrum for **3k**

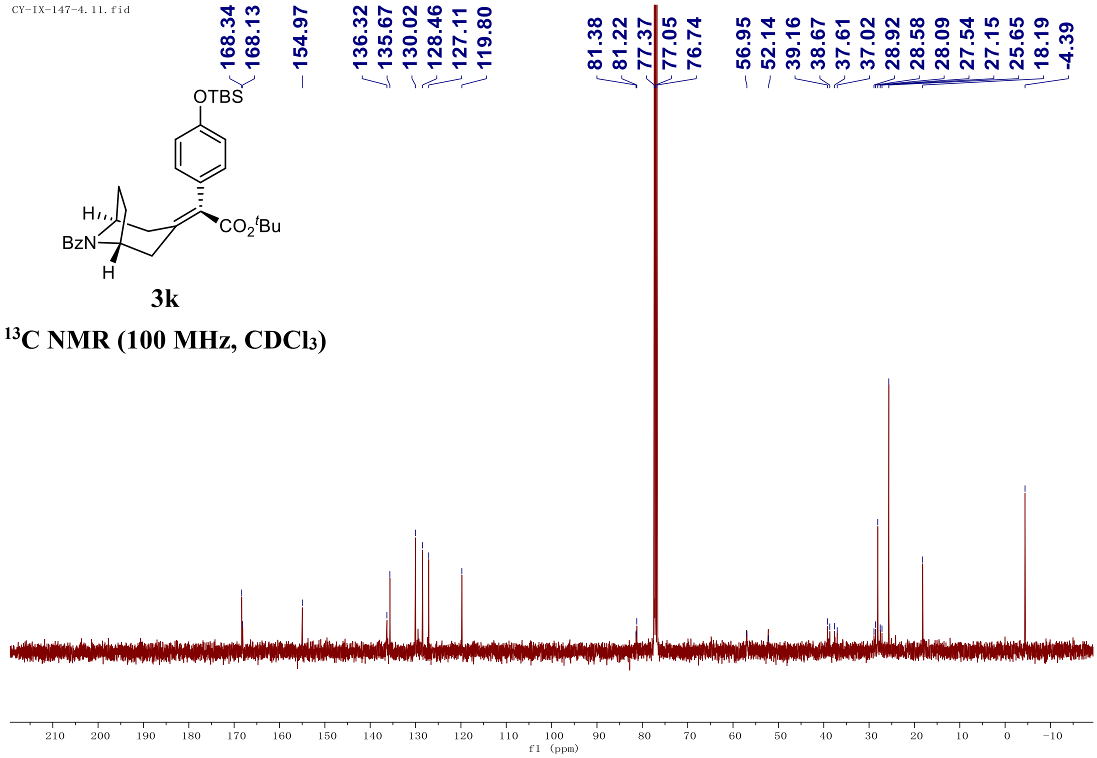

Figure S49 ^{13}C NMR (100 MHz, $CDCl_3$) spectrum for **3k**

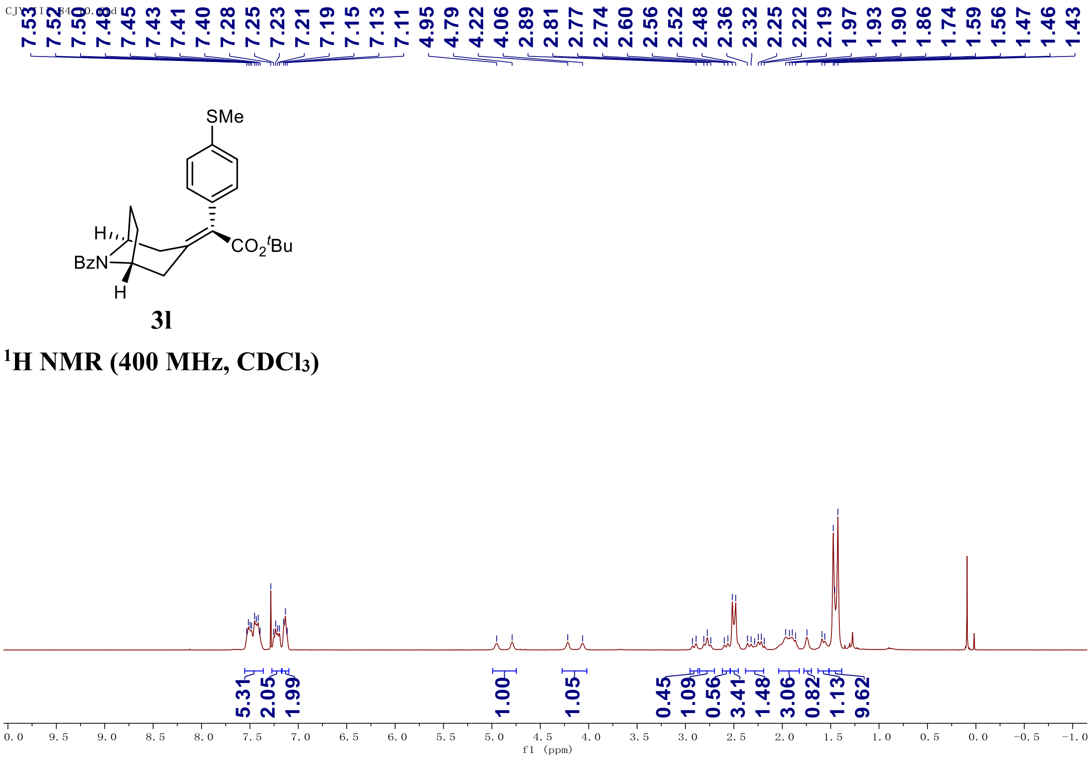

Figure S50 ^{1}H NMR (400 MHz, $CDCl_3$) spectrum for **3l**

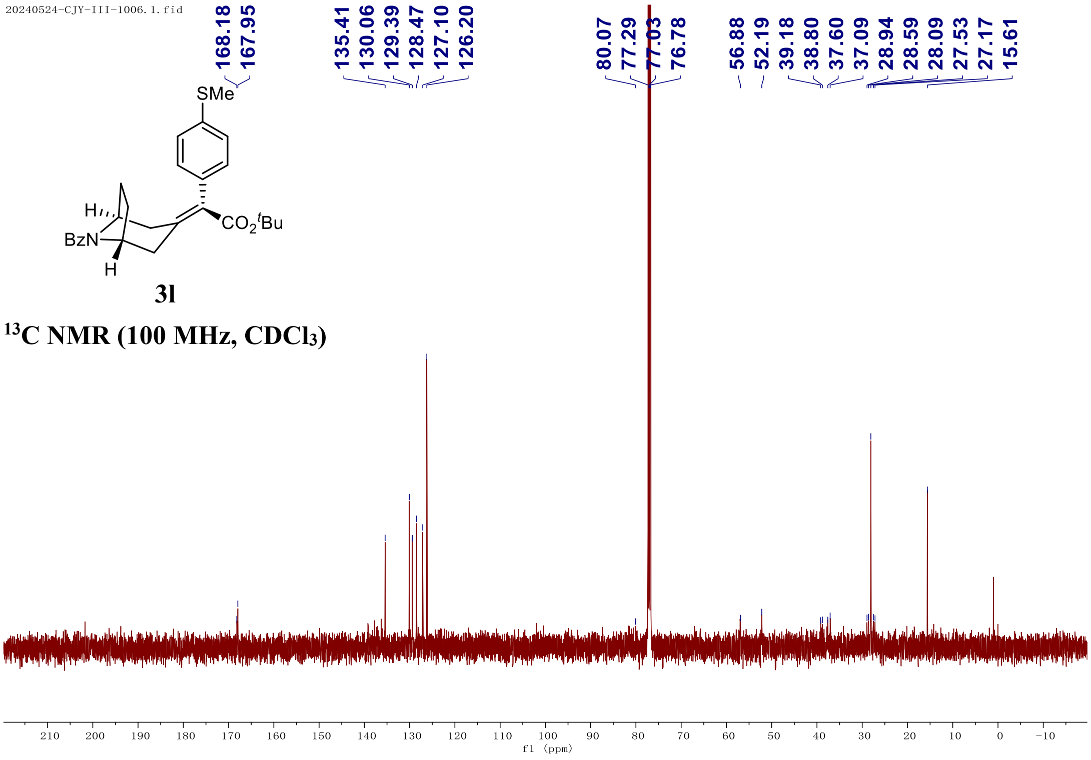

Figure S51 ^{13}C NMR (100 MHz, $CDCl_3$) spectrum for **3l**

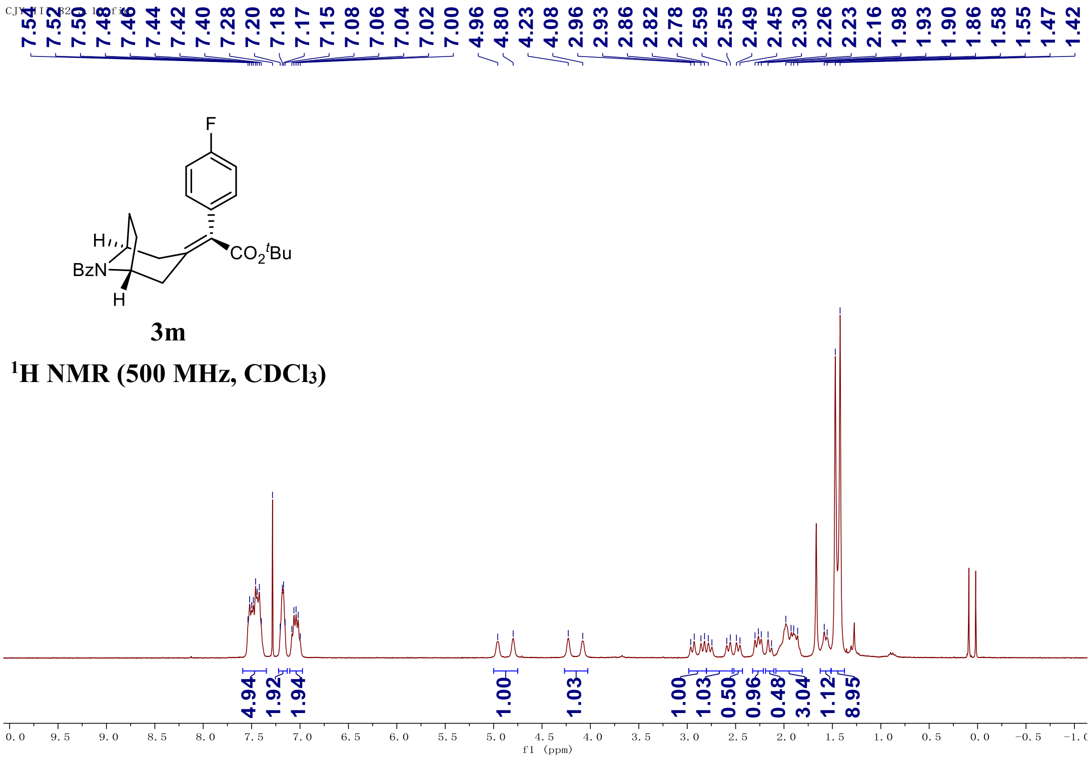

Figure S52 ^{1}H NMR (500 MHz, $CDCl_3$) spectrum for **3m**

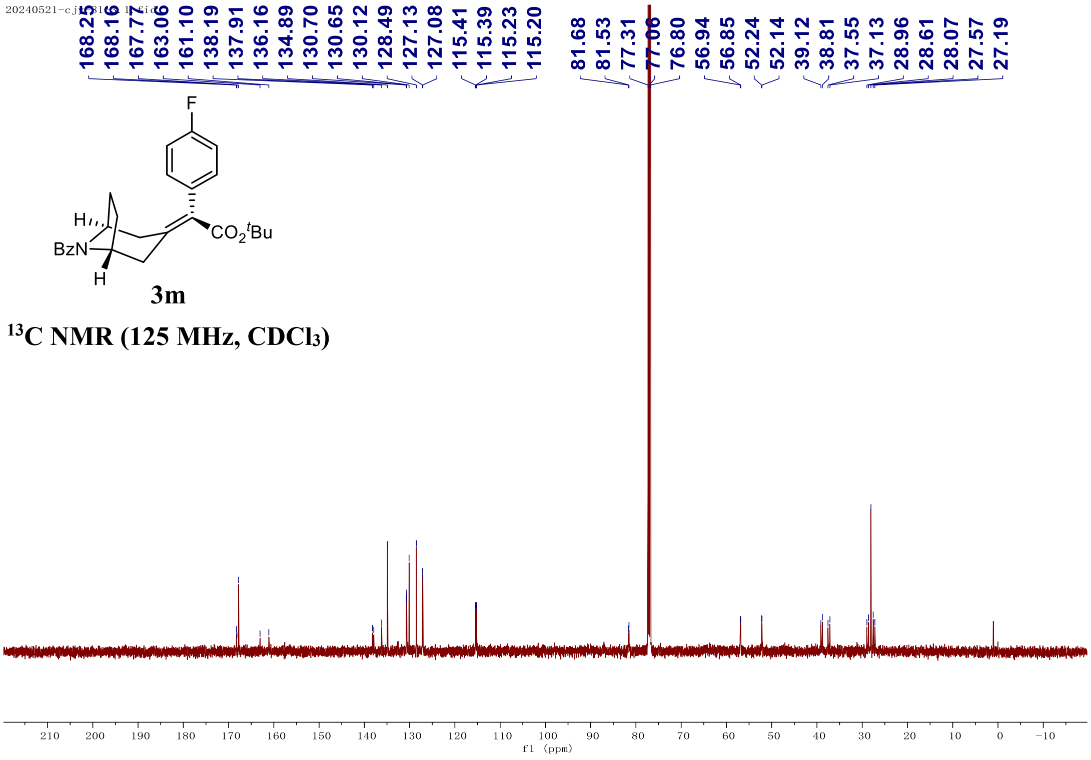

Figure S53 ^{13}C NMR (125 MHz, $CDCl_3$) spectrum for **3m**

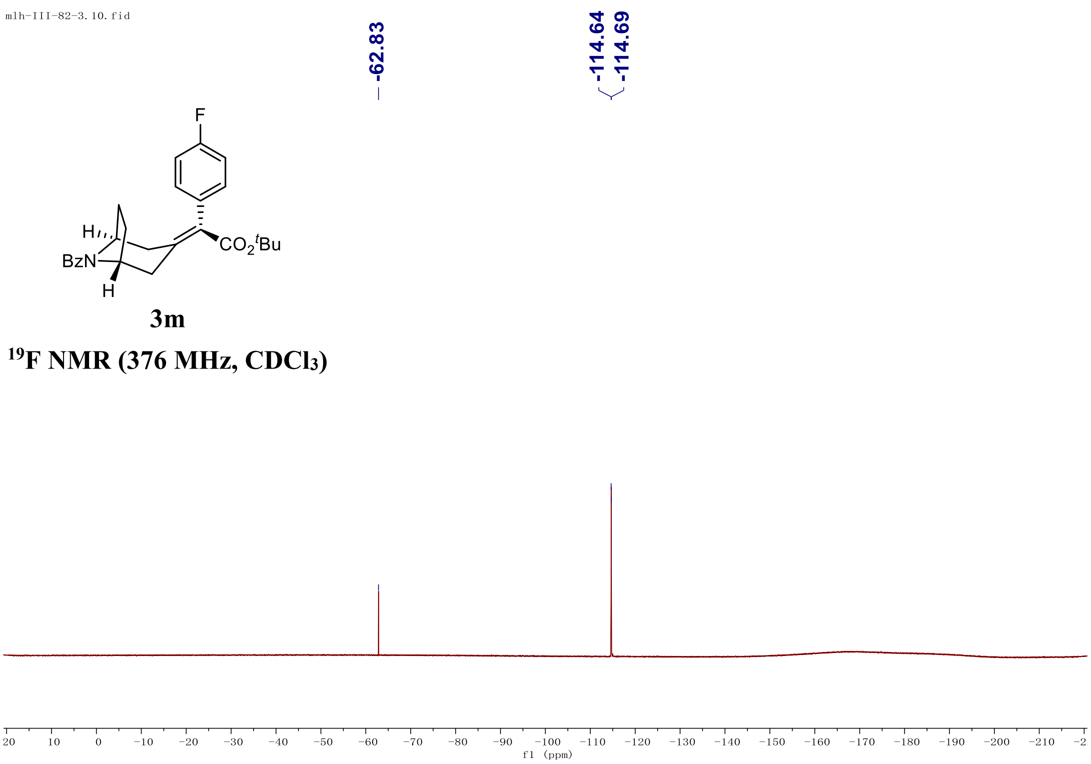

Figure S54 ^{19}F NMR (376 MHz, $CDCl_3$) spectrum for **3m**

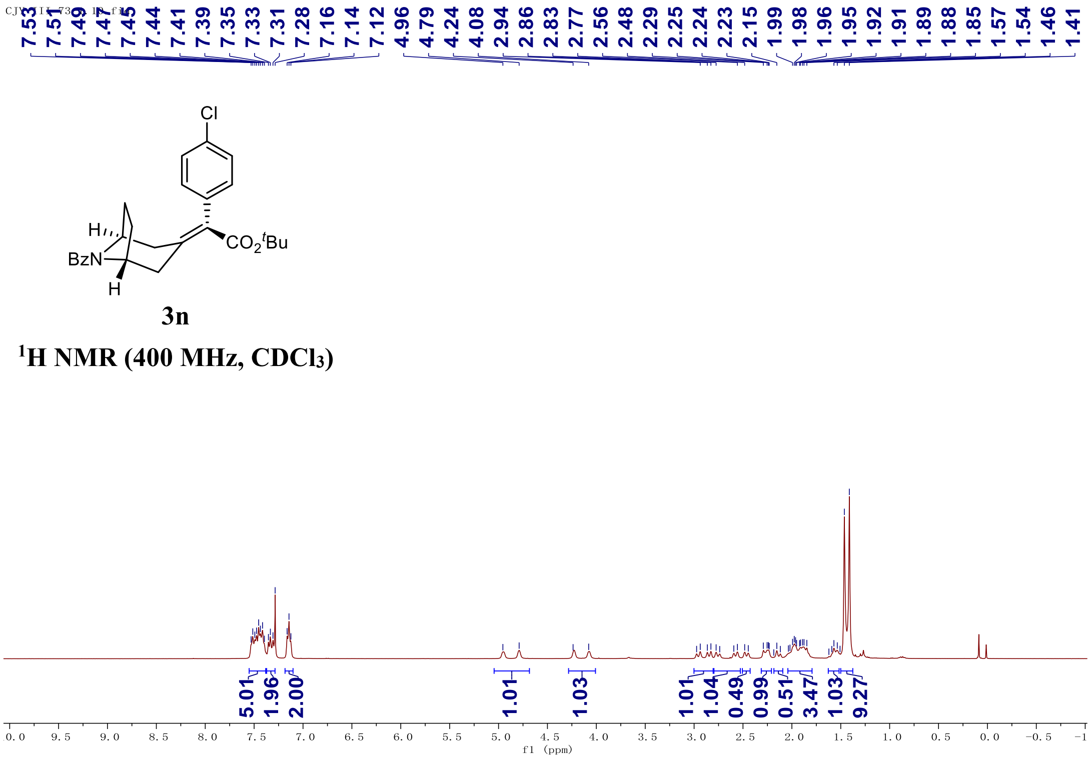

Figure S55 ^{1}H NMR (400 MHz, $CDCl_3$) spectrum for **3n**

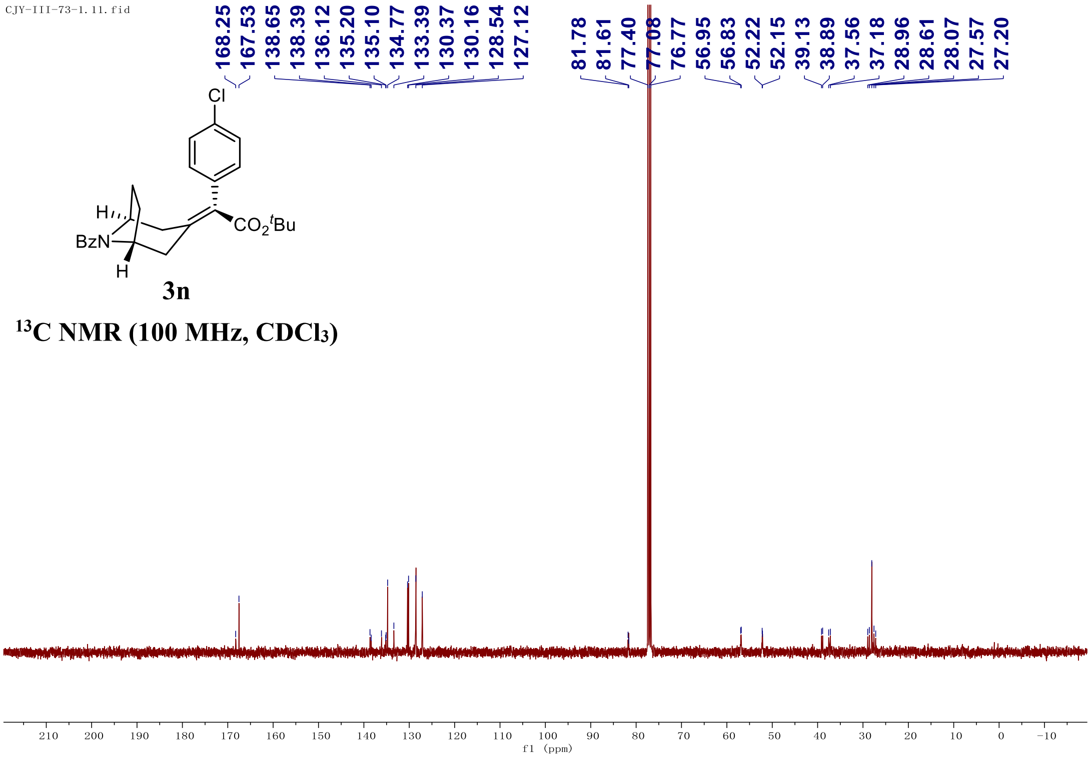

Figure S56 ^{13}C NMR (100 MHz, $CDCl_3$) spectrum for **3n**

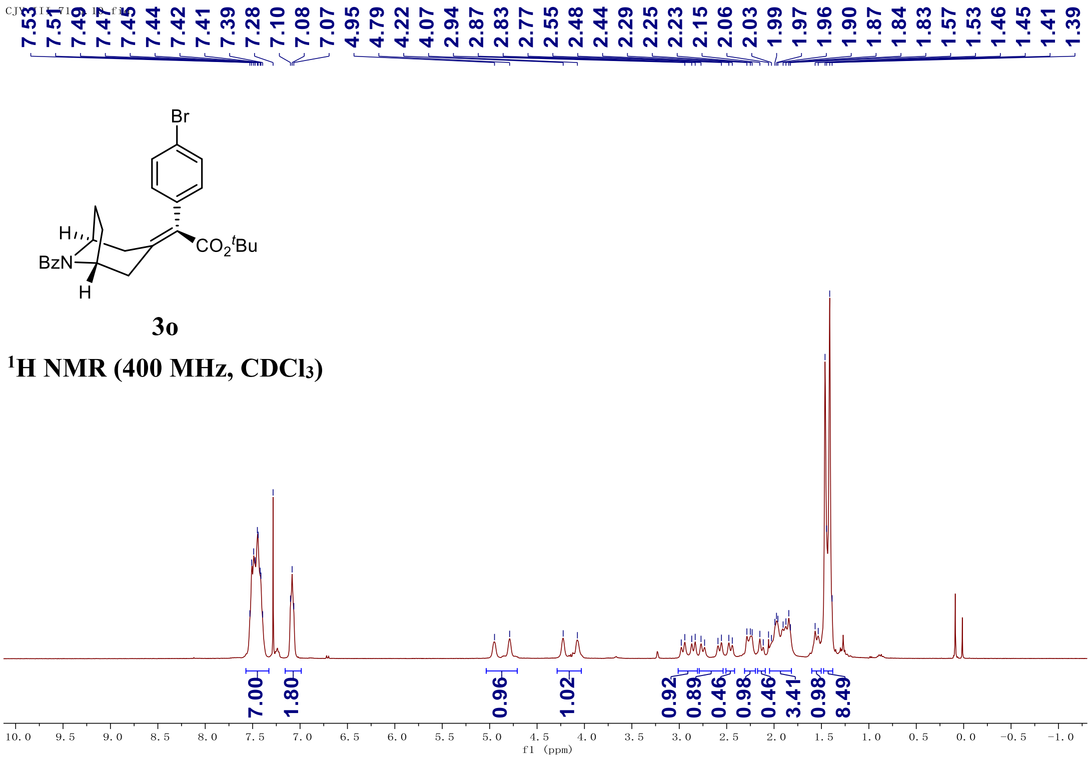

Figure S57 ^{1}H NMR (400 MHz, $CDCl_3$) spectrum for **3o**

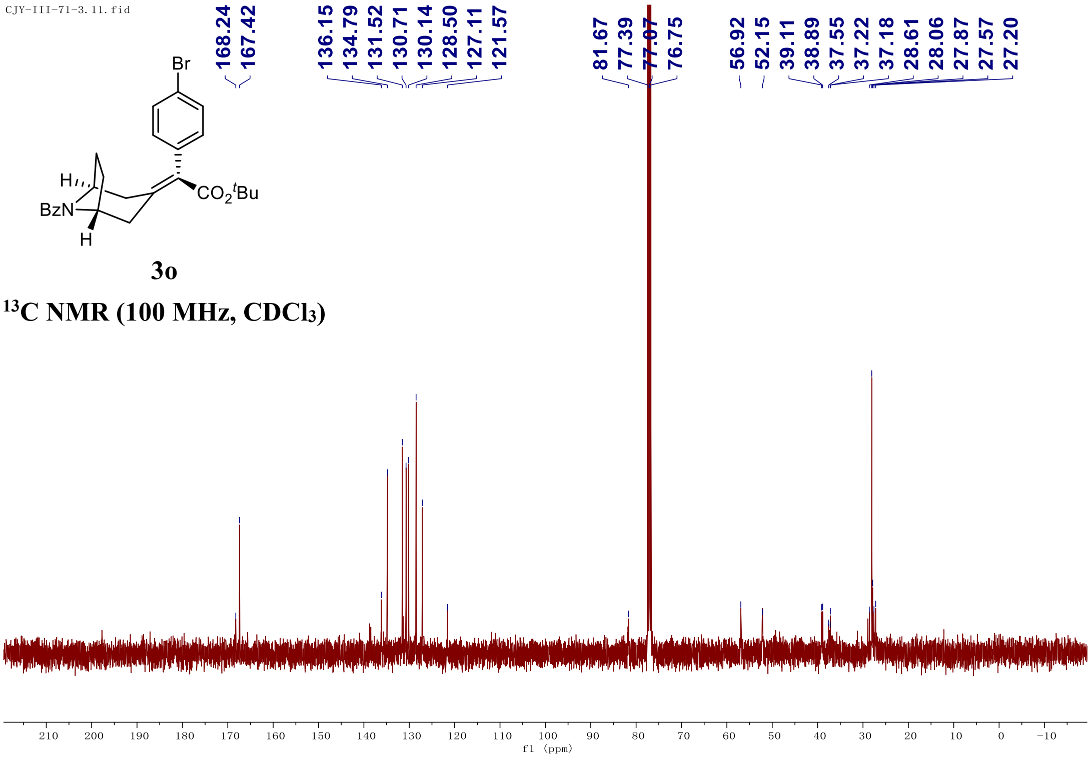

Figure S58 ^{13}C NMR (100 MHz, $CDCl_3$) spectrum for **3o**

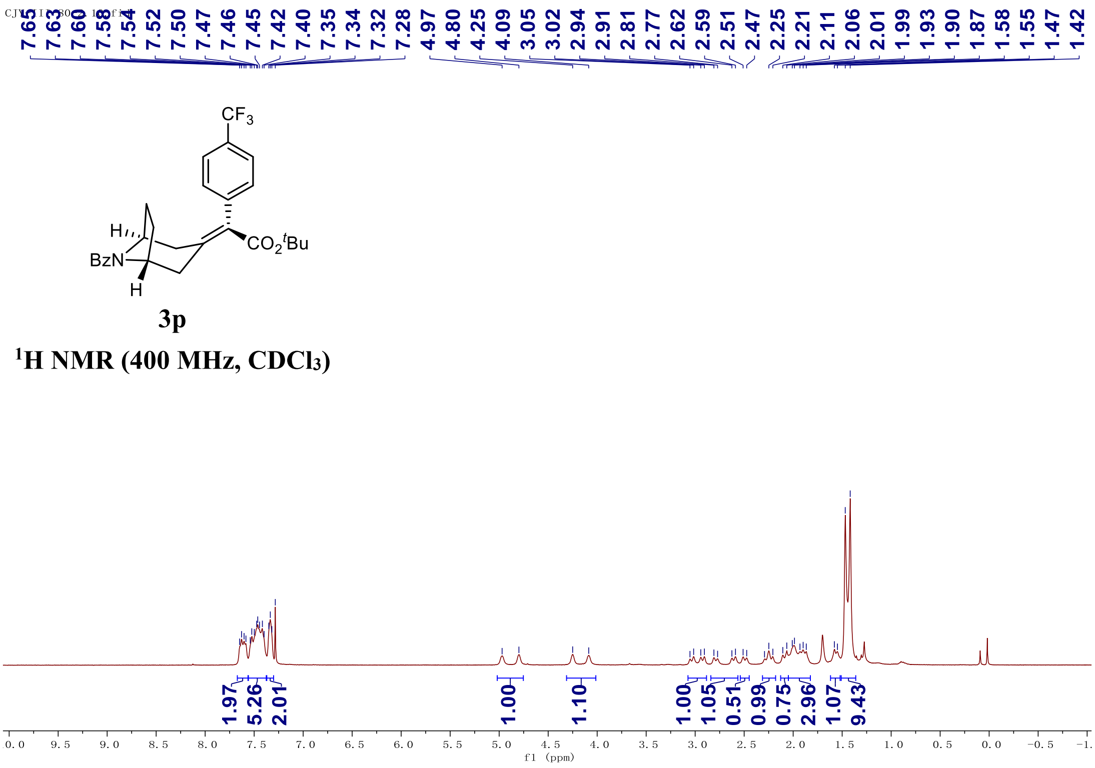

Figure S59 ^{1}H NMR (400 MHz, $CDCl_3$) spectrum for **3p**

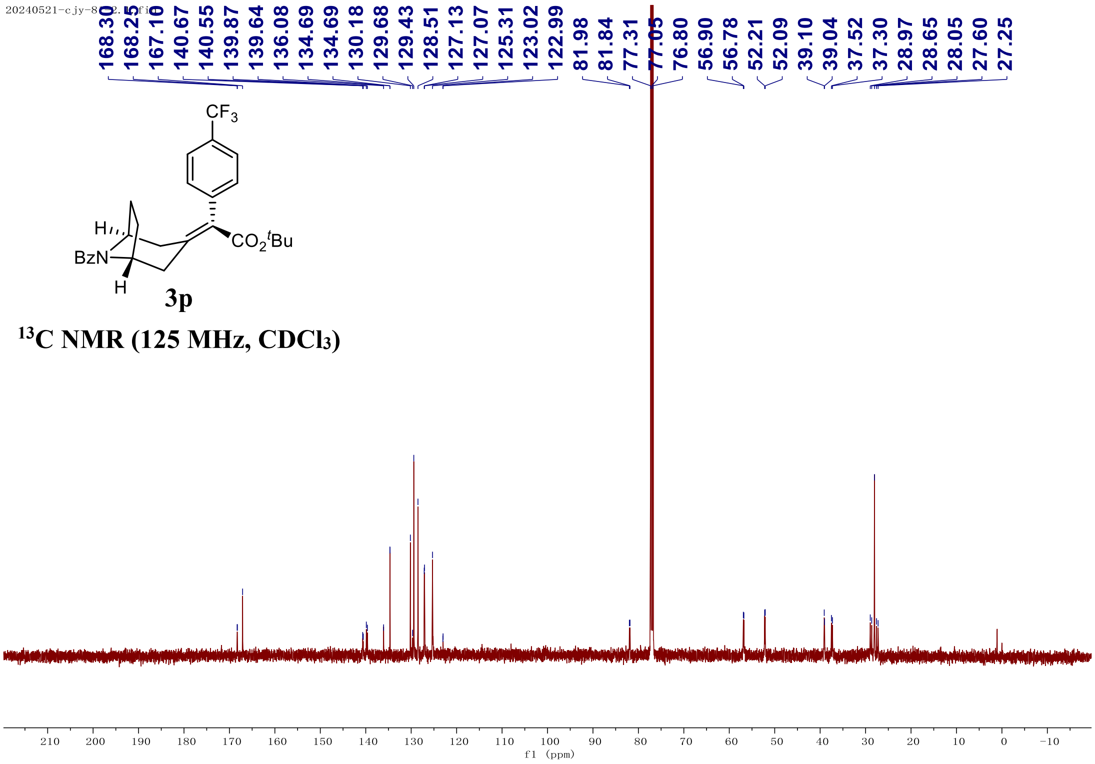

Figure S60 ^{13}C NMR (125 MHz, $CDCl_3$) spectrum for **3p**

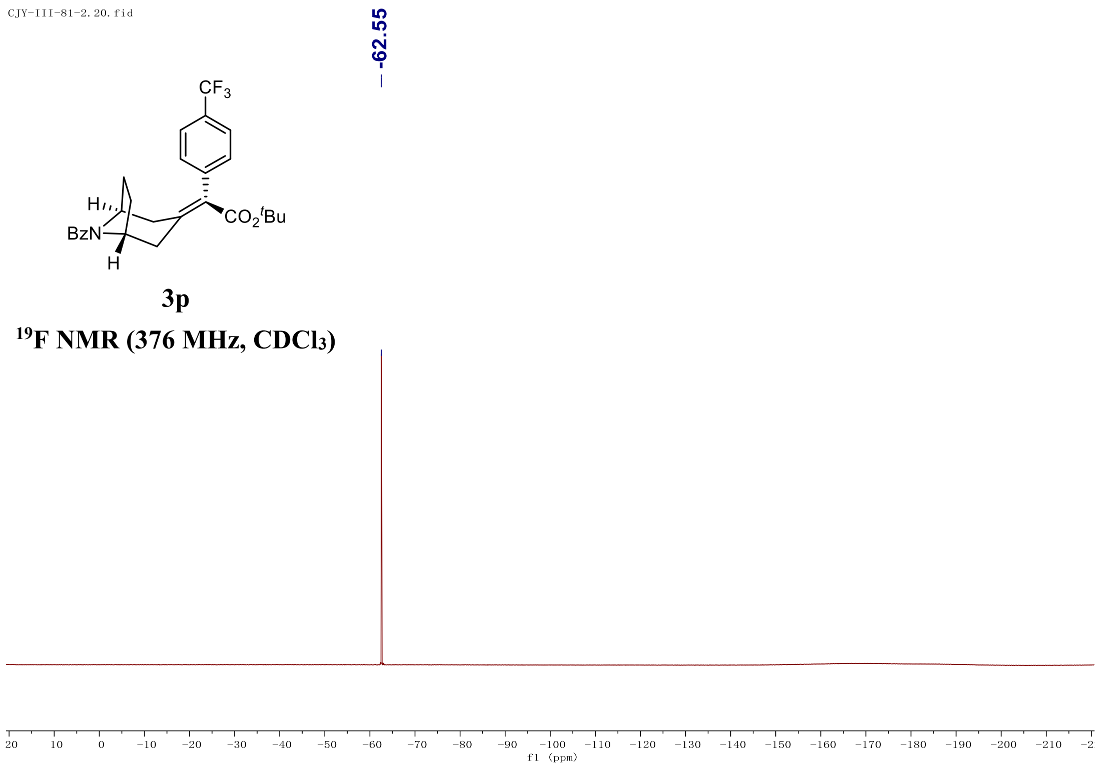

Figure S61 ^{19}F NMR (376 MHz, $CDCl_3$) spectrum for **3p**

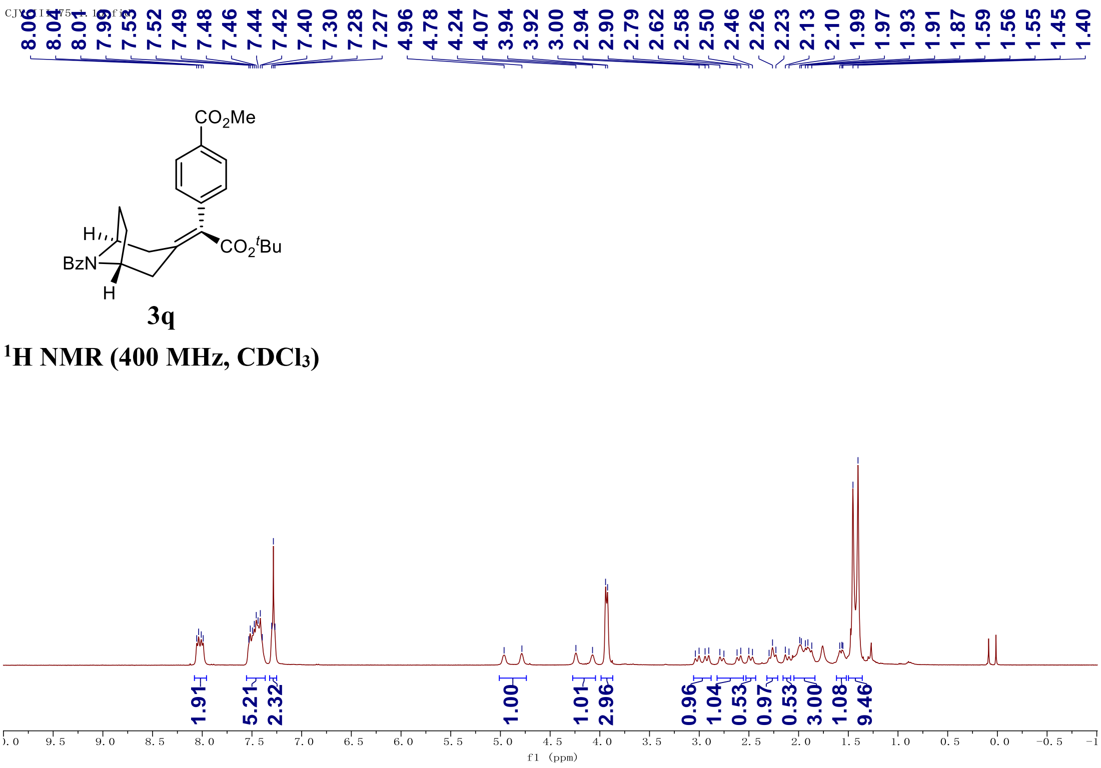

Figure S62 ^{1}H NMR (400 MHz, CDCl$_3$) spectrum for **3q**

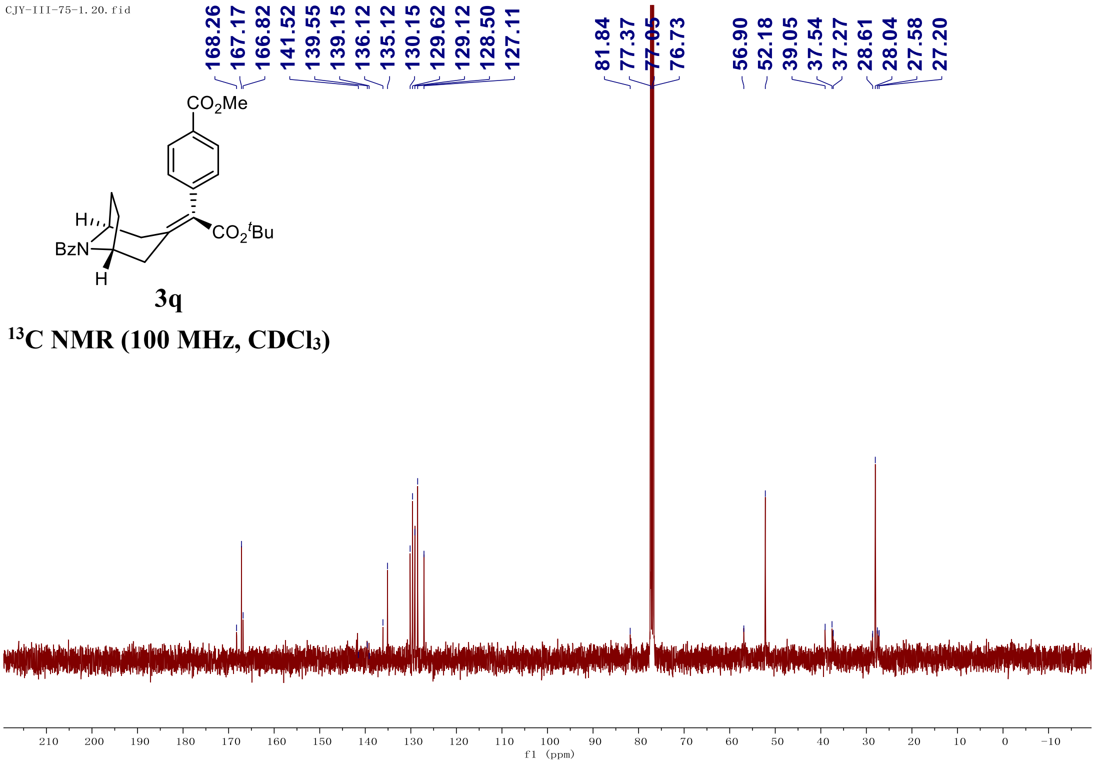

Figure S63 ^{13}C NMR (100 MHz, CDCl$_3$) spectrum for **3q**

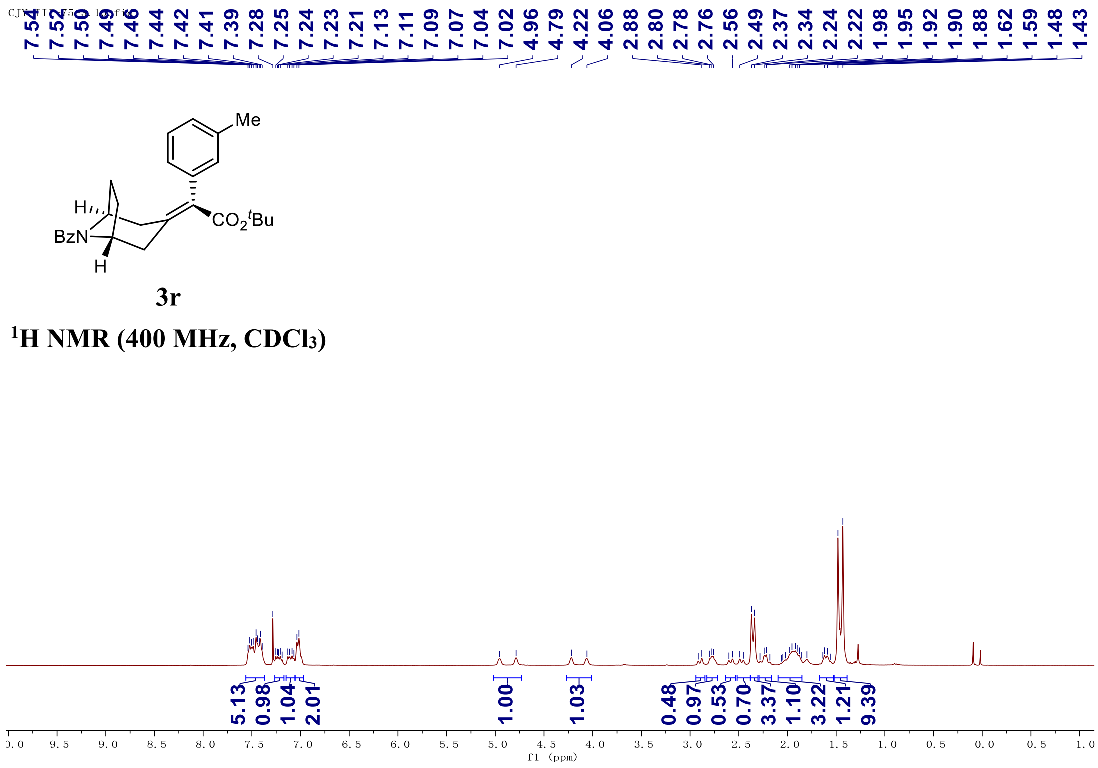

Figure S64 ^{1}H NMR (400 MHz, $CDCl_3$) spectrum for **3r**

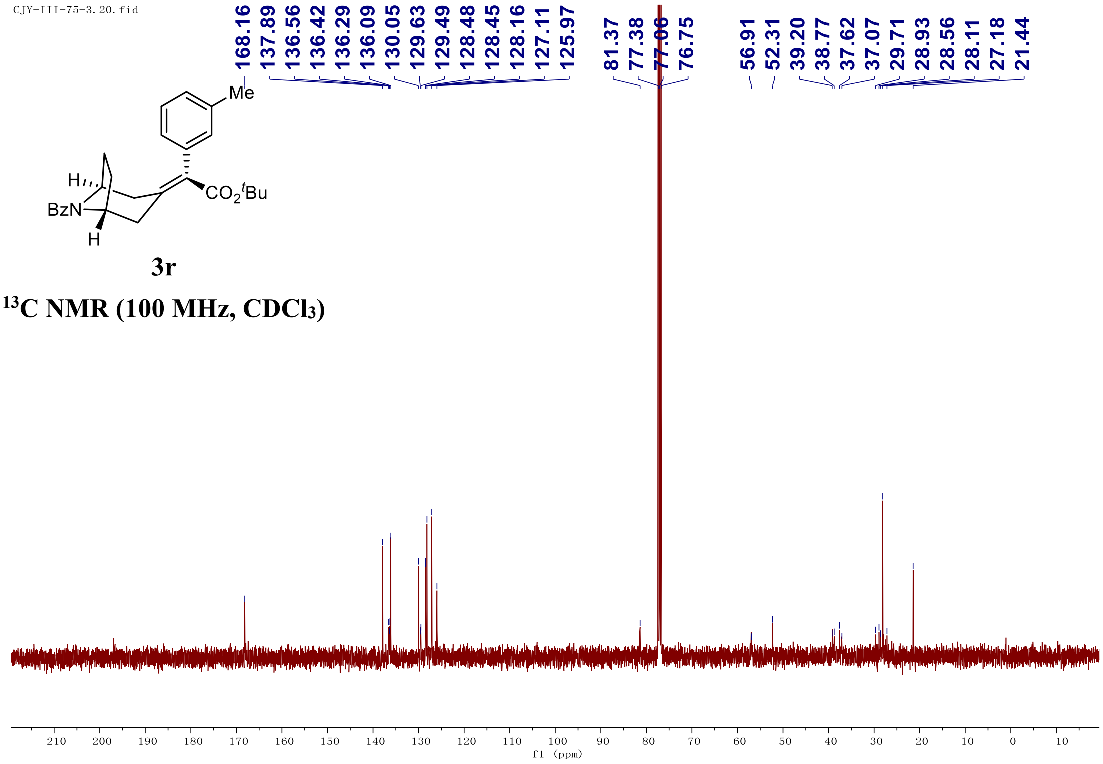

Figure S65 ^{13}C NMR (100 MHz, $CDCl_3$) spectrum for **3r**

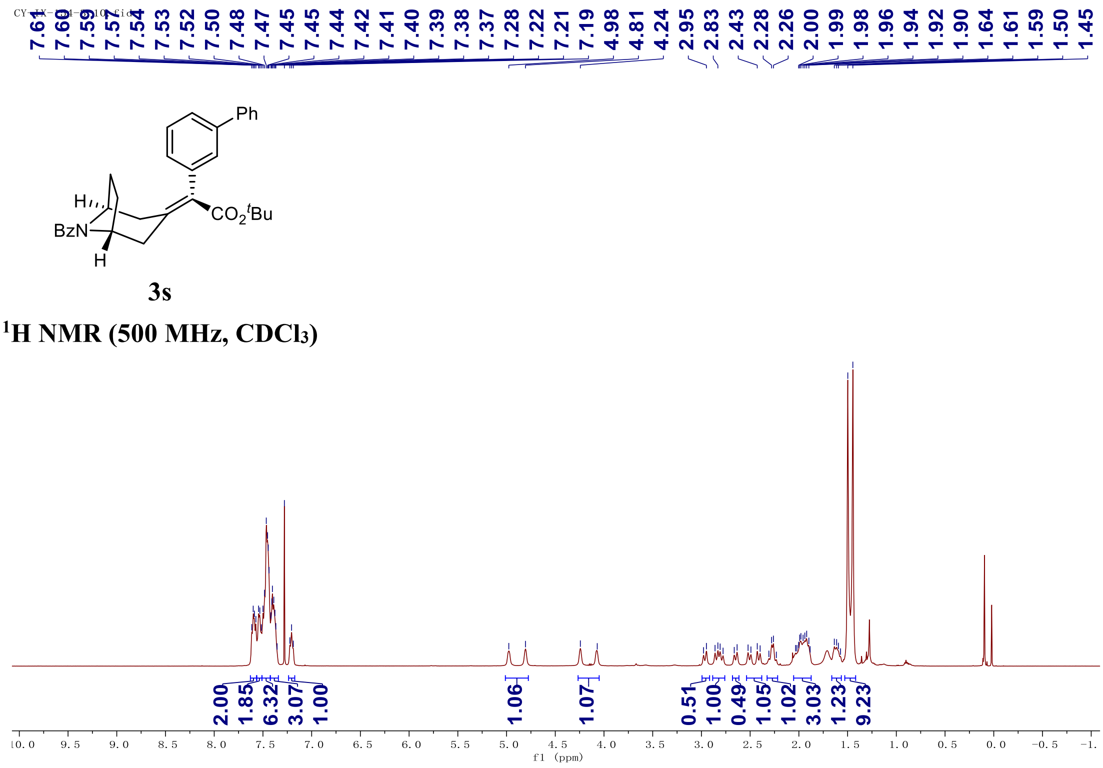

Figure S66 ^{1}H NMR (500 MHz, $CDCl_3$) spectrum for **3s**

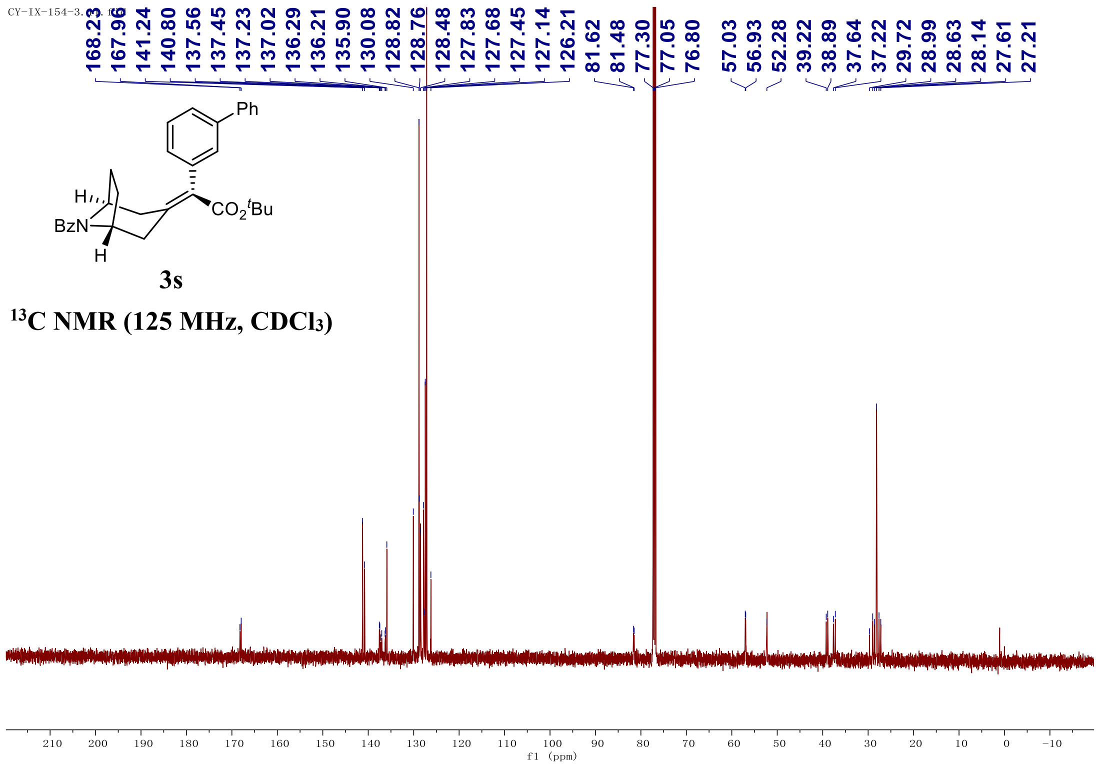

Figure S67 ^{13}C NMR (125 MHz, $CDCl_3$) spectrum for **3s**

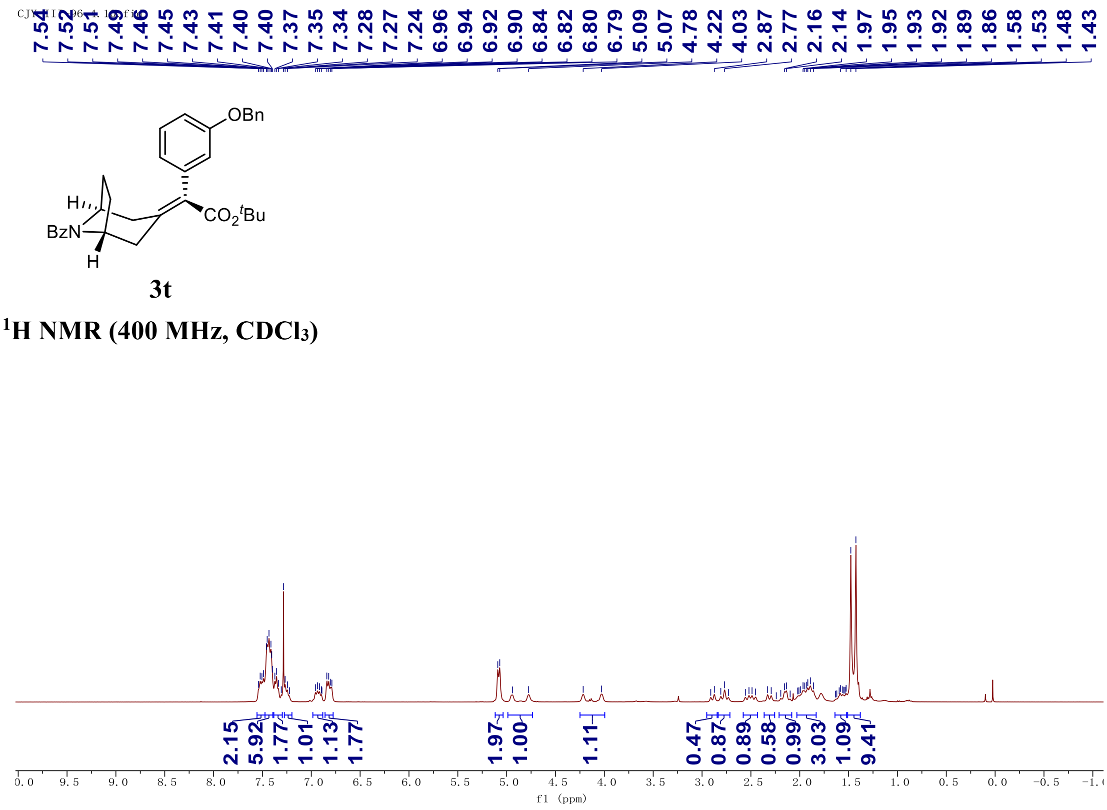

Figure S68 ^{1}H NMR (400 MHz, $CDCl_3$) spectrum for **3t**

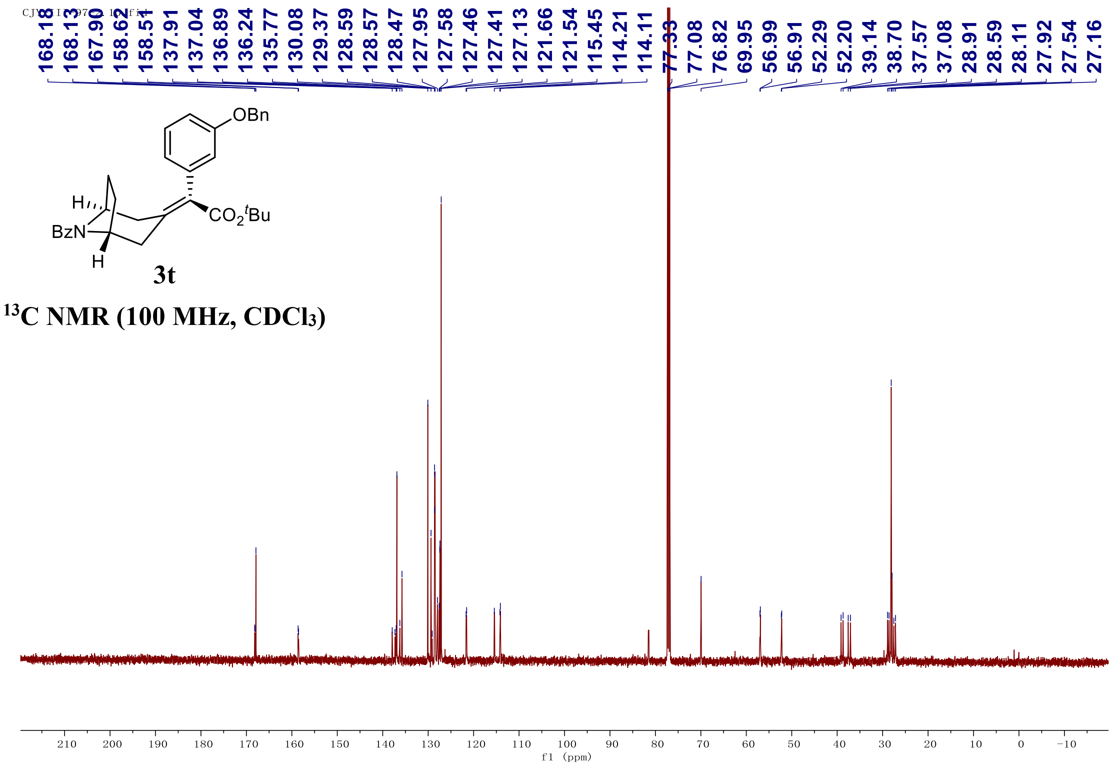

Figure S69 ^{13}C NMR (100 MHz, $CDCl_3$) spectrum for **3t**

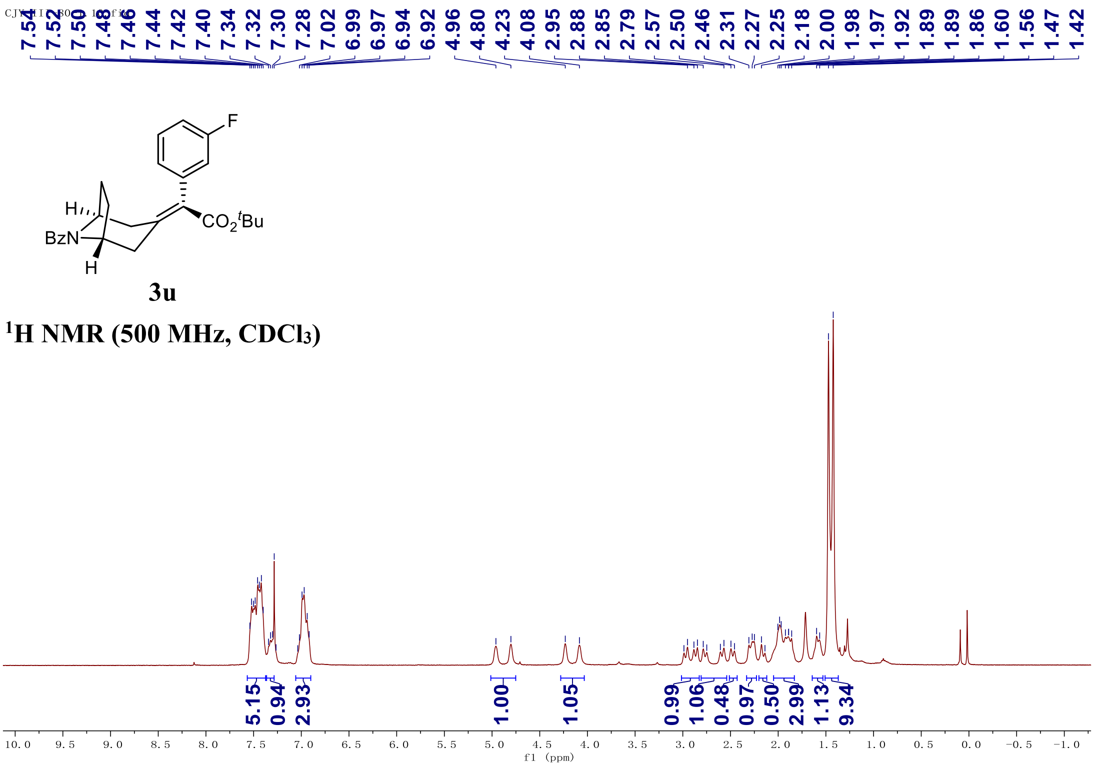

Figure S70 ^{1}H NMR (500 MHz, $CDCl_3$) spectrum for **3u**

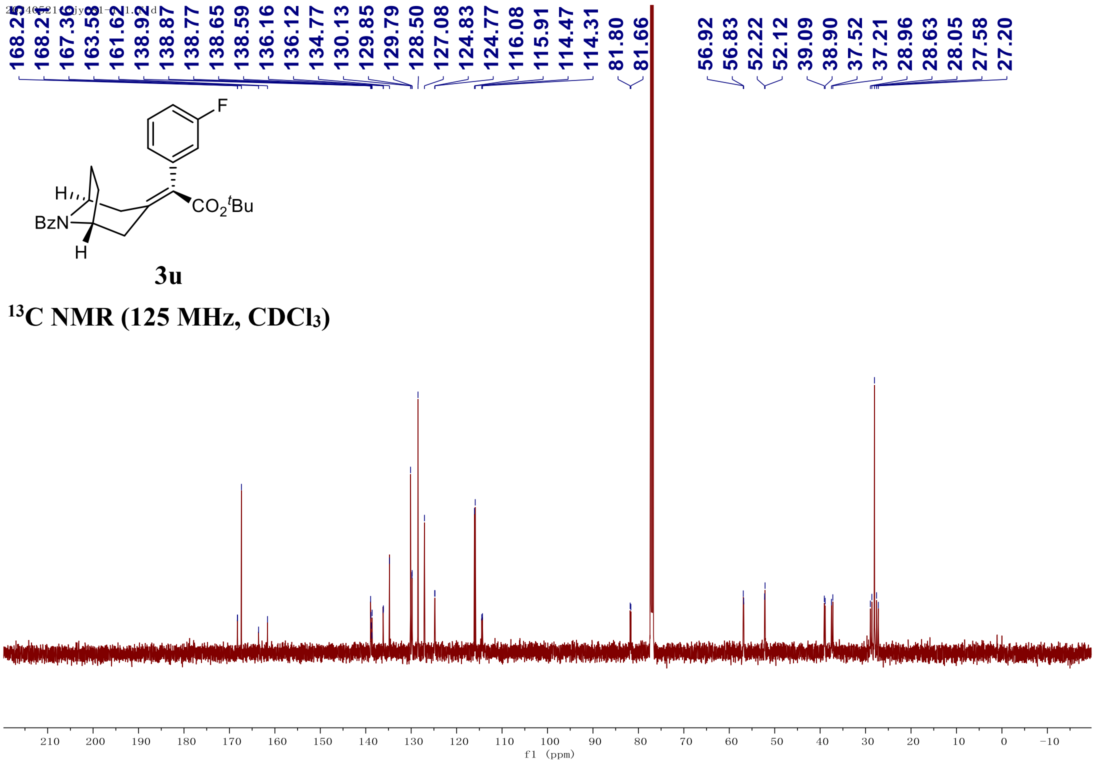

Figure S71 ^{13}C NMR (125 MHz, $CDCl_3$) spectrum for **3u**

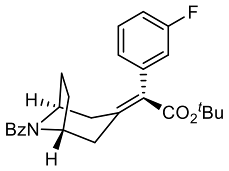

3u

^{19}F NMR (376 MHz, $CDCl_3$)

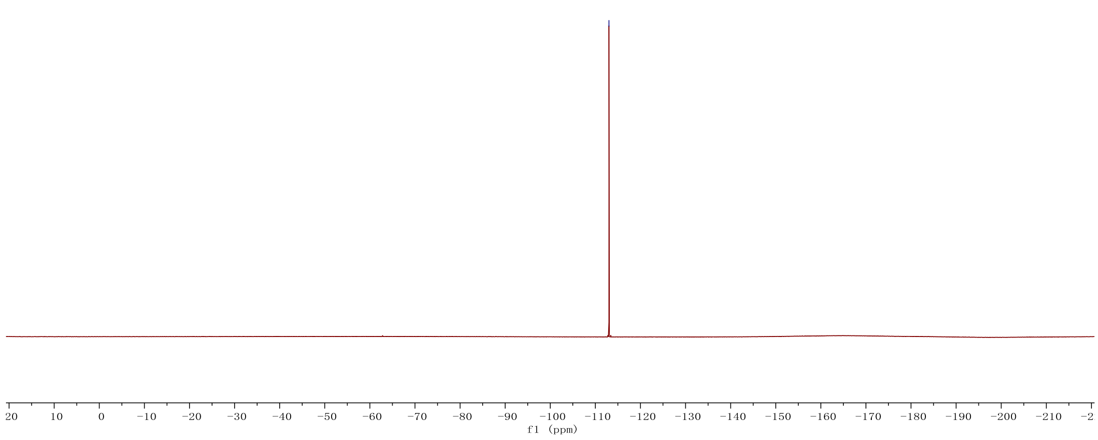

Figure S72 ^{19}F NMR (376 MHz, $CDCl_3$) spectrum for **3u**

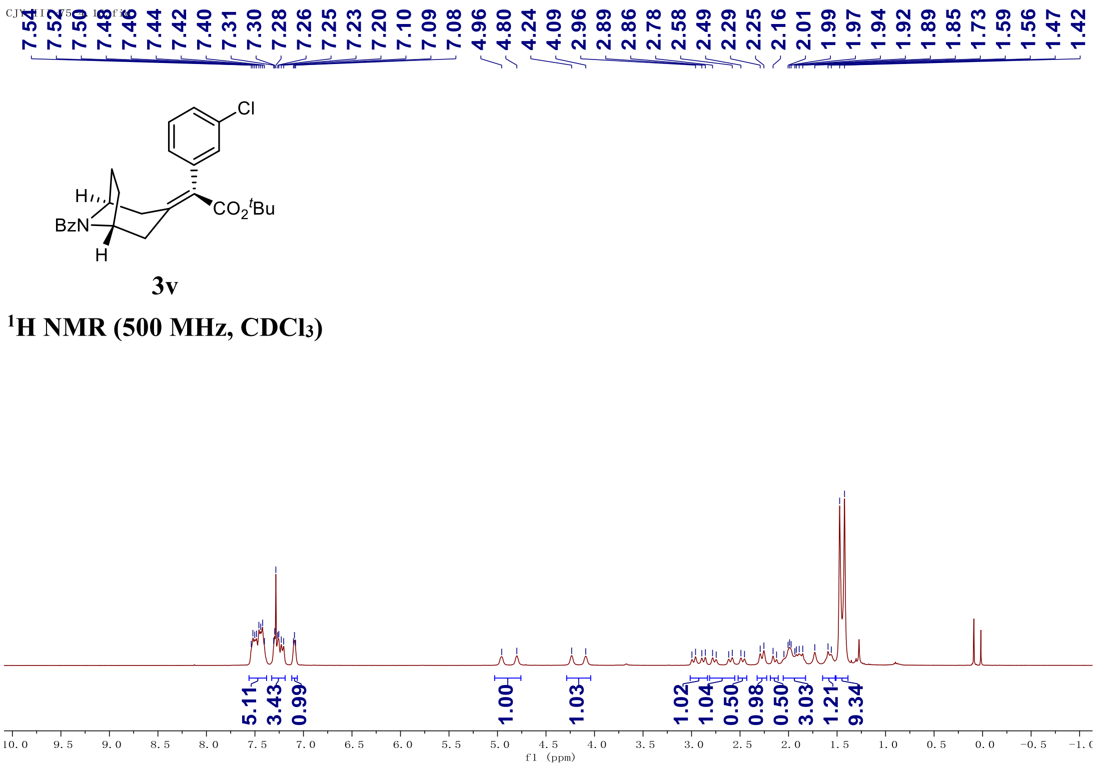

Figure S73 ^{1}H NMR (500 MHz, $CDCl_3$) spectrum for **3v**

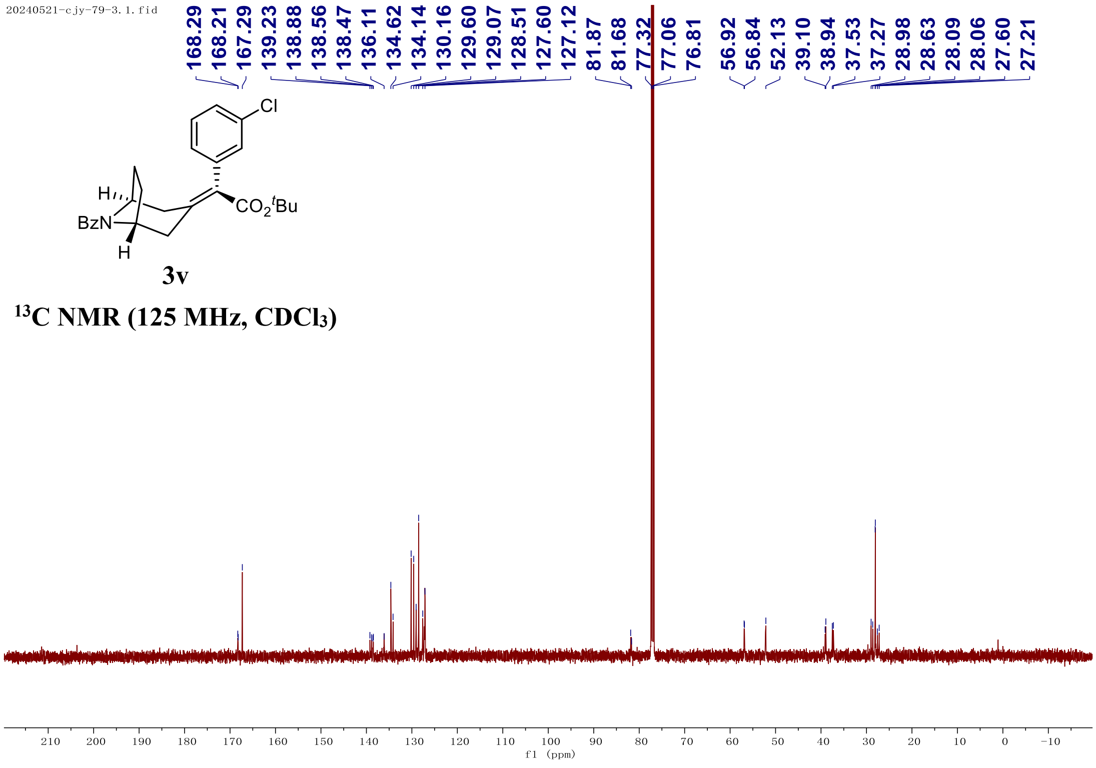

Figure S74 ^{13}C NMR (125 MHz, $CDCl_3$) spectrum for **3v**

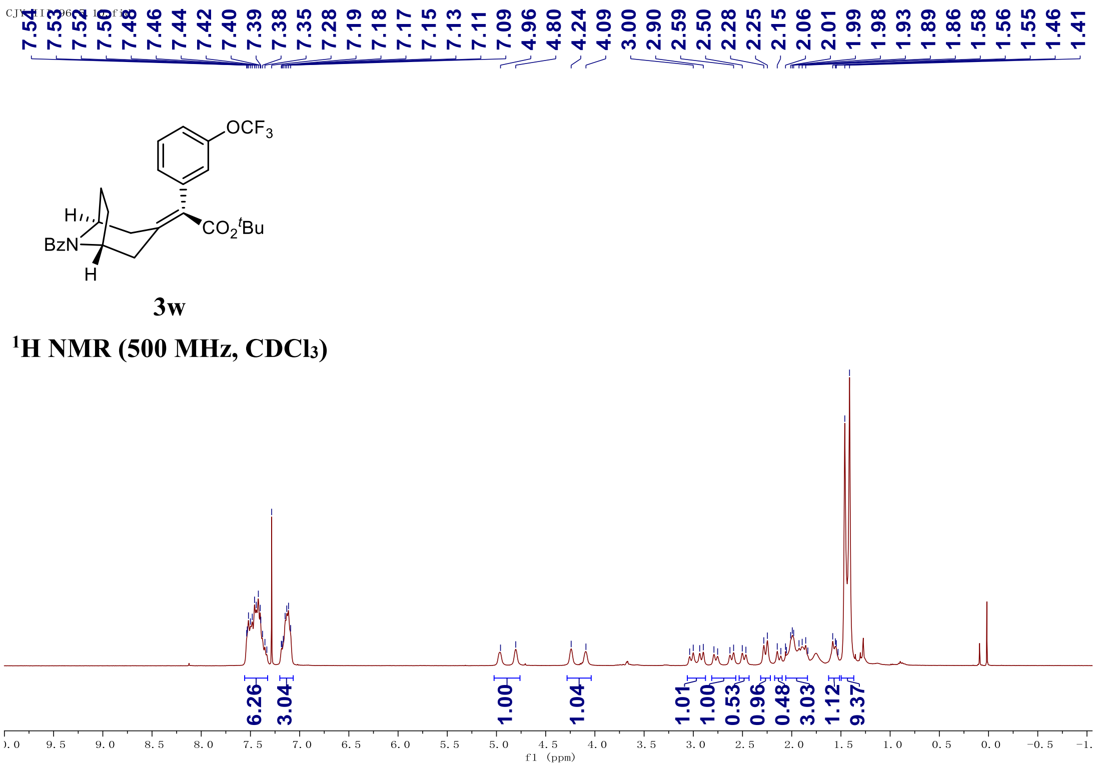

Figure S75 ^{1}H NMR (500 MHz, $CDCl_3$) spectrum for **3w**

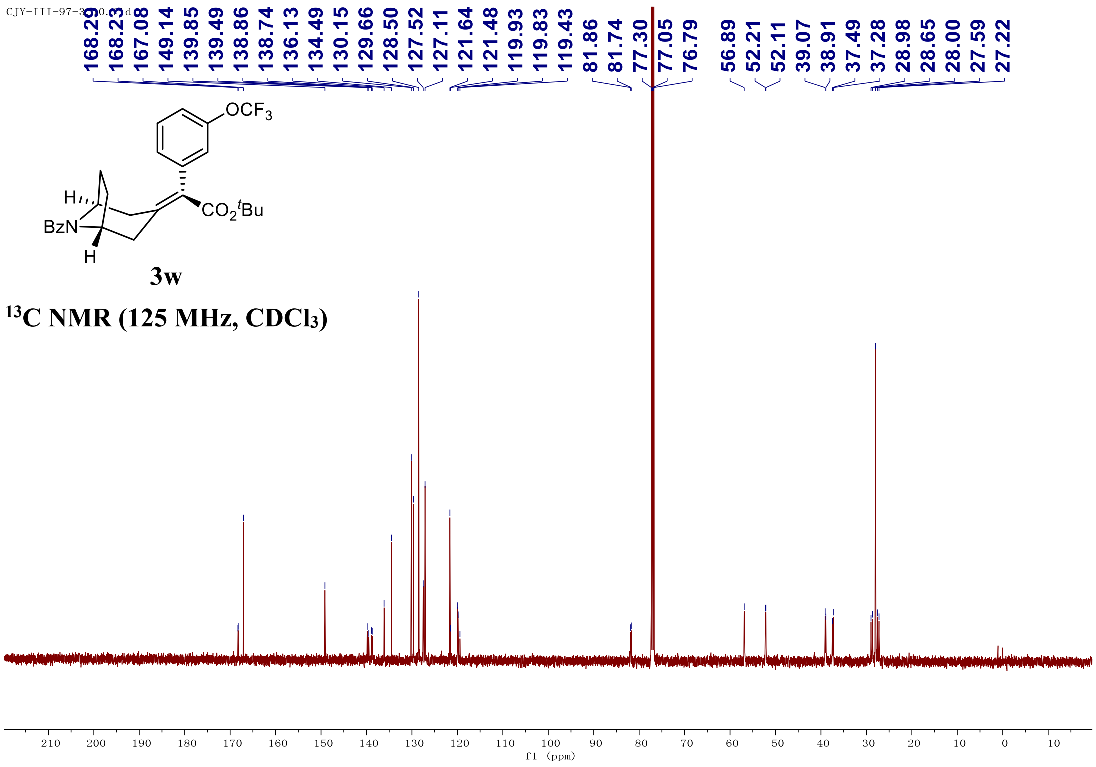

Figure S76 ^{13}C NMR (125 MHz, $CDCl_3$) spectrum for **3w**

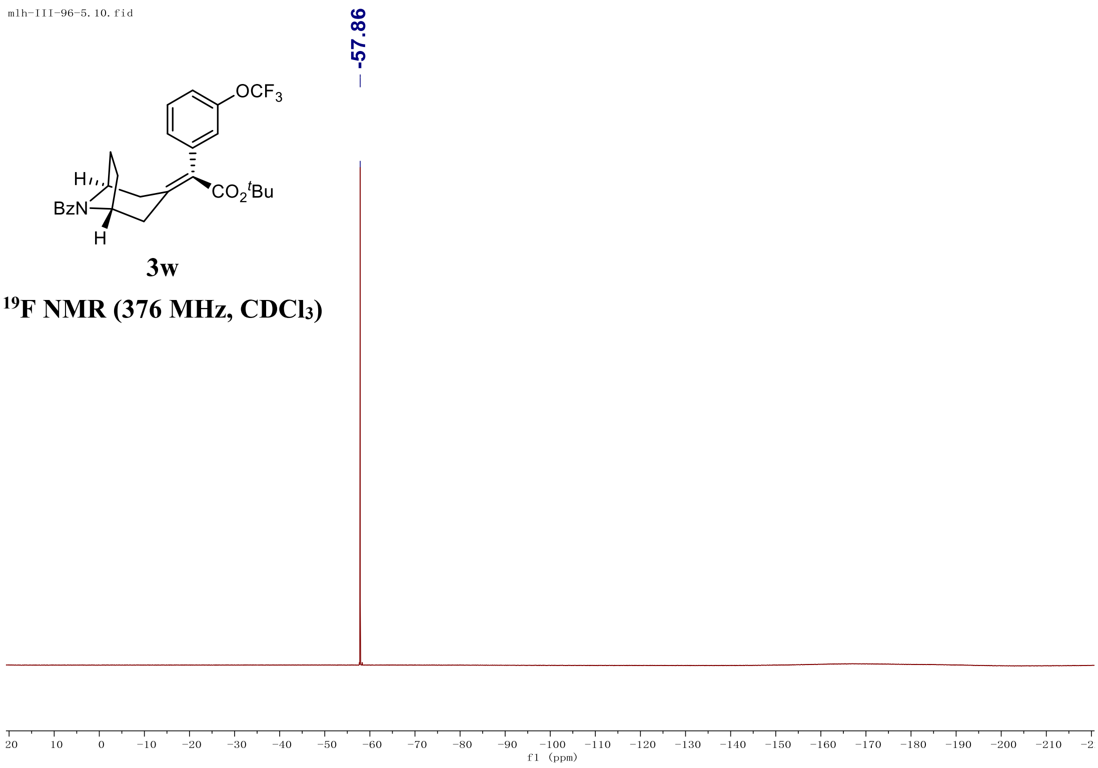

Figure S77 ^{19}F NMR (376 MHz, $CDCl_3$) spectrum for **3w**

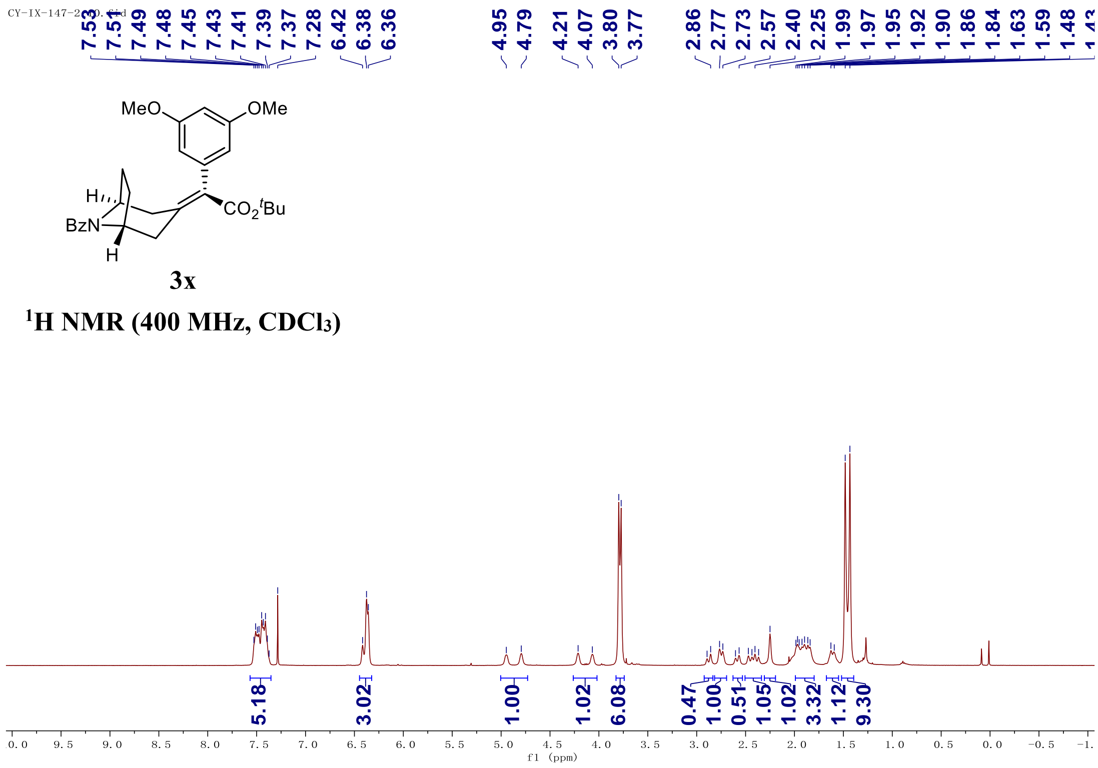

Figure S78 ^{1}H NMR (400 MHz, $CDCl_3$) spectrum for **3x**

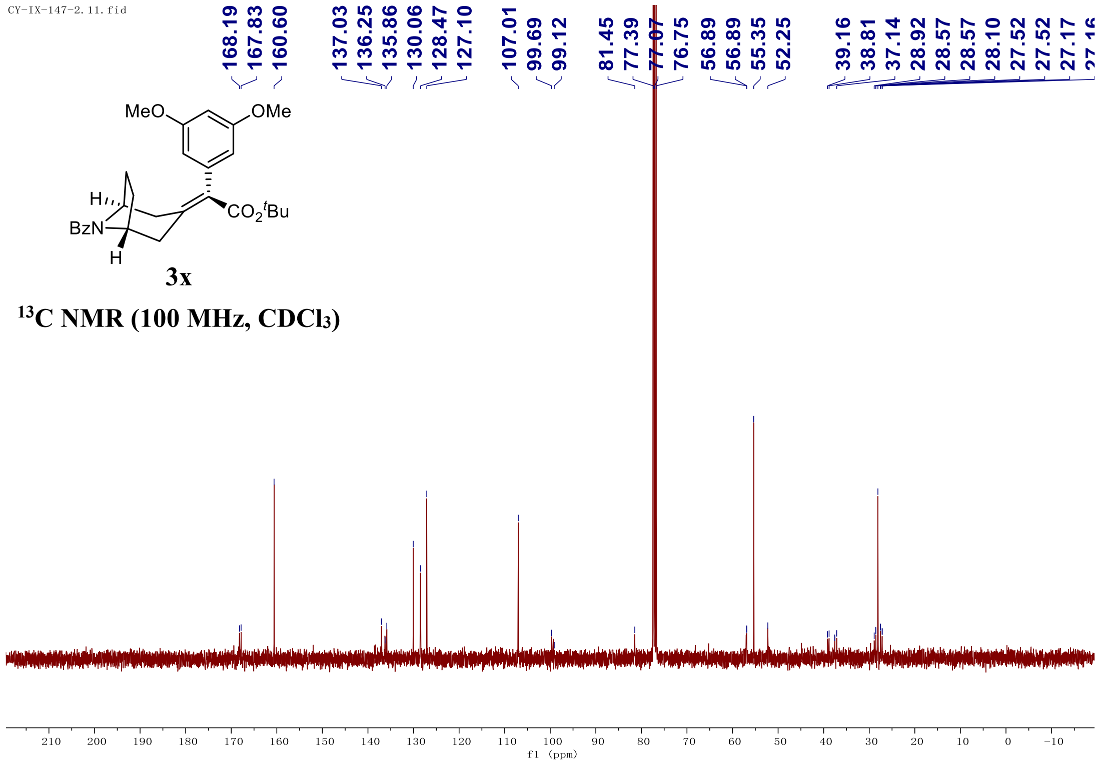

Figure S79 ^{13}C NMR (100 MHz, $CDCl_3$) spectrum for **3x**

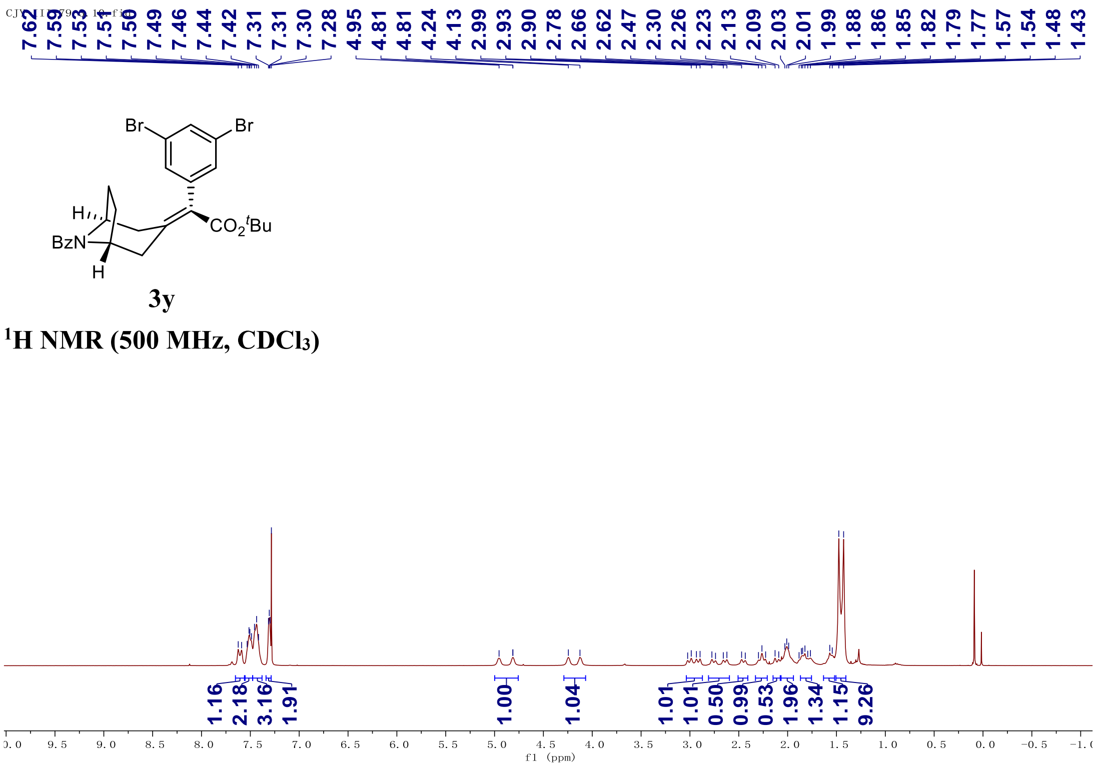

Figure S80 ^{1}H NMR (500 MHz, $CDCl_3$) spectrum for **3y**

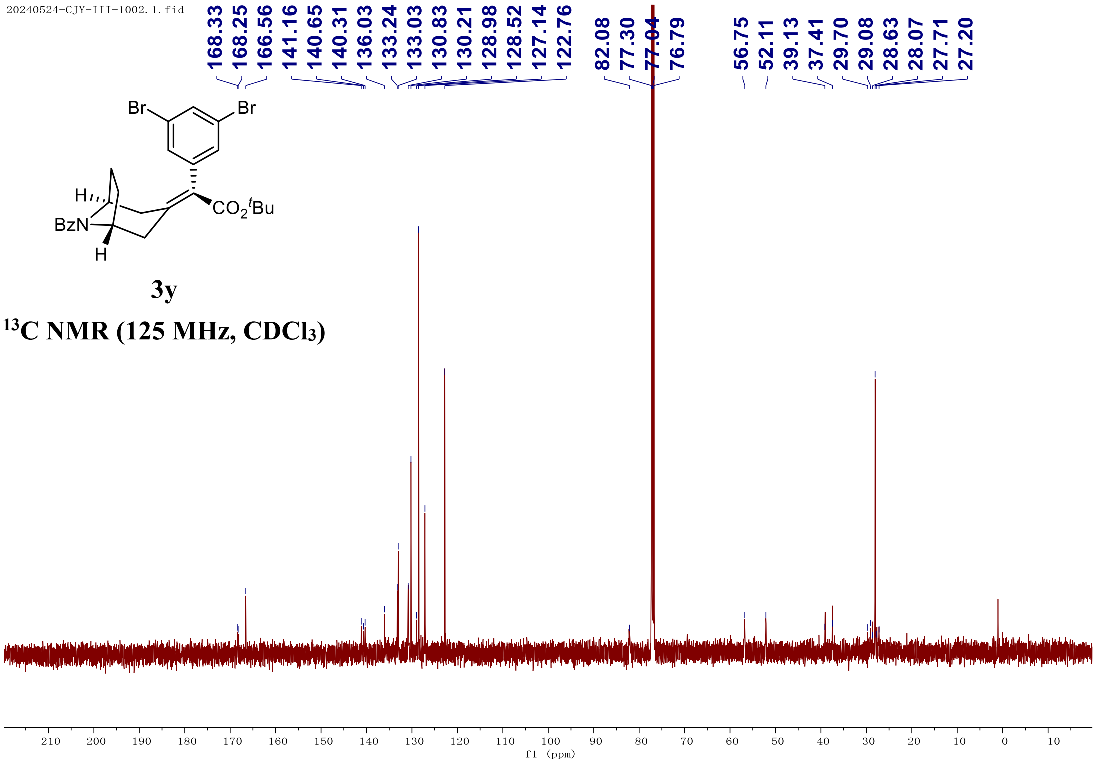

Figure S81 ^{13}C NMR (125 MHz, $CDCl_3$) spectrum for **3y**

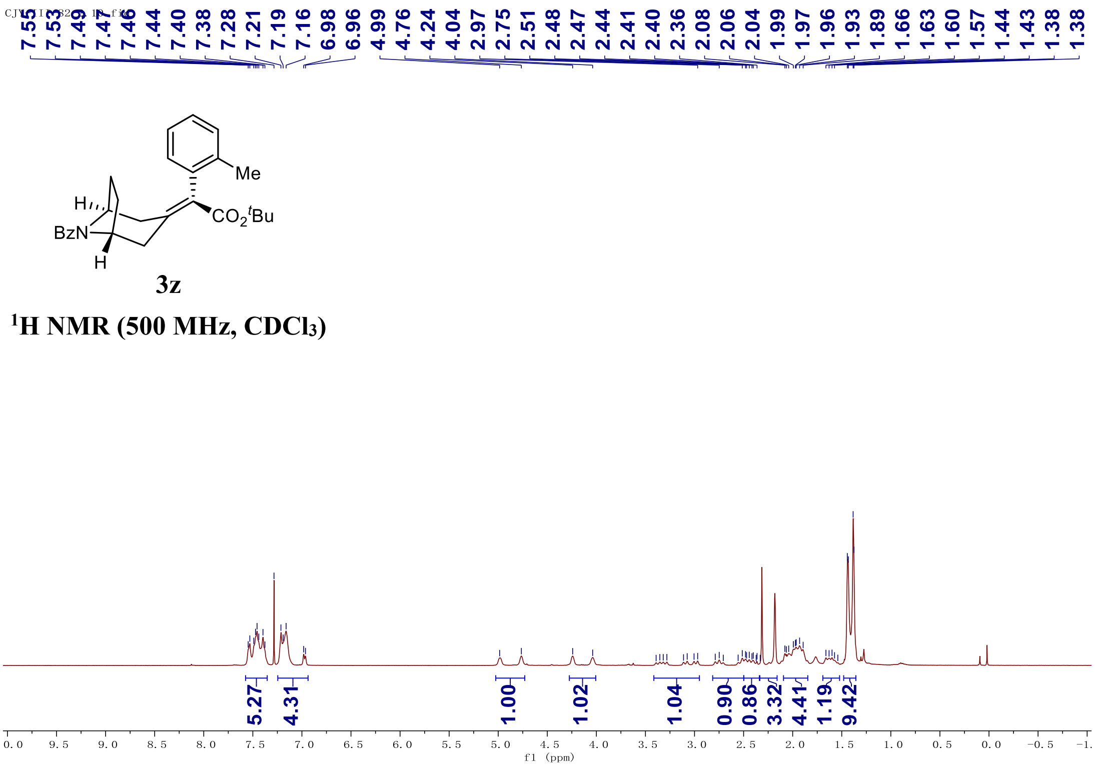

Figure S82 ^{1}H NMR (500 MHz, $CDCl_3$) spectrum for **3z**

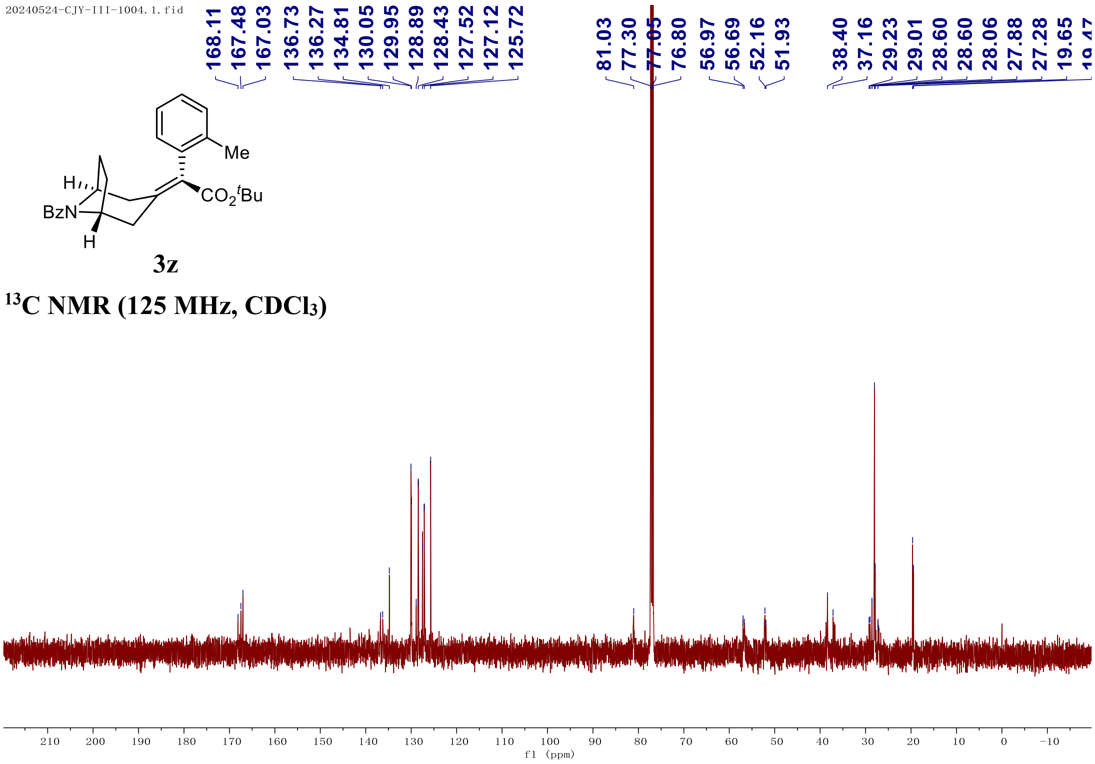

Figure S83 ^{13}C NMR (125 MHz, $CDCl_3$) spectrum for **3z**

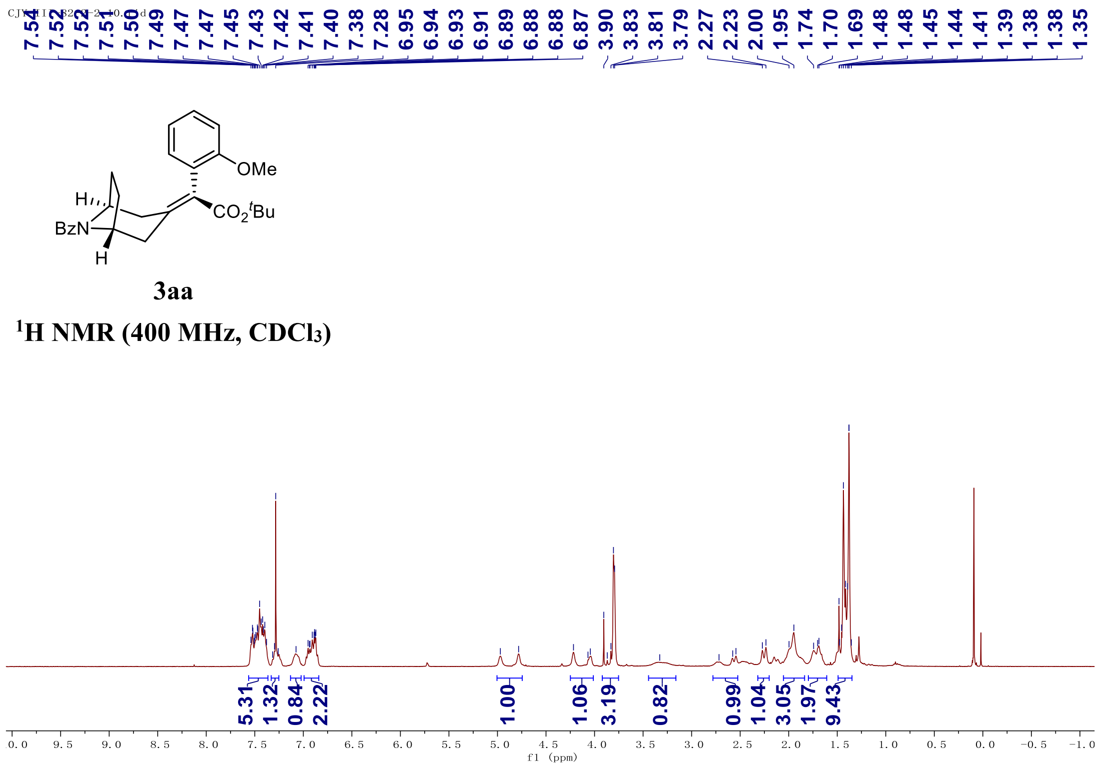

Figure S84 ^1H NMR (400 MHz, $CDCl_3$) spectrum for **3aa**

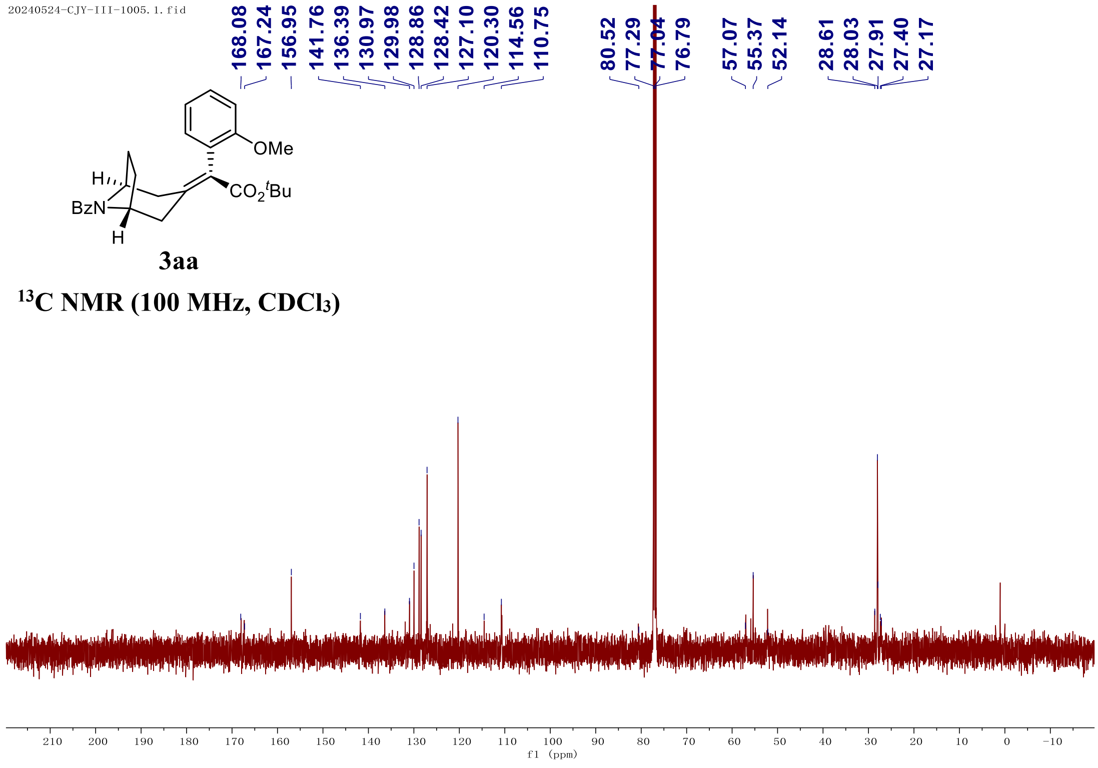

Figure S85 ^{13}C NMR (100 MHz, $CDCl_3$) spectrum for **3aa**

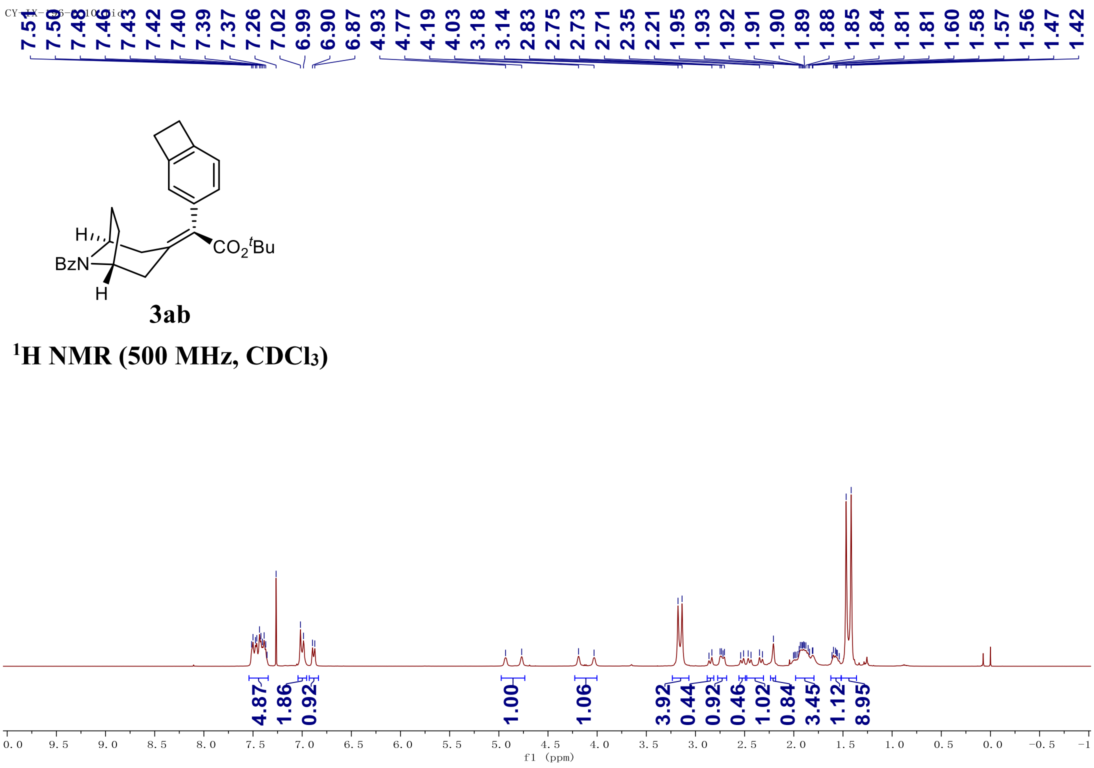

Figure S86 ^{1}H NMR (500 MHz, CDCl$_3$) spectrum for **3ab**

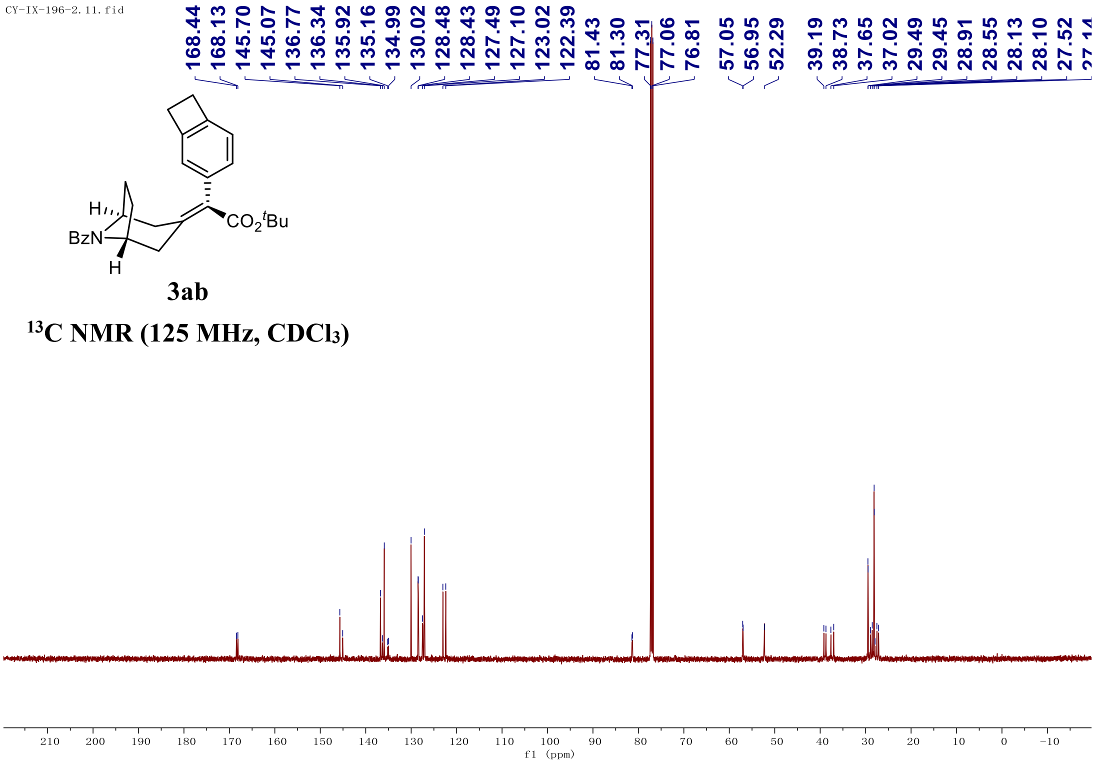

Figure S87 ^{13}C NMR (125 MHz, CDCl$_3$) spectrum for **3ab**

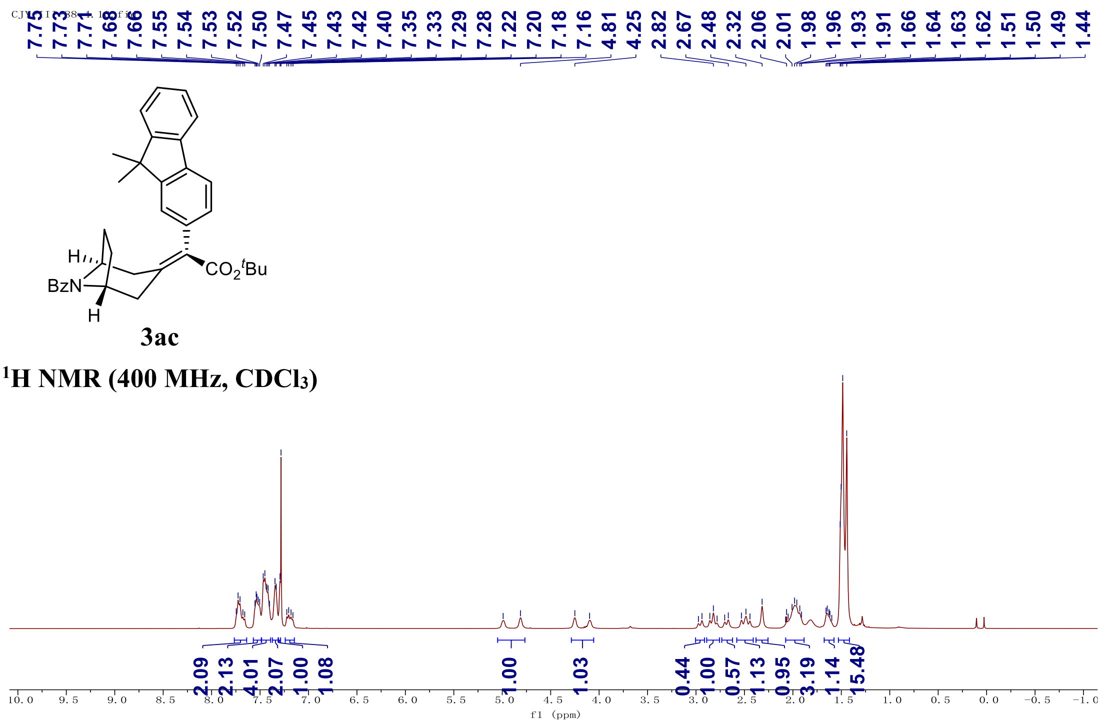

Figure S88 ^{1}H NMR (400 MHz, $CDCl_3$) spectrum for **3ac**

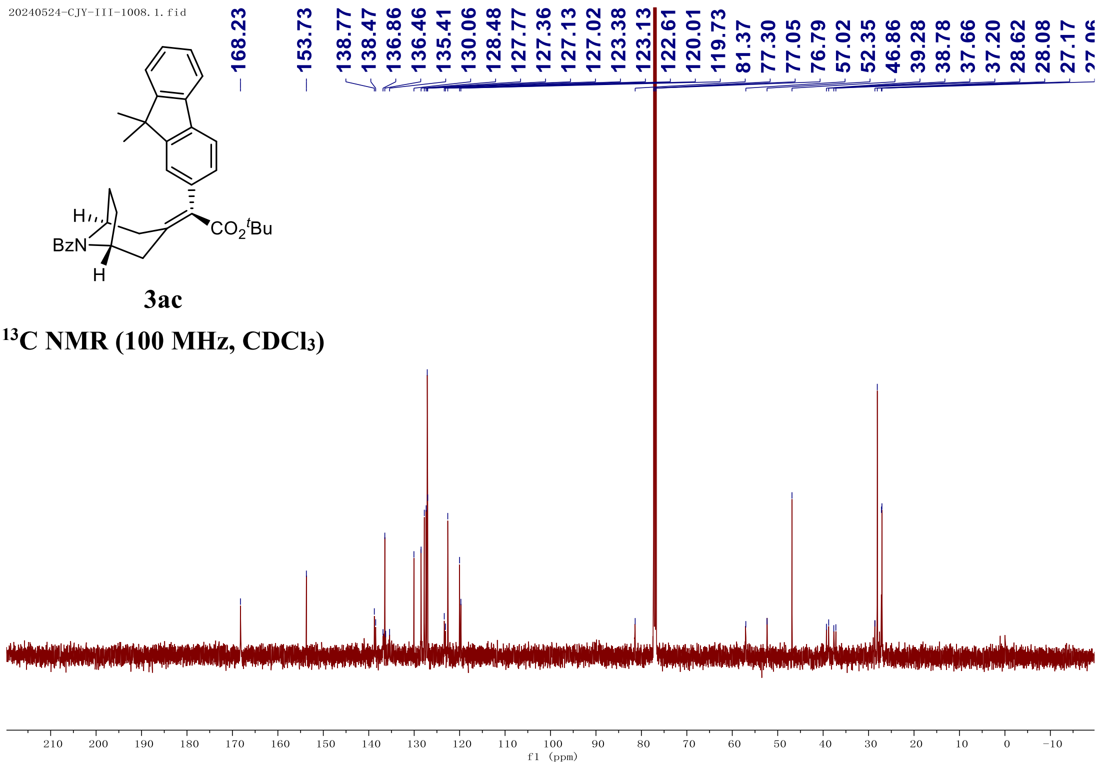

Figure S89 ^{13}C NMR (100 MHz, $CDCl_3$) spectrum for **3ac**

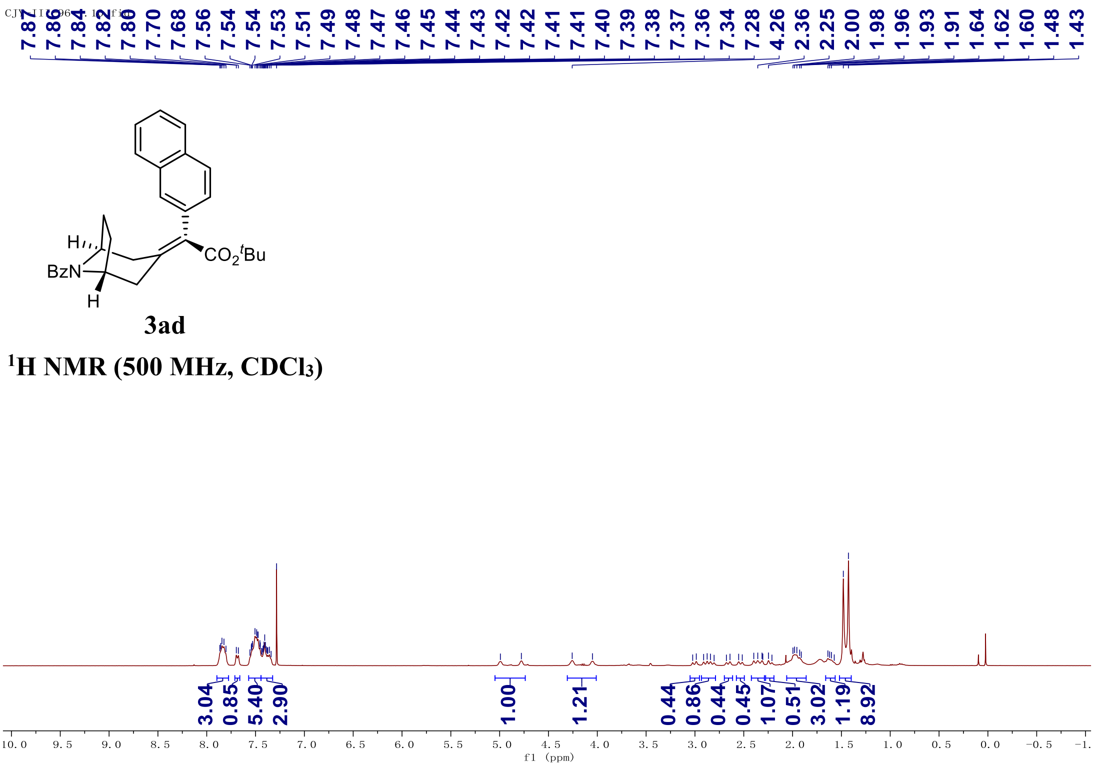

Figure S90 ^{1}H NMR (500 MHz, $CDCl_3$) spectrum for **3ad**

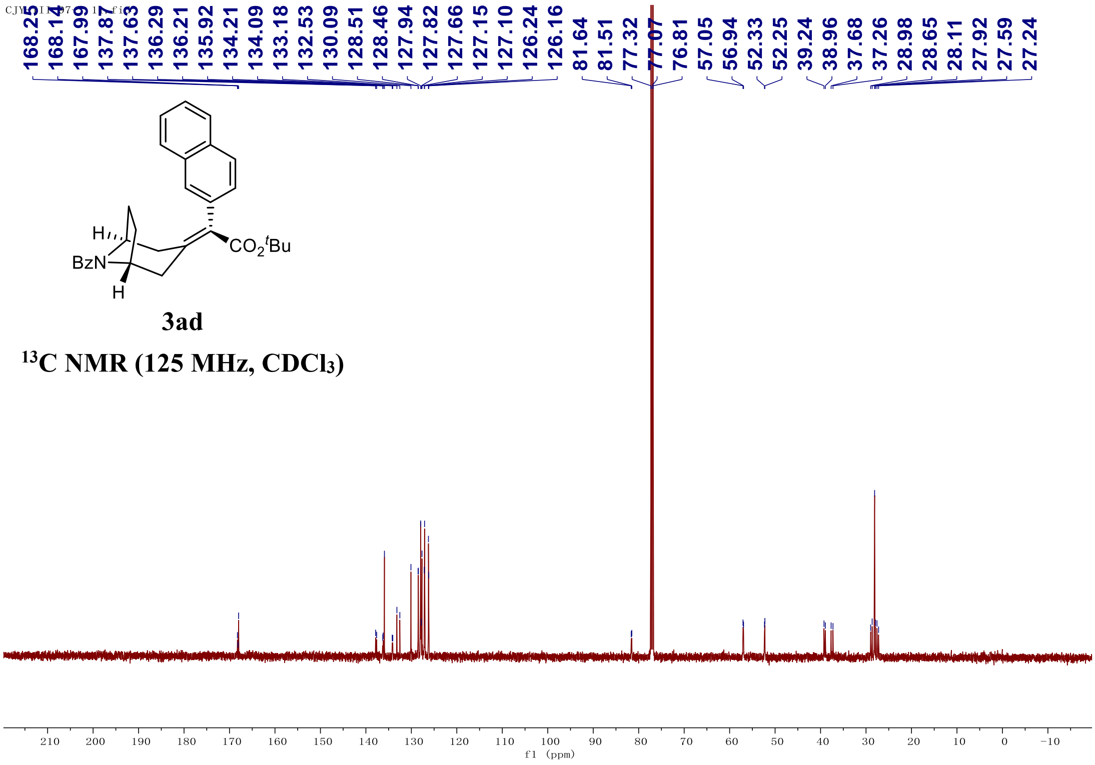

Figure S91 ^{13}C NMR (125 MHz, $CDCl_3$) spectrum for **3ad**

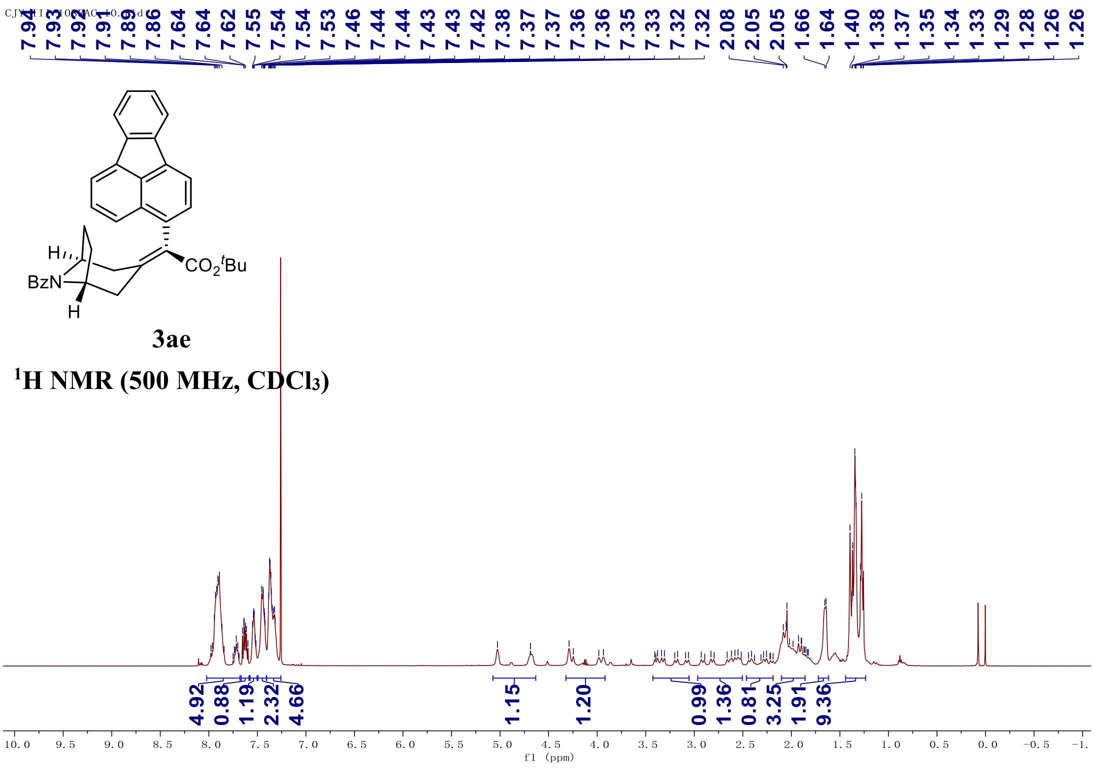

Figure S92 ^{1}H NMR (500 MHz, $CDCl_3$) spectrum for **3ae**

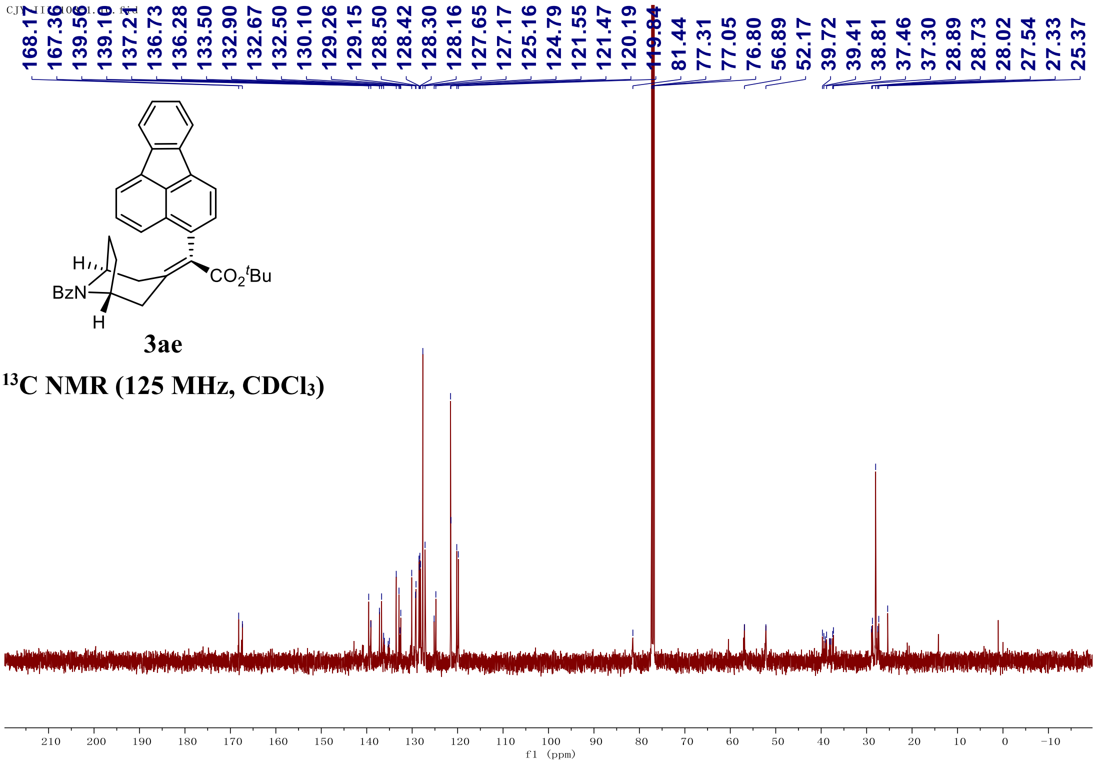

Figure S93 ^{13}C NMR (125 MHz, $CDCl_3$) spectrum for **3ae**

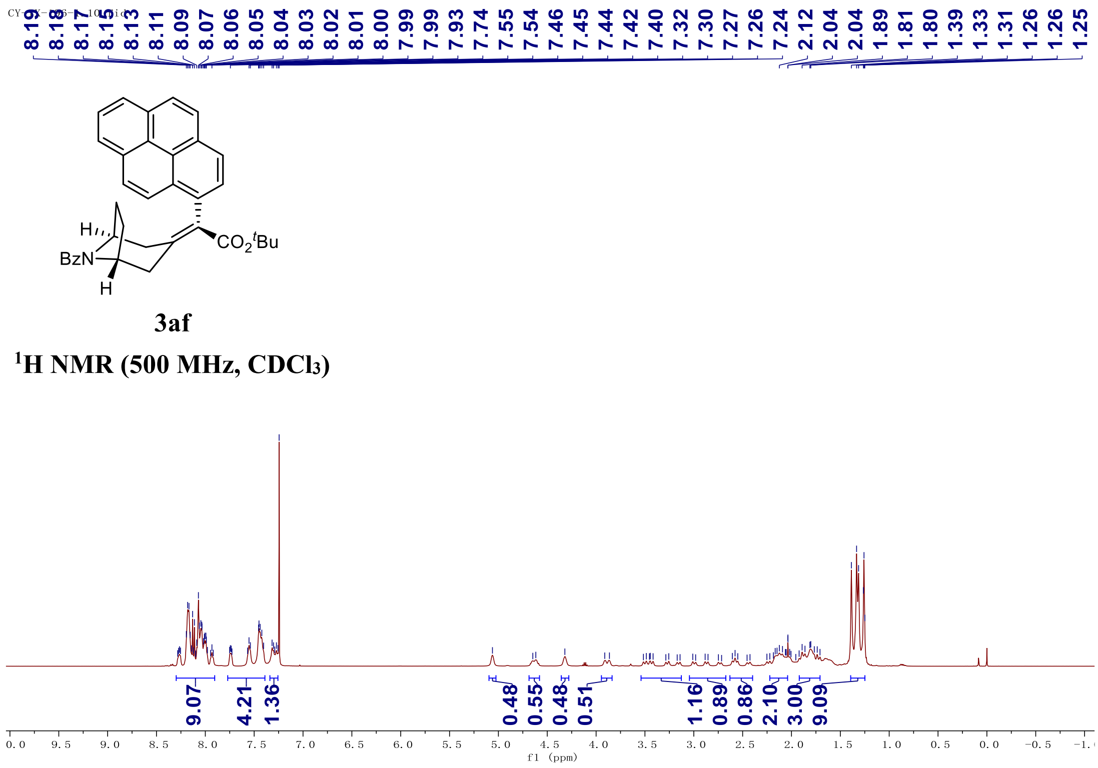

Figure S94 ^1H NMR (500 MHz, $CDCl_3$) spectrum for **3af**

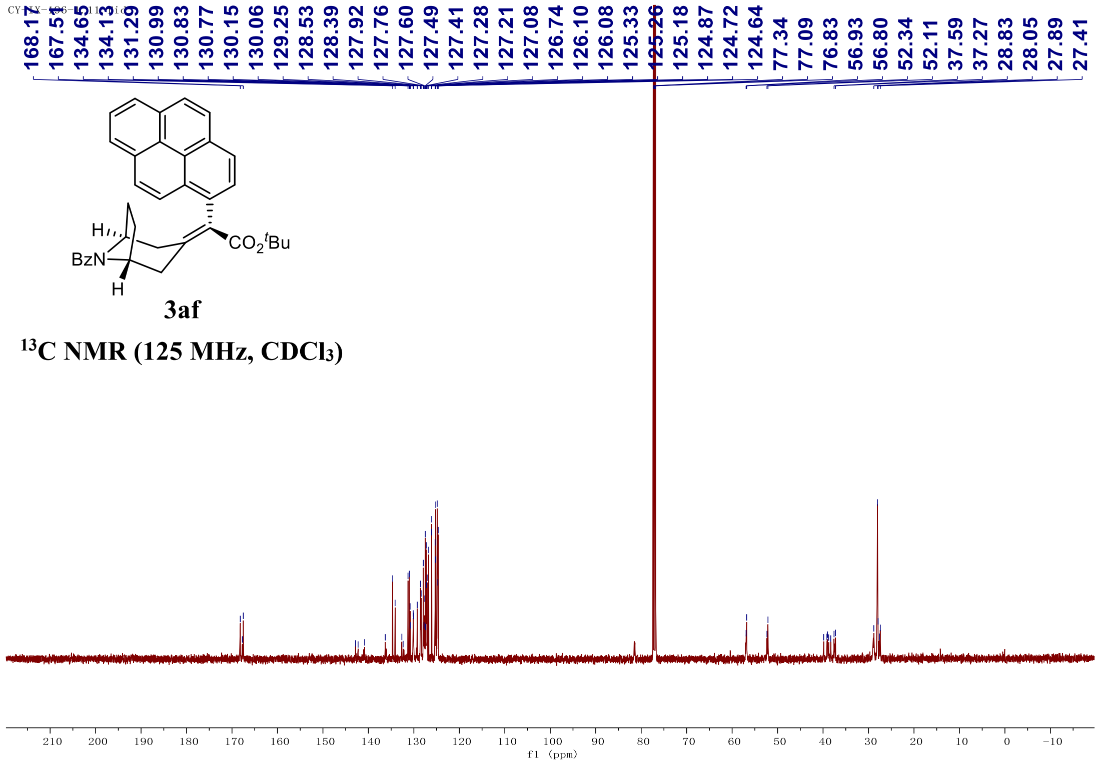

Figure S95 ^{13}C NMR (125 MHz, $CDCl_3$) spectrum for **3af**

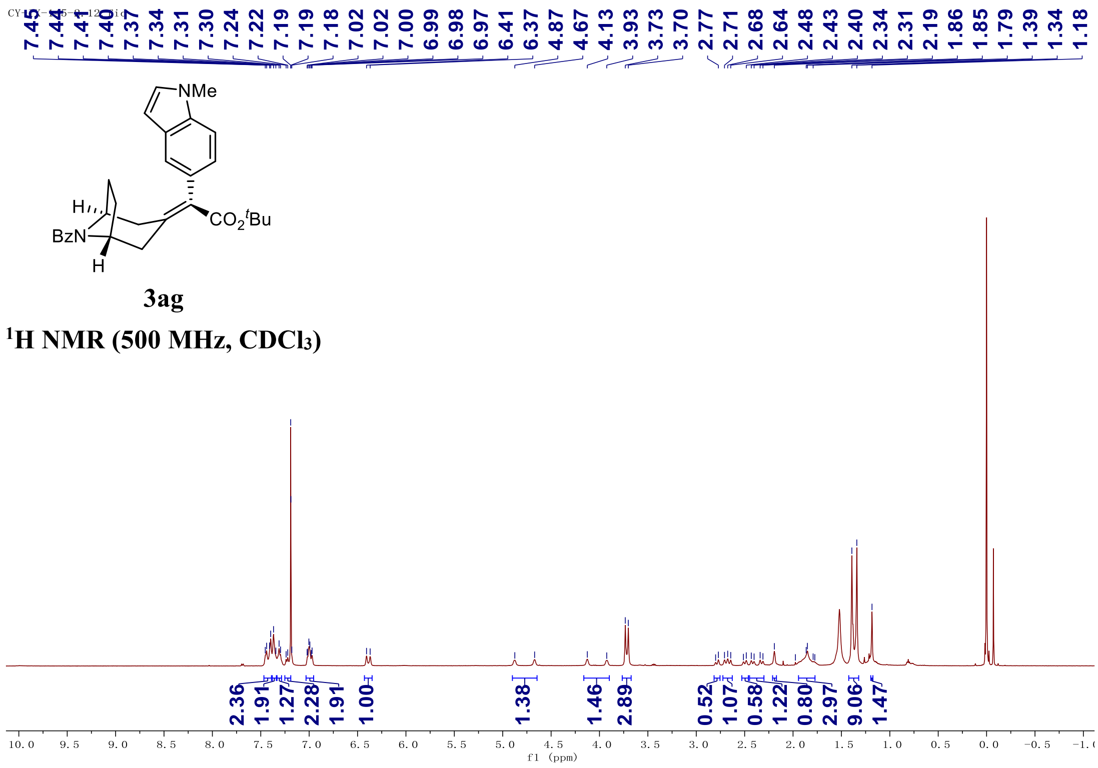

Figure S96 ^{1}H NMR (500 MHz, $CDCl_3$) spectrum for **3ag**

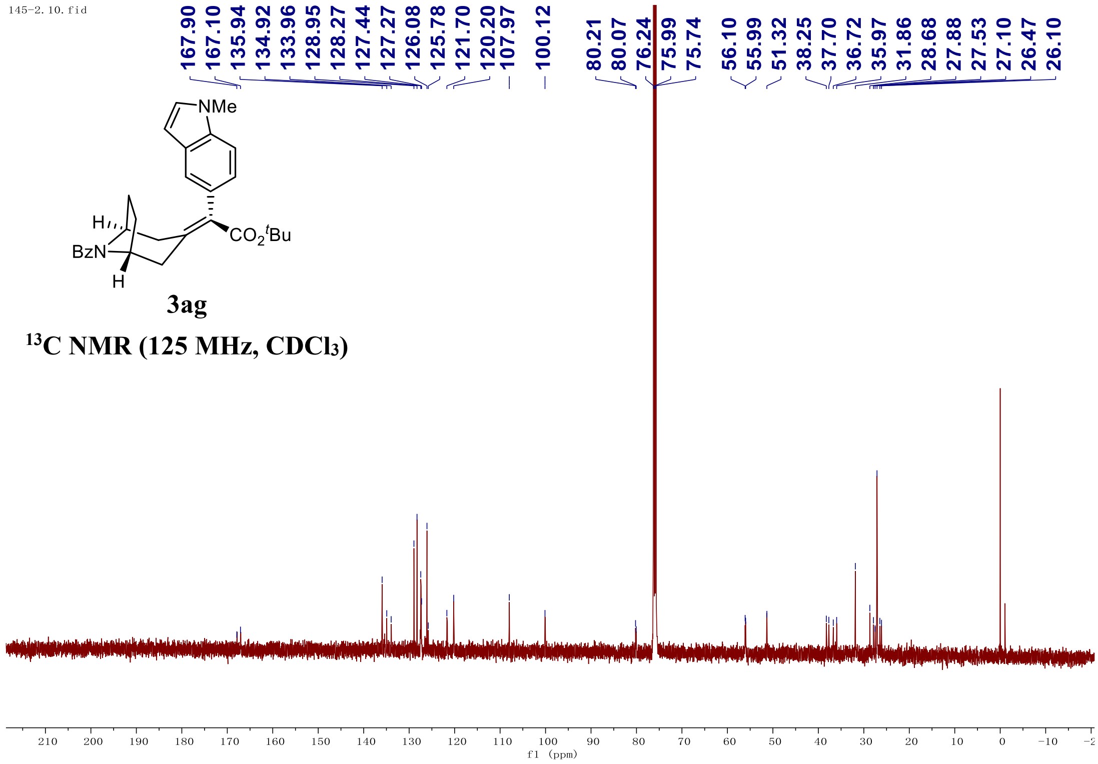

Figure S97 ^{13}C NMR (125 MHz, $CDCl_3$) spectrum for **3ag**

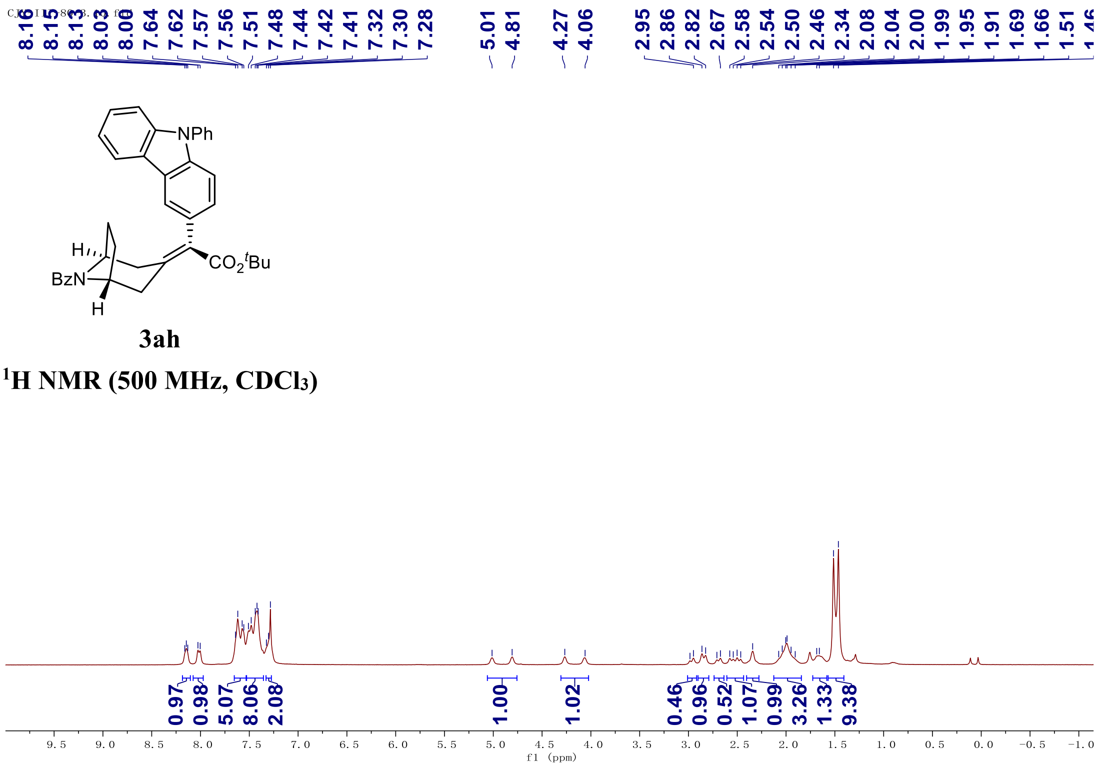

Figure S98 ^{1}H NMR (500 MHz, $CDCl_3$) spectrum for **3ah**

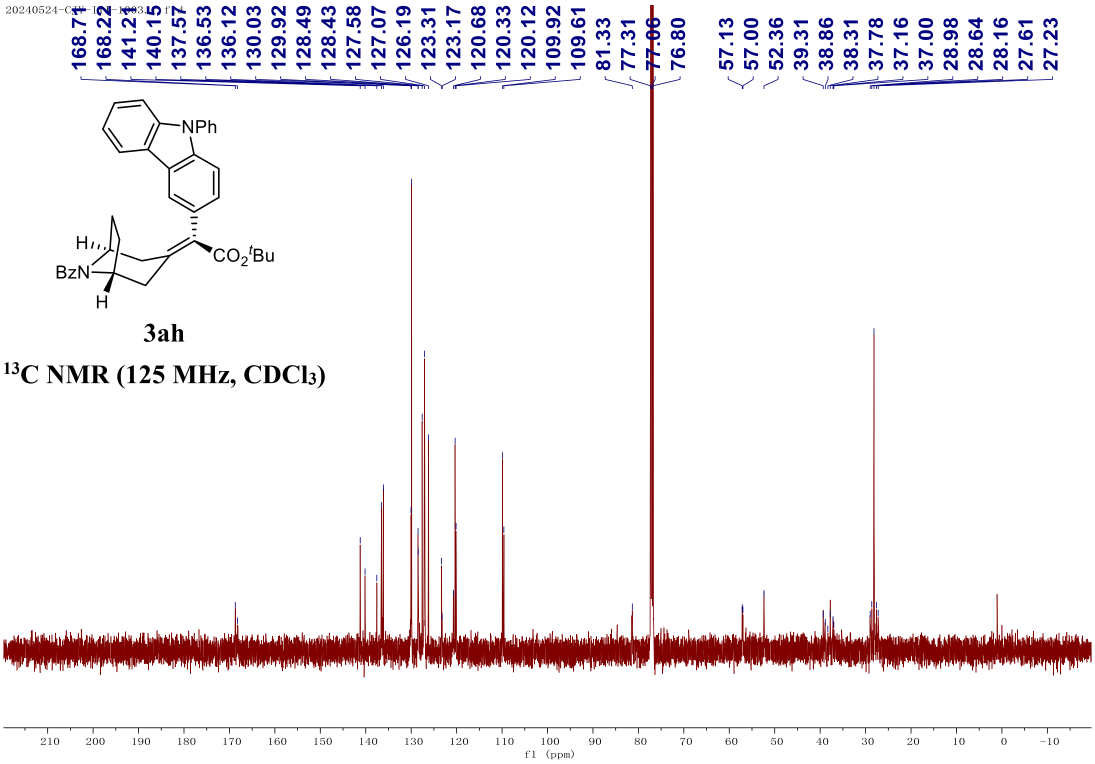

Figure S99 ^{13}C NMR (125 MHz, $CDCl_3$) spectrum for **3ah**

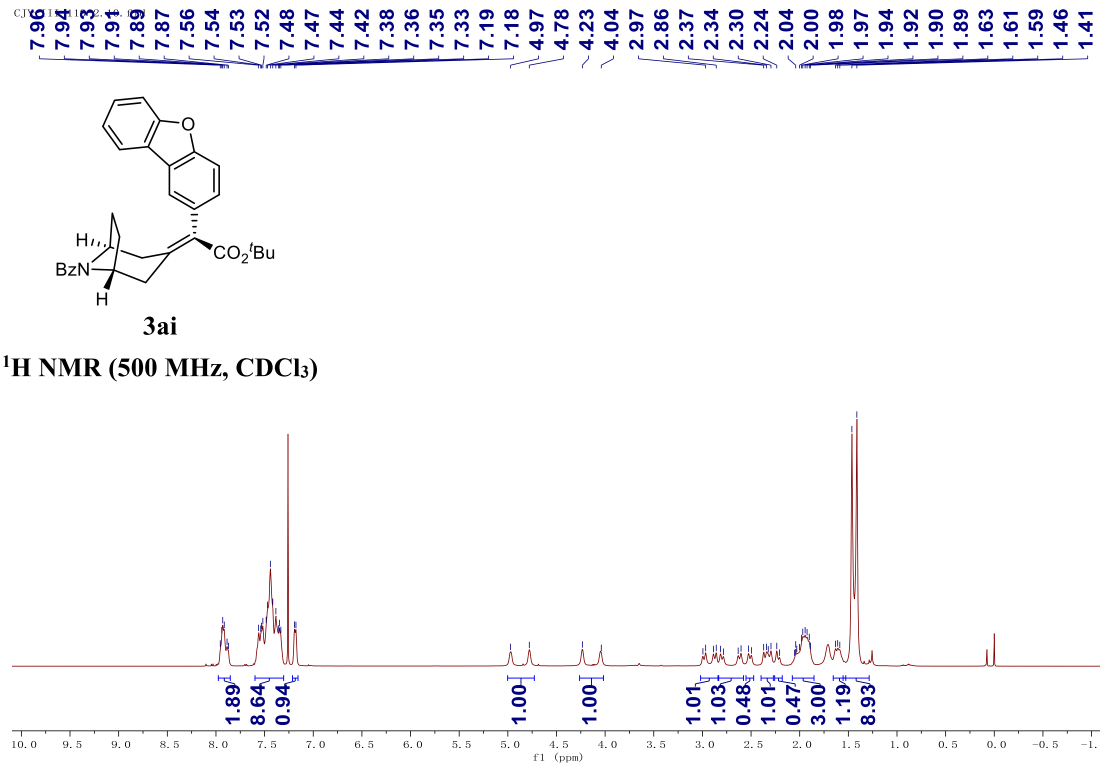

Figure S100 ^{1}H NMR (500 MHz, $CDCl_3$) spectrum for **3ai**

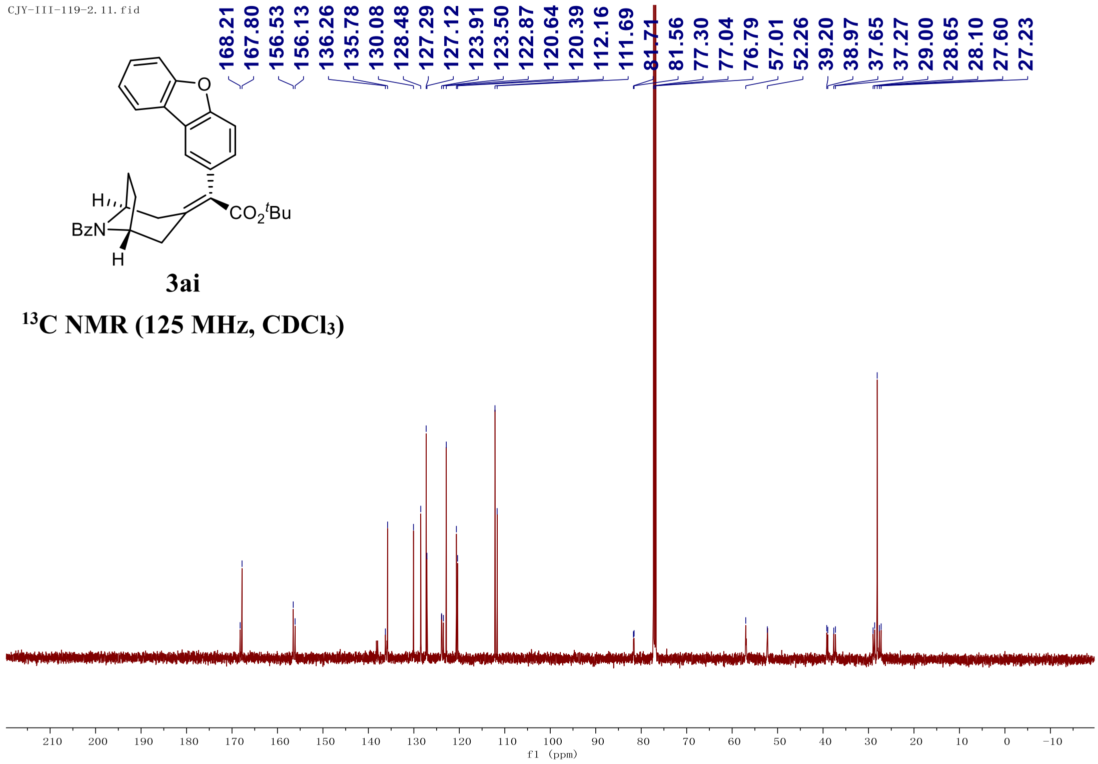

Figure S101 ^{13}C NMR (125 MHz, $CDCl_3$) spectrum for **3ai**

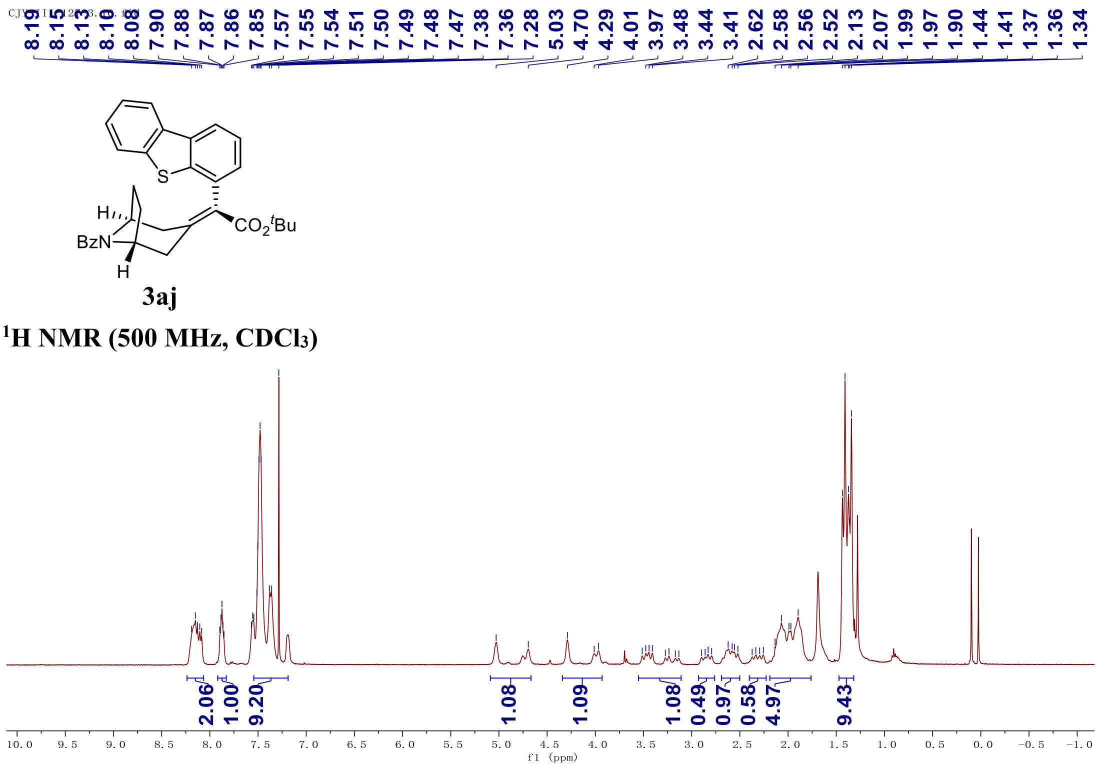

Figure S102 ^{1}H NMR (500 MHz, $CDCl_3$) spectrum for **3aj**

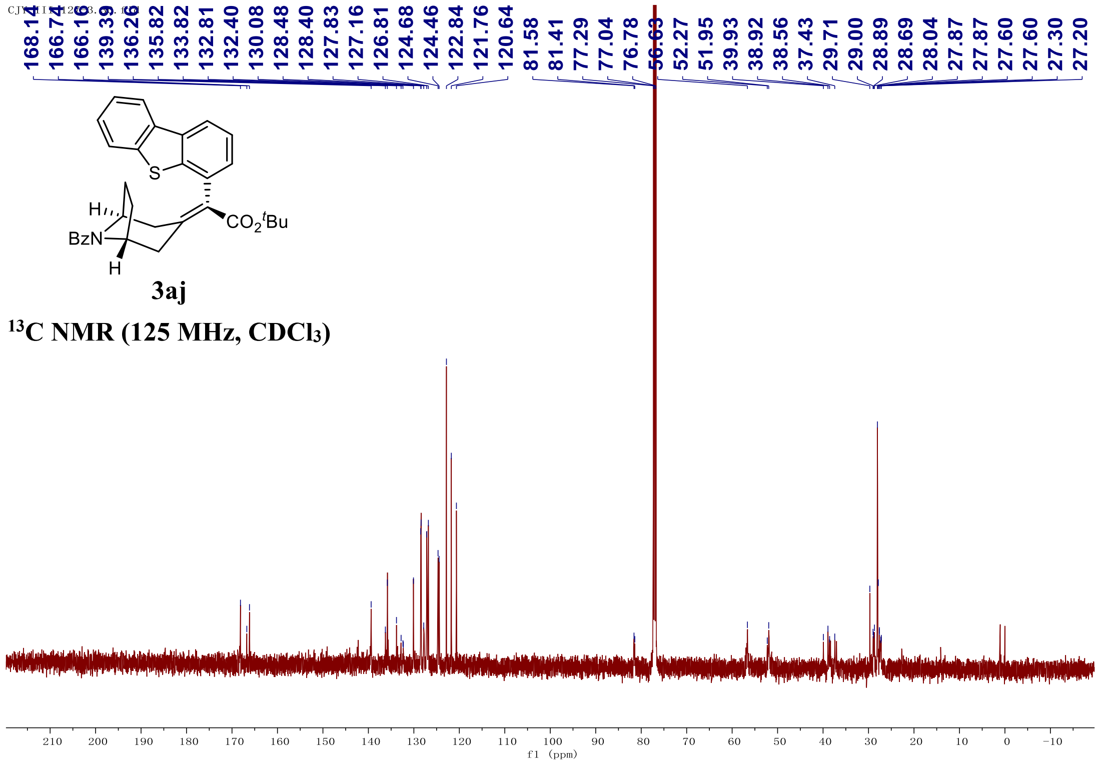

Figure S103 ^{13}C NMR (125 MHz, $CDCl_3$) spectrum for **3aj**

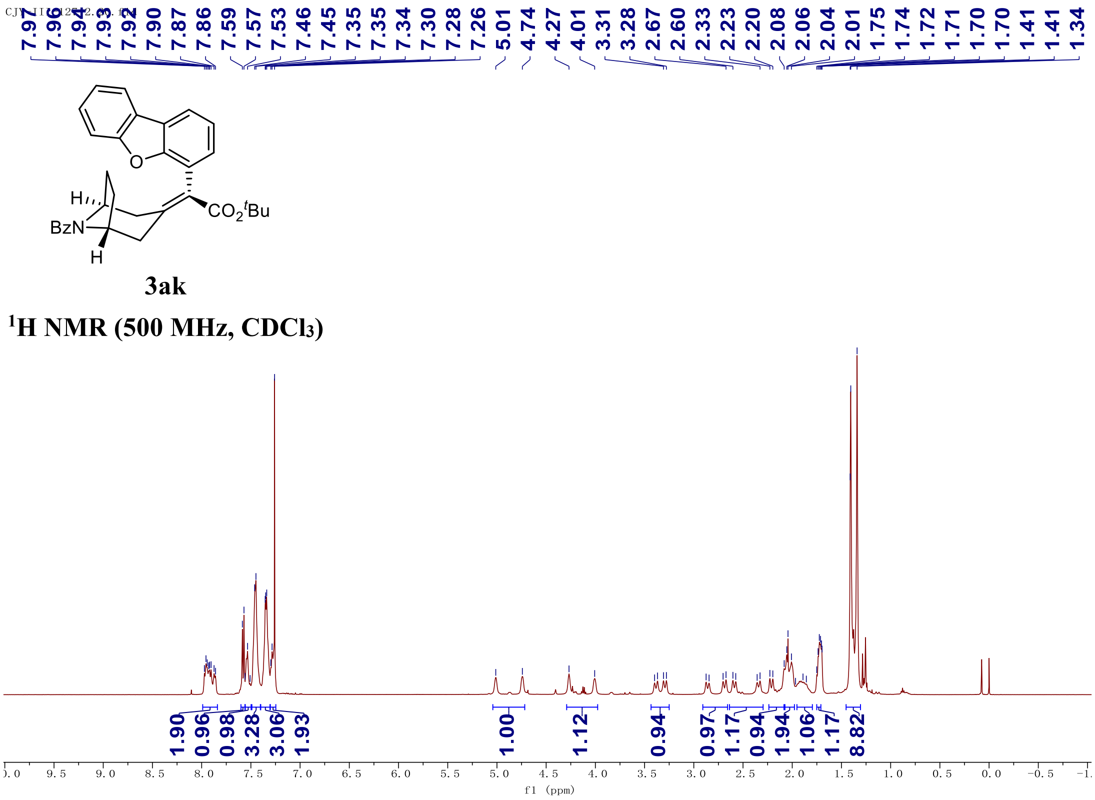

Figure S104 ^{1}H NMR (500 MHz, $CDCl_3$) spectrum for **3ak**

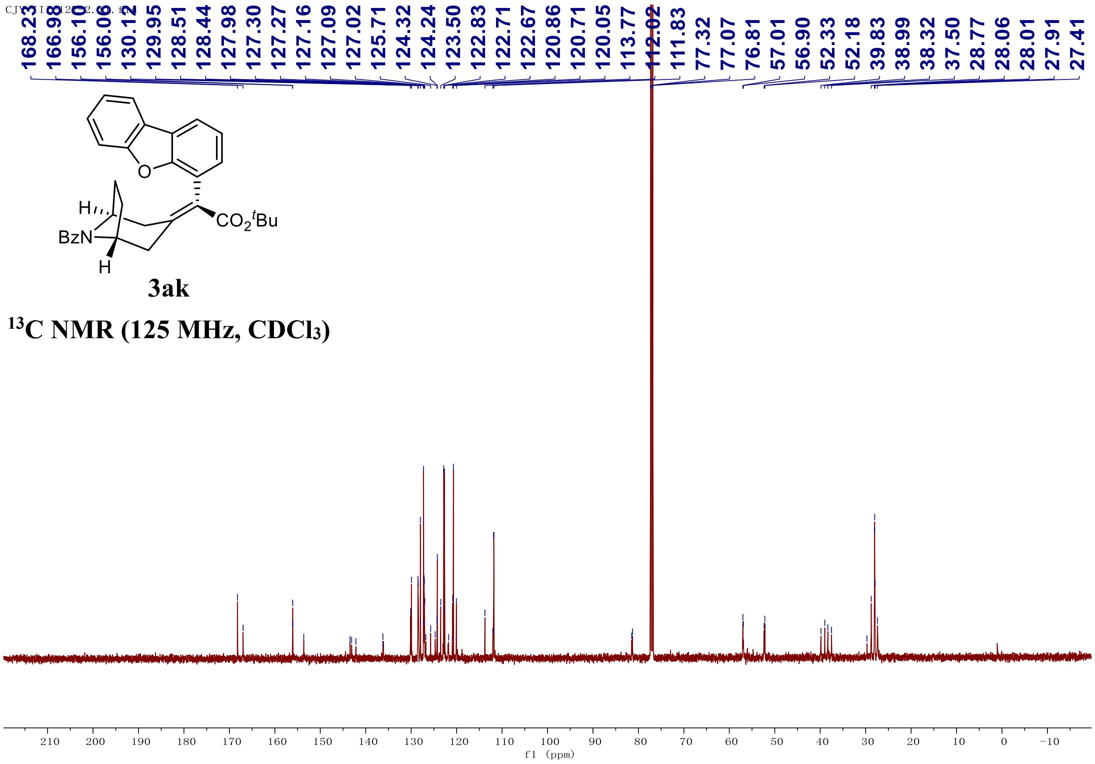

Figure S105 ^{13}C NMR (125 MHz, $CDCl_3$) spectrum for **3ak**

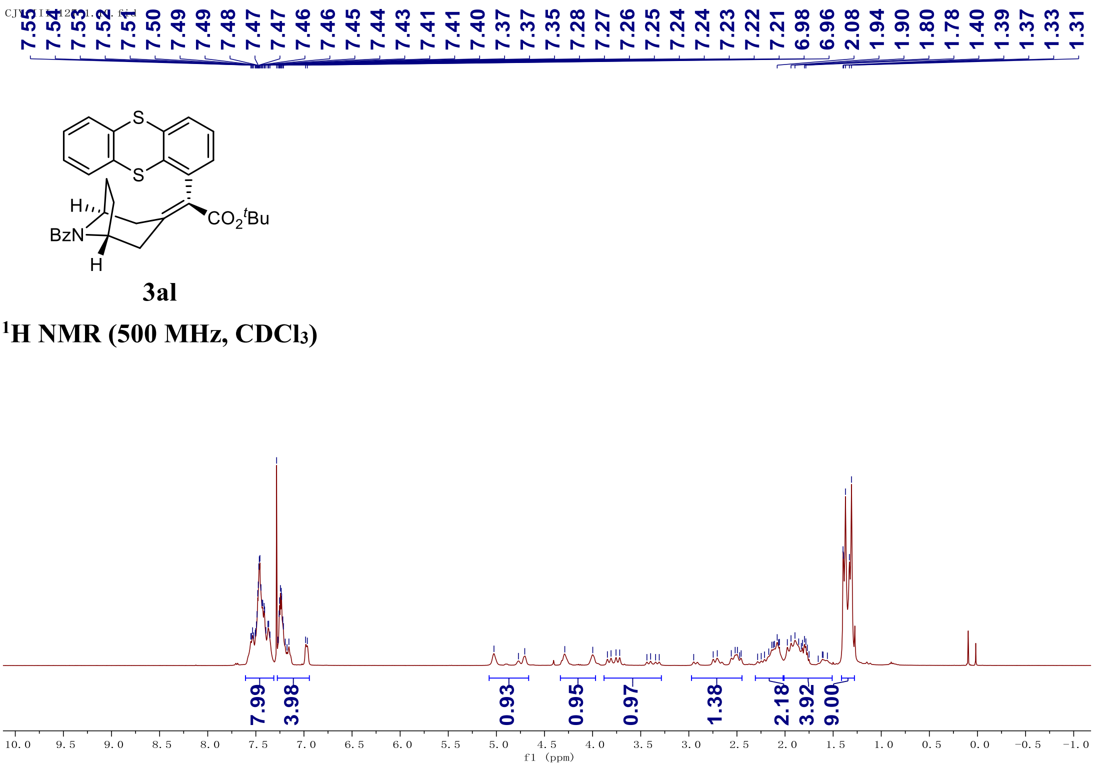

Figure S106 ^{1}H NMR (500 MHz, $CDCl_3$) spectrum for **3al**

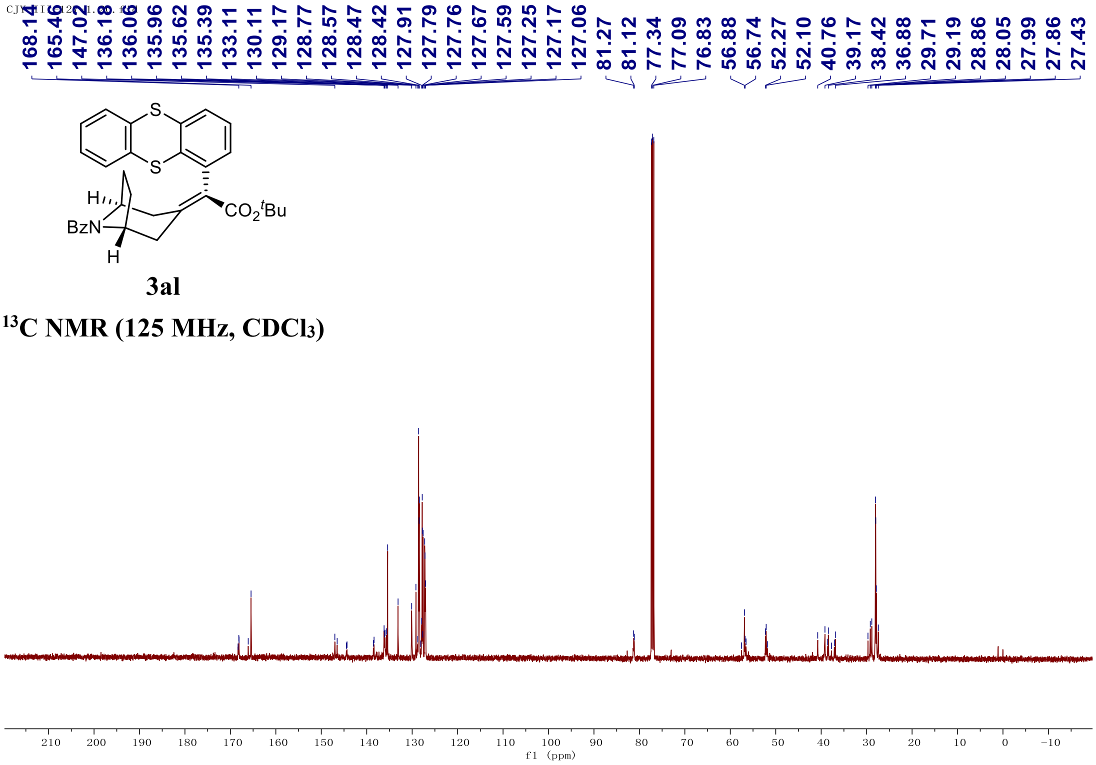

Figure S107 ^{13}C NMR (125 MHz, $CDCl_3$) spectrum for **3al**

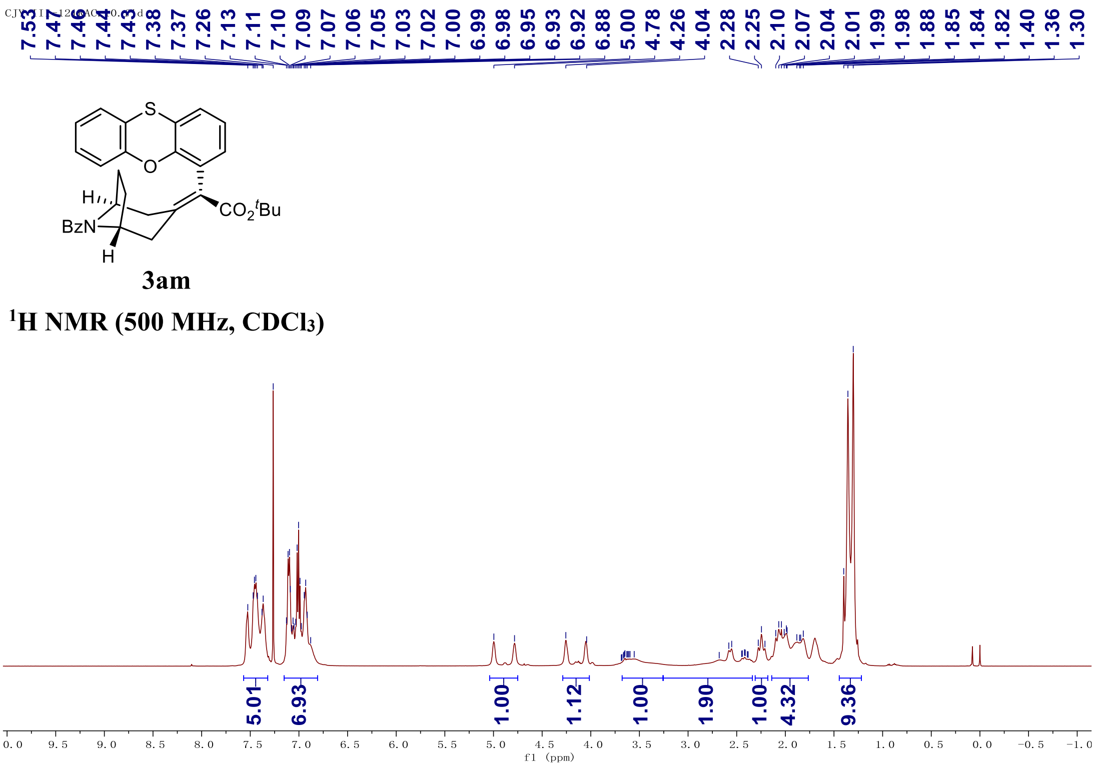

Figure S108 ^{1}H NMR (500 MHz, $CDCl_3$) spectrum for **3am**

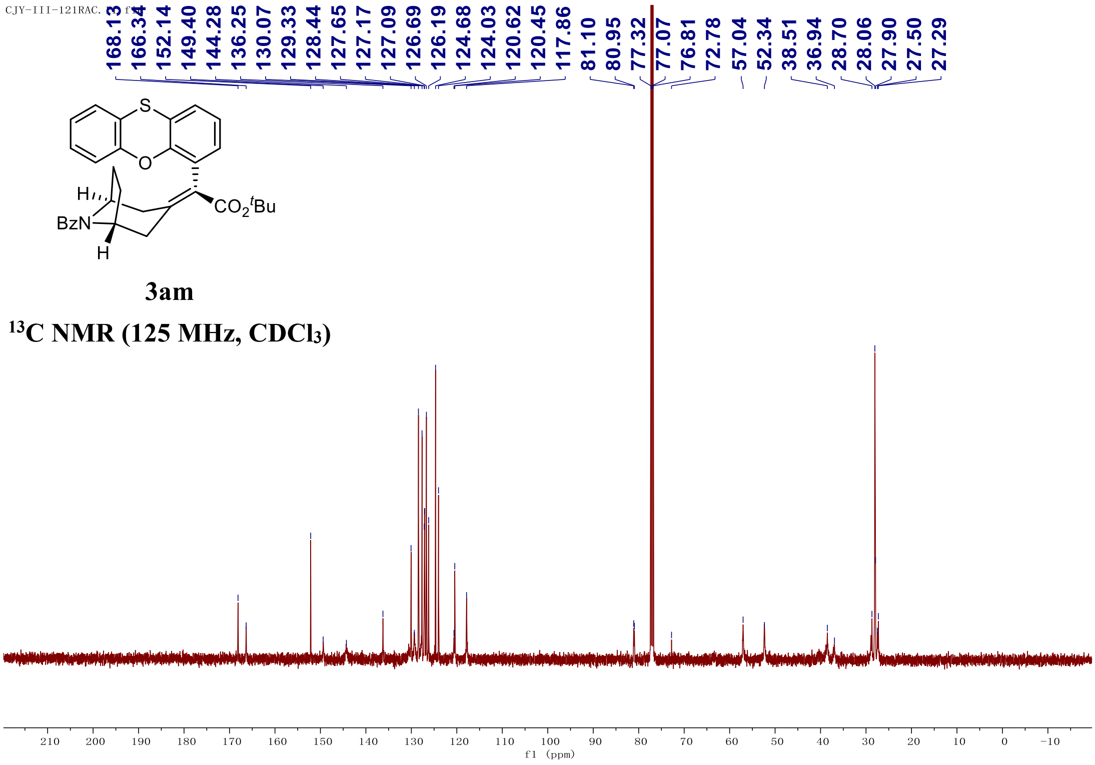

Figure S109 ^{13}C NMR (125 MHz, $CDCl_3$) spectrum for **3am**

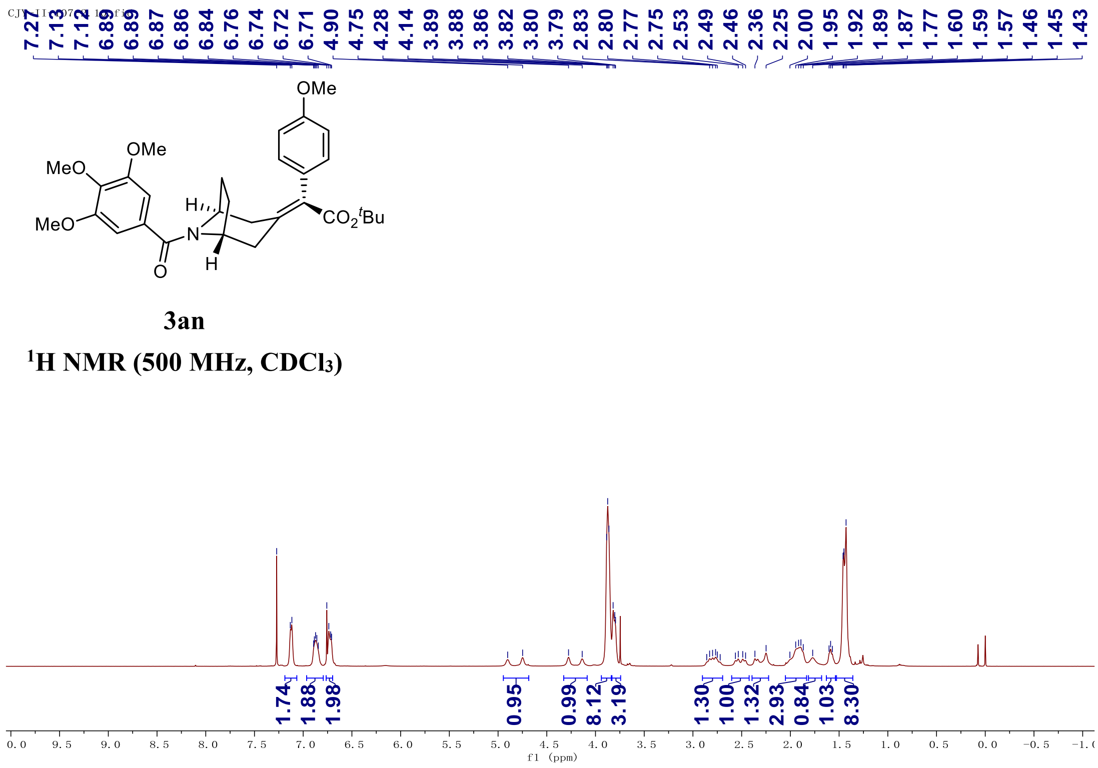

Figure S110 ^{1}H NMR (500 MHz, $CDCl_3$) spectrum for **3an**

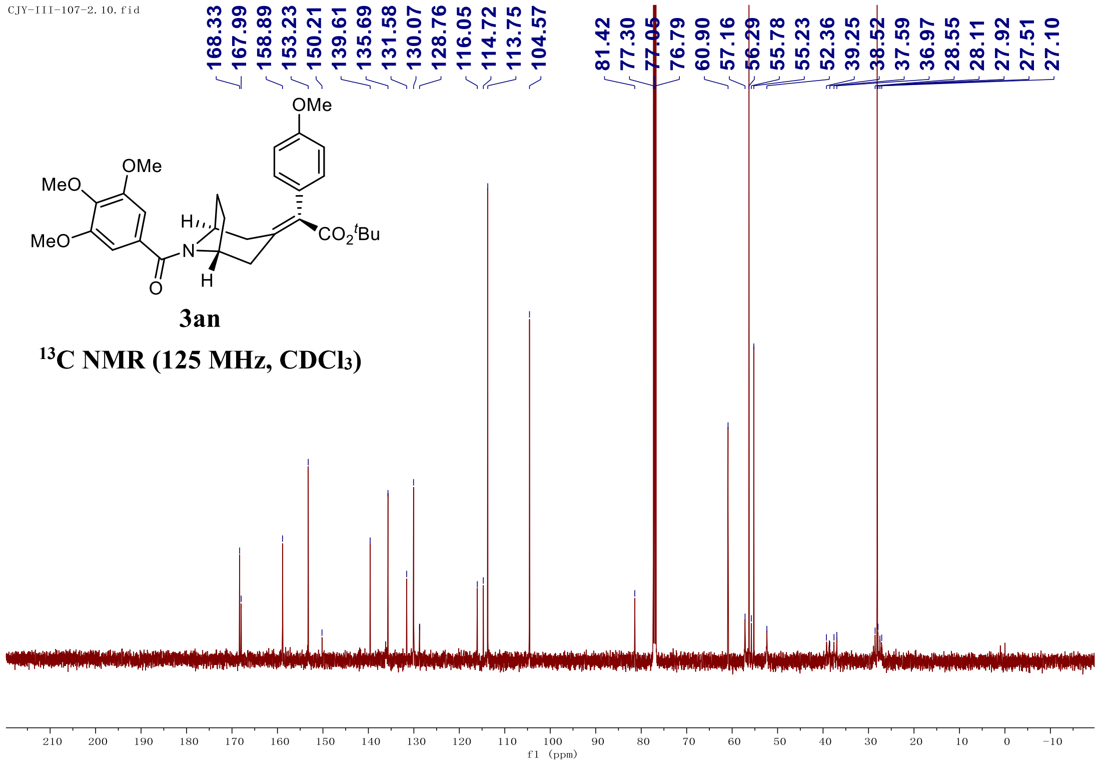

Figure S111 ^{13}C NMR (125 MHz, $CDCl_3$) spectrum for **3an**

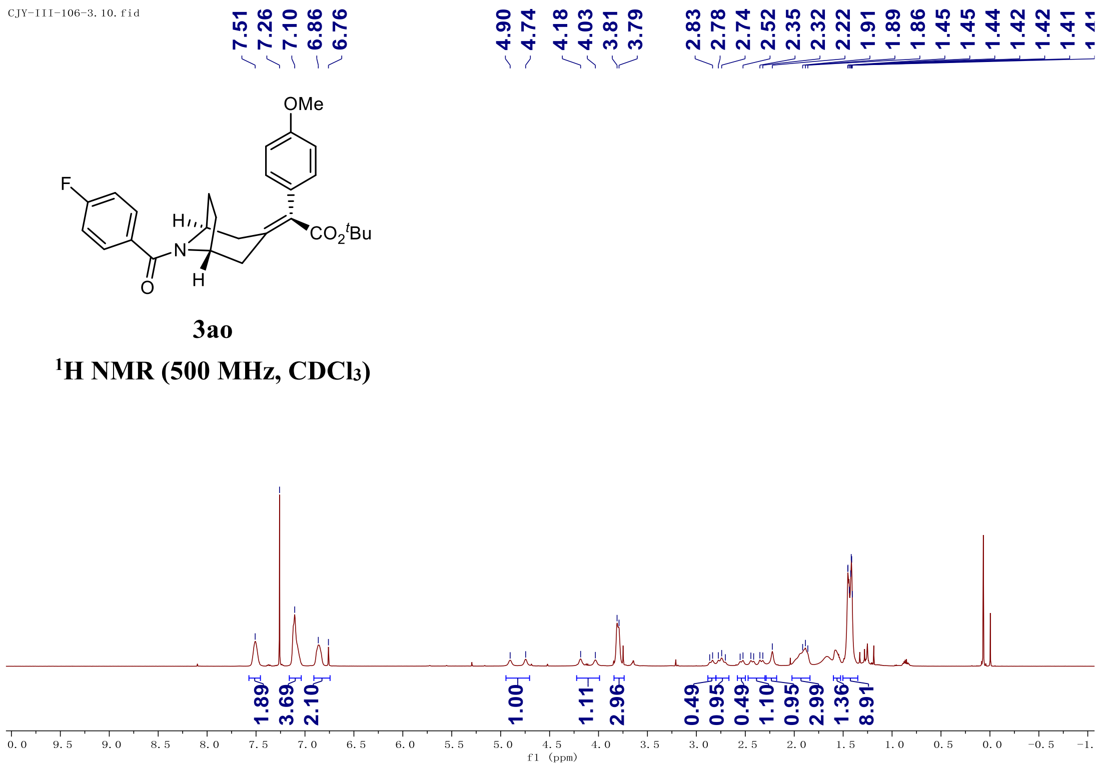

Figure S112 ^{1}H NMR (500 MHz, $CDCl_3$) spectrum for **3ao**

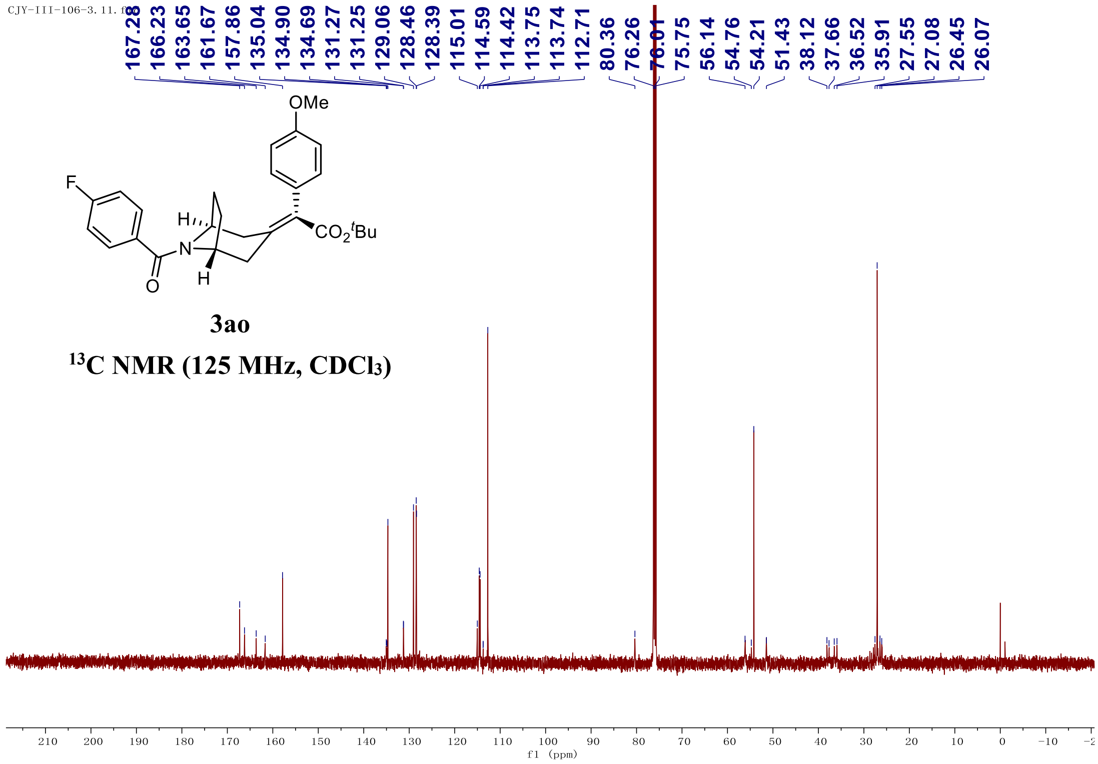

Figure S113 ^{13}C NMR (125 MHz, $CDCl_3$) spectrum for **3ao**

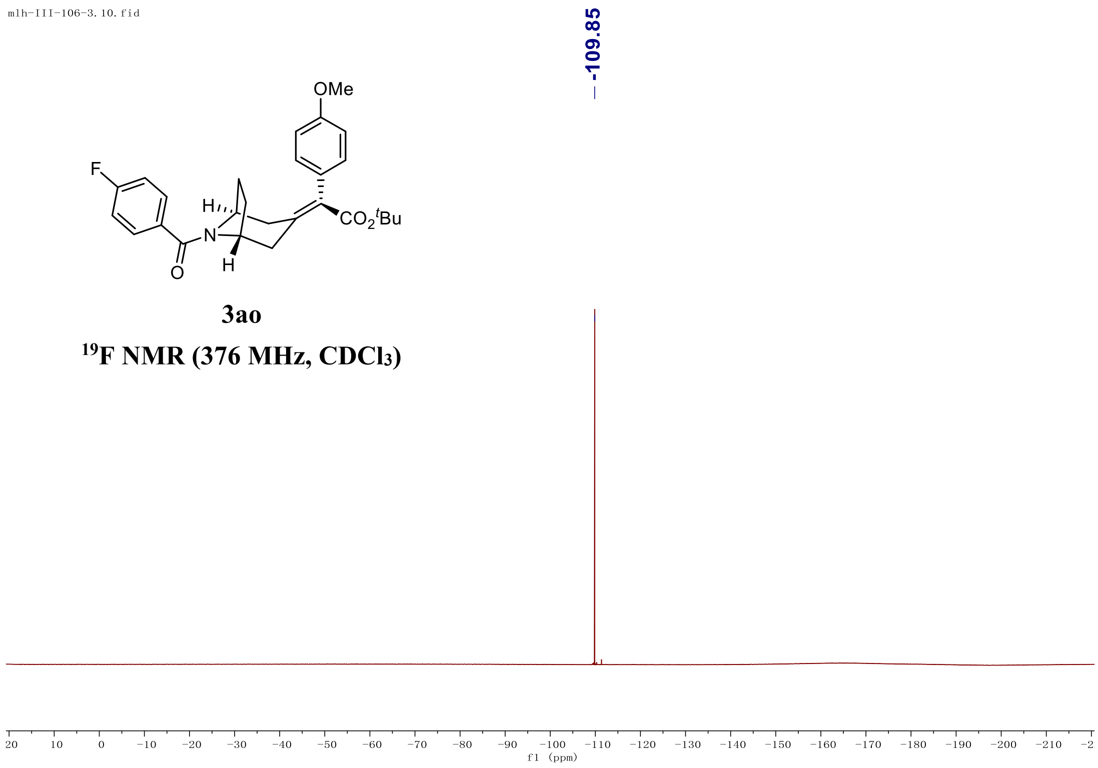

Figure S114 ^{19}F NMR (376 MHz, $CDCl_3$) spectrum for **3ao**

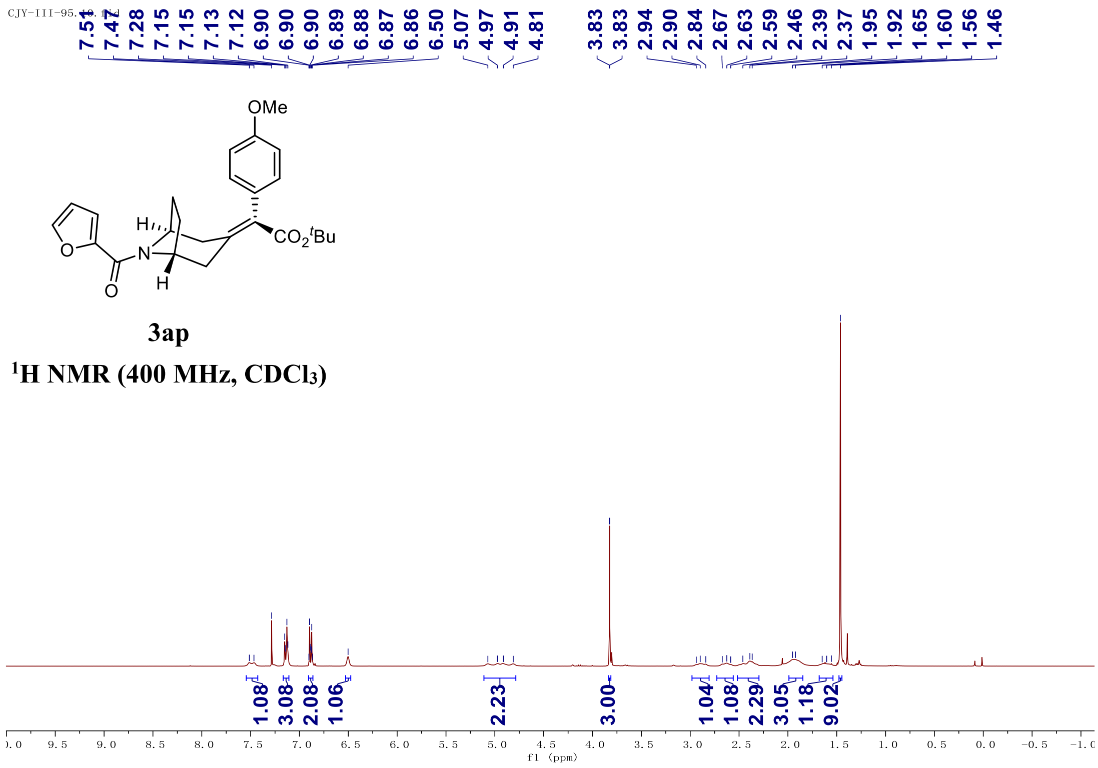

Figure S115 ^{1}H NMR (400 MHz, $CDCl_3$) spectrum for **3ap**

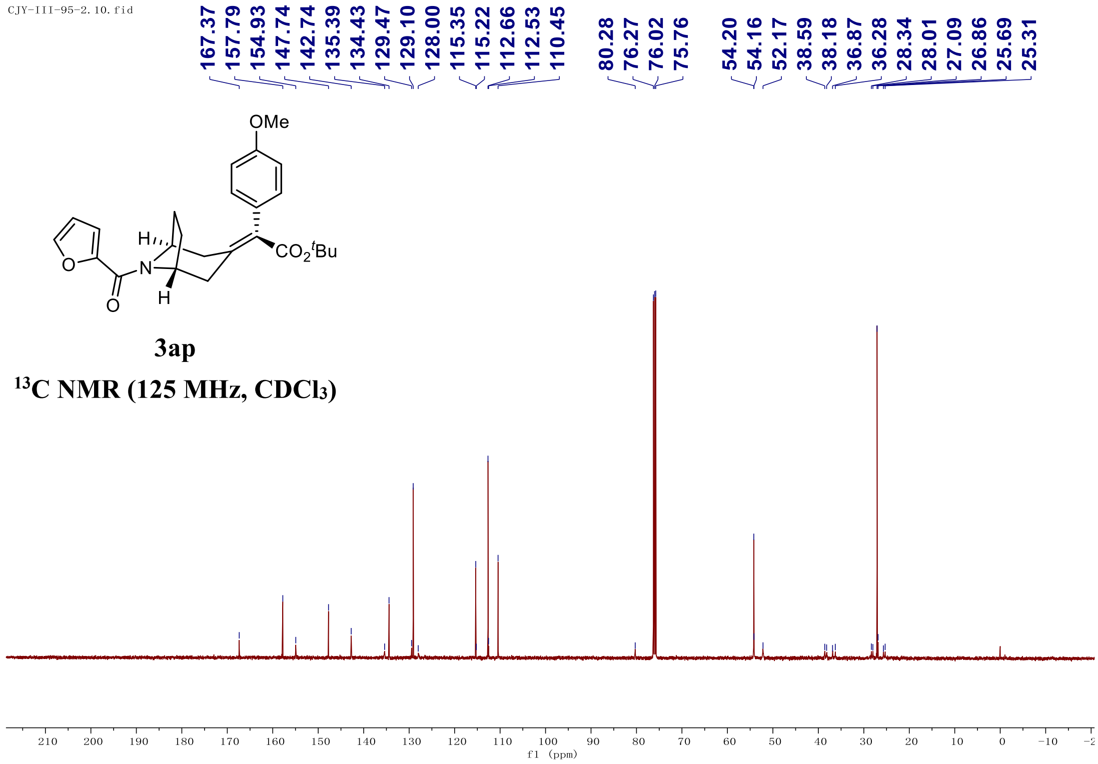

Figure S116 ^{13}C NMR (125 MHz, $CDCl_3$) spectrum for **3ap**

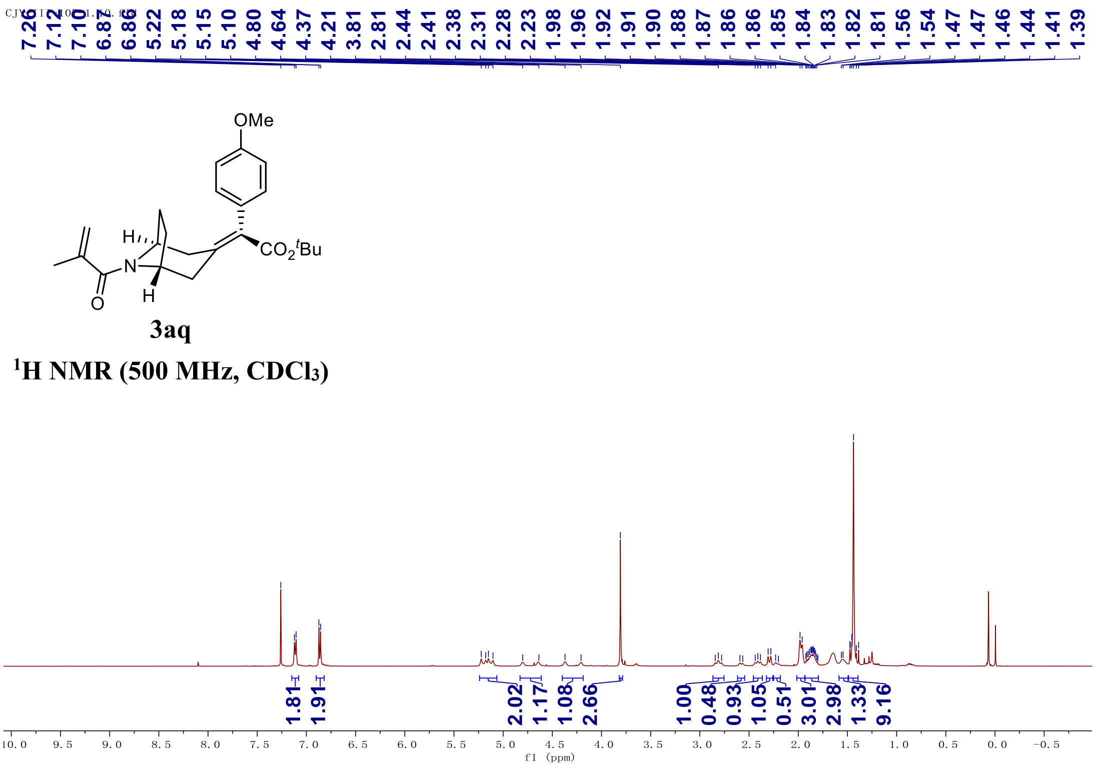

Figure S117 ^{1}H NMR (500 MHz, $CDCl_3$) spectrum for **3aq**

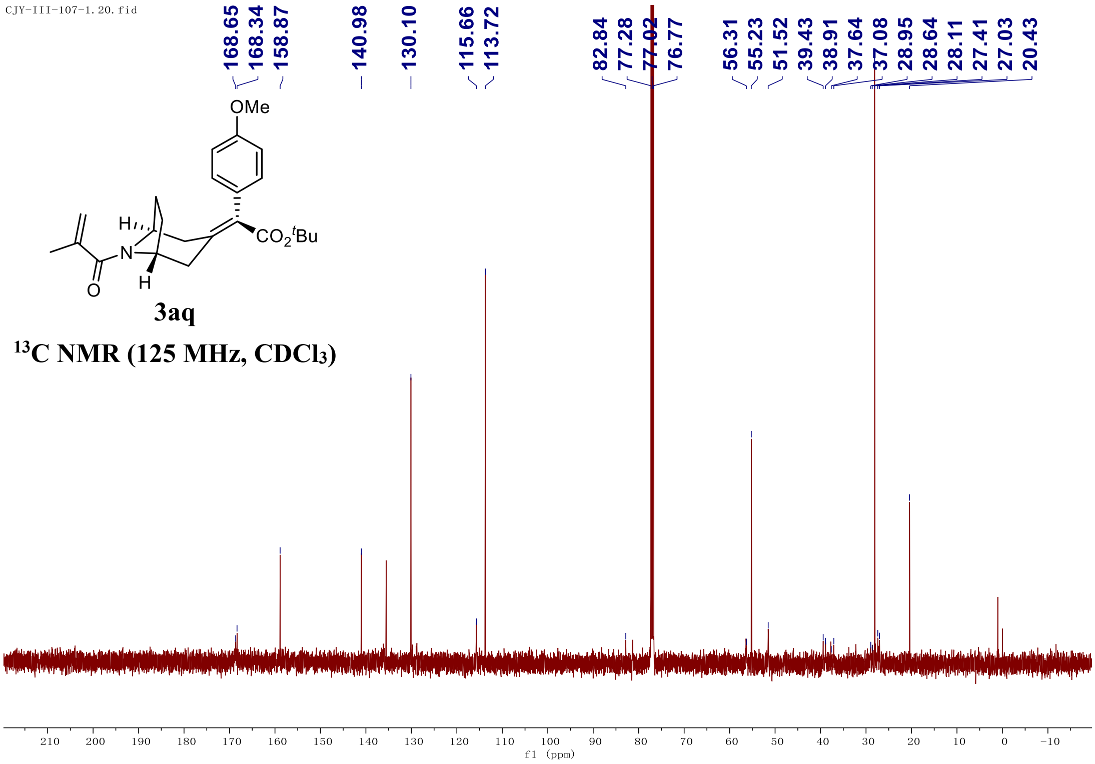

Figure S118 ^{13}C NMR (125 MHz, $CDCl_3$) spectrum for **3aq**

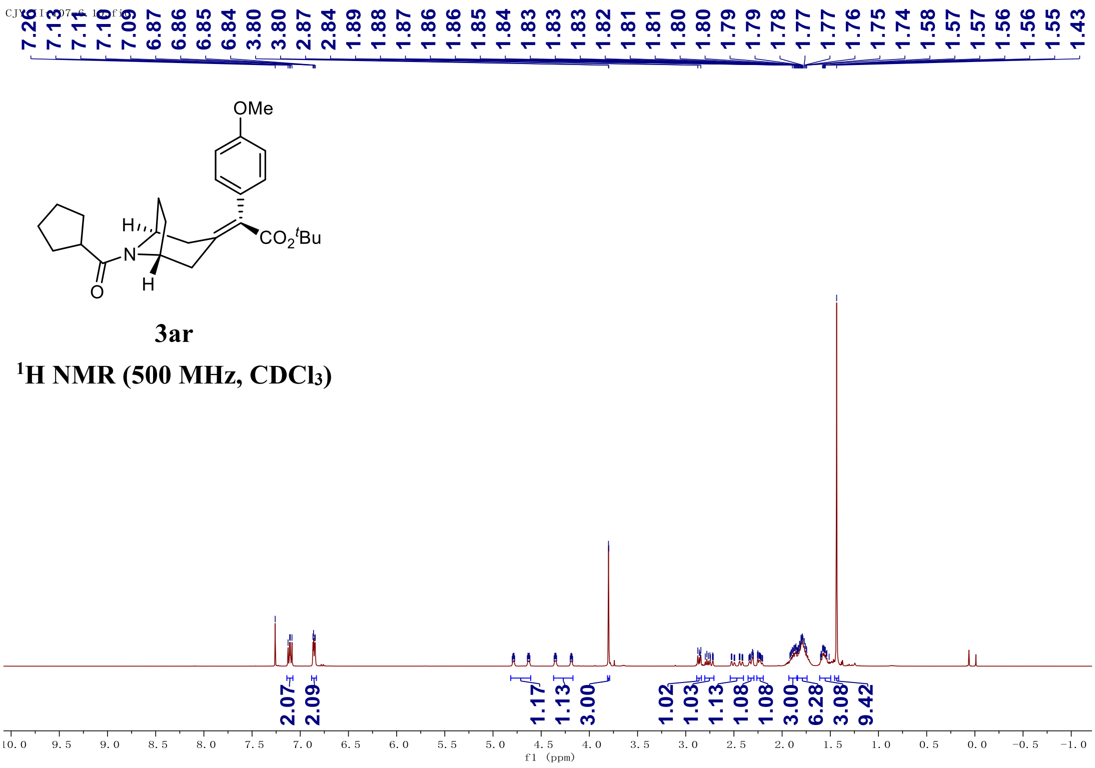

Figure S119 ^{1}H NMR (500 MHz, $CDCl_3$) spectrum for **3ar**

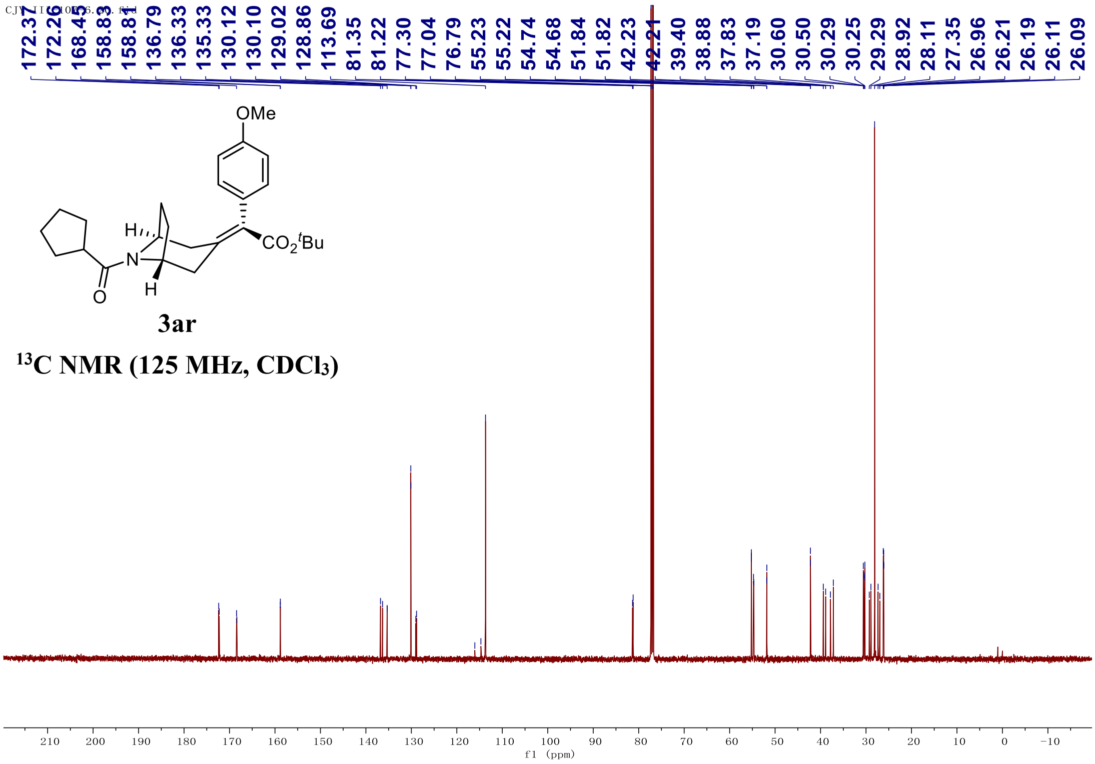

Figure S120 ^{13}C NMR (125 MHz, $CDCl_3$) spectrum for **3ar**

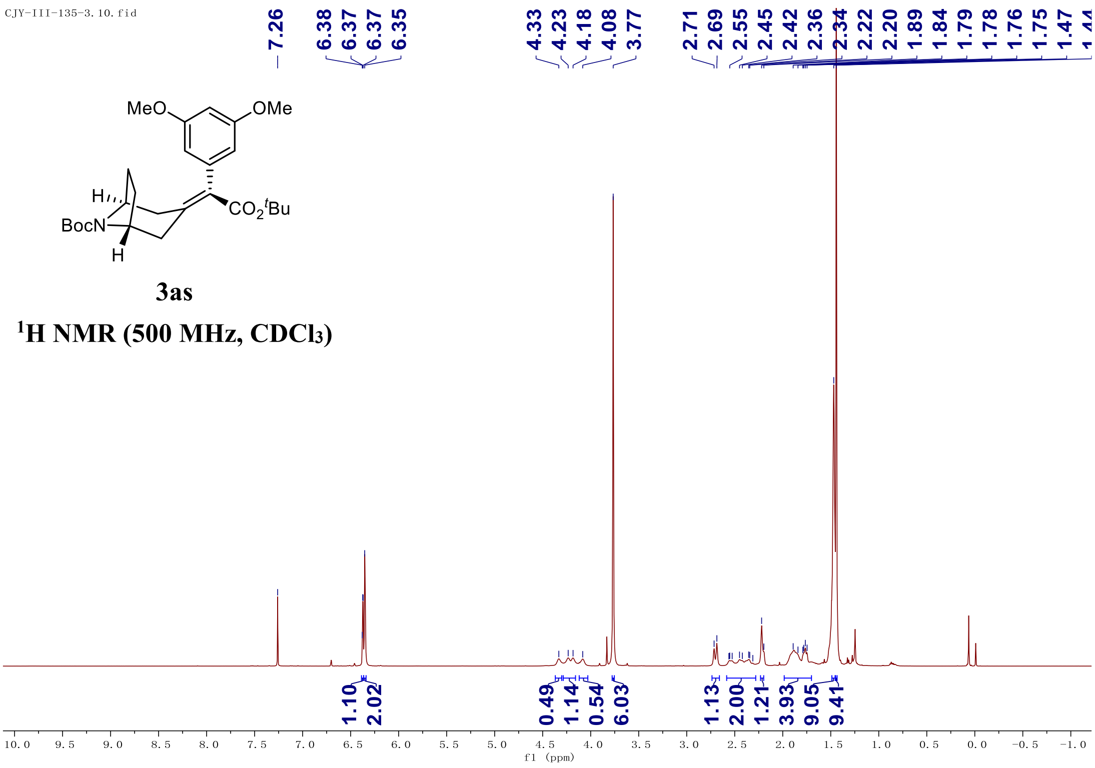

Figure S121 ^{1}H NMR (500 MHz, $CDCl_3$) spectrum for **3as**

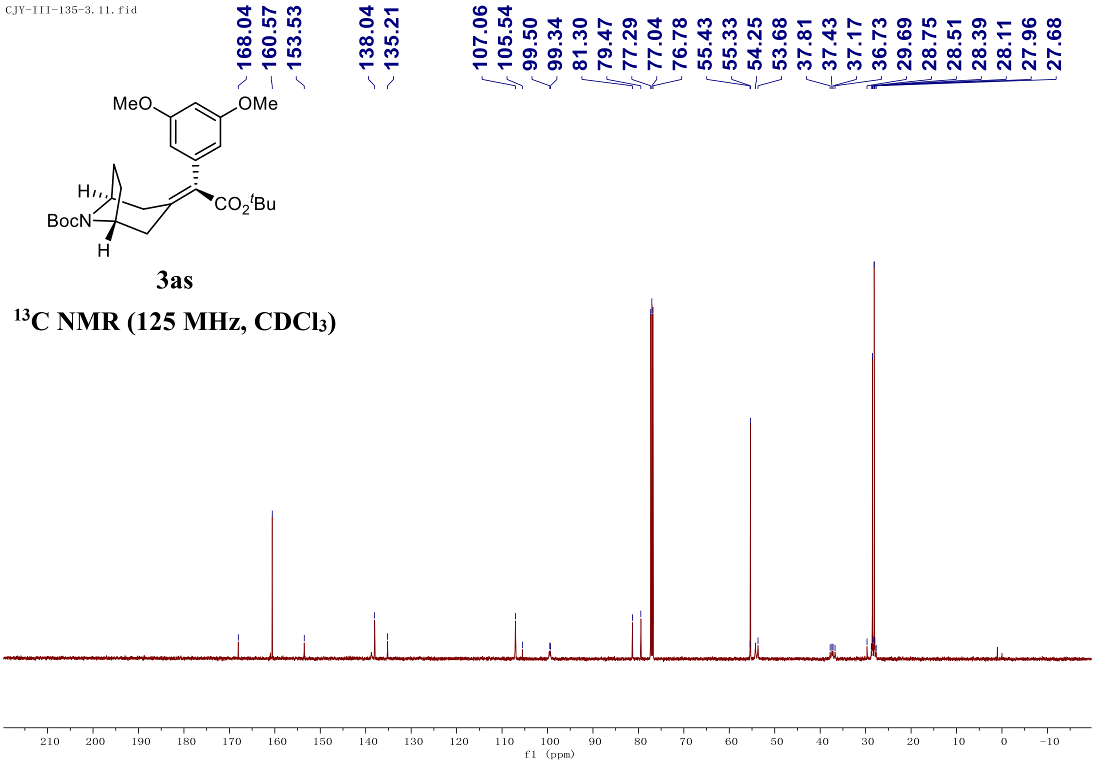

Figure S122 ^{13}C NMR (125 MHz, $CDCl_3$) spectrum for **3as**

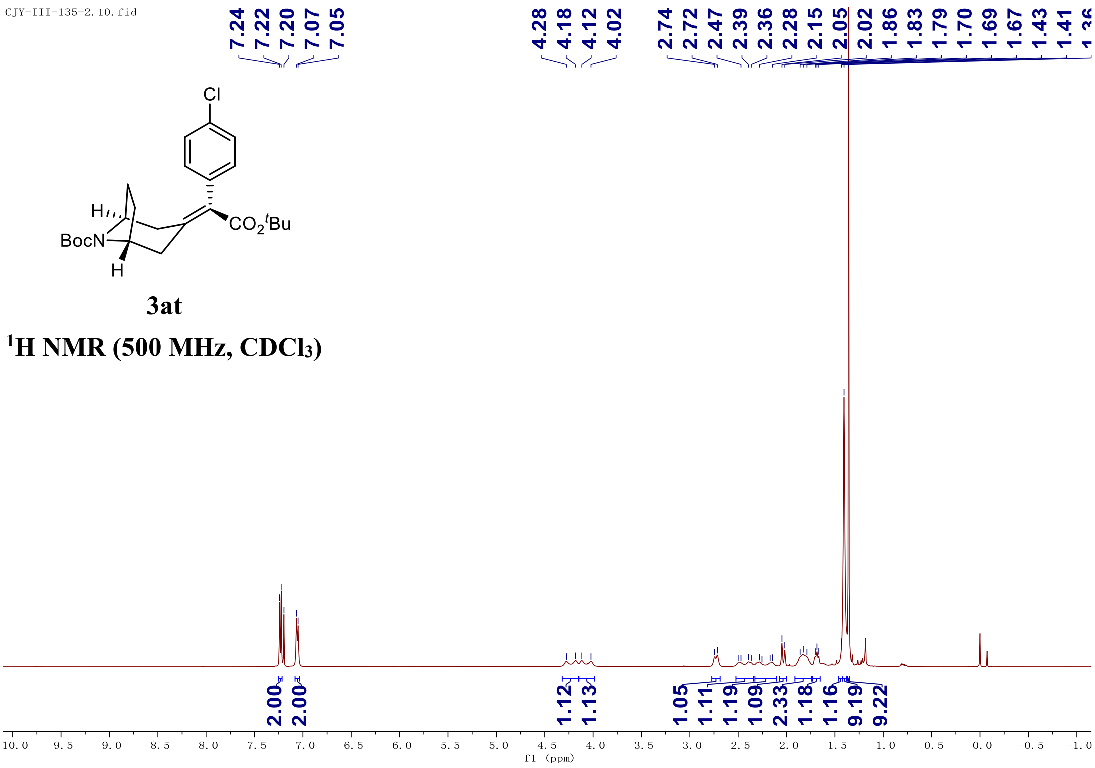

Figure S123 ^{1}H NMR (500 MHz, $CDCl_3$) spectrum for **3at**

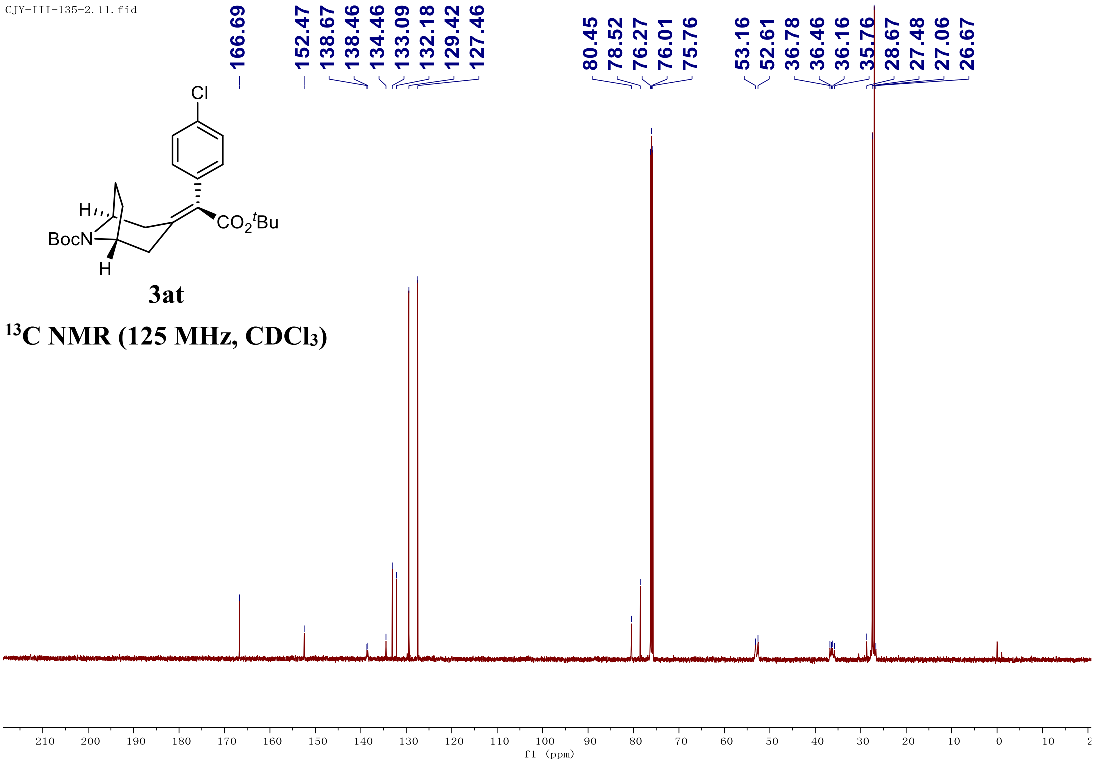

Figure S124 ^{13}C NMR (125 MHz, $CDCl_3$) spectrum for **3at**

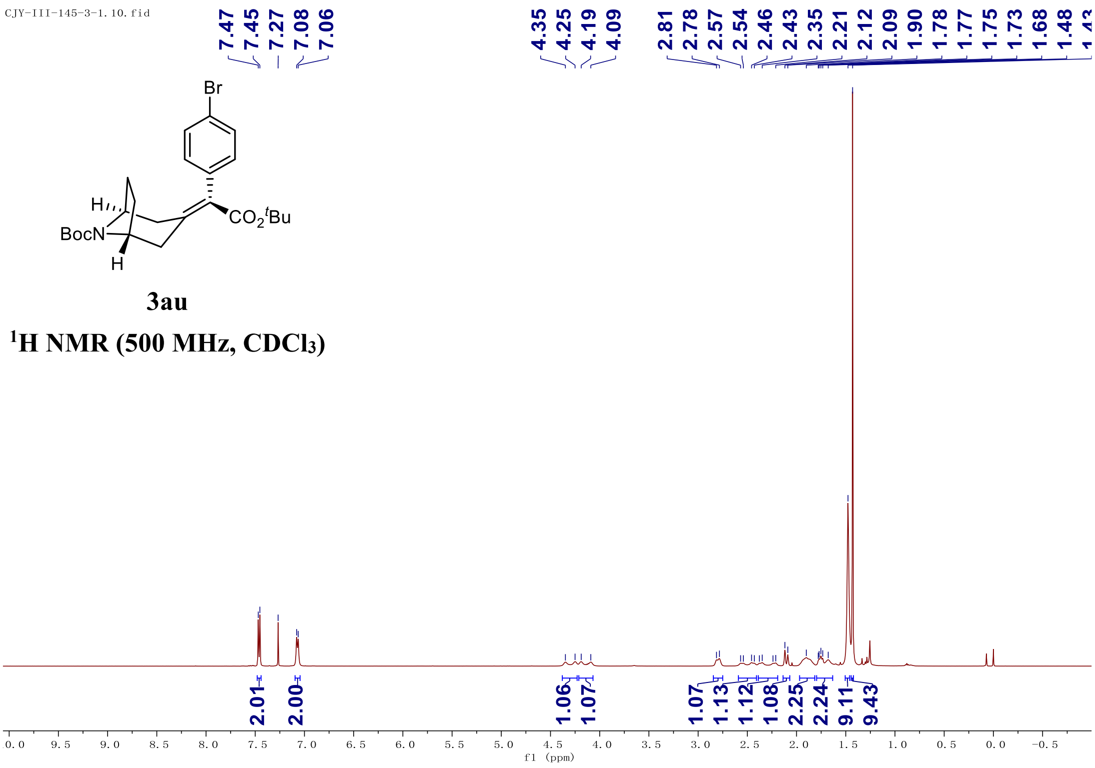

Figure S125 ^{1}H NMR (500 MHz, $CDCl_3$) spectrum for **3au**

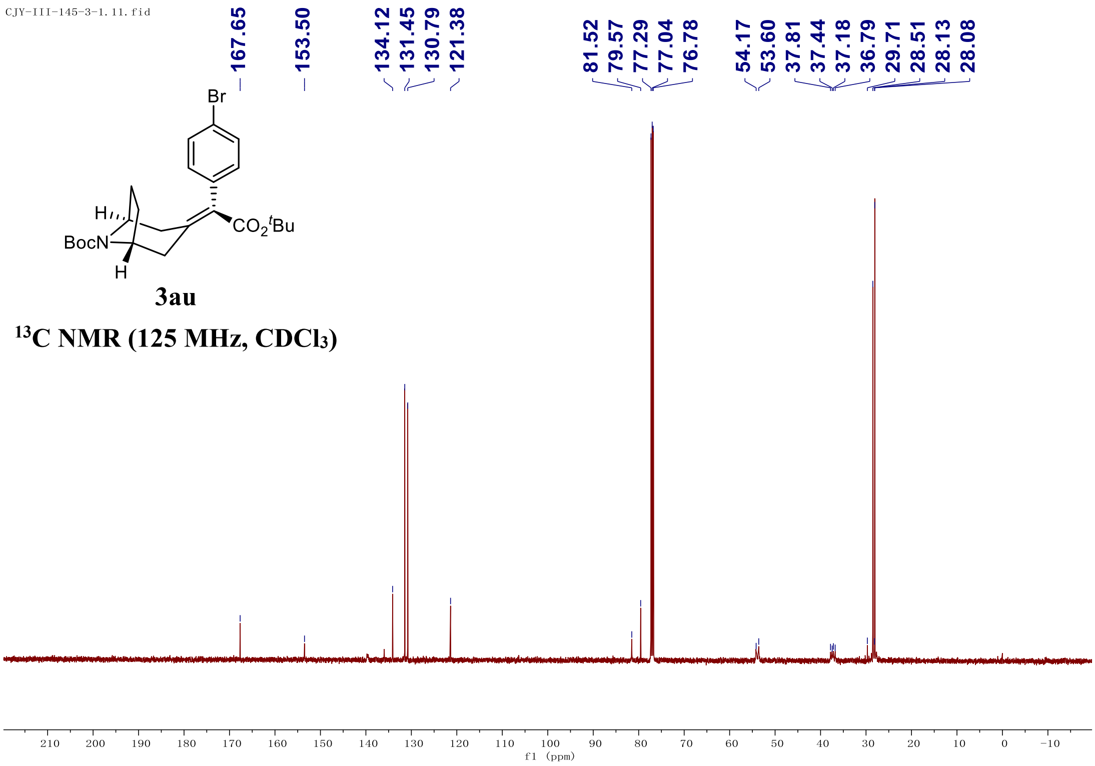

Figure S126 ^{13}C NMR (125 MHz, $CDCl_3$) spectrum for **3au**

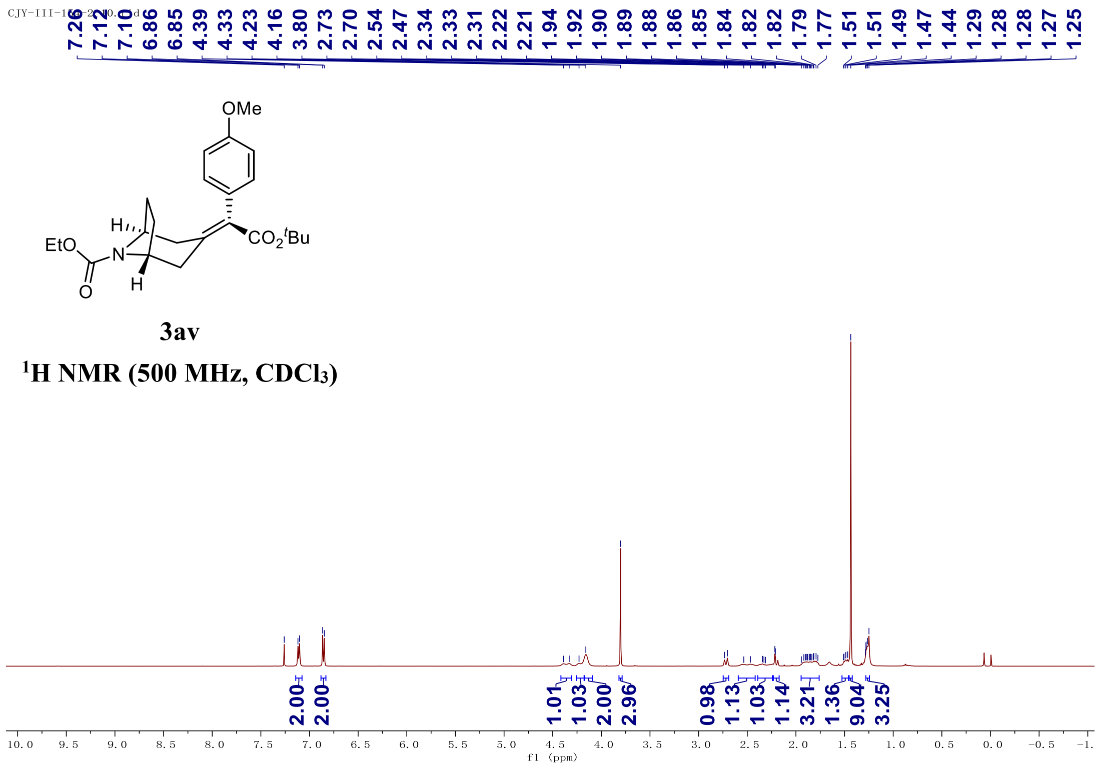

Figure S127 ^{1}H NMR (500 MHz, $CDCl_3$) spectrum for **3av**

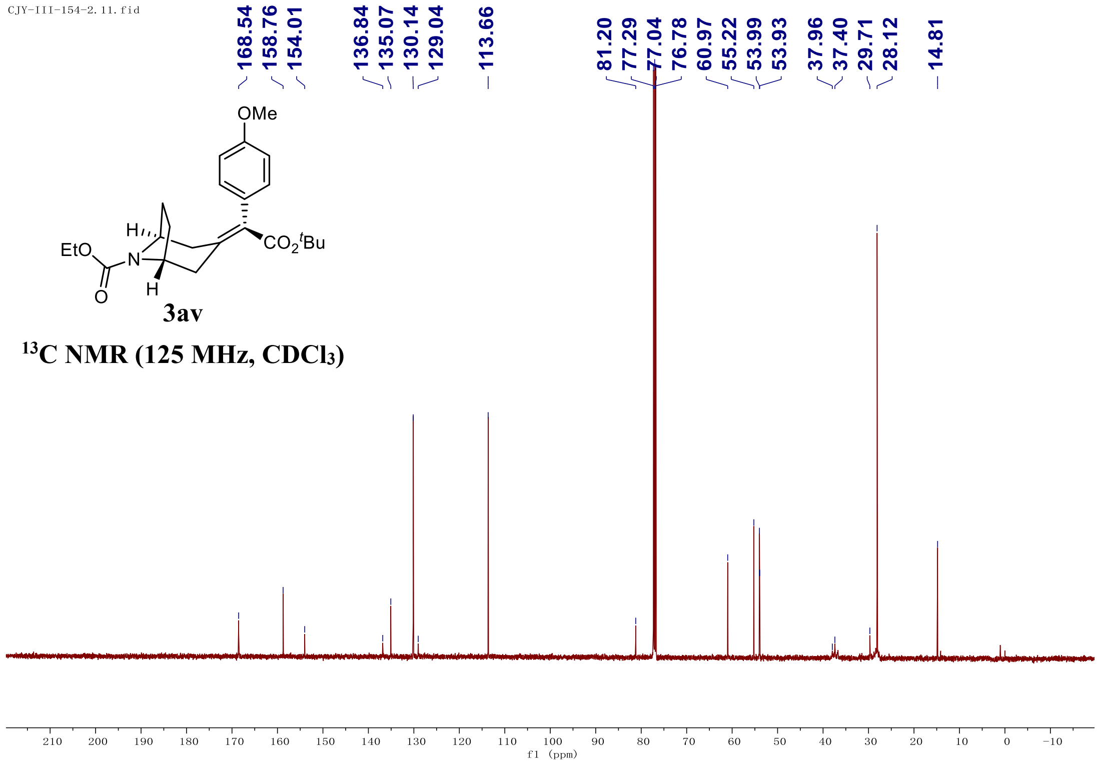

Figure S128 ^{13}C NMR (125 MHz, $CDCl_3$) spectrum for **3av**

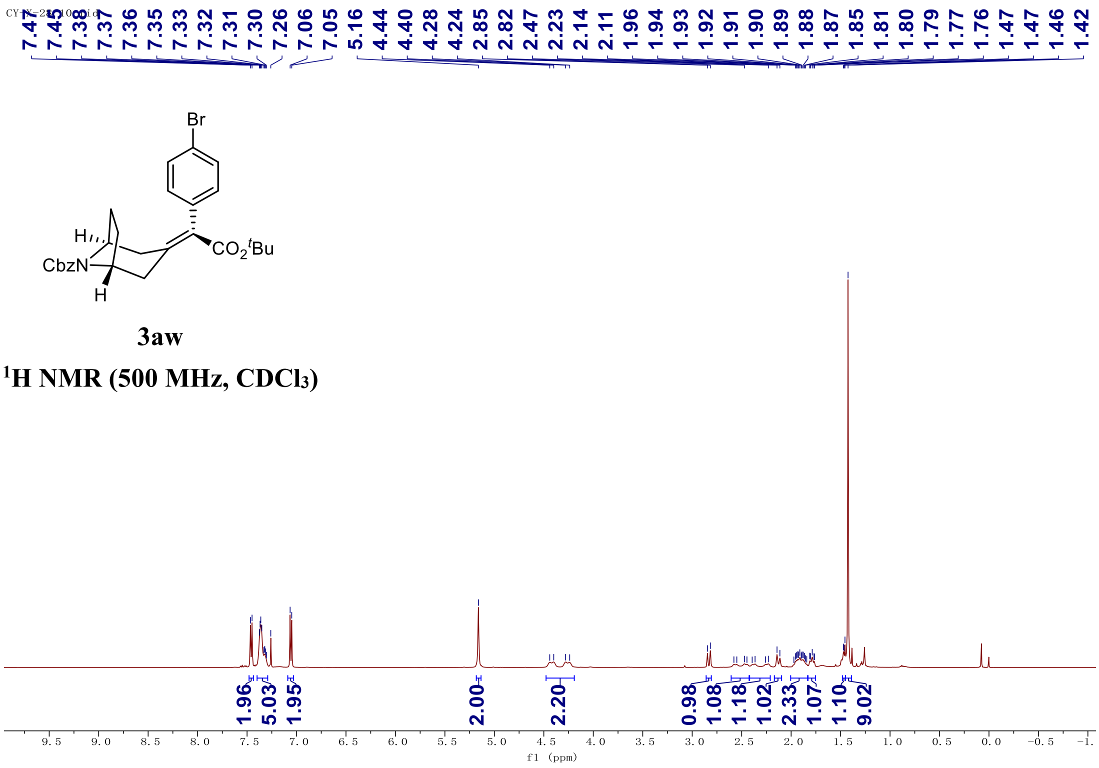

Figure S129 ^{1}H NMR (500 MHz, $CDCl_3$) spectrum for **3aw**

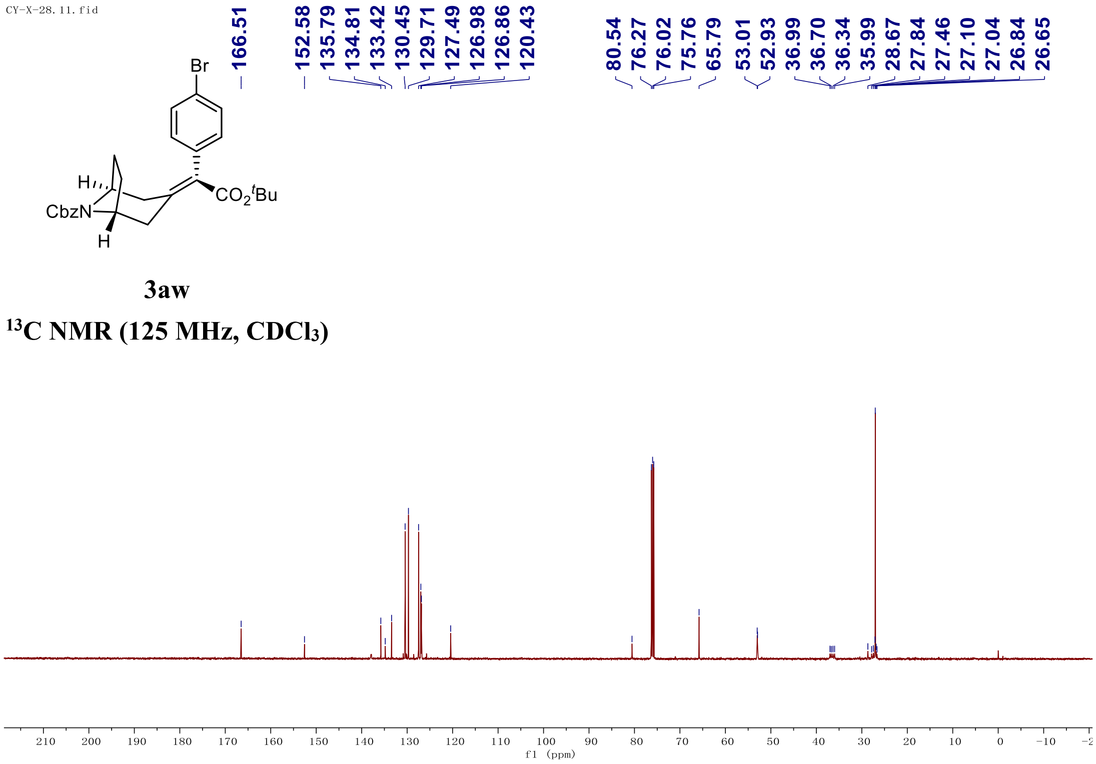

Figure S130 ^{13}C NMR (125 MHz, $CDCl_3$) spectrum for **3aw**

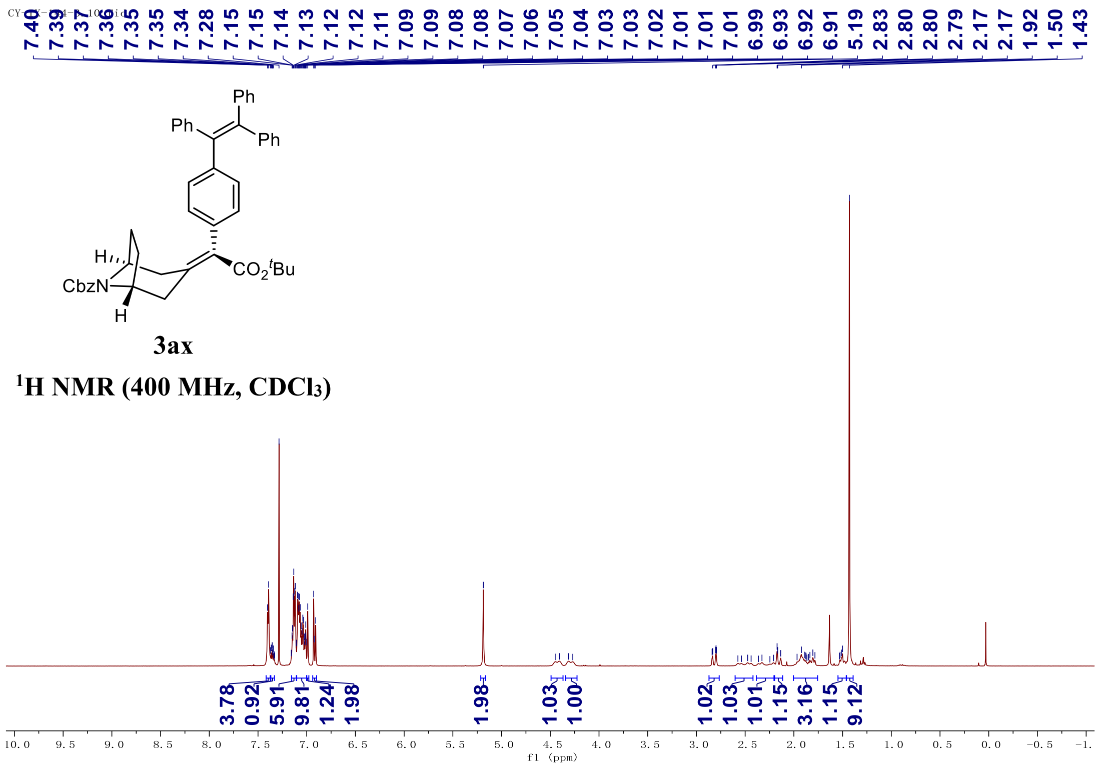

Figure S131 ^{1}H NMR (400 MHz, $CDCl_3$) spectrum for **3ax**

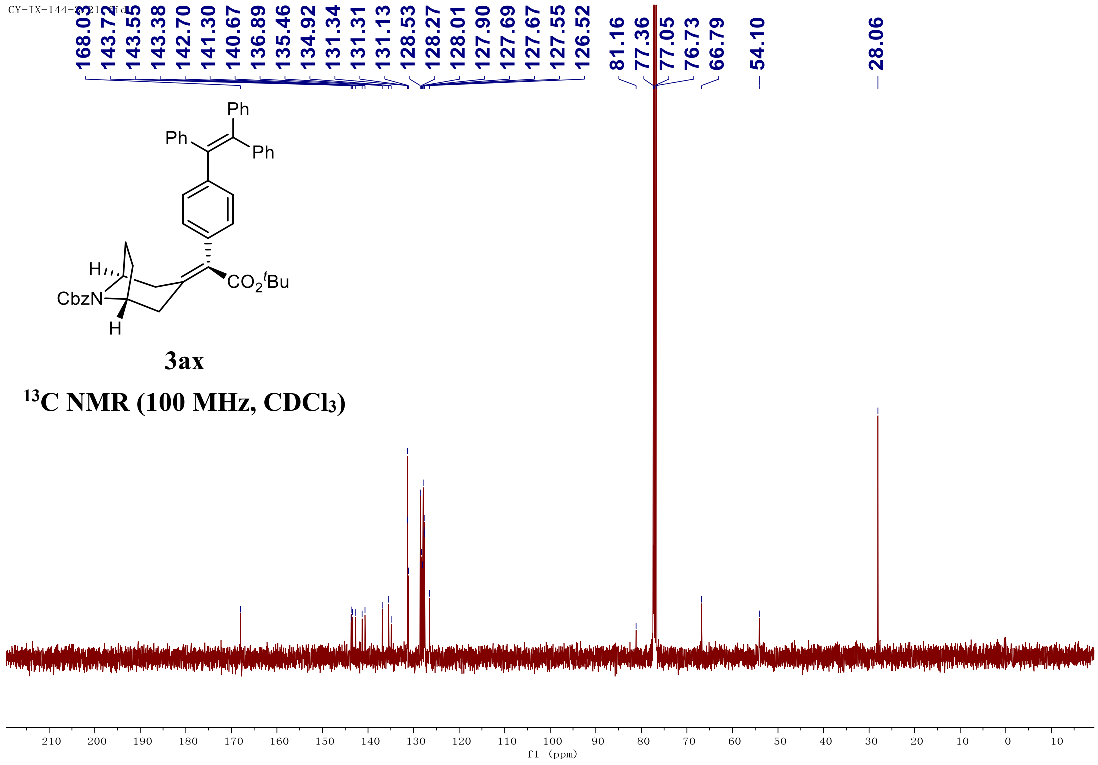

Figure S132 ^{13}C NMR (100 MHz, $CDCl_3$) spectrum for **3ax**

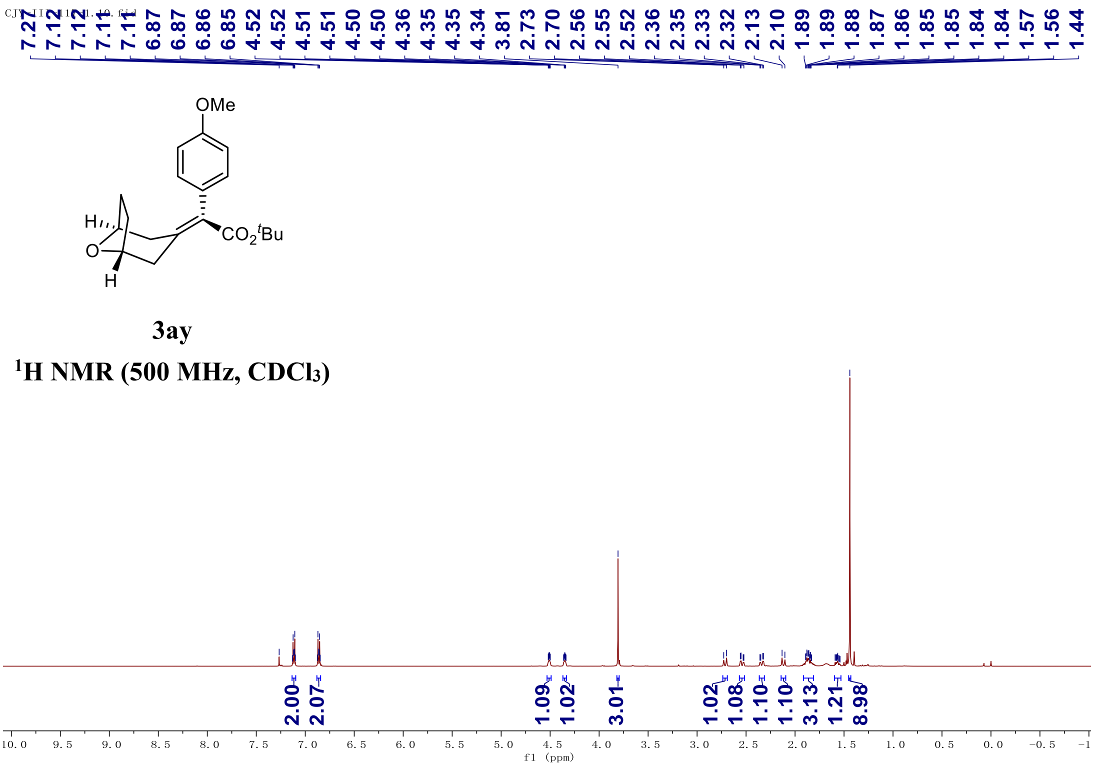

Figure S133 ^{1}H NMR (500 MHz, $CDCl_3$) spectrum for **3ay**

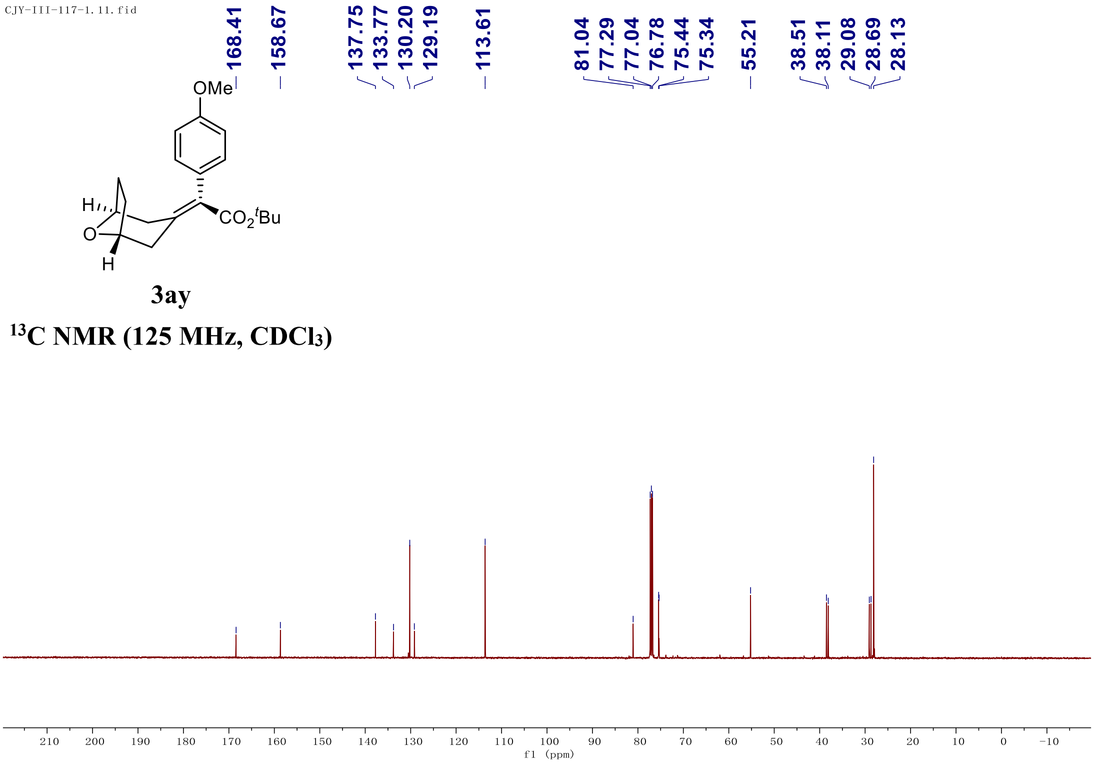

Figure S134 ^{13}C NMR (125 MHz, $CDCl_3$) spectrum for **3ay**

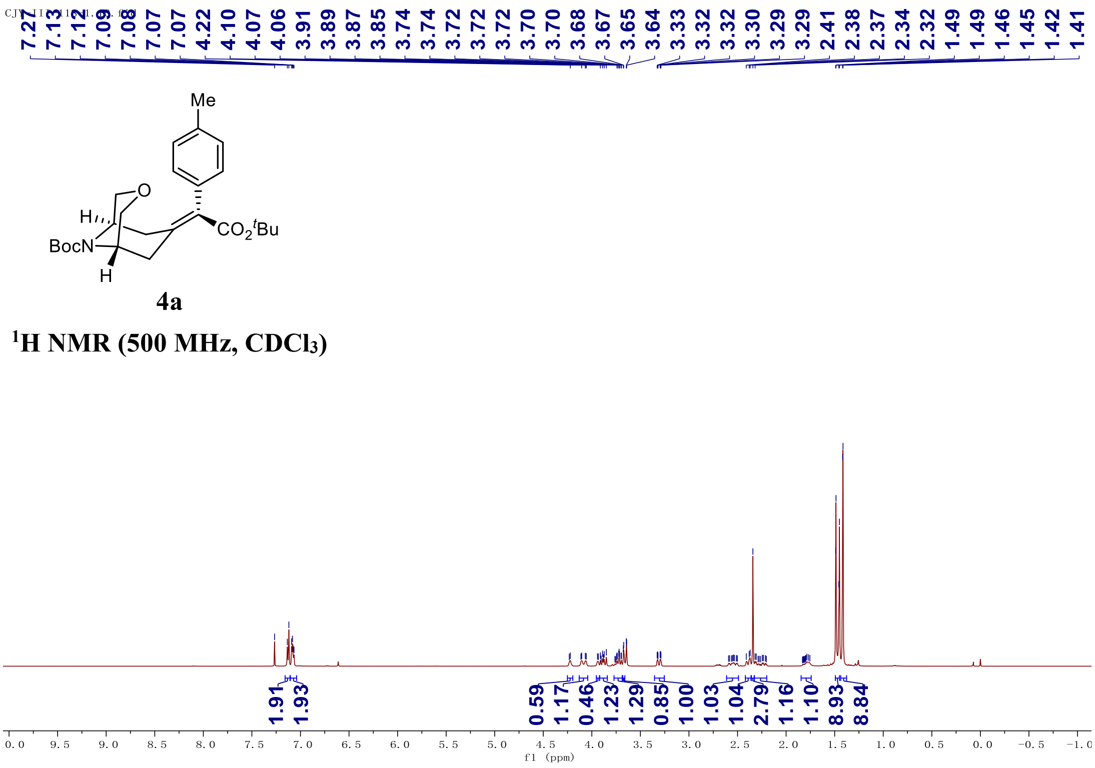

Figure S135 ^{1}H NMR (500 MHz, $CDCl_3$) spectrum for **4a**

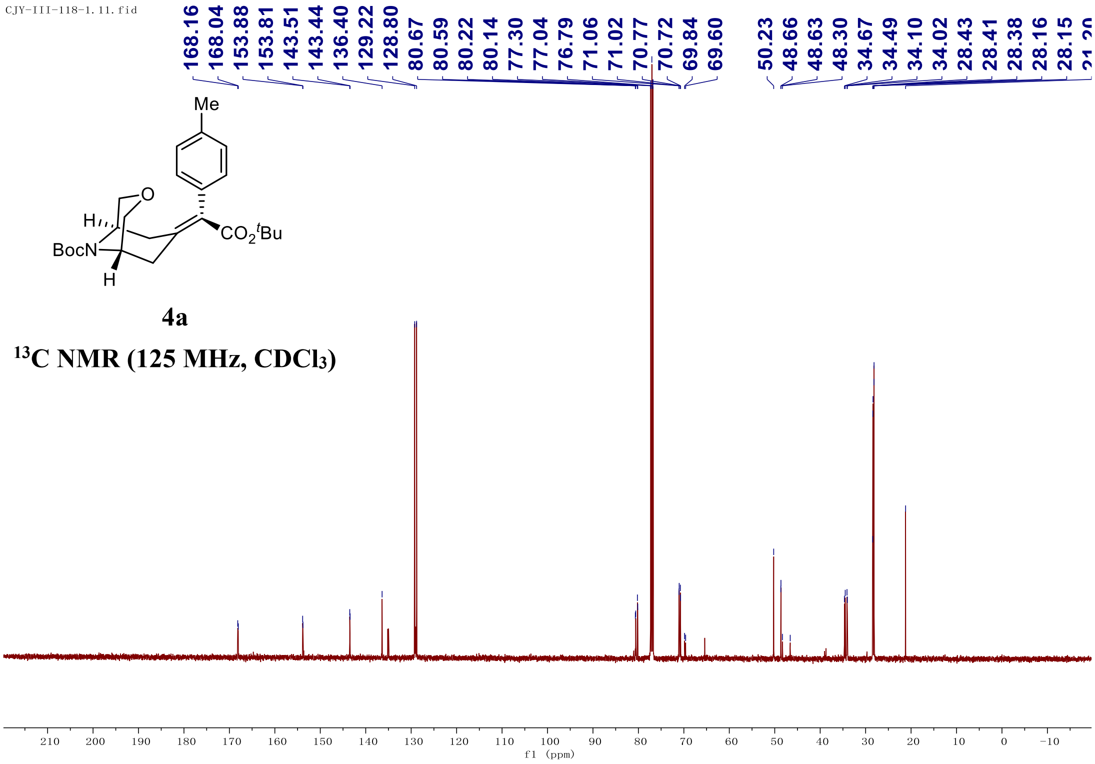

Figure S136 ^{13}C NMR (125 MHz, $CDCl_3$) spectrum for **4a**

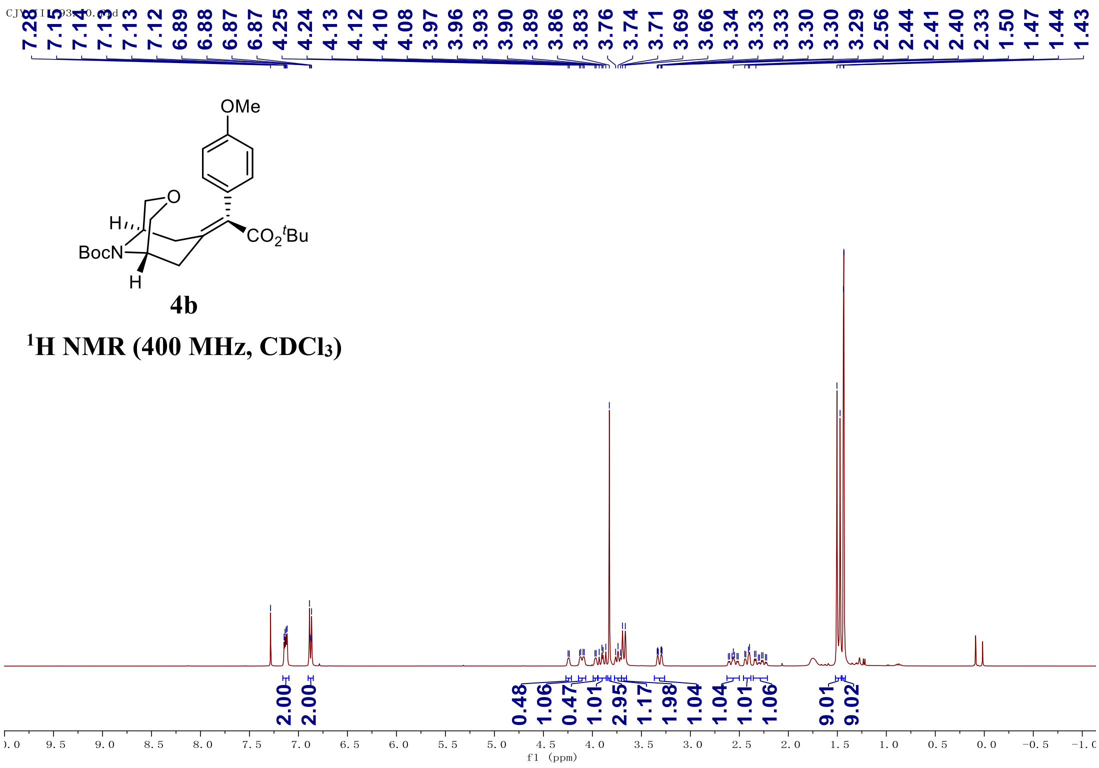

Figure S137 ^1H NMR (400 MHz, $CDCl_3$) spectrum for **4b**

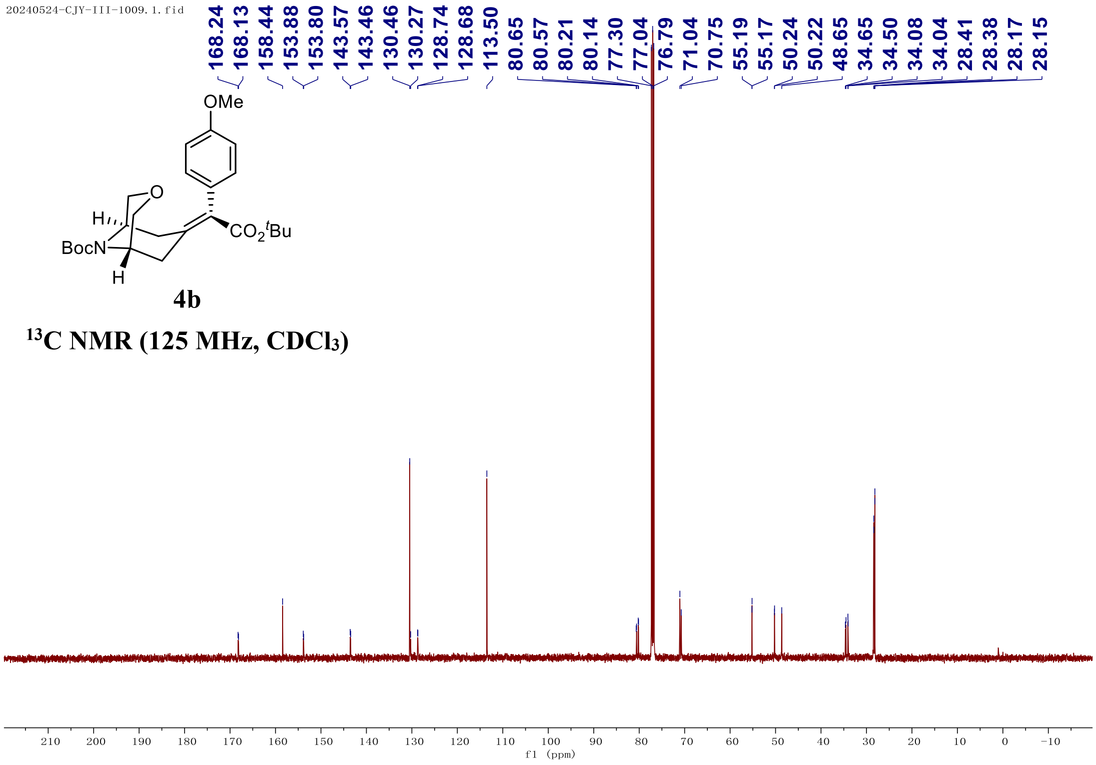

Figure S138 ^{13}C NMR (125 MHz, $CDCl_3$) spectrum for **4b**

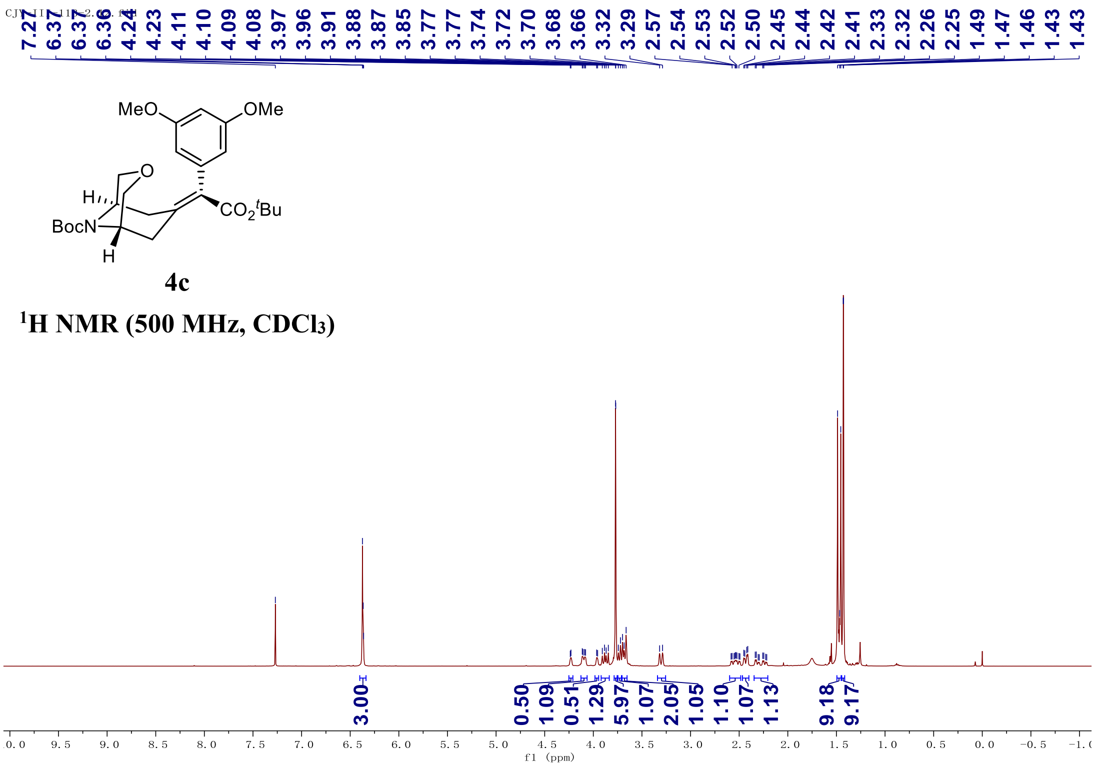

Figure S139 ^{1}H NMR (500 MHz, $CDCl_3$) spectrum for **4c**

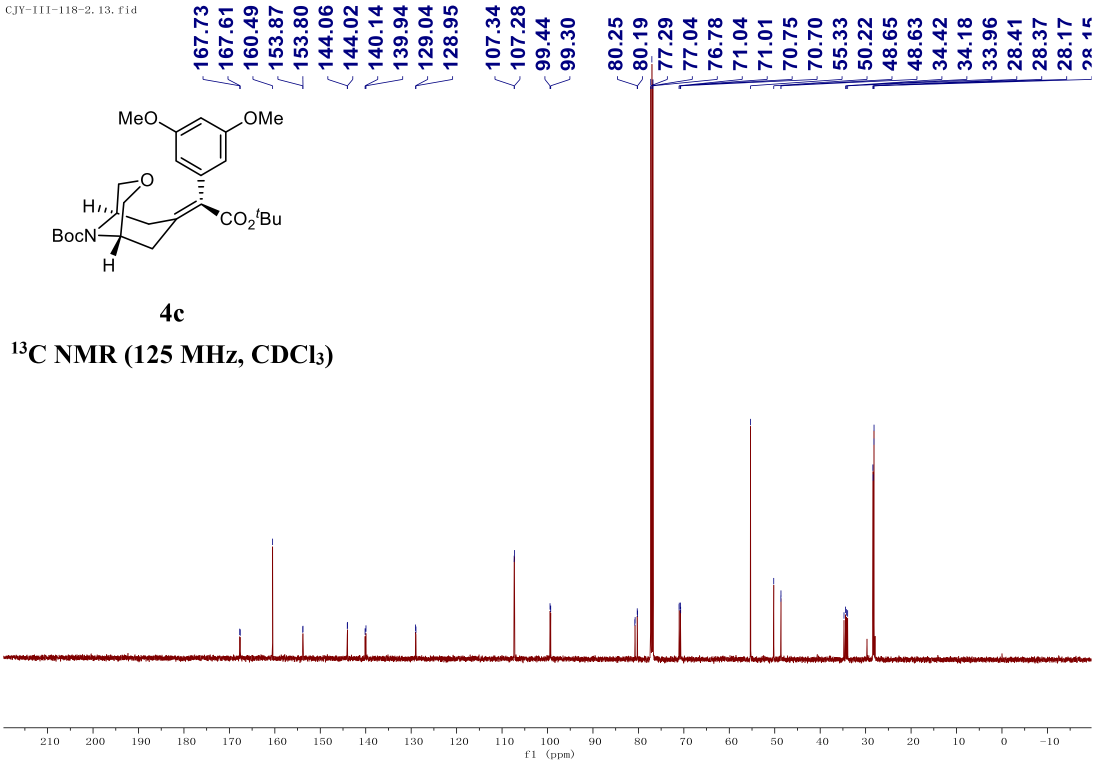

Figure S140 ^{13}C NMR (125 MHz, $CDCl_3$) spectrum for **4c**

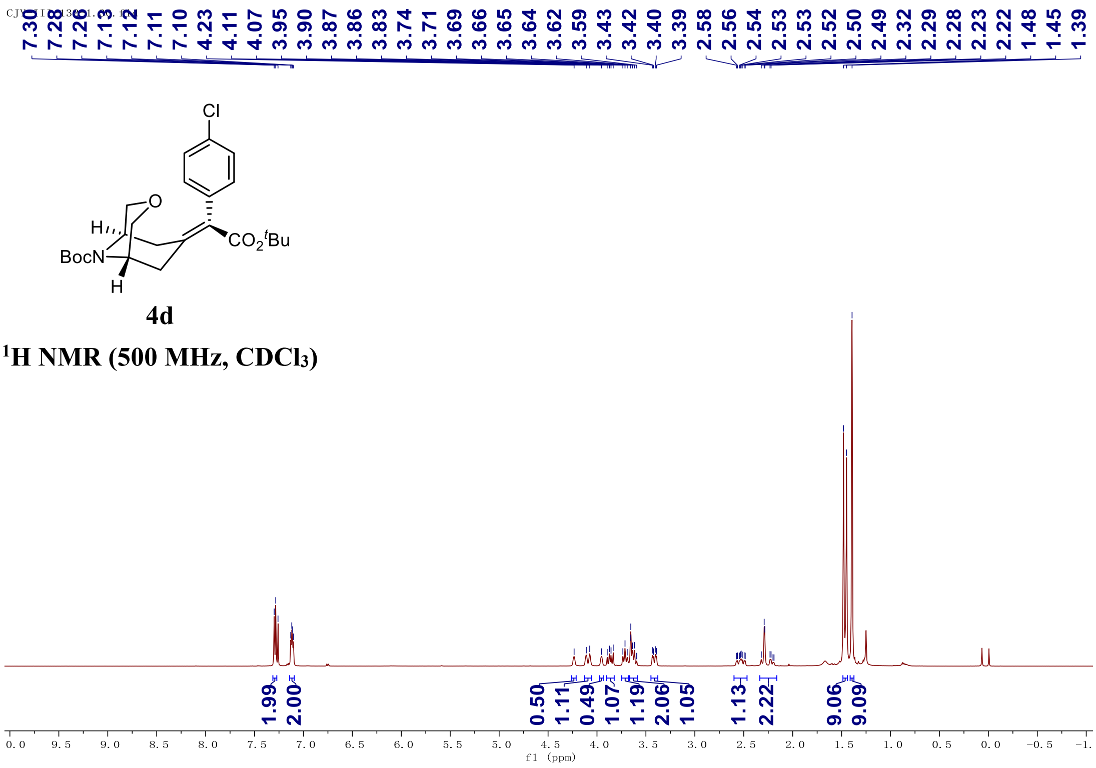

Figure S141 ^{1}H NMR (500 MHz, $CDCl_3$) spectrum for **4d**

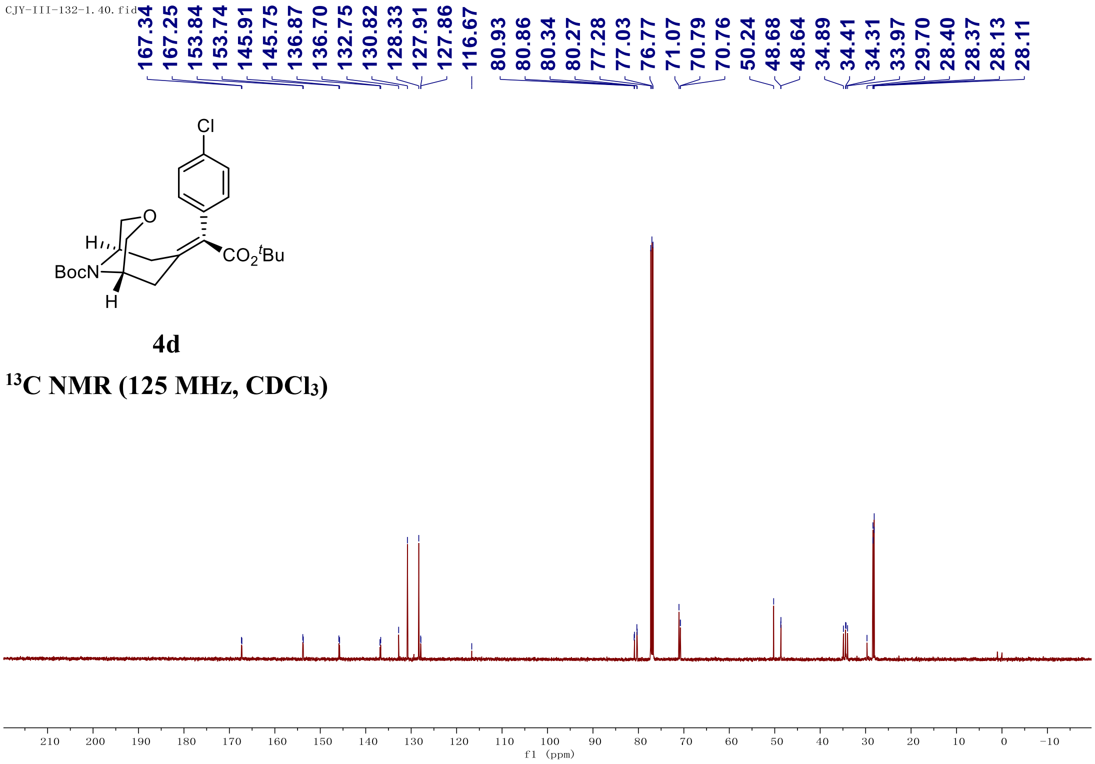

Figure S142 ^{13}C NMR (125 MHz, $CDCl_3$) spectrum for **4d**

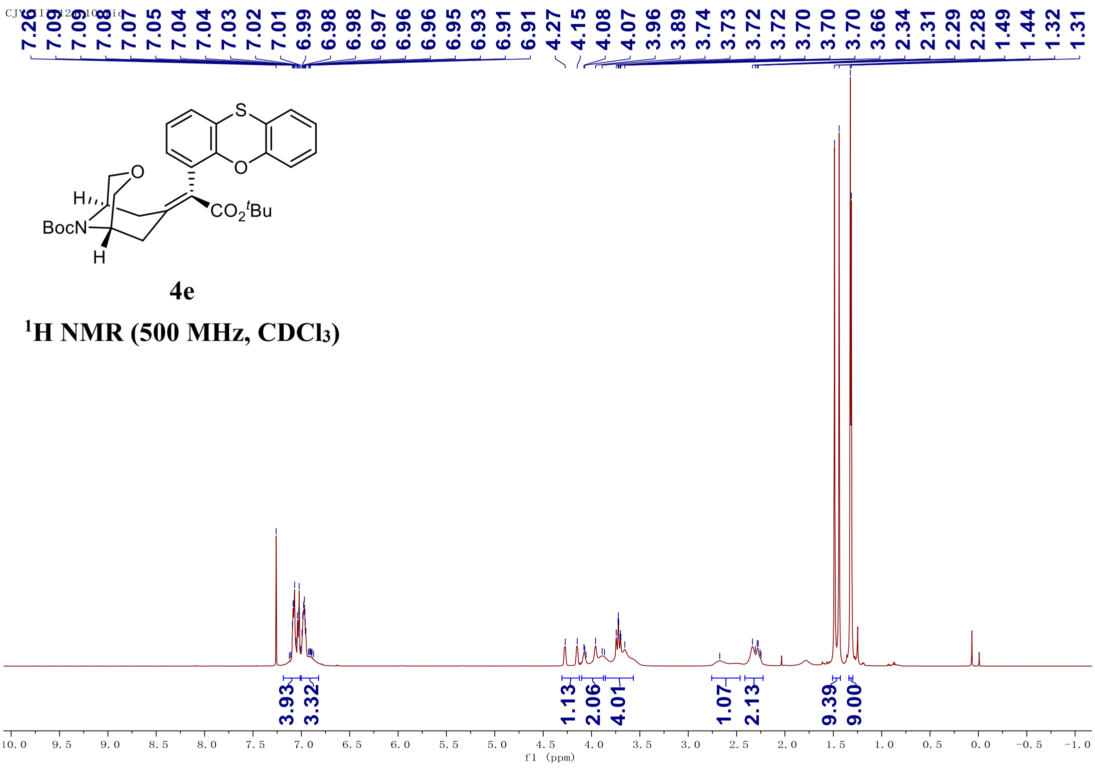

Figure S143 ^{1}H NMR (500 MHz, CDCl$_3$) spectrum for **4e**

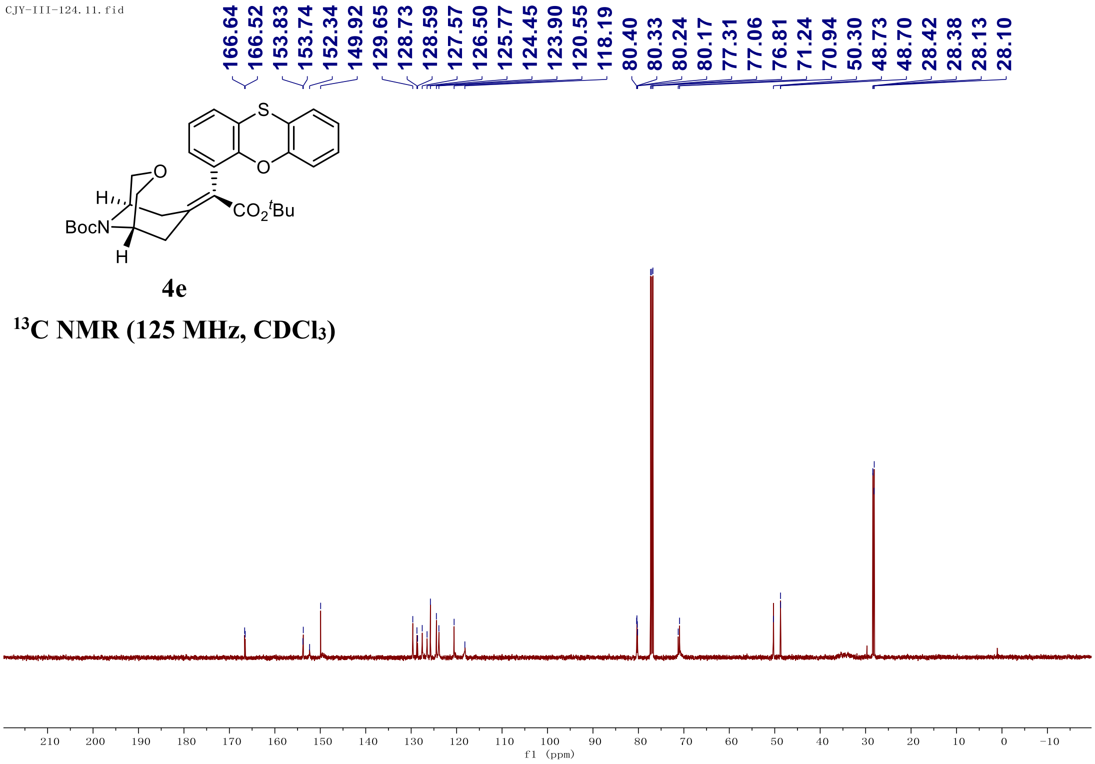

Figure S144 ^{13}C NMR (125 MHz, CDCl$_3$) spectrum for **4e**

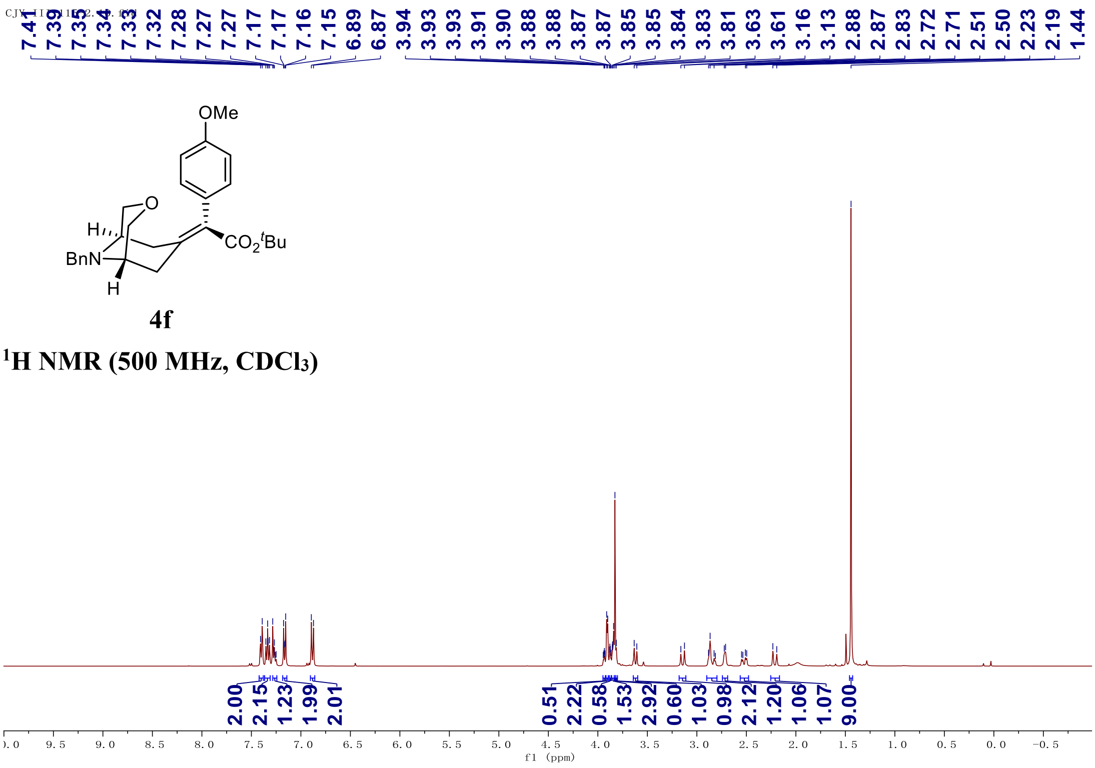

Figure S145 ^{1}H NMR (500 MHz, $CDCl_3$) spectrum for **4f**

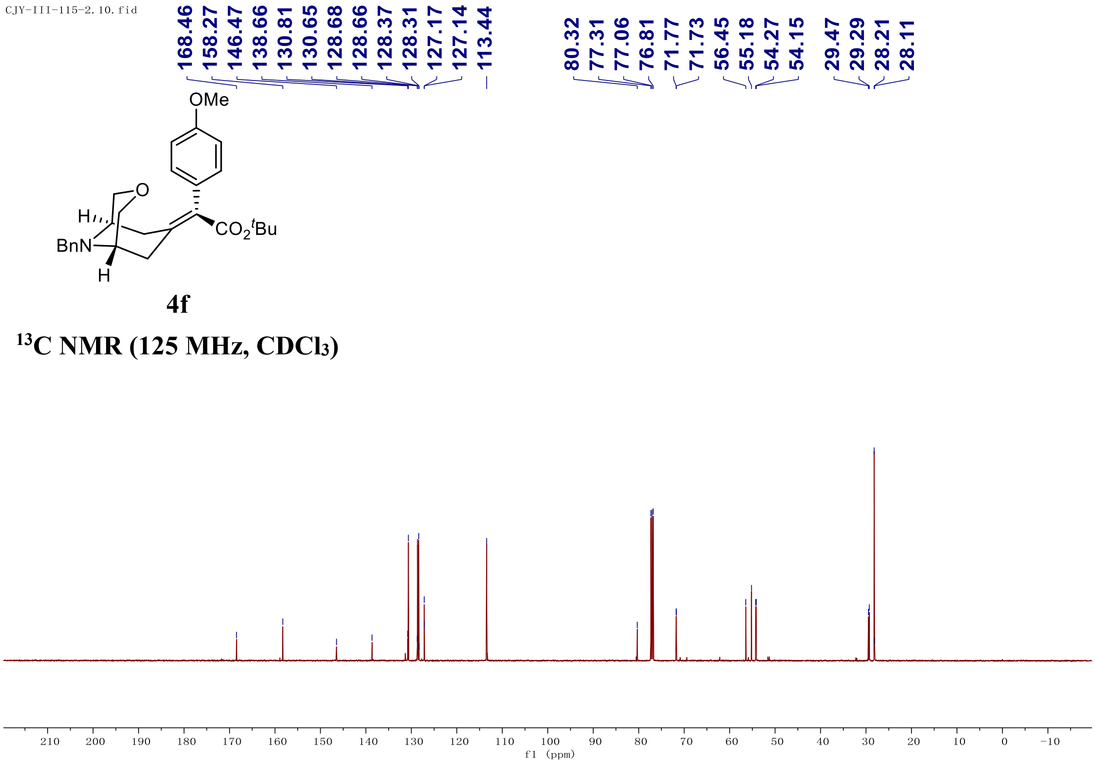

Figure S146 ^{13}C NMR (125 MHz, $CDCl_3$) spectrum for **4f**

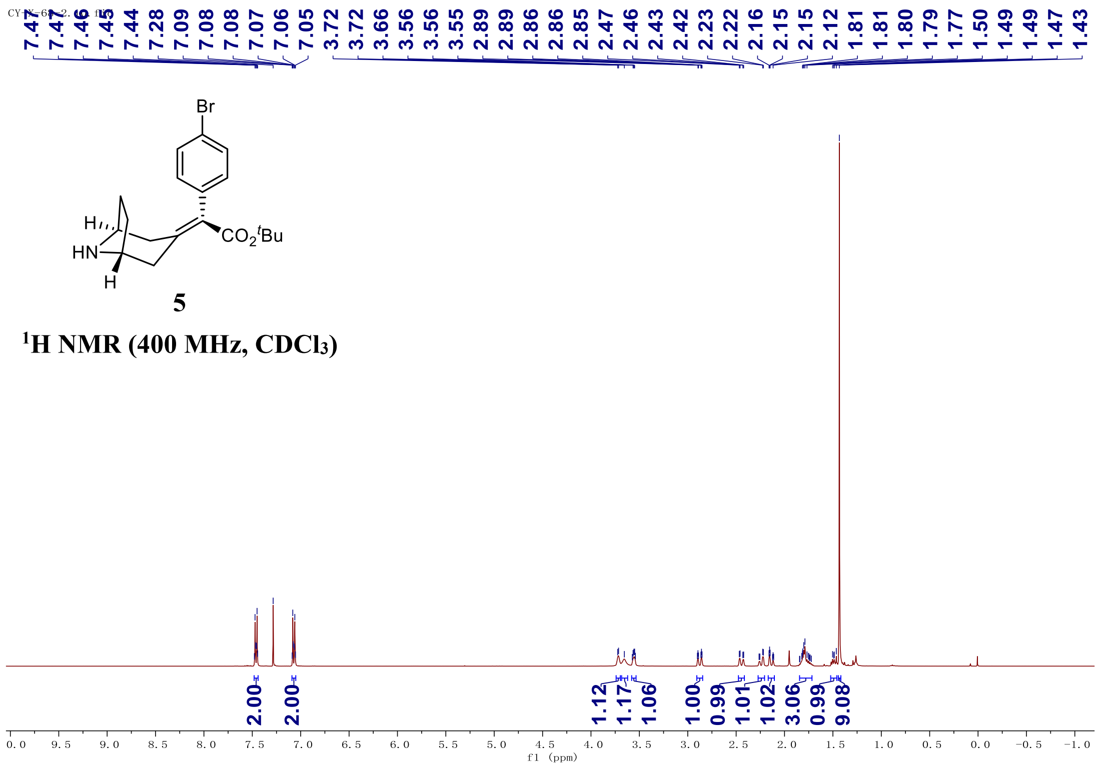

Figure S147 ^{1}H NMR (400 MHz, $CDCl_3$) spectrum for **5**

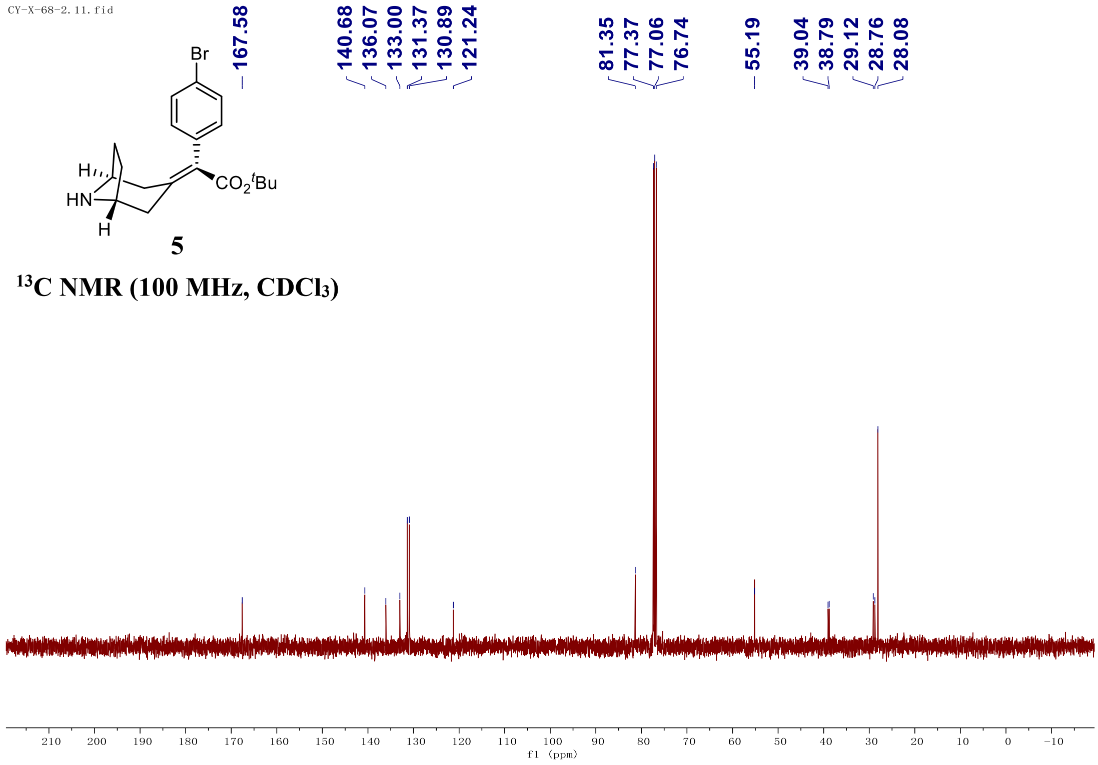

Figure S148 ^{13}C NMR (100 MHz, $CDCl_3$) spectrum for **5**

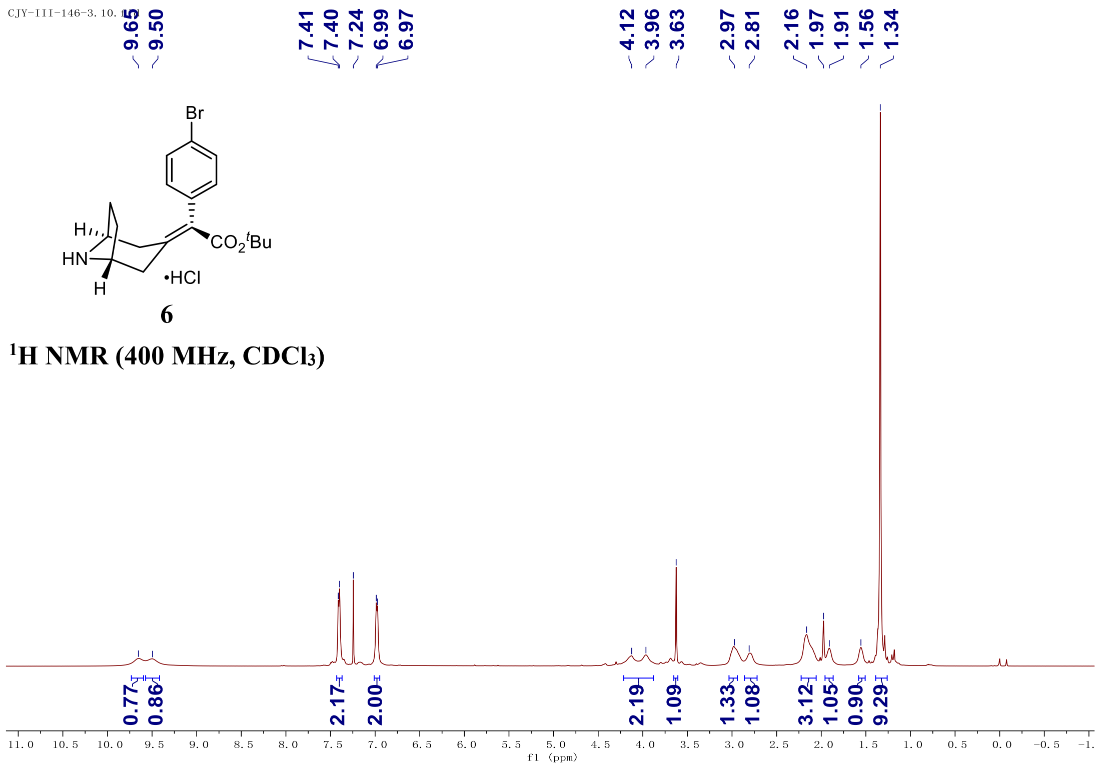

Figure S149 ^{1}H NMR (400 MHz, $CDCl_3$) spectrum for **6**

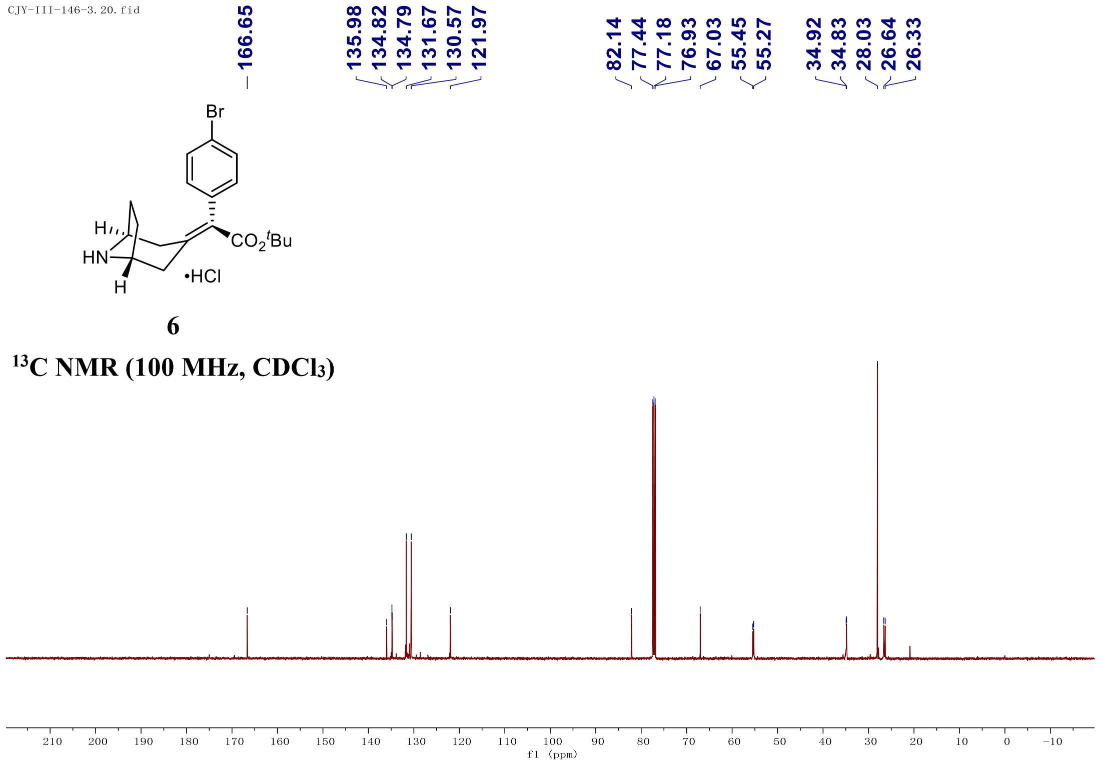

Figure S150 ^{13}C NMR (100 MHz, $CDCl_3$) spectrum for **6**

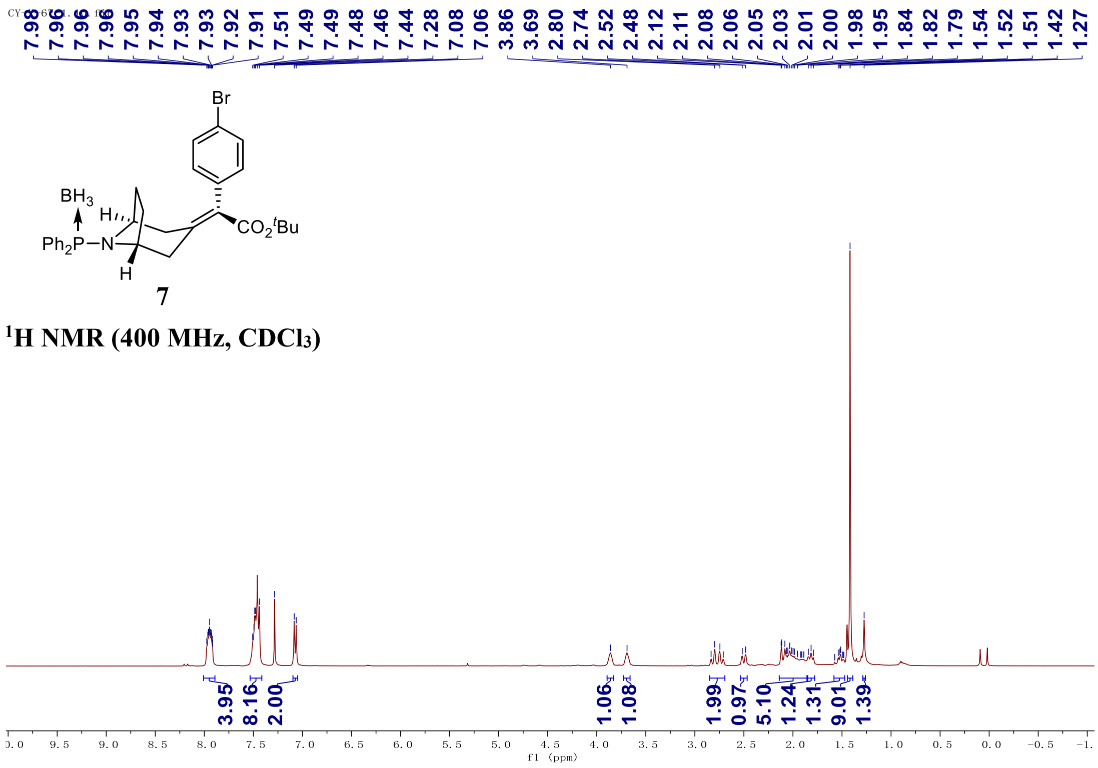

Figure S151 ^{1}H NMR (400 MHz, $CDCl_3$) spectrum for **7**

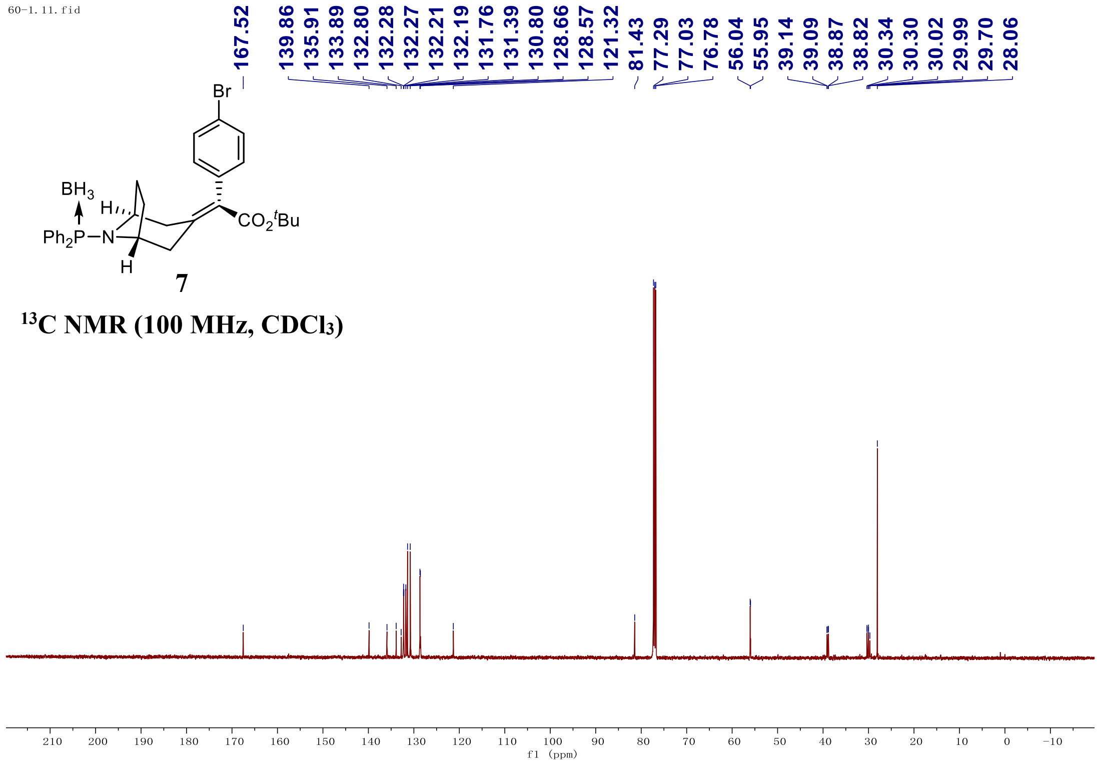

Figure S152 ^{13}C NMR (100 MHz, $CDCl_3$) spectrum for **7**

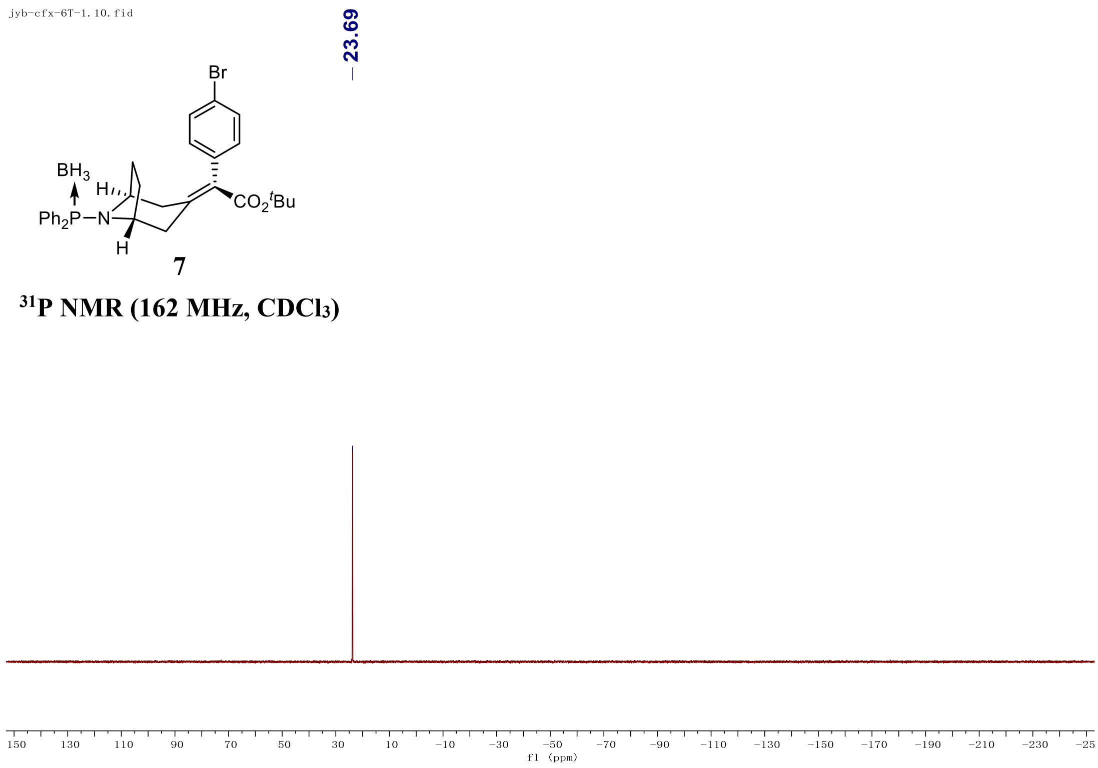

Figure S153 ^{31}P NMR (162 MHz, $CDCl_3$) spectrum for **7**

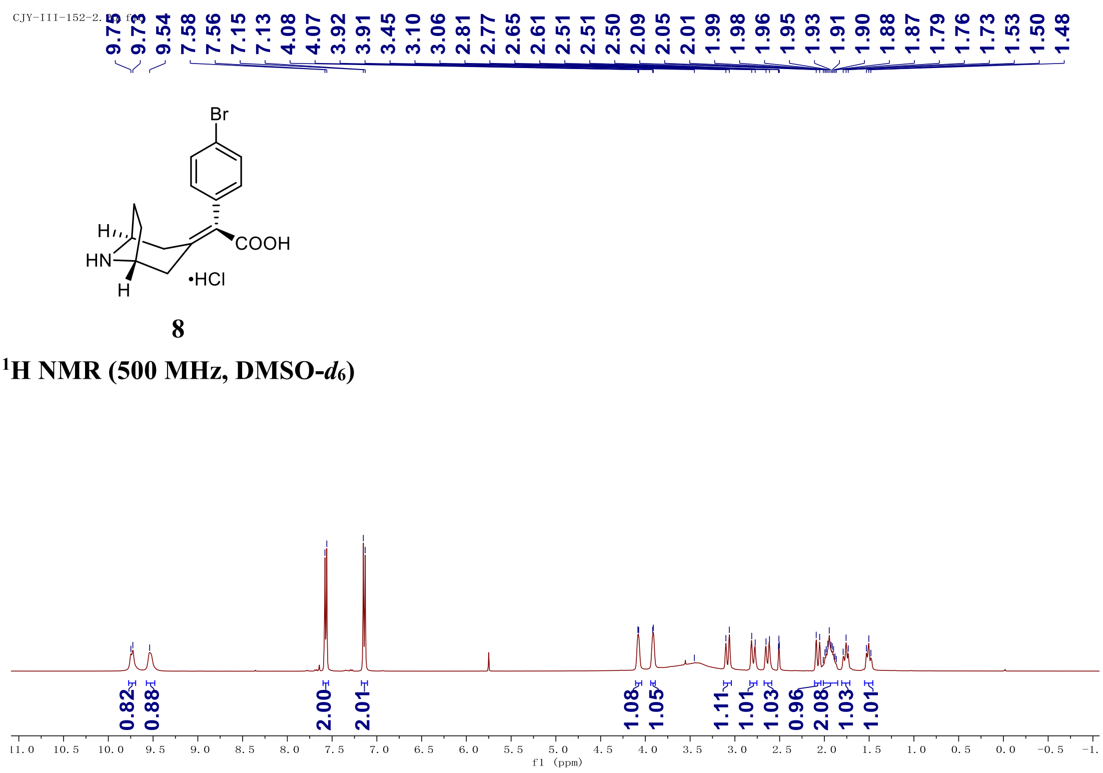

Figure S154 ^{1}H NMR (500 MHz, DMSO-d_6) spectrum for **8**

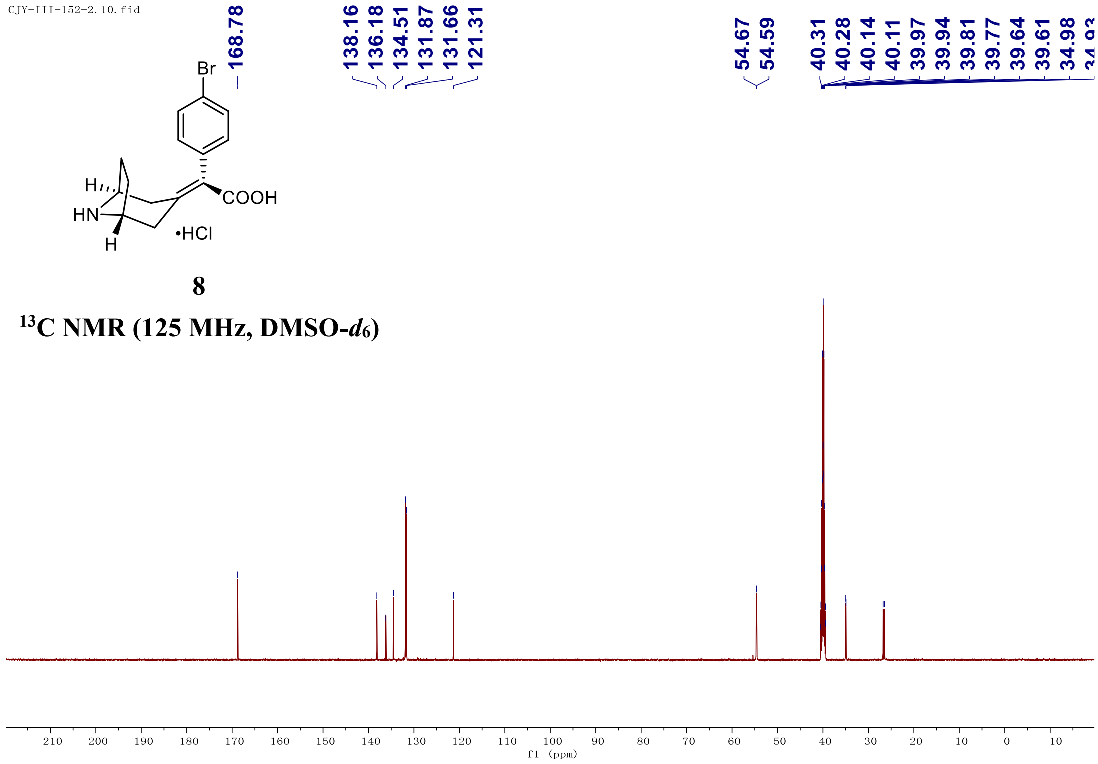

Figure S155 ^{13}C NMR (125 MHz, DMSO-d_6) spectrum for **8**

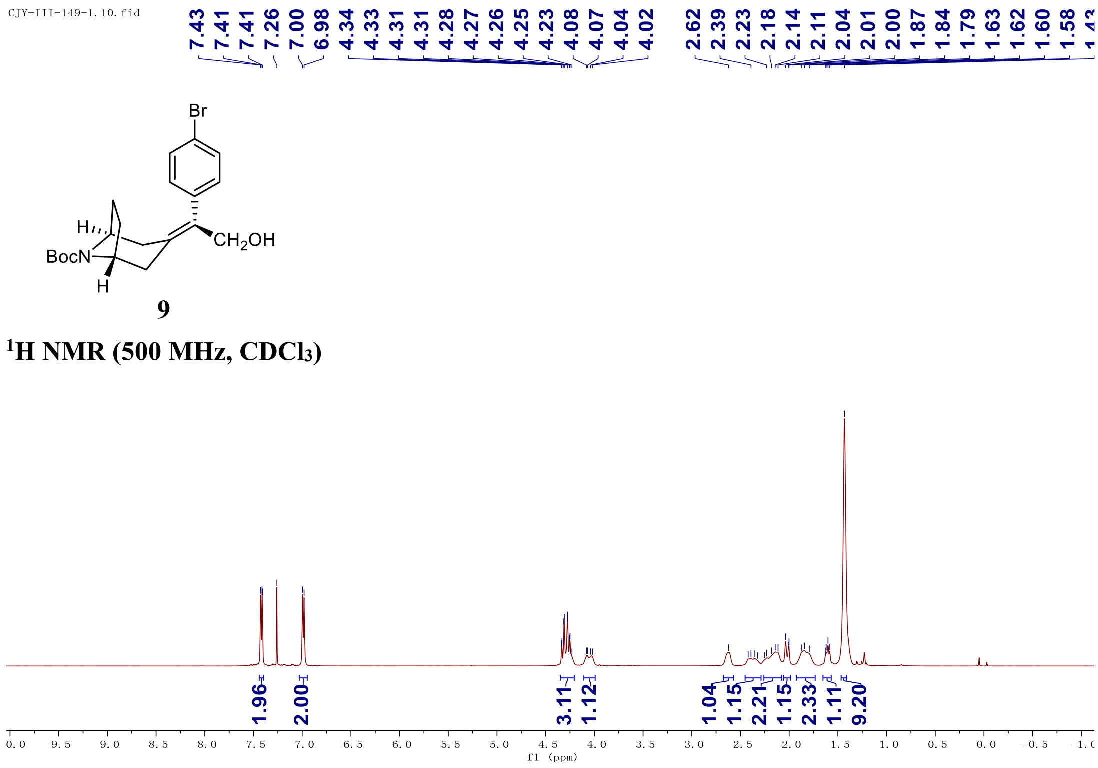

Figure S156 ^{1}H NMR (500 MHz, CDCl$_3$) spectrum for **9**

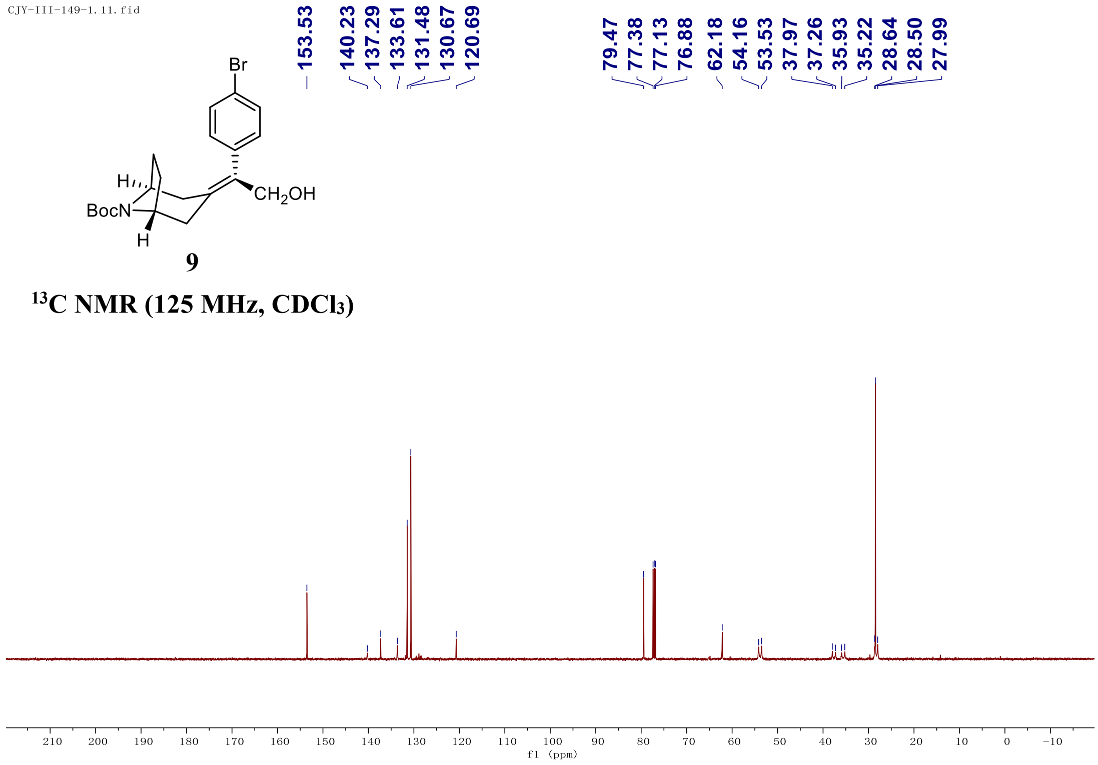

Figure S157 ^{13}C NMR (125 MHz, CDCl$_3$) spectrum for **9**

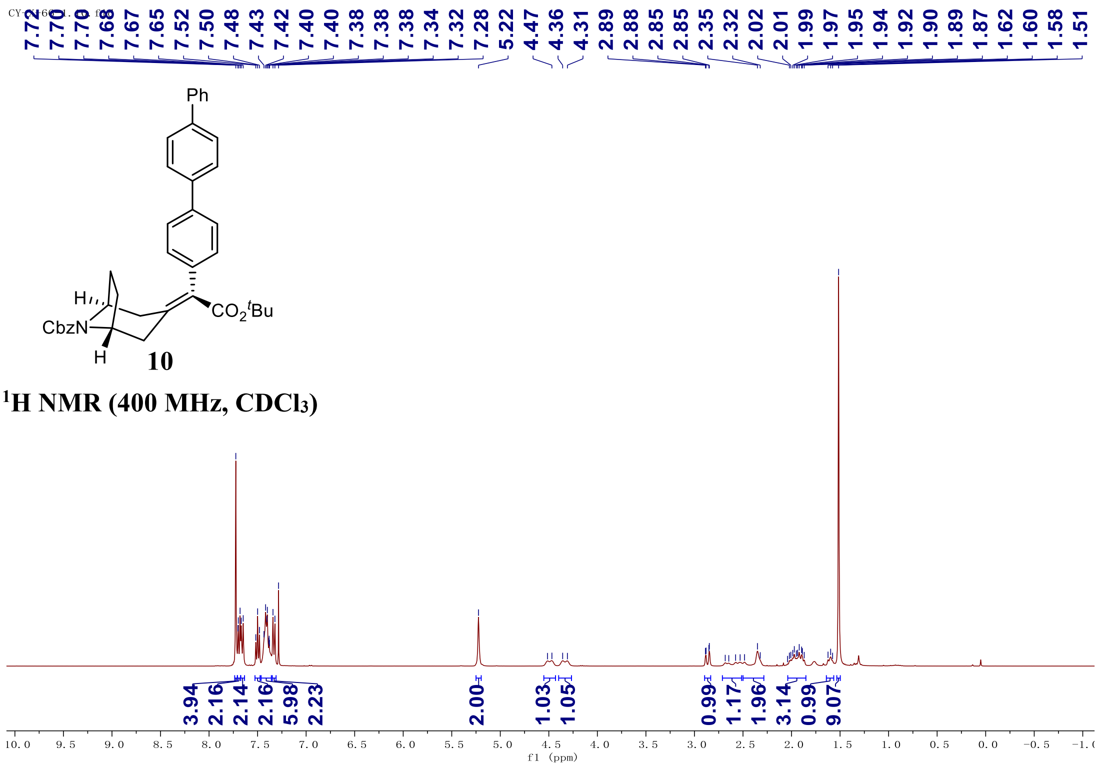

Figure S158 ^{1}H NMR (400 MHz, $CDCl_3$) spectrum for **10**

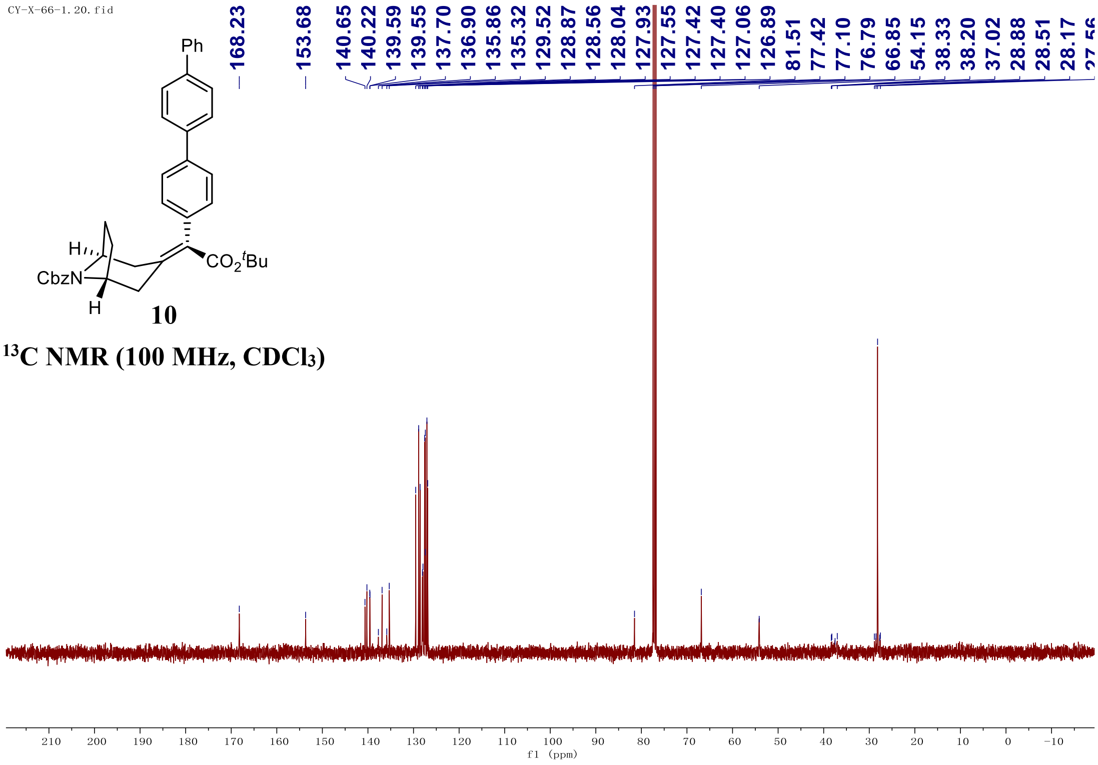

Figure S159 ^{13}C NMR (100 MHz, $CDCl_3$) spectrum for **10**

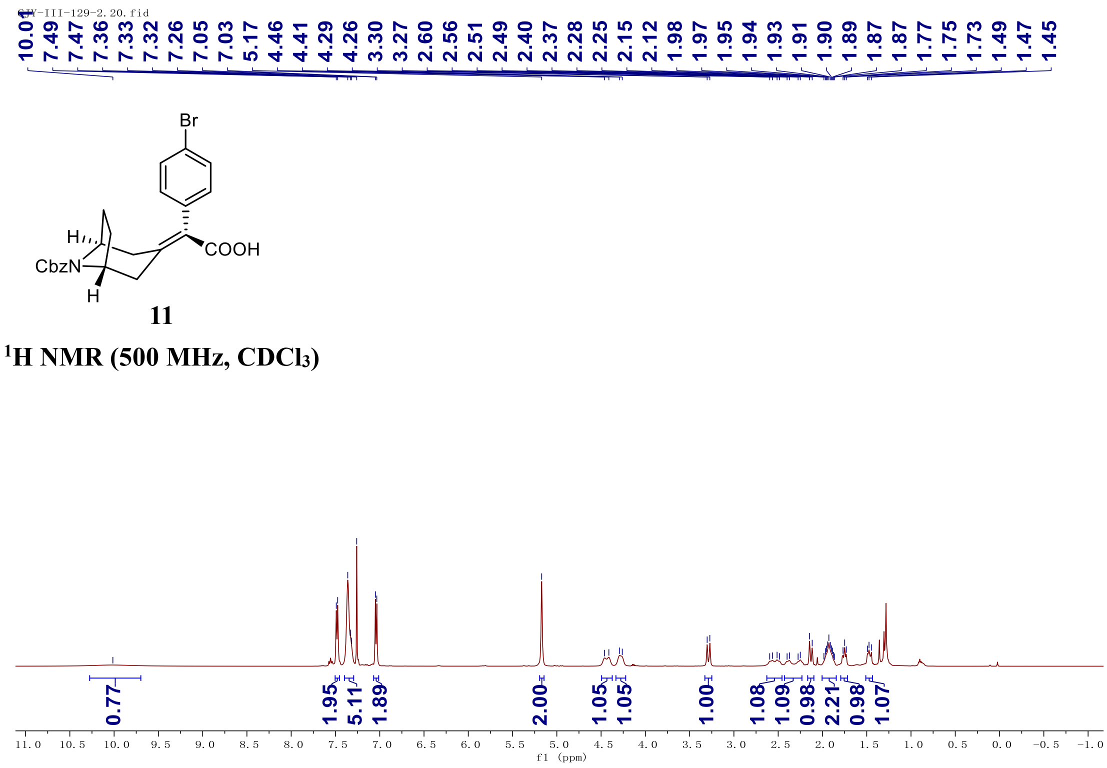

Figure S160 ^{1}H NMR (500 MHz, $CDCl_3$) spectrum for **11**

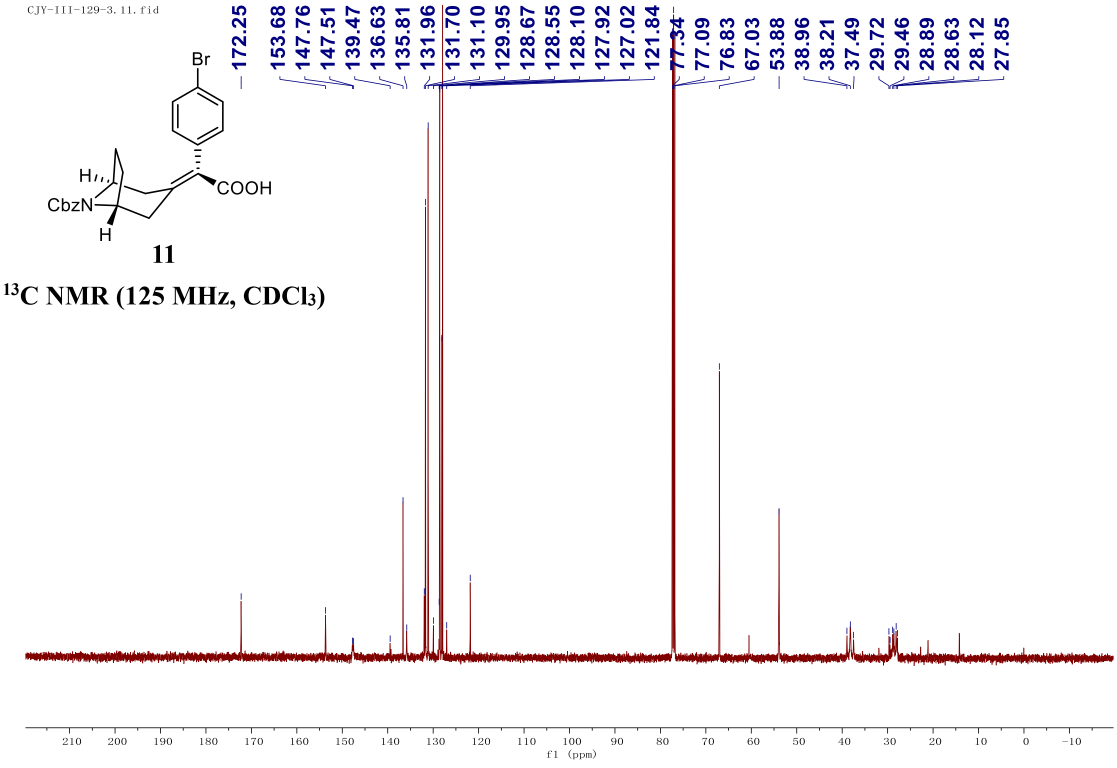

Figure S161 ^{13}C NMR (125 MHz, $CDCl_3$) spectrum for **11**

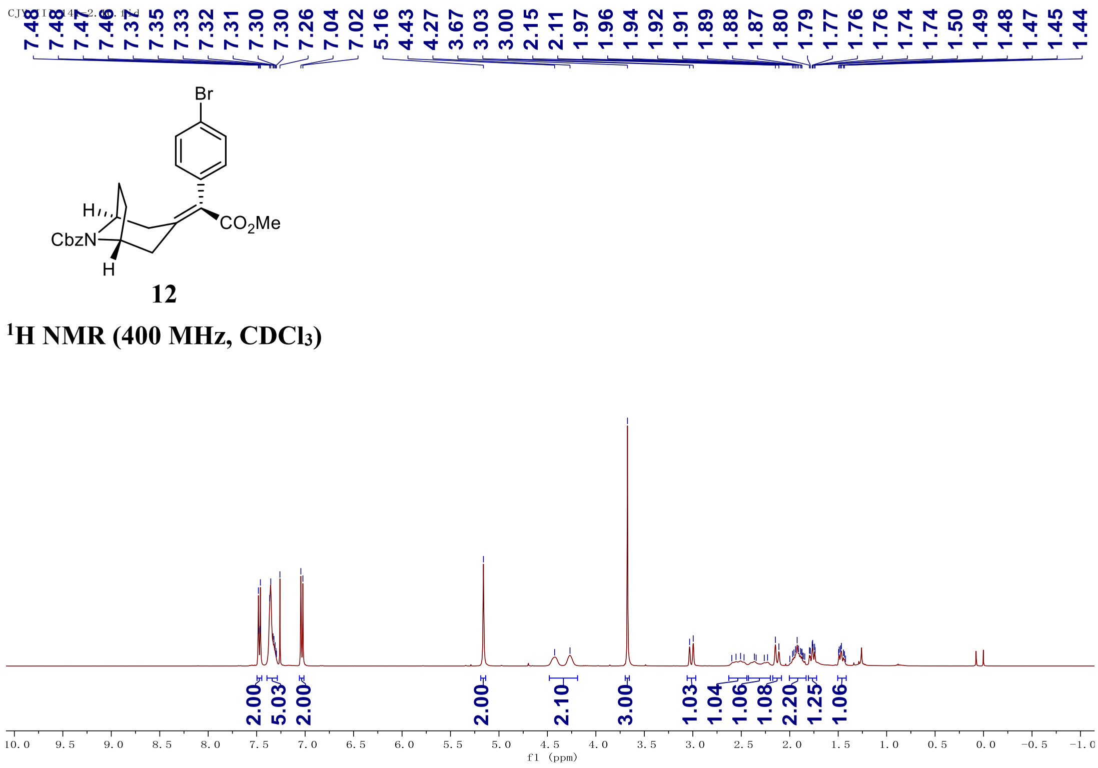

Figure S162 ^{1}H NMR (400 MHz, $CDCl_3$) spectrum for **12**

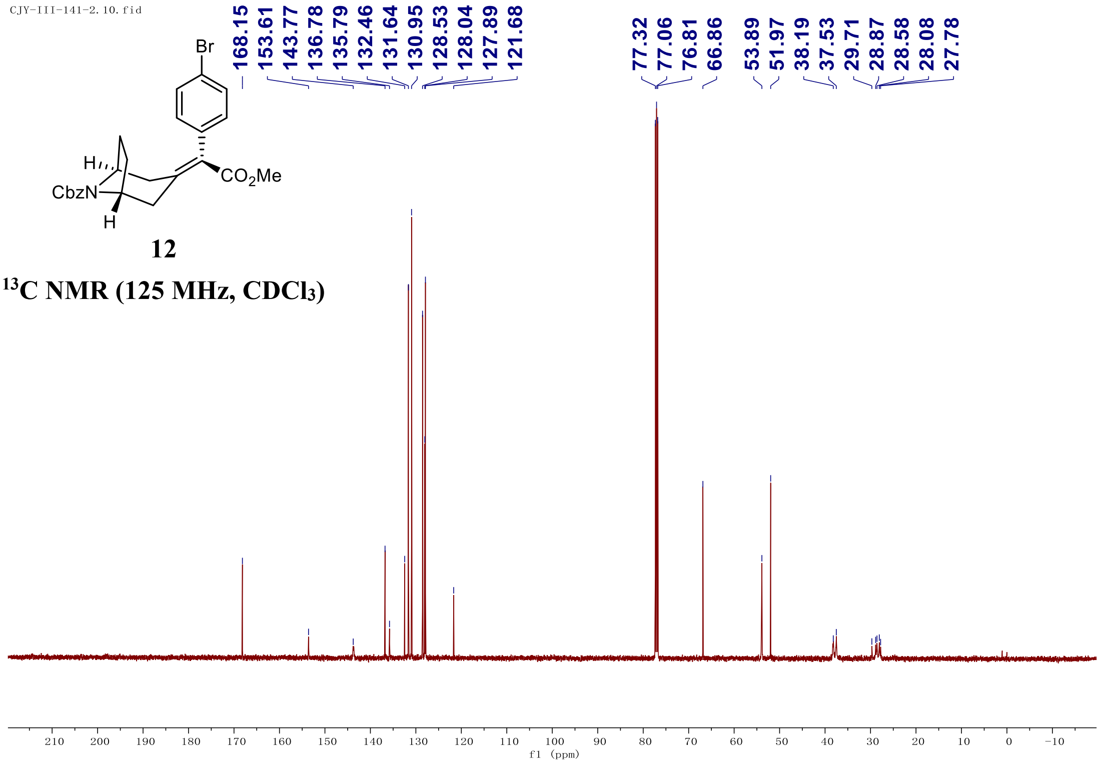

Figure S163 ^{13}C NMR (125 MHz, $CDCl_3$) spectrum for **12**

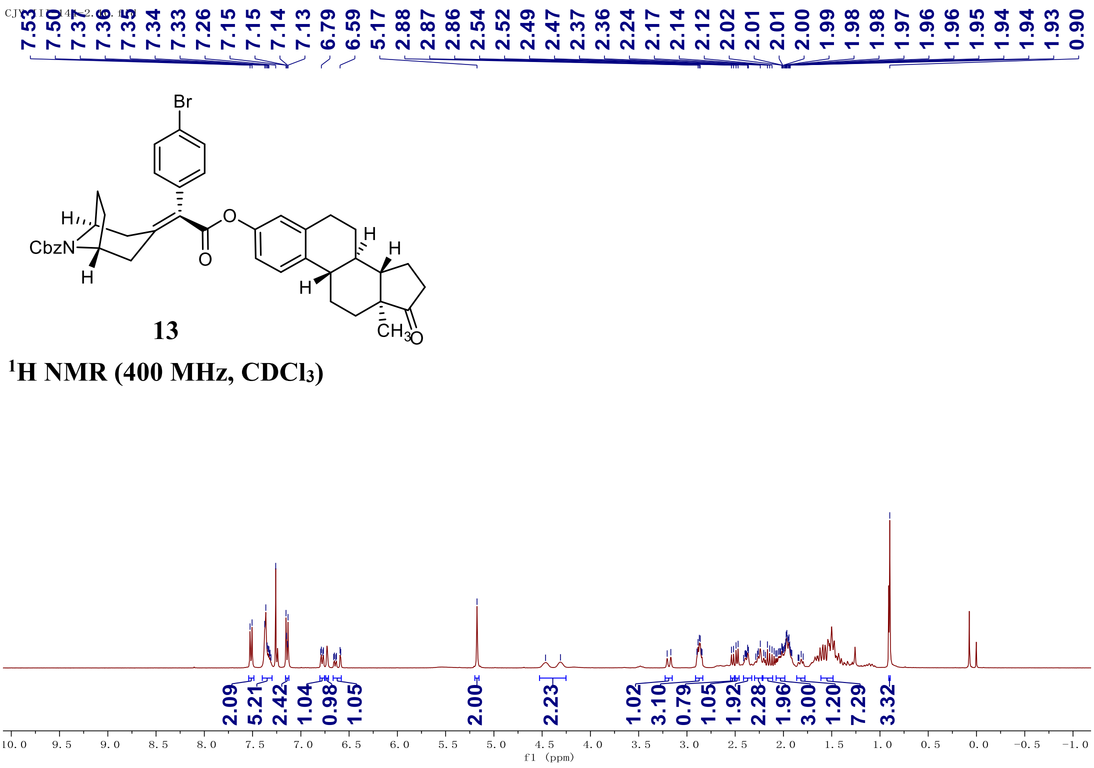

Figure S164 ^{1}H NMR (400 MHz, CDCl$_3$) spectrum for **13**

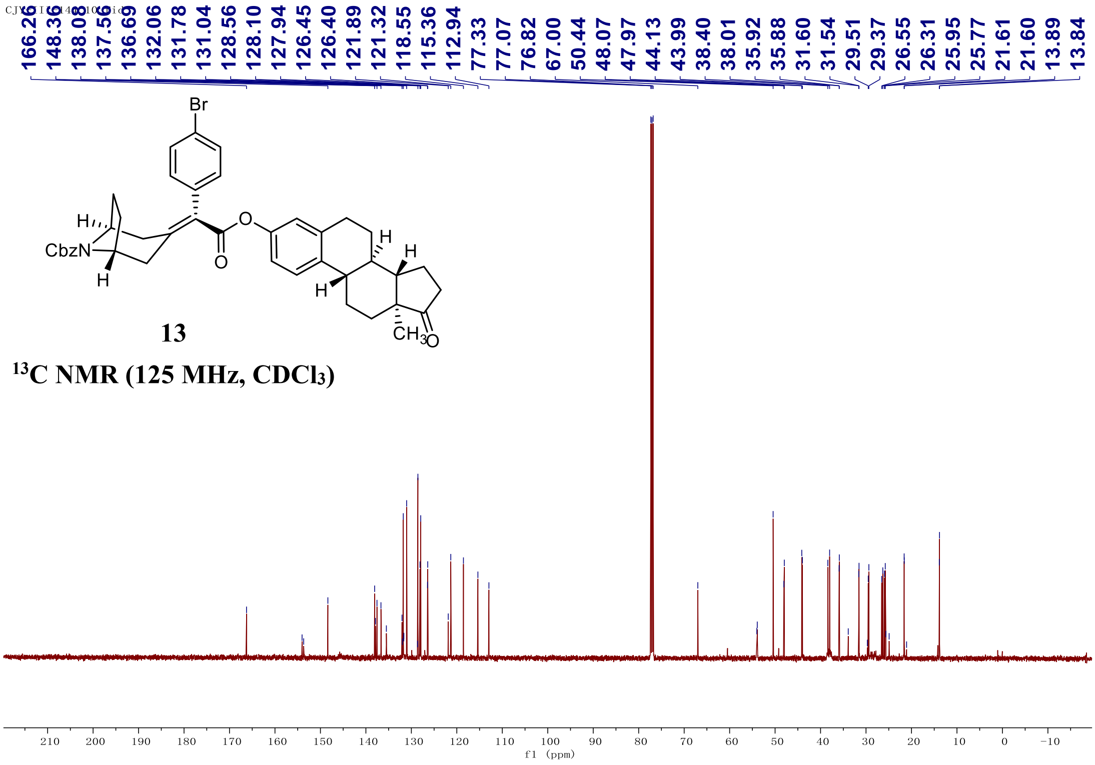

Figure S165 ^{13}C NMR (125 MHz, CDCl$_3$) spectrum for **13**

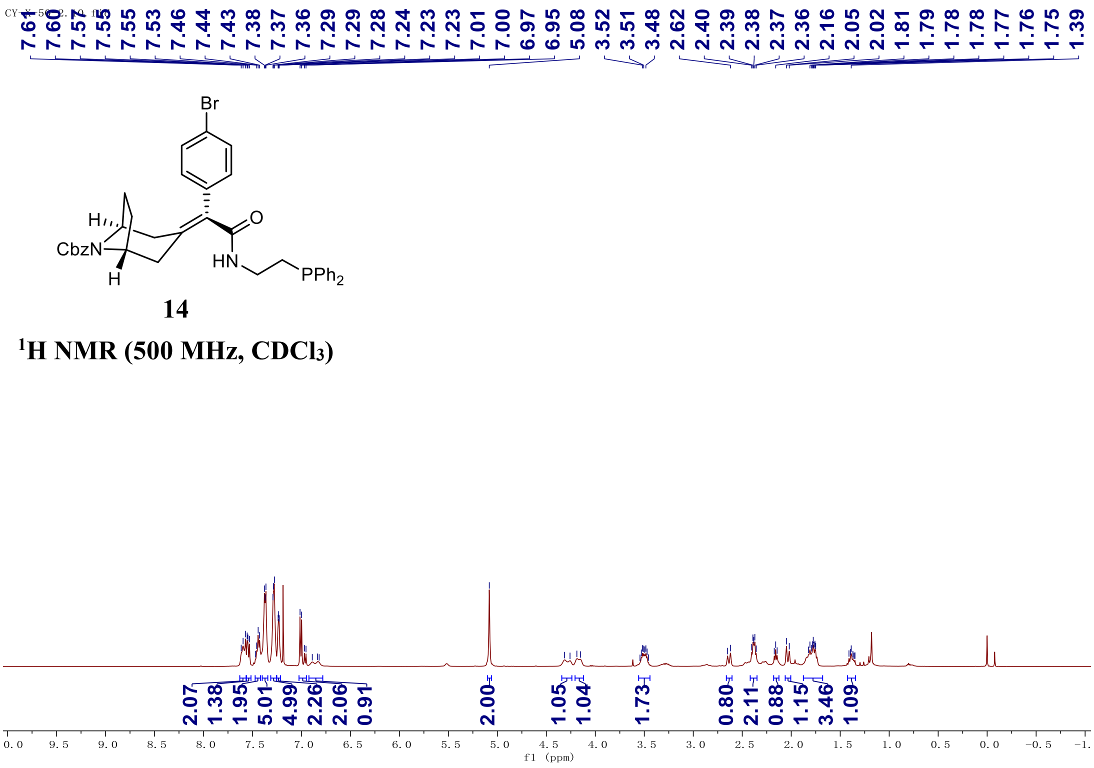

Figure S166 ^{1}H NMR (500 MHz, $CDCl_3$) spectrum for **14**

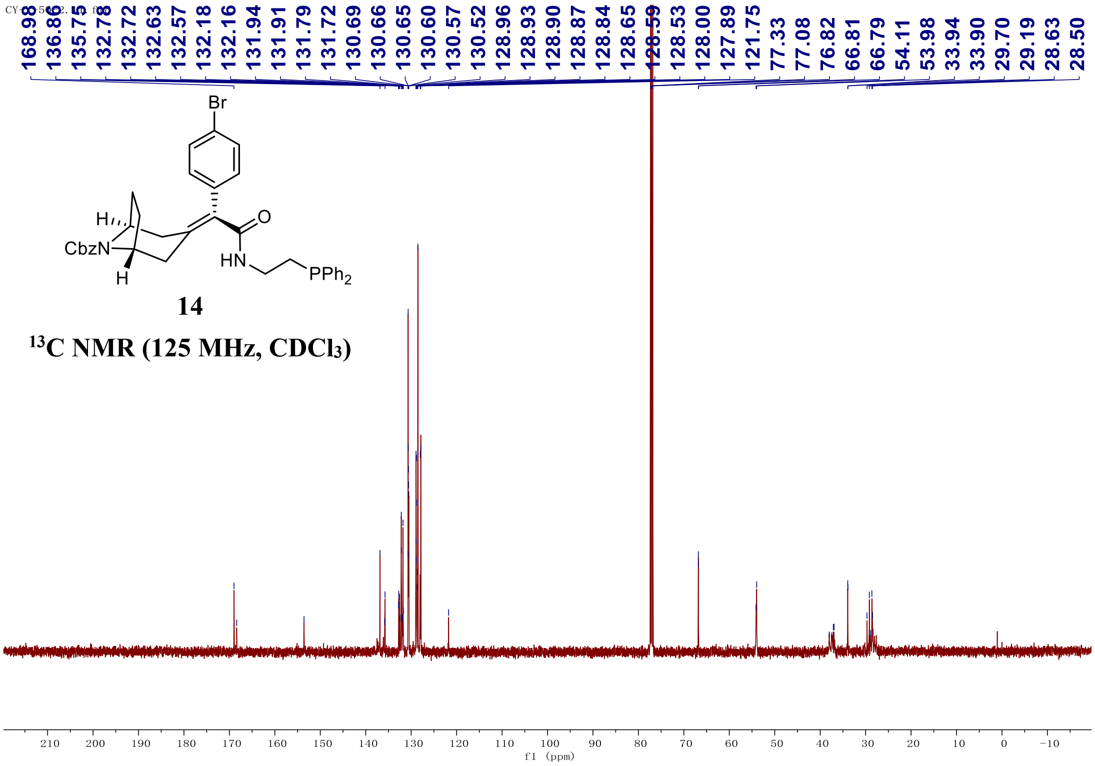

Figure S167 ^{13}C NMR (125 MHz, $CDCl_3$) spectrum for **14**

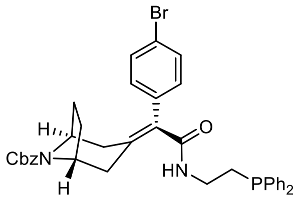

14

^{31}P NMR (162 MHz, CDCl$_3$)

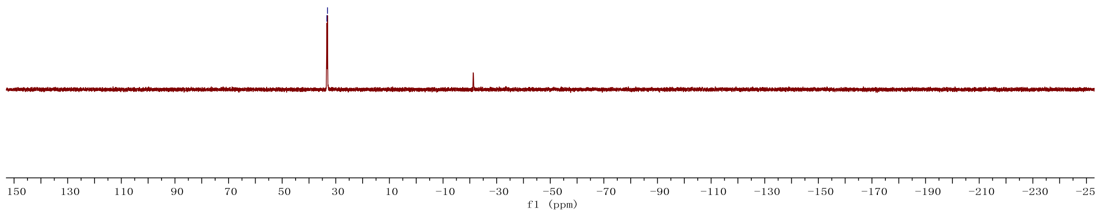

Figure S168 ^{31}P NMR (162 MHz, CDCl$_3$) spectrum for **14**
