# Supplementary material for: Rh(I)-Catalyzed Modular Synthesis of Axially Chiral Alkylidene Azacycloalkanes
Source: ACS Cent Sci. 2025 May 11;11(6):899–909. doi: 10.1021/acscentsci.5c00232 (PMC12203264; doi:10.1021/acscentsci.5c00232)

checkCIF/PLATON report

Structure factors have been supplied for datablock(s) cjy

THIS REPORT IS FOR GUIDANCE ONLY. IF USED AS PART OF A REVIEW PROCEDURE FOR PUBLICATION, IT SHOULD NOT REPLACE THE EXPERTISE OF AN EXPERIENCED CRYSTALLOGRAPHIC REFEREE.

No syntax errors found. CIF dictionary Interpreting this report

Datablock: cjy

Bond precision:	C-C = 0.0078 A		Wavelength=1.54178
Cell:	a=6.3008(4)	b=8.5061(5)	c=19.2716(12)
	alpha=90	beta=96.221(3)	gamma=90
Temperature:	200 K		

	Calculated	Reported
Volume	1026.78(11)	1026.78(11)
Space group	P 21	P 1 21 1
Hall group	P 2yb	P 2yb
Moiety formula	C23 H22 Br N O4	C23 H22 Br N O4
Sum formula	C23 H22 Br N O4	C23 H22 Br N O4
Mr	456.32	456.32
Dx,g cm-3	1.476	1.476
Z	2	2
Mu (mm-1)	2.975	2.975
F000	468.0	468.0
F000'	467.88	
h,k,lmax	7,10,23	0,0,0
Nref	3756[2016]	1995
Tmin,Tmax	0.735,0.788	0.258,0.753
Tmin'	0.666	

Correction method= # Reported T Limits: Tmin=0.258 Tmax=0.753
AbsCorr = MULTI-SCAN

Data completeness= 0.99/0.53 Theta(max)= 67.990

R(reflections)= 0.0800(991) wR2(reflections)= 0.2066(1995)

S = 0.849 Npar= 263

The following ALERTS were generated. Each ALERT has the format
test-name_ALERT_alert-type_alert-level.
Click on the hyperlinks for more details of the test.

Alert level C

```
PLAT090_ALERT_3_C Poor Data / Parameter Ratio (Zmax > 18) ........       7.59 Note
PLAT230_ALERT_2_C Hirshfeld Test Diff for    C1       --C2       .        5.5 s.u.
PLAT234_ALERT_4_C Large Hirshfeld Difference C4       --C5       .       0.18 Ang.
PLAT341_ALERT_3_C Low Bond Precision on  C-C Bonds ..............    0.00778 Ang.
PLAT906_ALERT_3_C Large K Value in the Analysis of Variance ......      3.838 Check
PLAT911_ALERT_3_C Missing FCF Refl Between Thmin & STh/L=    0.600         20 Report
                 1 10  0,  -1 10  1,   1 10  1,  -1 10  2,   1 10  3,   5  7  4,
                 3  9  4,   7  0  5,   7  1  5,   2  9  8,  -6  4 12,   4  6 12,
                -6  0 13,  -5  5 14,  -1  8 14,  -4  0 20,   3  0 20,   2  0 21,
                 2  1 21,   0  4 21,
PLAT914_ALERT_3_C No Bijvoet Pairs in FCF for Non-centro Structure     Please Check
```

Alert level G

```
PLAT002_ALERT_2_G Number of Distance or Angle Restraints on AtSite          6 Note
PLAT003_ALERT_2_G Number of Uiso or U(i,j) Restrained non-H Atoms           4 Report
PLAT007_ALERT_5_G Number of Unrefined Donor-H Atoms .............           1 Report
              H4
PLAT012_ALERT_1_G N.O.K.   _shelx_res_checksum Found in CIF ......     Please Check
PLAT072_ALERT_2_G SHELXL First  Parameter in WGHT  Unusually Large       0.14 Report
PLAT172_ALERT_4_G The CIF-Embedded .res File Contains DFIX Records          1 Report
PLAT178_ALERT_4_G The CIF-Embedded .res File Contains SIMU Records          2 Report
PLAT188_ALERT_3_G A Non-default SIMU Restraint Value has been used     0.0008 Report
PLAT188_ALERT_3_G A Non-default SIMU Restraint Value has been used     0.0008 Report
PLAT720_ALERT_4_G Number of Unusual/Non-Standard Labels .........           3 Note
              C00F    H00A    H00B
PLAT791_ALERT_4_G Model has Chirality at C9         (Sohncke SpGr)          S Verify
PLAT791_ALERT_4_G Model has Chirality at C12        (Sohncke SpGr)          R Verify
PLAT860_ALERT_3_G Number of Least-Squares Restraints ............          19 Note
PLAT912_ALERT_4_G Missing # of FCF Reflections Above STh/L=  0.600          1 Note
PLAT941_ALERT_3_G Average HKL Measurement Multiplicity ..........         1.0 Low
PLAT961_ALERT_5_G Dataset Contains no Negative Intensities .......     Please Check
PLAT969_ALERT_5_G The 'Henn et al.' R-Factor-gap value ..........       1.982 Note
              Predicted wR2: Based on SigI**2 10.42 or SHELX Weight 24.34
PLAT978_ALERT_2_G Number C-C Bonds with Positive Residual Density.          0 Info
PLAT992_ALERT_5_G Repd & Actual _reflns_number_gt Values Differ by          2 Check
```

0 **ALERT level A** = Most likely a serious problem - resolve or explain
0 **ALERT level B** = A potentially serious problem, consider carefully
7 **ALERT level C** = Check. Ensure it is not caused by an omission or oversight
19 **ALERT level G** = General information/check it is not something unexpected

1 ALERT type 1 CIF construction/syntax error, inconsistent or missing data
5 ALERT type 2 Indicator that the structure model may be wrong or deficient
9 ALERT type 3 Indicator that the structure quality may be low
7 ALERT type 4 Improvement, methodology, query or suggestion
4 ALERT type 5 Informative message, check

It is advisable to attempt to resolve as many as possible of the alerts in all categories. Often the minor alerts point to easily fixed oversights, errors and omissions in your CIF or refinement strategy, so attention to these fine details can be worthwhile. In order to resolve some of the more serious problems it may be necessary to carry out additional measurements or structure refinements. However, the purpose of your study may justify the reported deviations and the more serious of these should normally be commented upon in the discussion or experimental section of a paper or in the "special_details" fields of the CIF. checkCIF was carefully designed to identify outliers and unusual parameters, but every test has its limitations and alerts that are not important in a particular case may appear. Conversely, the absence of alerts does not guarantee there are no aspects of the results needing attention. It is up to the individual to critically assess their own results and, if necessary, seek expert advice.

Publication of your CIF in IUCr journals

A basic structural check has been run on your CIF. These basic checks will be run on all CIFs submitted for publication in IUCr journals (*Acta Crystallographica*, *Journal of Applied Crystallography*, *Journal of Synchrotron Radiation*); however, if you intend to submit to *Acta Crystallographica Section C* or *E* or *IUCrData*, you should make sure that full publication checks are run on the final version of your CIF prior to submission.

Publication of your CIF in other journals

Please refer to the *Notes for Authors* of the relevant journal for any special instructions relating to CIF submission.

PLATON version of 15/07/2024; check.def file version of 15/07/2024

Datablock cjy - ellipsoid plot

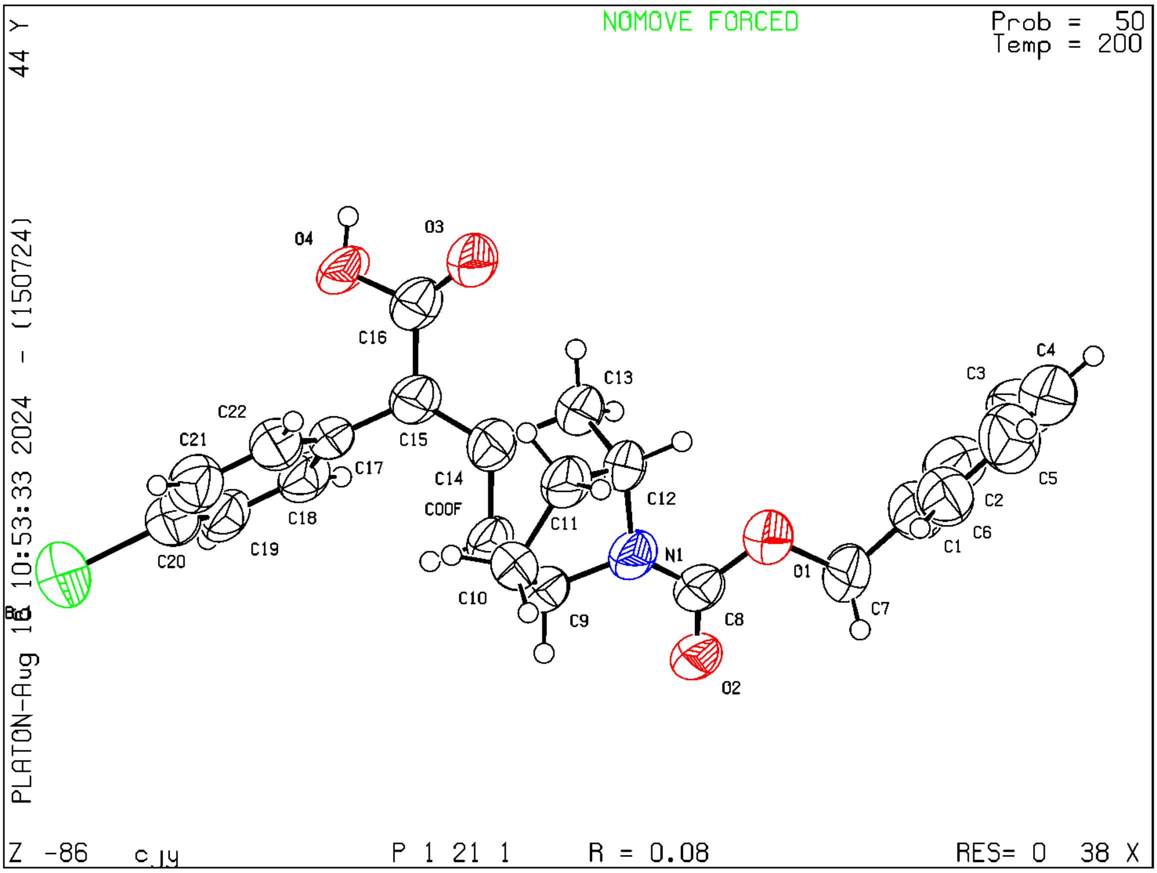

Supplement: Supplementary file 3 [file oc5c00232_si_003.pdf]
